# Kunxinning granules alleviate perimenopausal syndrome by supplementing estrogen deficiency

**DOI:** 10.3389/fphar.2025.1554479

**Published:** 2025-03-26

**Authors:** Wenshuang Wang, Wen Yang, Fangwenting Wang, He Gao, Kaixin Liu, Jinling Zhang, Yunjuan Li, Man Zhang, Guirong Zhou, Yuanyuan Hou, Gang Bai

**Affiliations:** ^1^ State Key Laboratory of Medicinal Chemical Biology, College of Pharmacy and Tianjin Key Laboratory of Molecular Drug Research, Nankai University, Tianjin, China; ^2^ State Key Laboratory of Chinese Medicine Modernization, Tasly Pharmaceutical Group Co., Ltd., Tianjin, China; ^3^ Tianjin Key Laboratory of Component-Based Chinese Medicine, Tianjin, China

**Keywords:** Kunxinning Granules, perimenopausal syndrome, hormone disorders, steroid hormone biosynthesis pathway, molecular network, multi omics analysis

## Abstract

**Introduction:**

Ovarian function decline results in reduced estrogen levels, leading to endocrine disorders, oxidative stress damage, and excessive activation of inflammatory factors, all of which contribute to the development of premenstrual syndrome (PMS). Kunxinning Granules (KXN) has been clinically approved for PMS treatment, but its bioactive ingredients and mechanism of action remain unclear. This study aimed to investigate the active metabolites and molecular mechanism of KXN in treating PMS rats, laying a foundation for the clinical development of PMS treatment.

**Methods:**

An ovariectomized (OVX) rat model was established to evaluate the efficacy of KXN in treating PMS. Molecular network (MN) analysis, combined with UPLC/Q-TOF-MS, identified prototype compounds in the samples and constructed a chemical classification map based on their structures. A network analysis and proteomics were conducted to predict potential pathways through which KXN regulates PMS. Quantitative metabolomics assays were used to confirm these potential pathways. Additionally, target prediction and binding enzyme activity detection elucidated the key active metabolites and mechanisms of action in KXN.

**Results:**

KXN exhibited significant effectiveness in supplementing estrogen deficiency and uterine atrophy in the OVX model. We identified 16 absorbed metabolites as the potential pharmacological ingredients of KXN *in vivo*. The steroid hormone biosynthesis pathway, a crucial pathway of KXN in PMS, played a key role in KXN’s effectiveness. KXN improved hormonal metabolic disorders by regulating this pathway. The main metabolites in KXN, including astragaloside IV, icariin and baohuoside I increased estradiol levels by enhancing the activity of CYP19A1, the representative enzyme in hormone biosynthesis pathway.

**Discussion:**

This study shows that KXN could relieve anxiety, depression, and osteoporosis in PMS. This pharmacological effect is exerted through steroid hormone synthesis to address estrogen deficiency. The findings provide valuable insights into the underlying mechanisms and support its clinical application.

## 1 Introduction

In the aging population, the imperative to augment women’s health during menopause is accentuated (Goriely, 2024). Menopause is a physiological transition from fertility to non-fertility in women (Davis et al., 2023). The decline of ovarian function and the accompanying hormonal imbalance, especially estrogen deficiency, are the leading causes of perimenopausal syndrome (Peacock et al., 2023). This phase precipitates a spectrum of systemic symptoms encompassing hot flashes, anxiety, depression, insomnia, lipid metabolism disorders and osteoporosis, collectively known as perimenopausal syndrome (PMS) (Davis et al., 2015). While hormone replacement therapy (HRT) has been the primary treatment for alleviating symptoms, its effectiveness is overshadowed by safety concerns, such as the heightened risk of endometrial, coronary, breast, and gallbladder issues (Cho et al., 2023; Qin et al., 2021). Consequently, more perimenopausal women are turning to complementary and alternative medicine, leading to a growing need to thoroughly explore non-hormonal treatments, particularly in the area of natural products, to ameliorate PMS.

Natural products offer a promising alternative for managing PMS, as evidenced by the therapeutic effectiveness of traditional Chinese medicine in relieving symptoms (Kargozar et al., 2017). The Qing Yan Formula regulates the biosynthesis of unsaturated fatty acids, arachidonic acid metabolism and bile secretion to restore the estrous cycle of ovariectomized (OVX) rats (Zou et al., 2024). Jiangu Granules restore bone homeostasis by modulating monocytes and the Notch signaling pathway, thereby alleviating postmenopausal osteoporosis symptoms (Li et al., 2024). Similarly, the Qing’e formula elevates serum estradiol and enhances estrogen receptor expression, helping regulate PMS-related emotional disturbances (Xiong et al., 2020).

Kunxinning Granules (KXN), originating from the botanical drug pair known as “Er Xian” in traditional Chinese medicine, is a formulated remedy designed to address PMS. “Er Xian” botanical drugs include *Curculigo orchioides* Gaertn [Amaryllidaceae; *Curculiginis Rhizoma*] and *Epimedium brevicornu* Maxim [Berberidaceae; *Epimedii Folium*] which regulate hormone disturbance (Nie et al., 2013; Sun et al., 2024). On this basis, KXN incorporates *Rehmannia glutinosa* (Gaertn.) Libosch. ex DC [Scrophulariaceae; *Rehmanniae Radix Praeparata*] and *Astragalus mongholicus* Bunge [Fabaceae; *Astragali Radix*] to enhance mitochondrial function and support energy metabolism (Li et al., 2023; Shen et al., 2024). Additional botanical drug like *Paeonia lactiflora* Pall [Paeoniaceae; *Paeoniae Radix Rubra*] and animal drug like *Haliotis* [Haliotidae; *Haliotidis Concha*] clear liver heat (Fan et al., 2024; Xu et al., 2024). While *Albizia julibrissin* Durazz [Fabaceae; *Albiziae Cortex*] brings calming effects and alleviates depression (Zhang et al., 2021). The above pharmacopoeial names are all referenced from the Chinese Pharmacopoeia 2020. The botanical names have been authenticated using the Plants of the World Online database (http://www.plantsoftheworldonline.org). KXN has been approved for the clinical treatment of PMS. However, its specific active metabolites and mechanisms of action still remain to be clarified.

In this research, a bilateral OVX rat model is established to validate the therapeutic effects of KXN on PMS. The study utilizes molecular networking construction, network analysis, and multi-omics analysis to find the key pathway affected by KXN in the treatment of PMS. It is suggested that the potential bioactive metabolites in KXN related to steroid hormone biosynthesis pathway could motivate CYP19A1 to improve estrogen deficiency in PMS. This research will contribute to a better understanding of how KXN works to improve PMS and will aid in the development of therapeutic agents for PMS treatment.

## 2 Materials and methods

### 2.1 Chemicals and reagents

KXN granules were provided by Tasly Pharmaceutical Group Co., Ltd. (Tianjin, China) (Batch number: 2023E06). Isoflurane was purchased from Rayward Biotechnology Co., Ltd. (Shenzhen, China). Neutral formalin tissue fixative was provided by Solaibao Technology Co., Ltd. (Beijing, China). Sodium penicillin, 17β-estradiol (E2) and 7-Methoxy-4-trifluoromethylcoumarin (MFC) were purchased from Yuanye Bio-Technology Co., Ltd. (Shanghai, China). Nicotinamide adenine dinucleotide phosphate tetrasodium salt (NADPH-4Na^+^) and dimethyl sulfoxide (DMSO) were purchased from Solaibao Technology Co., Ltd. (Beijing, China). ELISA kits (E2, FSH, LH, PG) were purchased from Cloud-clone Technology Co., Ltd. (Wuhan, China). Analytical reference Astragaloside IV and Icaritin were purchased from Yuanye Bio-Technology Co., Ltd. (Shanghai, China). Hyperoside was purchased from Nature-Standard Technical Service Co., Ltd. (Shanghai, China). -(−)-syringaresinol was purchased from Alfa Biotechnology Co., Ltd. (Chengdu, China). Baohuoside I was purchased from Meilune Biotechnology Co., Ltd. (Dalian, China). SiCYP19A1 was synthesized by Shanghai Quanyang Biotechnology Co., Ltd. (Shanghai, China).

### 2.2 Drugs

Kunxinning Granule (KXN) is an innovative traditional Chinese medicine (TCM) has been approved for PMS treatment by National Medical Products Administration (NMPA) of China in 2021 (State Medical Permitment No. Z20210006). The details of the botanical drug formula are provided in Supplementary Data Sheet S2. The materials, including Chinese medicine decoction pieces, excipients, and preparations, were supplied by Tasly Pharmaceutical Group Co., Ltd., the new drug holder and manufacturer. All decoction pieces and excipients comply with the Chinese Pharmacopoeia. The preparation process of KXN is as follows: (1) Extraction: Qualified medicinal materials are pre-processed and extracted to obtain an extract. (2) Concentration: The extract filtrate is vacuum-concentrated to the required specific gravity, forming a paste. (3) Granulation: The paste is granulated using fluidized spray technology after adding excipients. (4) Drying and Packaging: The granules are dried at ≤80°C and packaged in aluminum foil bags. All materials have been inspected and certified as qualified, accompanied by inspection reports (Supplementary Data Sheet S2). All materials have been inspected and certified, with inspection reports provided in Supplementary Data Sheet S2.

We hereby confirm that the collection and processing of plant materials for this study fully comply with the Nagoya Protocol, CITES, all associated treaties including phytosanitary regulations, as well as the laws and regulations of China and the requirements of the Chinese Pharmacopoeia.

### 2.3 Cell lines

NCI-H295R cells were purchased from Punosai Life Technology Co., LTD. (Wuhan, China) and cultured in Dulbecco’s Modified Eagle Medium/Nutrient Mixture F-12 (DMEM/F-12) supplemented with 10% fetal bovine serum (FBS), 0.5% Insulin-Transferrin-Selenium (ITS-G) (×100) and 1% solution of Penicillin/Streptomycin (P/S). The cells were grown at 37°C in a humidified atmosphere containing 5% CO_2_.

### 2.4 Animal experiments

Animal experiments were conducted in accordance with the National Institutes of Health Guide for the Care and Use of Laboratory Animals and approved by the Nankai University Institutional Animal Care and Use Committee (2023-SYDWLL-000583). Female SD rats (8–10 weeks old, 200 ± 20 g) were procured from Beijing Vital River Laboratory Animal Technology Co., Ltd. The rats were housed under standard specific pathogen-free conditions at a temperature of 23°C ± 2°C with a 12/12-h light/dark cycle and provided *ad libitum* access to water and food.

#### 2.4.1 OVX model

Female SD rats were randomly assigned to six groups: Control (Con), Model (Mod), 17β-Estradiol (E2) positive drug, and KXN groups, each with three different dosage levels. Except for the Con group, which underwent only minimal fat removal around the ovary, the remaining five groups underwent bilateral oophorectomy following previously reported methods (Tian et al., 2023). Post-surgery, vaginal lavage smears from the rats were regularly stained with Wright-Giemsa over five consecutive days. Levels of E2 and Follicle-stimulating hormone (FSH) in rat serum were measured using ELISA after blood collection from the retro-orbital venous plexus. Rats displaying estrous cycle disorders, accompanied by decreased E2 and increased FSH levels, were selected for subsequent experiments (Supplementary Figure S3).

#### 2.4.2 Animal administration and pharmacological effect tests

The sham operation group and OVX group received intragastric administration of vehicle, which contained 3% Tween-80 in normal saline. The other four groups were administered E2 (0.1 mg/kg/d) and KXN (3, 6, and 12 mg/kg/d) via gavage over 28 days. The open field test was utilized to assess anxiety and depression levels in rats from each group. Pain thresholds in the legs and paws of the rats were measured using a pressure application measuring instrument (Ugo Basile, 38,500, Italy). Subsequently, all rats were euthanized, and the right tibia, adrenal glands, uterus, and serum samples were collected for structural analysis and biochemical examination. Adrenal tissues were promptly stored at −80°C for subsequent 4D-DIA proteomics analysis (detailed in Supplementary Material S1). Serum was collected to detect E2, FSH, PG, and LH levels by ELISA.

### 2.5 Open-field test

The Open Field Test (OFT) was conducted following the protocol described by Gebara et al. (2021), with some minor adjustments. The test took place in a soundproof room with a neutral ambiance, from 8:30 a.m. to 2:30 p.m. The OFT apparatus consisted of a black wooden box measuring 50 cm in length, width, and height. Each animal was placed in the box individually, and their movements were recorded by cameras as they freely explored for 5 min. In between tests, the box was thoroughly cleaned with 90% ethanol and dried to remove any lingering odors from the previous occupant. The rats’ activity levels and the time spent in the central area were analyzed using the Animal Behavior Video Analysis System, version 2.0.

### 2.6 Micro-computed tomography

Micro-CT scans of rats’ tibias were performed using a PerkinElmer Quantum GX2 scanner with the following settings: 90 kV, 88 μA, and a Cu 0.06 + Al 0.5 filter over a 72 mm × 72 mm field-of-view. Each scan was completed in about 5 min, yielding images in VOX format. These images were then processed with Analyze software for 3D reconstruction and microstructural analysis.

### 2.7 Molecular network and chemical scaffold classification assay

KXN extracts were prepared by sonication in 50% methanol and centrifugation. Rats were administered KXN orally at 30 g/kg, and blood samples were collected at 30, 60, 120, and 240 min from the retroorbital vein. After centrifugation and methanol extraction, 200 μL of the supernatant from each time point was combined for UPLC/Q-TOF/MS analysis. The processed MS/MS data were uploaded to GNPS (https://gnps.ucsd.edu), where they were analyzed with strict parameters and then merged into a molecular network using the method described by Wang et al. (2019). Cytoscape software v3.8.1 (www.cytoscape.org) was employed to construct the molecular network. Moreover, Scaffold Hunter V2.6.3 software (www.scaffoldhunter.sourceforge.net) was utilized to visually elucidate the relationships between metabolites in plasma for chemical scaffold classification.

### 2.8 Network analysis

The compound structure information of representative metabolites in plasma was input into the PharmMapper database (http://www.lilab-ecust.cn/pharmmapper/) in SDF format to predict potential targets. The top 30 proteins for each compound, based on the fitting values, were selected for analysis of protein-protein interaction and KEGG pathway enrichment using the String database (https://cn.string-db.org/). The PMS-related KEGG pathways were retrieved from the GeneCards database (https://www.genecards.org/) combined String database.

### 2.9 CYP19A1 activity detection

CYP19A1 activity detection was performed based on the experimental method (Song et al., 2016). Protein solutions from H295R was quantified using a BCA kit. Lysates (5 mg/mL) and drugs were first incubated at 4°C for 18 h and then preheated in a water bath at 37°C for 10 min. Reactions were initiated using substrate solutions containing MFC and NADPH-4Na^**+**^ in PBS, and then halted with acetonitrile-dissolved Tris base (0.1 M). The fluorescent product, 7-Hydroxy-4-trifluoromethylcoumarin (HFC), was promptly measured using a microplate reader with excitation-emission wavelengths of 409–530 nm.

### 2.10 Statistical analysis

All experimental results are presented as mean ± standard deviation (SD). Each group in the cell experiments included three samples, each group in the animal experiments included six samples. Student’s t-test was used to analyze the significance between the two groups. One - way ANOVA corrected by Dunnett’s test was used to analyze the significance among multiple groups. Data analysis was performed using GraphPad Prism 9.0 (GraphPad Software, Inc., San Diego, CA, United States).

## 3 Results

### 3.1 KXN regulated hormonal disorders and improved abnormal uterine conditions

**FIGURE 1:**
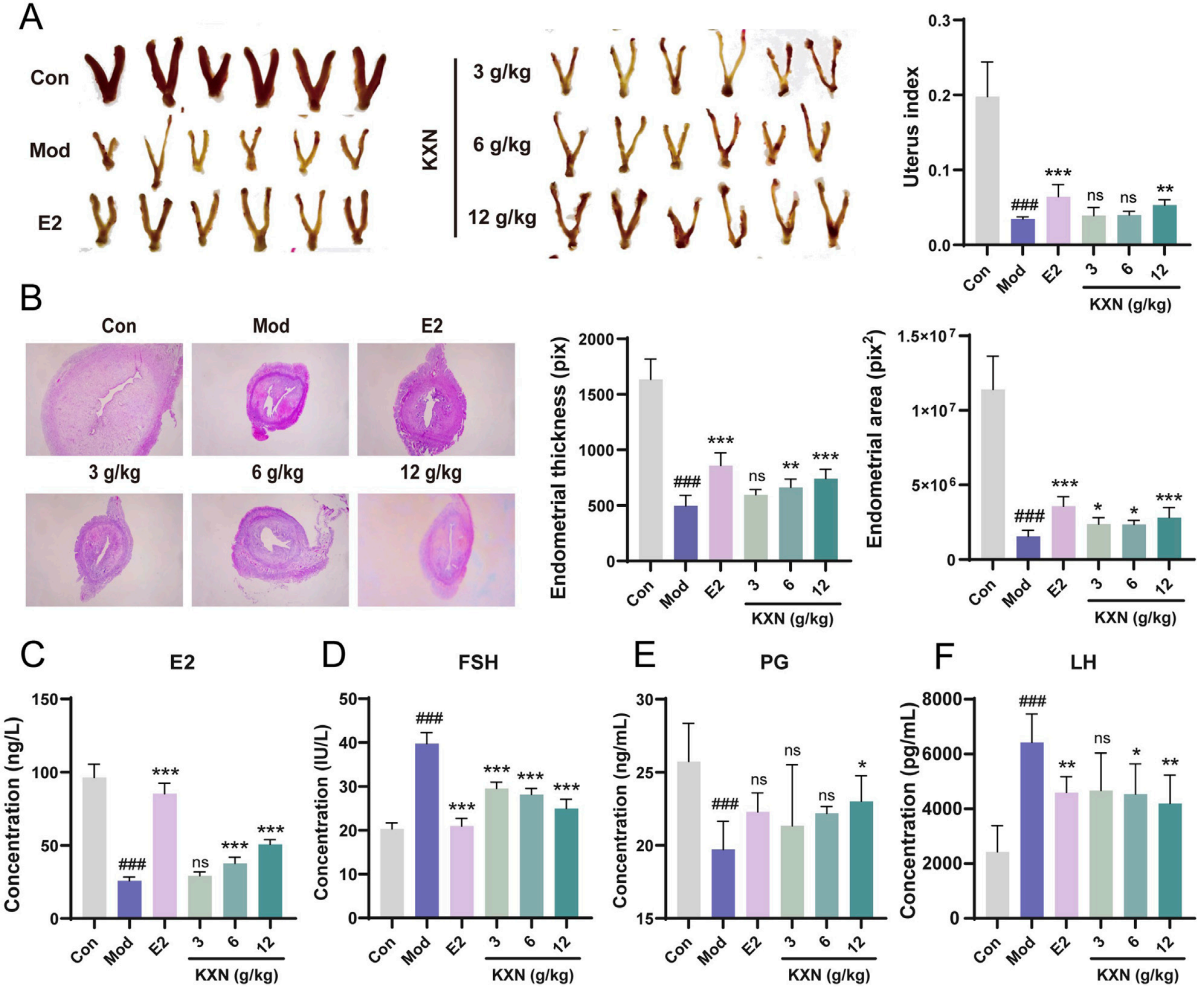
KXN relieved uterine atrophy and reversed hormone levels in ovariectomized (OVX) rats. **(A)** KXN restored uterine morphology and increased uterine index in OVX rats. **(B)** Representative HE staining images of the uterus cross-section of rats in each group and the average thickness and area of the endometrial. **(C–F)** KXN reversed serum E2, FSH, PG and LH levels in OVX rats. Bars represent the Mean ± SD (n = 6). ^###^ *p* < 0.001 compared with the Con group; *p < 0.05, **p < 0.01, ***p < 0.001 compared with the Mod group; ns indicates no significant difference compared with the Mod group.

In order to study the effects of KXN on perimenopausal syndrome in OVX rats, KXN was administered orally for 28 days, with 17β-estradiol as a positive control. Changes in uterine morphology and serum sex hormones were then observed. In Figure 1A, the uterus of rats in the Mod group (OVX rats without treatment group) exhibited significant atrophy compared to the blank group, along with a notable reduction in uterine index. However, after KXN treatment varying degrees of improvement were observed. Histological examination (HE staining) revealed reduced endometrial thickness and area, unclear uterine glands, incomplete endometrial shedding, and tightly arranged interstitial cells in OVX rats. These phenomena were alleviated to varying extents after KXN treatment (Figure 1B). The levels of serum sex hormones were then measured, and the results are presented in Figures 1C–F. Notably, KXN reversed the declining trend of serum E2 and PG levels in OVX rats. At the same time, it reduced the high levels of FSH and LH, indicating that KXN helped improve the imbalance of sex hormone related to PMS.

### 3.2 Chemical metabolites *in vitro* and absorbable metabolites *in vivo* of KXN

**FIGURE 2:**
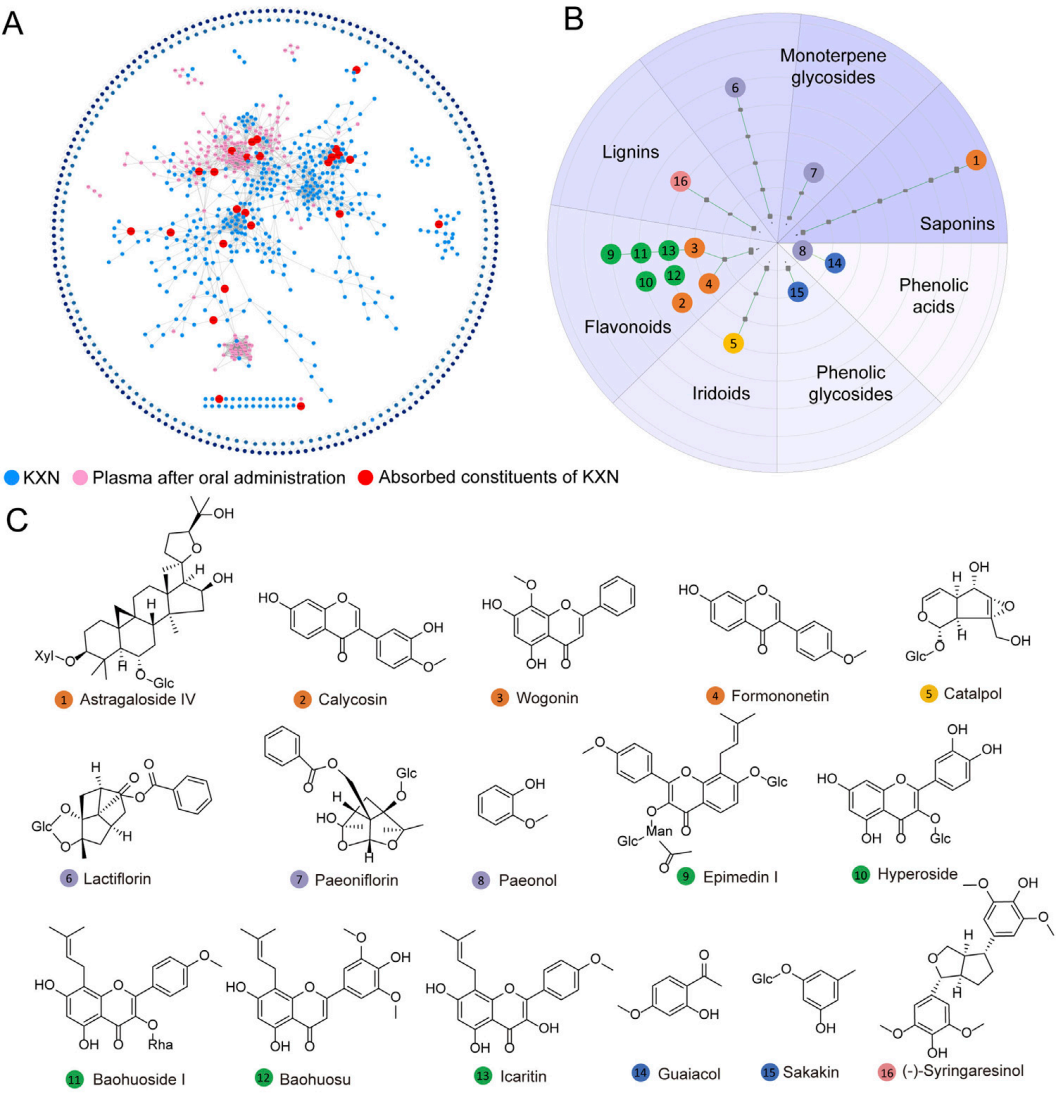
Identification and classification of absorbable metabolites in KXN. **(A)** Establishment of a molecular network for the absorbable metabolites of KXN utilizing the GNPS platform. The blue nodes denote the chemical constituents of KXN, the pink nodes indicate the plasma constituents in rats’ post-administration, and the red nodes signify the absorbable metabolites of KXN present in the plasma. **(B)** The classification of absorbable metabolites carried out by Scaffold Hunter. **(C)** The key chemical structures of potential active pharmacodynamic metabolites (APMs) in KXN.

To identify the absorbable metabolites of KXN, we analyzed an integrated Molecular network (MN) data of KXN extracts, blank plasma and drug-loaded plasma after oral administration of 30 mg/kg KXN on the GNPS platform using UPLC/Q-TOF-MS data (Supplementary Figures S1, S2). As depicted in Figure 2A, the MN map contains 1,093 precursor ions, comprising 307 clusters (node ≥2) and 437 single nodes. Based on accurate mass measurements and fragmentation patterns, a total of 115 chemical metabolites of KXN (Supplementary Table S1) and 16 representative absorbable metabolites of KXN extracts (Supplementary Table S2) were identified. These metabolites can be classified as eight flavonoids, two phenolic acids, one phenolic glycoside, one lignan, one saponin, one iridoid, and two monoterpene glycosides, as revealed in the Scaffold tree format shown in Figure 2B. The chemical structures of the 16 metabolites, depicted in Figure 2C, are considered the potential active pharmacodynamic metabolites (APMs) in KXN.

### 3.3 Network analysis of KXN

**FIGURE 3:**
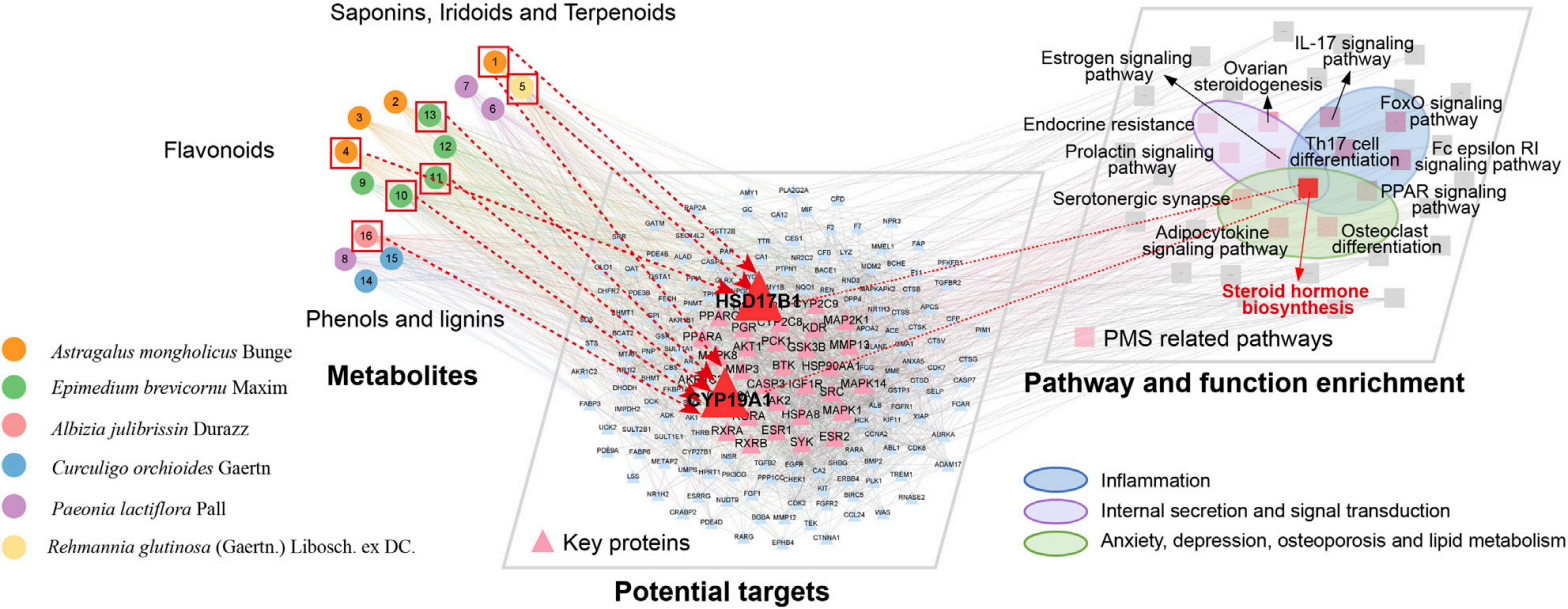
Multi-dimensional network analysis of “metabolite-target-pathway” improvement of PMS by KXN.

To elucidate the potential mechanisms underlying KXN’s pharmacological effects, we employed computational network analysis to predict the molecular targets of its metabolic metabolites, followed by pathway enrichment analysis. A comprehensive landscape network was constructed shown in Figure 3, including APMs, targets, pathways, and functions. The initial tier consisted of 16 KXN APMs, categorized into three distinct groups including “Phenols and Lignins,” “Flavonoids,” “Saponins, Iridoids and Terpenoids” based on their structural characteristics. Next, the target proteins of APMs were predicted using PharmMapper, and the protein-protein interaction (PPI) network was analyzed by STRING. Lastly, there was an enrichment network of pivotal pathways and functions. Thirteen pathways associated with PMS were identified, and three functions related to inflammation, internal secretion, anxiety, depression, osteoporosis and lipid metabolism (ADOL) were discovered. Steroid hormone biosynthesis, which involves inflammation, internal secretion, and the ADOL complex, probably played a critical role in alleviating PMS symptoms by KXN. Specifically, astragaloside IV, hyperoside, icaritin, (−)-syringaresinol and baohuoside I were predicted to influence CYP19A1, thereby contributing to PMS symptom relief. Additionally, astragaloside IV, formononetin and catalpol were identified as potential regulators of steroid hormone biosynthesis through their effects on HSD17B1, further supporting their therapeutic relevance in PMS.

### 3.4 Steroid hormone biosynthesis pathway is the key signaling pathway for KXN in improving PMS

**FIGURE 4:**
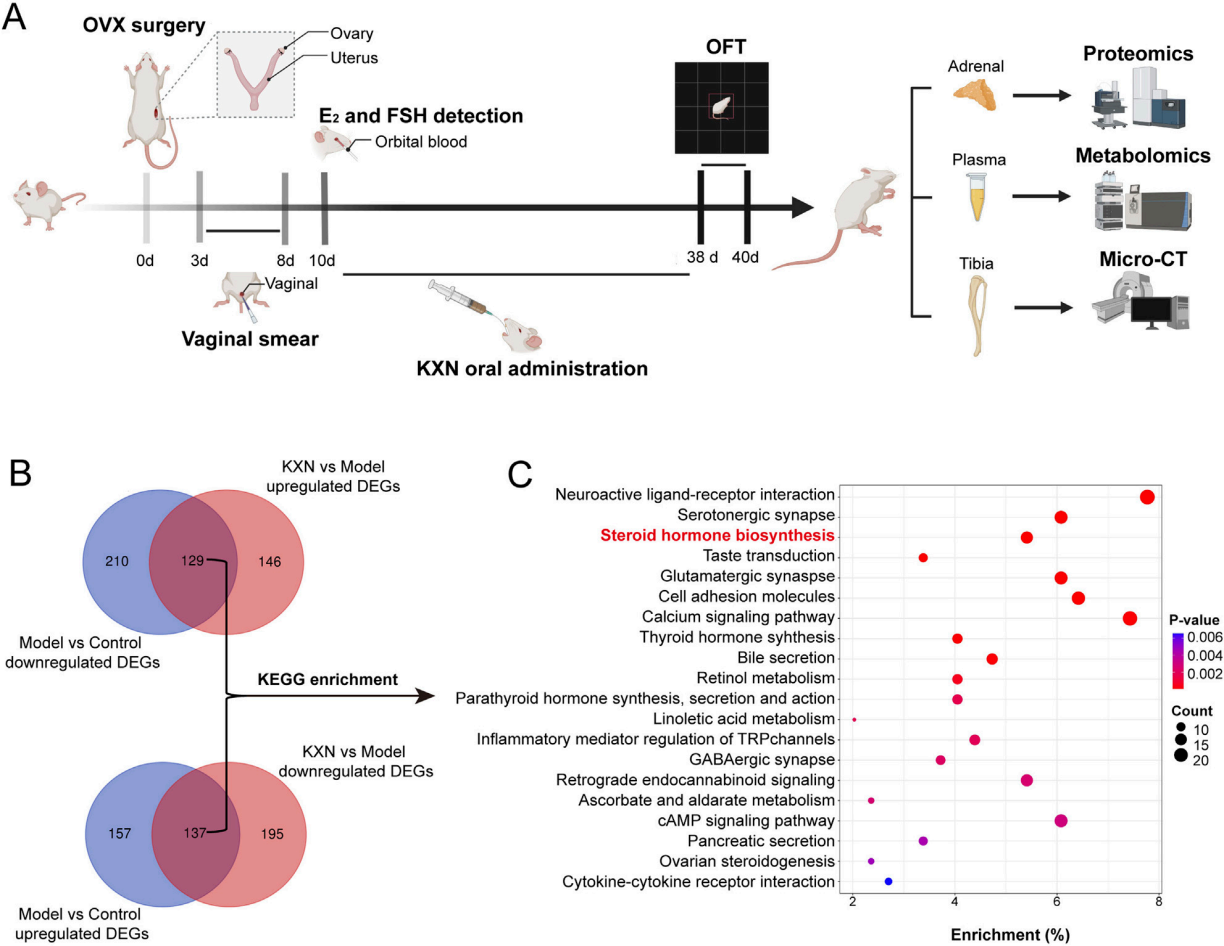
Proteomic analysis of rat adrenal glands revealed that Steroid hormone biosynthesis is the key pathway for KXN to alleviate PMS. **(A)** Schematic diagram of the experimental procedure to investigate the potential signaling pathway of KXN on OVX rats. **(B)** 4D-DIA quantitation proteomics of rat adrenal and revealed the different proteins affected by KXN. **(C)** Steroid hormone biosynthesis pathway was predicted as the pivotal signaling pathway for KXN’s amelioration on PMS. The false discovery rate (FDR) of search results was adjusted to <1% to quantification analysis. And the fold change (FC)≥ 1.5 or FC ≤ 0.6667, and *p*-value ≤ 0.05 were defined as significantly different proteins.

To understand how KXN enhances PMS at the molecular level, a 4D-DIA quantitation proteomics of rat adrenal gland was employed (Figure 4A). As a result, 11,730 proteins were identified with a false discovery rate of ≤0.01. OPLS-DA plot indicated substantial variations in the overall proteomic profiles among the Con, Mod, and KXN groups (Supplementary Figure S4). Compared to the Con group, 294 proteins were upregulated and 339 proteins were downregulated in the model group. However, after treatment with KXN granules, 275 proteins were upregulated and 332 proteins were downregulated proteins compared to the model group (*p* < 0.05) (Figure 4B; Supplementary Figures S5, S6). Subsequent data analysis revealed that a total of 266 proteins, including the intersection of 129 upregulated and 137 downregulated proteins demonstrated altered expression following intervention with KXN granules. The KEGG enrichment analysis of these findings highlighted the identification of the steroid hormone biosynthesis pathway as the pivotal signaling pathway for KXN’s improvement of PMS due to its smaller p-value and highest relevance to PMS (Figure 4C). Compared to the OVX model group, KXN treatment reversed the expression of steroid hormone biosynthesis-related proteins, including HSD3B, CYP21A2, StAR, and HSD11B2 (Supplementary Figure S7).

### 3.5 The key pharmacological metabolites in KXN activate CYP19A1 to alleviate estrogen dysregulation

**FIGURE 5:**
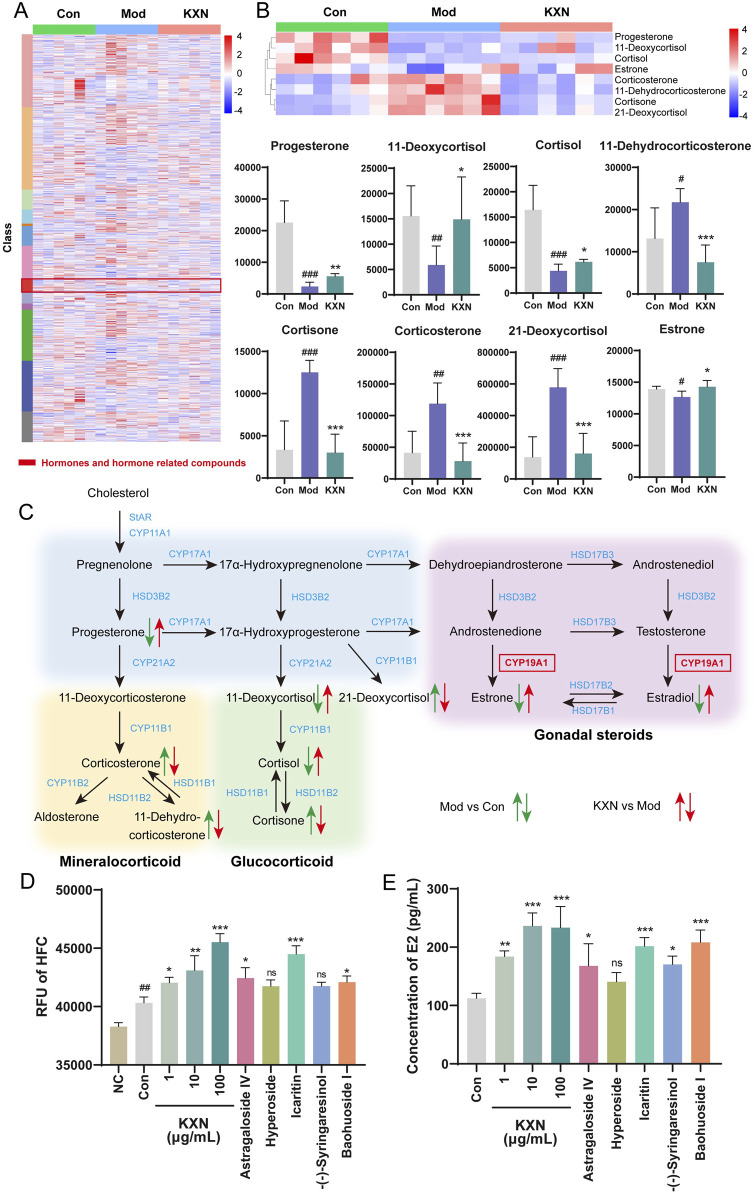
Metabolomics revealed hormones changes in steroid hormone biosynthesis after KXN treatment in OVX rats. **(A)** Heatmap showing the cluster analysis of total metabolites following KXN treatment. Low to high expression is represented by a change of color from blue to red, respectively. **(B)** Differential metabolites enriched in the steroid hormone biosynthesis pathway. Low to high expression is represented by a change of color from blue to red, respectively. Bars represent the Mean ± SD (n = 6). ^#^ *p* < 0.05, ^##^ *p* < 0.01, ^###^ *p* < 0.001 compared with the Con group; *p < 0.05, **p < 0.01, ***p < 0 0.001 compared with the Mod group. **(C)** Schematic representation of the comprehensive regulatory framework governing the steroid hormone biosynthesis pathway and its metabolites influenced by KXN. Different colors signify alterations in metabolite levels between groups, with upward arrows denoting increased metabolite concentrations and downward arrows indicating decreased metabolite concentrations. **(D)** KXN and astragaloside IV, icaritin, and baohuoside I increased the activity of CYP19A1. Bars represent the Mean ± SD (n = 3). ^##^ *p* < 0.01 compared with the Negative Control (NC) group; *p < 0.05, **p < 0.01, ***p < 0 0.001 compared with the Con group; ns indicates no significant difference compared with the Con group **(E)** KXN and astragaloside IV, icaritin, and baohuoside I upregulated the level of E2 secretion in H295R cells. Bars represent the Mean ± SD (n = 3). *p < 0.05, **p < 0.01, ***p < 0 0.001 compared with the Con group; ns indicates no significant difference compared with the Con group.

A serum metabolomics study was conducted to examine the metabolic regulatory mechanism further following KXN treatment (Supplementary Figures S8, S9). The heat map revealed the changes of total differential metabolites after KXN intervention, with red modules representing changes in a class of hormones and hormone-related metabolites (Figure 5A). Levels of progesterone, 11-deoxycortisol, cortisol, and estrone in the Mod group were decreased compared to the Con group. In contrast, levels of corticosterone, 11-dehydrocorticosterone, cortisone, and 21-deoxycortisol were increased in the Mod group; however, these levels significantly decreased following KXN treatment (Figure 5B). Based on these findings, the steroid hormone biosynthesis pathway impacted by KXN in OVX rats was summarized (Figure 5C). Cytochrome P450 Family 19 Subfamily A Member 1 (CYP19A1) catalyzes the conversion of C19 androgens, androst-4-ene-3,17-dione (androstenedione) and testosterone to the C18 estrogens, estrone and estradiol respectively. The expression levels of CYP19A1 in rat adrenal, uterus, hypothalamus tissues were detected by ELISA. The result showed that KXN improve the expression of CYP19A1 in rat adrenal, uterus, hypothalamus tissues (Supplementary Figure S10). The enzymatic activity of CYP19A1 was also detected after KXN intervention in H295R cell lysates. The results demonstrated that KXN granules enhanced the enzyme activity of CYP19A1 (Figure 5D). Network analysis target prediction suggested that astragaloside IV, hyperoside, icaritin, -(−)-syringaresinol, and baohuoside I may affect CYP19A1. Corresponding enzyme activity assays showed that astragaloside IV, icaritin, and baohuoside I indeed increased the activity of CYP19A1. Treatment with KXN also led to an increase in estradiol level, as well as the presence of these three metabolites in H295R cells (Figure 5E). To further confirm the essential role of CYP19A1 in this process, we performed siRNA-mediated knockdown of CYP19A1 in H295R cells. Notably, after CYP19A1 knockout, the promoting effects of KXN and its three active metabolites (astragaloside IV, icaritin, and baohuoside I) on estradiol secretion were significantly inhibited (Supplementary Figure S11). These results indicated that the key pharmacological metabolites of KXN, namely, astragaloside IV, icaritin, and baohuoside I, could enhance the steroid hormone biosynthesis pathway by activating CYP19A1, thus potentially addressing estrogen deficiency in PMS.

### 3.6 KXN relieves anxiety, depression and osteoporosis

**FIGURE 6:**
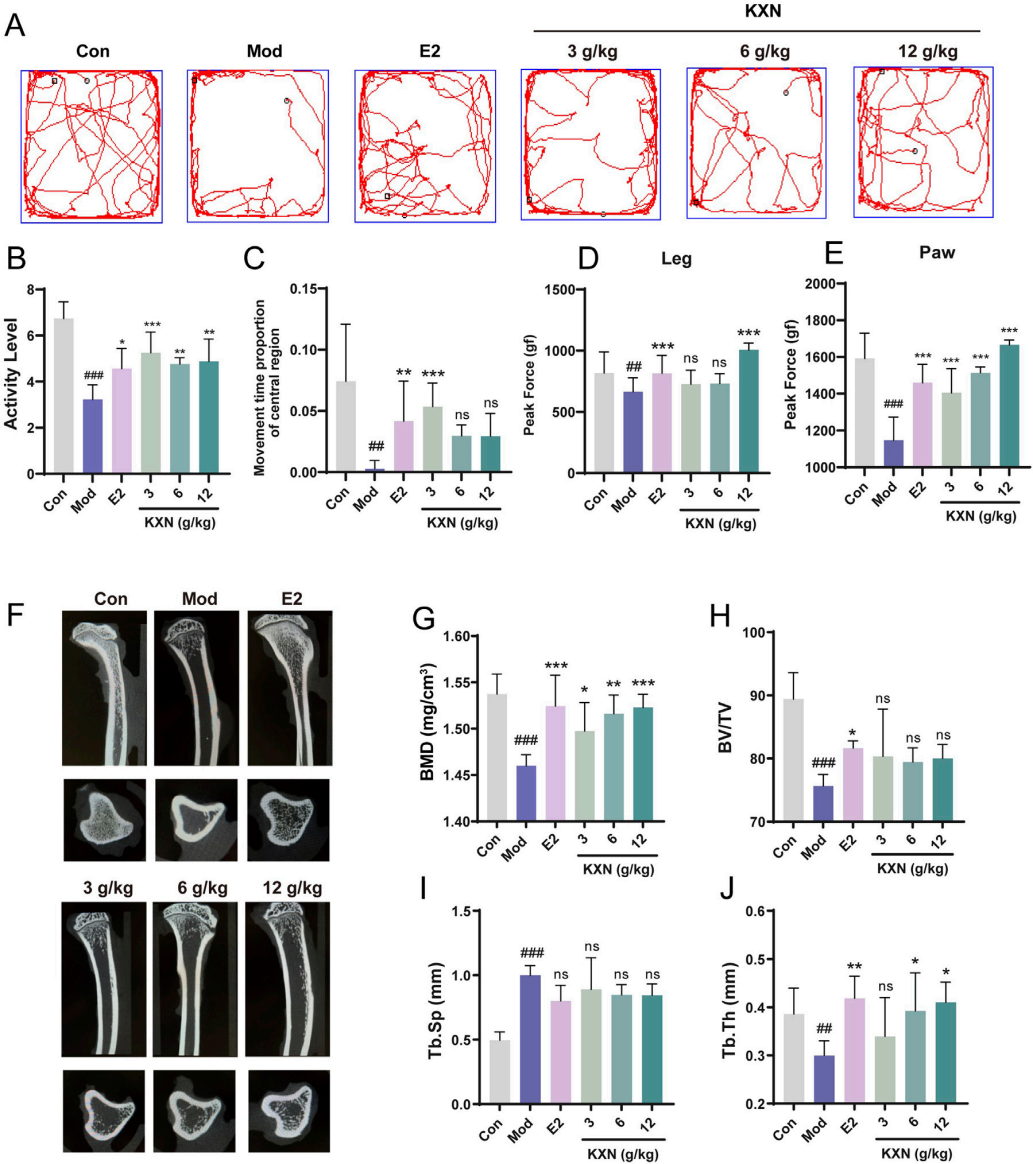
KXN relieved anxiety, depression and osteoporosis in OVX rats. **(A)** The movement track of each group of rats in the open field test. **(B, C)** KXN increased activity and central area residence time in OVX rats. Bars represent the Mean ± SD (n = 6). ^##^ *p* < 0.01, ^###^ *p* < 0.001 compared with the Con group; *p < 0.05, **p < 0.01, ***p < 0 0.001 compared with the Mod group; ns indicates no significant difference compared with the Mod group. **(D, E)** Pain thresholds of left posterior thigh and left posterior paw were improved in OVX rats. Bars represent the Mean ± SD (n = 6). ^##^ *p* < 0.01, ^###^ *p* < 0.001 compared with the Con group; ***p < 0 0.001 compared with the Mod group, ns indicates no significant difference compared with the Mod group. **(F)** Representative micro-CT 3D reconstruction images of bone microstructure in different groups. The above image is the sagittal plane image of the tibia, and the lower is the image in the view of the cross section. **(G–J)** KXN downturned the rats’ bone mineral density (BMD), bone volume/tissue volume fraction (BV/TV), trabecular spacing (Tb. Sp) and the trabecular thickness (Tb. Th) after OVX. Bars represent the Mean ± SD (n = 6). ^##^ *p* < 0.01, ^###^ *p* < 0.001 compared with the Con group; *p < 0.05, **p < 0.01, ***p < 0 0.001 compared with the Mod group, ns indicates no significant difference compared with the Mod group.

Anxiety, depression and osteoporosis are complications of perimenopausal syndrome associated with estrogen deficiency. To investigate whether KXN’s regulation of hormones could ameliorate perimenopausal symptoms, an open-field test was designed to evaluate the therapeutic effect of KXN on mood. OFT illustrated that the locomotor activity and residence time in the central area of OVX rats were significantly reduced compared to the control group (Figures 6A–C). Conversely, these parameters were markedly elevated in the KXN group, suggesting a notable alleviation of neurological symptoms by KXN. Previous studies have reported that declining estrogen levels in menopausal women may lead to pain sensitization (Li et al., 2014). In this study, we utilized the pressure application measuring instrument (Ugo Basile, 38,500, Italy) to assess the pain threshold of both hind legs and paws of OVX rats. The results indicated that the response threshold to mechanical stimulation in the hind legs and paws of OVX rats decreased but returned to normal levels in KXN-treated rats (Figures 6D, E). The micro-CT analysis of tibial samples revealed a significant reduction and disruption in bone trabeculae in the model group. In contrast, Con and KXN groups displayed closely arranged mesh-like structures in the bone trabeculae (Figure 6F). Further analysis demonstrated that the KXN group effectively increased the bone mineral density (BMD) and trabecular thickness (Tb.Th) compared to the OVX group. While the bone volume/tissue volume fraction (BV/TV) and trabecular bone spacing (Tb. Sp) were not improved in the KXN group (Figures 6G–J). These findings provided evidence supporting the therapeutic effectiveness of KXN in treatment PMS by improving hormonal metabolic disorders.

## 4 Discussion

Endocrine disruptions resulting from decreased ovarian function and reductions in estrogen levels, specifically estradiol and estrone, are the primary causes of PMS. This study found that KXN can improve the hormone metabolism disorder in OVX rats by regulating the biosynthesis of steroid hormones. Multidimensional analysis of the “metabolite-target-pathway” revealed that the hormone biosynthesis pathway, where HSD17B1 and CYP19A1 are located, represents a promising signaling mechanism for the therapeutic effects of KXN on PMS. Proteomic studies further confirmed that KXN alleviates hormonal imbalances by targeting adrenal steroid hormone synthesis, thereby improving perimenopausal anxiety, depression, and osteoporosis in PMS.

Impaired steroid hormone biosynthesis can change aldosterone, cortisol, and E2 levels. These substances play important roles in body development, immune regulation, regulation of sexual function and fertility control. The steroid hormone biosynthesis process begins with cholesterol absorption as a raw material into the mitochondria. CYP11A1 catalyzes the conversion of cholesterol to pregnenolone. Pregnenolone is then converted to progesterone by a bifocal enzyme complex (HSD3B2) (Huang et al., 2022). Pregnenolone and progesterone serve as the precursors for glucocorticoids, mineral corticoids, and gonadal hormones. Early investigations have shown that a decrease in progesterone is one of the earliest changes during the menopausal transition (Motlani et al., 2023). Supplementation with progesterone can help alleviate menopausal symptoms and improve sleep without impacting cognition, blood lipids or cardiovascular risk factors (Spark and Willis, 2012). Our findings also revealed a reversal in progesterone levels following KXN treatment, as opposed to the model group, where a decrease in progesterone levels was observed.

Pregnenolone and progesterone are catalyzed by enzymes CYP17A1 and HSD3B2 to produce androgens such as testosterone and androstenedione. These androgens are further converted into estrogen by aromatization, which is facilitated by the enzyme CYP19A1. Before menopause, the ovaries are the primary source of estrogen hormones E1 and E2. During this time, endometrial cells within the follicle, under the action of luteinizing hormone (LH), produce androstenedione and testosterone. Aromatase (CYP191) catalyzes the conversion of androstenedione to E1 and testosterone to E2 (Giacomini et al., 2024). After menopause, the estrogen source in a woman’s body changes due to decreased ovarian function. More than 75% of the total proandrogens that synthesize E2 are produced by the adrenal glands. As a result, the steroid hormone synthesis pathway of the adrenal glands gradually becomes the main source of estrogen in the blood circulation after menopause (Chakraborty et al., 2021). Corticosterone is produced from progesterone catalyzed sequentially by CYP21A2 and CYP11B1/2. After OVX, corticosterone levels rise, leading to increased visceral fat accumulation and causing visceral obesity in women (Xu et al., 2023). In this study, KXN administration reduced the corticosterone levels and enhanced the activity of CYP19A1, promoting adrenal steroid hormone synthesis in OVX rats. Furthermore, mineralocorticoids and glucocorticoids like 11-deoxycortisol, 11-dehydrocorticosterone, and 21-deoxycortisol were found to be dysregulated in PMS, potentially contributing to disease progression as causative factors.

Perimenopausal women have an increased risk of developing mood disorders and depression due to changes in enzyme activity and receptor distribution influenced by estrogen within the neurotransmitter system (Freeman et al., 2014; Albert and Newhouse, 2019). Estrogen deficiency reduces the availability of 5-HT, affects the status of 5-HT receptors, and regulates norepinephrine levels and its receptor distribution (Gu et al., 2022). Additionally, cholinergic activity decreases in various brain regions, leading to impaired performance on learning and memory tasks in OVX rats (Gedankien et al., 2023). Our study presents evidence that KXN effectively mitigates anxiety and depression in ovariectomized rats by modulating estrogen levels.

KXN’s effect on estrogen levels also benefits bone health. Osteoporosis is a series of reactions to bone homeostasis dysregulation mediated by estrogen deficiency in the postmenopausal women (Li et al., 2024). Estrogen deficiency leads to the disorder of RANK-RANKL-OPG pathway, activation of osteoclasts and promotion of bone loss (Li and Wang, 2018). In addition, immune imbalances due to estrogen withdrawal contribute to bone degradation (Sapir-Koren and Livshits, 2017; Tsukasaki and Takayanagi, 2019). Estrogen decline can lead to further imbalance in bone health by impacting the production of osteocalcin and vitamin D (Li et al., 2020). Research has shown that treatment with KXN can improve bone mineral density in ovariectomized rats. Additionally, it enhances the microstructure of trabecular bone, suggesting its potential as a therapeutic agent for osteoporosis.

KXN’s multifaceted action in regulating estrogen levels indicates that it could provide comprehensive support for the complex physiological changes that occur during the perimenopausal period. Certainly, several limitations are present in this research. The network analysis calculation method carries the risk of generating false positives. Our study provides insights into the modern biological mechanisms of KXN in improving PMS based on animal experiments. However, further clinical studies are still needed to validate these mechanistic findings in human subjects. Moreover, the specific mechanisms underlying the absorbable active metabolites in KXN remain to be further elucidated.

## 5 Conclusion

**FIGURE 7:**
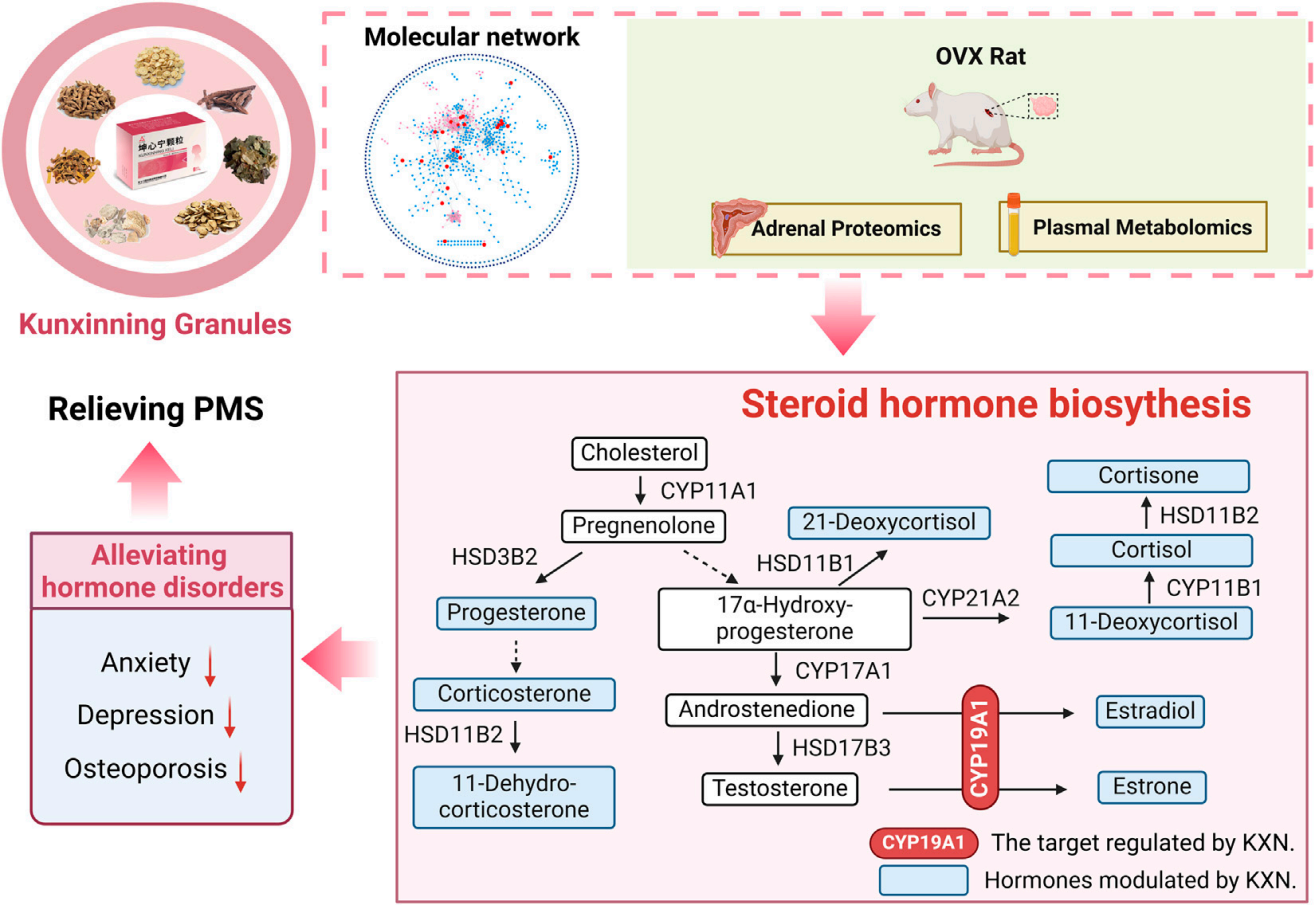
Mechanism diagram of KXN supply estrogen deficiency in PMS.

This study elucidates the pharmacological mechanisms of KXN in alleviating PMS by modulating the steroid hormone biosynthesis pathway (Figure 7). Through enhancing CYP19A1 activity, key bioactive metabolites including astragaloside IV, icariin, and baohuoside I promote estradiol synthesis, effectively addressing estrogen deficiency. These findings provide mechanistic insights into KXN’s therapeutic potential and support its clinical application in PMS treatment.

## Data Availability

The original contributions presented in the study are included in the article/Supplementary Material, further inquiries can be directed to the corresponding authors.

## Abbreviations

KXN, Kunxinning Granules; PMS, Perimenopausal Syndrome; UPLC/Q-TOF-MS, Ultra-high performance liquid chromatography with quadrupole time-of-flight mass spectrometry; OVX, ovariectomy; E2, Estradiol; E1, Estrone; FSH, Follicle-stimulating hormone; PG, Progesterone; LH, Luteinizing hormone; OFT, Open-filed test; Micro-CT, Micro-Computed Tomography; MFC, 7-Methoxy-4-trifluoromethylcoumarin; HFC, 7-Hydroxy-4-trifluoromethylcoumarin; DMSO, Dimethyl sulfoxide; NADPH-4Na+, Nicotinamide adenine dinucleotide phosphate tetrasodium salt; MN, Molecular network; APMs, active pharmacodynamic metabolites; PPI, protein-protein interaction; ADOL, anxiety, depression, osteoporosis and lipid metabolism; CYP19A1, Cytochrome P450 Family 19 Subfamily A Member 1; NC, Negative Control; BMD, bone mineral density; BV/TV, bone volume/tissue volume fraction; Tb. Sp, trabecular spacing; Tb. Th, trabecular thickness; ELISA, Enzyme-Linked Immunosorbent Assay.
