# Supplementary material for: Kunxinning granules alleviate perimenopausal syndrome by supplementing estrogen deficiency
Source: Front Pharmacol. 2025 Mar 26;16:1554479. doi: 10.3389/fphar.2025.1554479 (PMC11979375; doi:10.3389/fphar.2025.1554479)
Supplement: Supplementary file 1 [file DataSheet2.pdf]

1. The prescription for Kunxinning granules

Table 1. Prescription for Kunxinning granules

Chinese Name	Botanical name	Part used	Granule dosage
地黄 (Di-Huang)	*Rehmannia glutinosa* (Gaertn.) Libosch. ex DC.	Dried Root	444 g
黄芪 (Huang-Qi)	*Astragalus mongholicus Bunge*	Dried Root	666 g
仙茅 (Xian-Mao)	*Curculigo orchioides* Gaertn	Dried roots and stems	444 g
淫羊藿 (Yin-Yang-Huo)	*Epimedium brevicornu* Maxim	Dried Stems and Leaves	444 g
赤芍 (Chi-Shao)	*Paeonia lactiflora* Pall	Dried Root	444 g
合欢皮 (He-Huan-Pi)	*Albizia julibrissin* Durazz	Dried Bark	333 g
石决明 (Shi-Jue-Ming)	Haliotis	Shell	555 g

地黄 (Di-Huang) - *Rehmannia glutinosa* (Gaertn.) Libosch. ex DC. [Scrophulariaceae; *Rehmanniae Radix Praeparata*]
https://powo.science.kew.org/taxon/urn:lsid:ipni.org:names:808644-1

黄芪 (Huang-Qi) - *Astragalus mongholicus* Bunge [Fabaceae; *Astragali radix*]

https://powo.science.kew.org/taxon/urn:lsid:ipni.org:names:478720-1

仙茅 (Xian-Mao) - Curculigo orchioides Gaertn [Amaryllidaceae; *Curculiginis Rhizoma*]
https://powo.science.kew.org/taxon/urn:lsid:ipni.org:names:64163-1

淫羊藿 (Yin-Yang-Huo) - *Epimedium brevicornu* Maxim [Berberidaceae; *Epimedii Folium*]
https://powo.science.kew.org/taxon/urn:lsid:ipni.org:names:107262-1

赤芍 (Chi-Shao) - *Paeonia lactiflora* Pall [Paeoniaceae; *Paeoniae Radix Rubra*]

https://powo.science.kew.org/taxon/urn:lsid:ipni.org:names:711734-1

合欢皮 (He-Huan-Pi) - *Albizia julibrissin* Durazz [Fabaceae; *Albiziae Cortex*]

https://powo.science.kew.org/taxon/urn:lsid:ipni.org:names:473275-1

石决明 (Shi-Jue-Ming) - Haliotis [Haliotidae; *Haliotidis Concha*]

It's both an animal drug and a mineral drug in traditional Chinese medicine. It is obtained from derived from the shells of Haliotis diversicolor Reeve, Haliotis discus hannai Ino, Haliotis ovina Gmelin, Haliotis ruber (Leach), Haliotis asinina Linnaeus or Haliotis laevigata (Donovan).

The pharmacopoeial names are all referenced from the Chinese Pharmacopoeia 2020. The botanical names have been verified with Plants of the Worlds Online (http://www.plantsoftheworldonline.org).

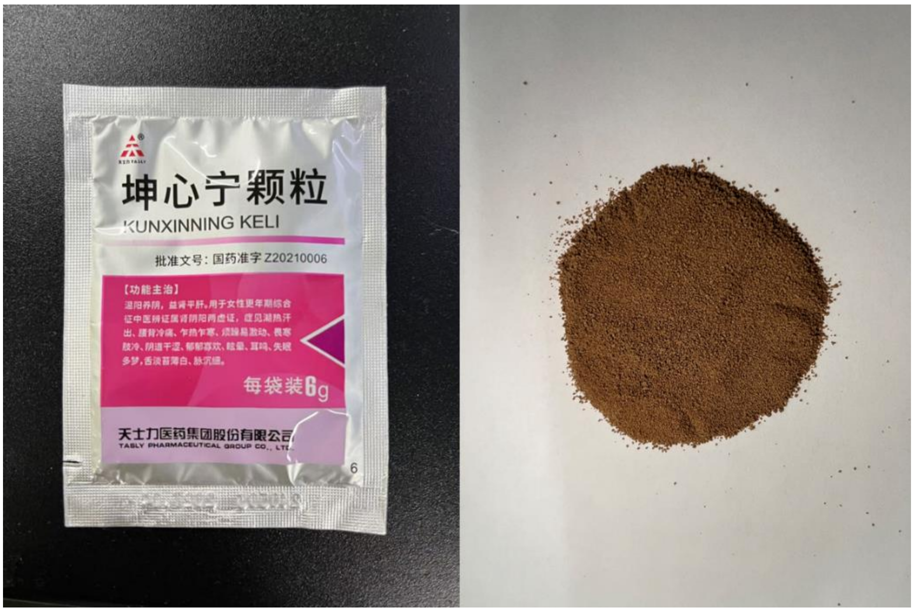

Figure 1. Image of KXN

2. The analytical certificate provided by manufacturer

The materials used in this study were all listed commodity materials (including Chinese medicine decoction pieces, excipients and preparations) provided by Tasly Pharmaceutical Group Co., LTD., the new drug holder and manufacturer. Among them, all the Chinese medicine decoction pieces and excipients comply with Chinese Pharmacopoeia. Production process and preparation standards Strictly implement the production process and quality standards of listed products approved by NMPA. All materials have been inspected and certified as qualified, accompanied by inspection reports (Figure 2). As KXN is a listed product, the specific quality standards are not readily disclosed due to regulatory and competitive considerations.

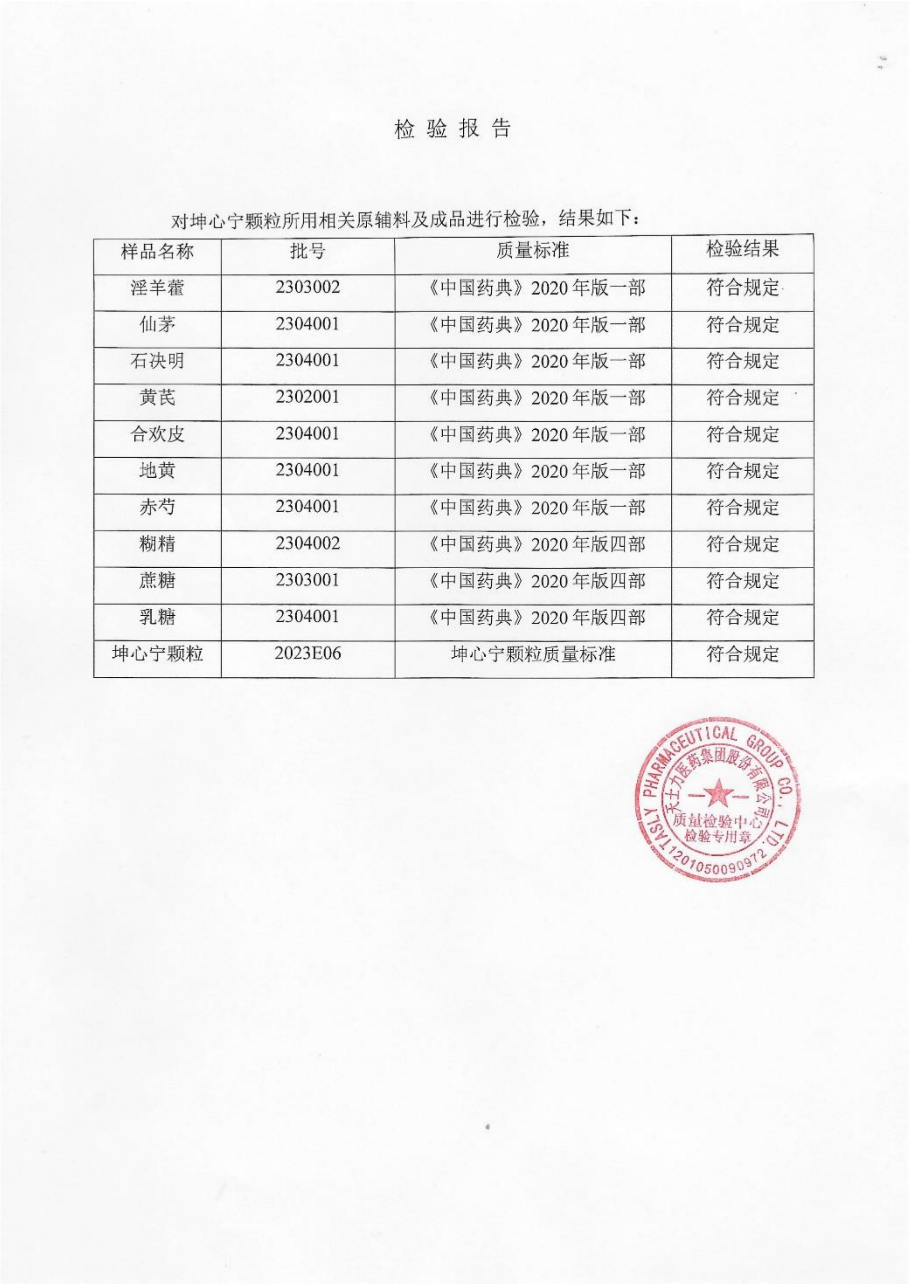

检 验 报 告

对坤心宁颗粒所用相关原辅料及成品进行检验，结果如下：

样品名称	批号	质量标准	检验结果
淫羊藿	2303002	《中国药典》2020年版一部	符合规定
仙茅	2304001	《中国药典》2020年版一部	符合规定
石决明	2304001	《中国药典》2020年版一部	符合规定
黄芪	2302001	《中国药典》2020年版一部	符合规定
合欢皮	2304001	《中国药典》2020年版一部	符合规定
地黄	2304001	《中国药典》2020年版一部	符合规定
赤芍	2304001	《中国药典》2020年版一部	符合规定
糊精	2304002	《中国药典》2020年版四部	符合规定
蔗糖	2303001	《中国药典》2020年版四部	符合规定
乳糖	2304001	《中国药典》2020年版四部	符合规定
坤心宁颗粒	2023E06	坤心宁颗粒质量标准	符合规定

Figure 2. The analytical certificate provided by manufacturer.

3. The analytical methods and results relevant for KXN extracts

3.1 Preparation of KXN extracts:

Take 2 bags of Kunxinning Granules (KXN) and grind them into a uniform powder. Take an appropriate amount of powder and dissolve it with 50% methanol to make 20 mg/mL KXN sample solution.

3.2 HPLC analysis of KXN extracts:

Instrument: Prominence LC-20AT (SHIMADZU, Japan) The chromatography was performed on Phenomenex® NX-C18 110A column (4.6 mm×250 mm, 5 μm) at 28℃ with mobile phase A consisting of water and mobile phase B consisting of acetonitrile by gradient elution (0~20 min，3%~10%B；20~25 min，10%~20%B；25~45 min，

20%~40%B；45~55 min，40%~50%B；55~65 min，50%~70%B；65~70 min，70%~85%B；70~72 min，85%~100%B), the flow rate was 1 mL·min^{-1}, and the sample size was 10 μL. The detection wavelength was 210 nm.

3.2.1 HPLC fingerprint of Kunxinning granules with three different detection wavelengths.

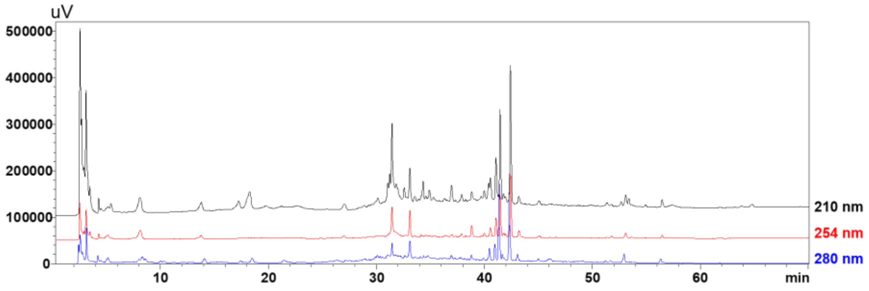

Figure 2. HPLC fingerprint of Kunxinning granules with three different detection wavelengths

3.2.2 Overlay of the chromatogram of the sample with that of officially specified reference standards:

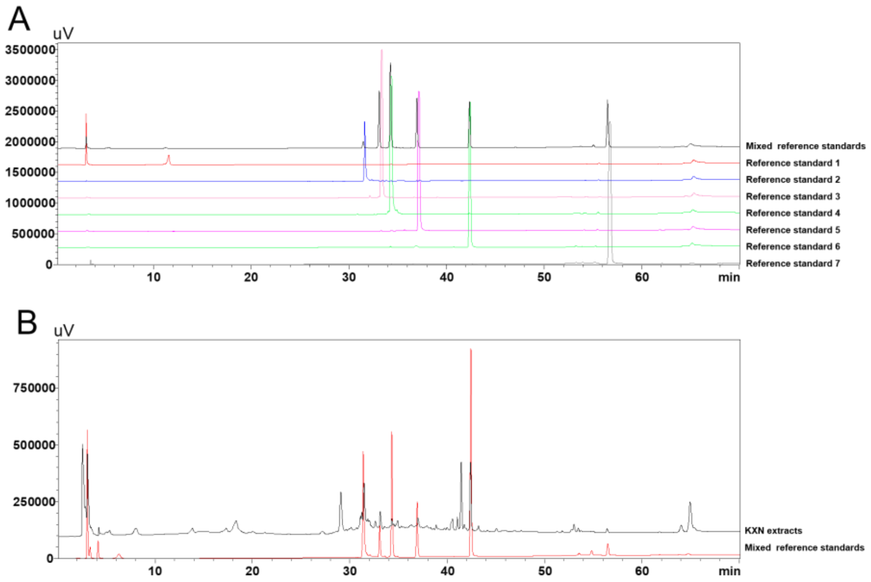

Figure 3. The chromatogram of KXN extracts with reference standards

Note: Reference standard 1~7 are Rhmannioside D, Paeoniflorin, Calycosin glucoside, (-)-Syringaresnol-4-O-beta-D-apiofuranosyl-(1→2)-beta-D-glucopyranoside, Curculigoside, Icariin and Baohuoside I spectral peaks.

3.2.3 Quantification of marker compounds:

According to the Chinese Pharmacopoeia, quantitative analysis was carried out for Rhmannioside D in Rehmanniae Radix Praeparata, paeoniflorin in Paeoniae Radix Rubra, calycosin-7-O-β-D-glucoside in Astragali radix, (-)-Syringaresnol-4-O-beta-D-apiofuranosyl-(1→2)-beta-D-glucopyranoside in Albiziae Cortex, Curculigoside in

Curculiginis Rhizoma, icariin and Baohuoside I in Epimedium brevicornu Maxim.

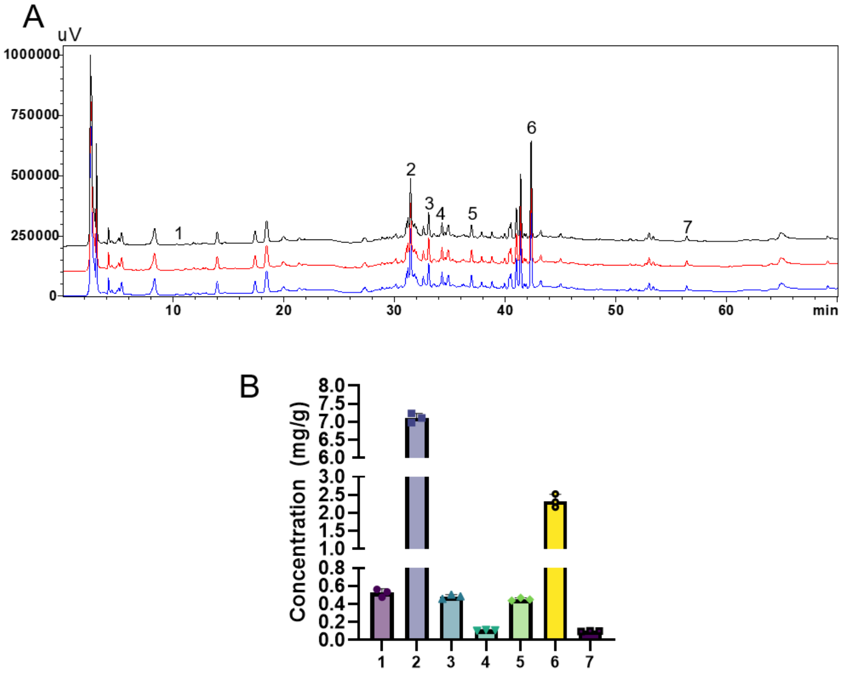

Figure 4. Quantification of marker compounds

Note: Number 1~7 are Rhmannioside D, Paeoniflorin, Calycosin glucoside, (-)-Syringaresnol-4-O-beta-D-apiofuranosyl-(1→2)-beta-D-glucopyranoside, Curculigoside, Icariin and Baohuoside I spectral peaks.

3.2.4 Chromatogram comparison and similarity evaluation of different KXN extracts.

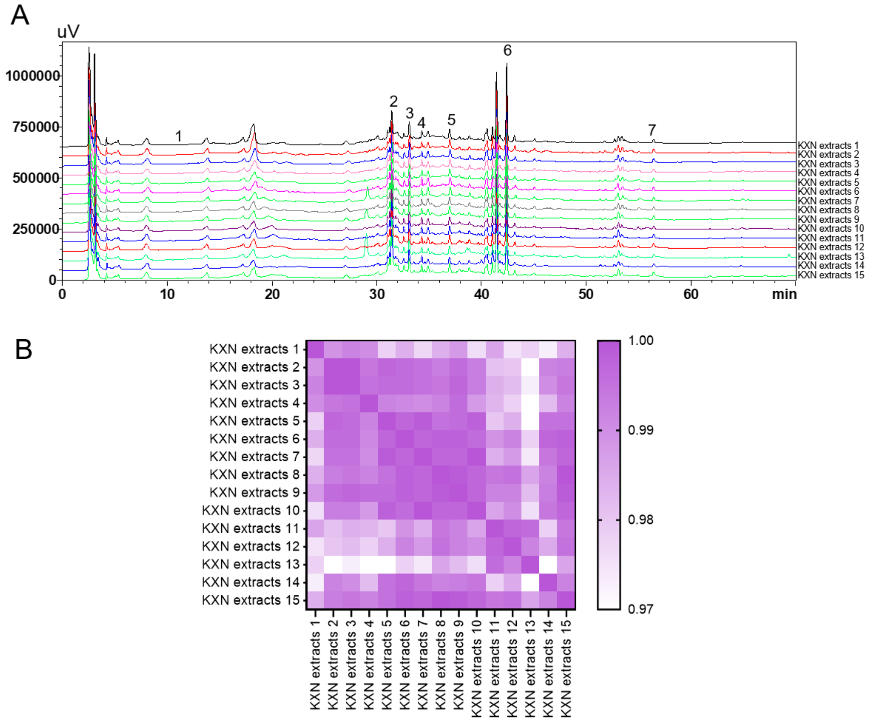

Figure 5. Quantification of marker compounds

Note: Number 1~7 are Rhmannioside D, Paeoniflorin, Calycosin glucoside, (-)-Syringaresnol-4-O-beta-D-apiofuranosyl-(1→2)-beta-D-glucopyranoside, Curculigoside, Icariin and Baohuoside I spectral peaks.
