# Supplementary material for: Kunxinning granules alleviate perimenopausal syndrome by supplementing estrogen deficiency
Source: Front Pharmacol. 2025 Mar 26;16:1554479. doi: 10.3389/fphar.2025.1554479 (PMC11979375; doi:10.3389/fphar.2025.1554479)
Supplement: Supplementary file 3 [file DataSheet3.pdf]

Table 1 Chemical components of KXN

No.	t_R/min	Measured value	Precursor ions	Formula	Theoretical value	Compound	Fragment ion	CAS No.
1	0.88	179.0573	$[M-H]^-$	$C_6H_{12}O_6$	179.0556	Inositol	160.9172	551-72-4
2	0.90	387.1156	$[M-H]^-$	$C_{17}H_{24}O_{10}$	387.1291	Geniposide	225.0657	24512-63-8
3	0.92	397.0942	$[M-H]^-$	$C_{22}H_{22}O_7$	397.1287	Baohuosu	397.0804, 259.0176, 191.0193	119730-90-4
4	1.42	191.0221	$[M-H]^-$	$C_6H_8O_7$	191.0192	Citric acid	173.0098, 129.0203	77-92-9
5	1.54	191.0193	$[M-H]^-$	$C_6H_8O_7$	191.0192	Citric acid isomer	173.0063, 129.0203, 111.0083	-
6	1.96	685.2255	$[M-H]^-$	$C_{27}H_{42}O_{20}$	685.2191	Rehmannioside D	517.1172, 365.1054	81720-08-3
7	2.10	169.0146	$[M-H]^-$	$C_7H_6O_5$	169.0137	Gallic acid	125.0256	149-91-7
8	2.92	509.1918	$[M-H]^-$	$C_{21}H_{34}O_{14}$	509.1870	Rehmannioside C	449.1263, 179.0573	81720-07-2
9	3.12	371.1020	$[M+Na]^+$	$C_{15}H_{24}O_9$	371.1318	Ajugol	191.0596	52949-83-4
10	3.69	527.1464	$[M-H]^-$	$C_{23}H_{28}O_{14}$	527.1401	6'-O-galloyldesbenzoyl paeoniflorin	403.0494, 169.0146, 125.0256	262350-51-6
11	3.85	471.1115	$[M+Na]^+$	$C_{19}H_{28}O_{12}$	471.1479	Anacardoside isomer	309.0966, 125.0504	164991-86-0
12	4.21	309.0714	$[M+Na]^+$	$C_{13}H_{18}O_7$	309.0950	Sakakin	125.0573	21082-33-7
13	4.31	125.0504	$[M+H]^+$	$C_7H_8O_2$	125.0603	Guaiacol	110.0286	90-05-1
14	4.84	417.1438	$[M-H]^-$	$C_{22}H_{26}O_8$	417.1549	(-)-Syringaresinol	181.0548	6216-81-5
15	5.10	342.1419	$[M+H]^+$	$C_{20}H_{23}O_4N$	342.1705	Magnoflorine	297.0087, 282.0677, 265.0652	2141-09-5
16	5.59	495.1552	$[M-H]^-$	$C_{23}H_{28}O_{12}$	495.1508	Oxypaeoniflorin	465.1453, 333.1017, 281.0671, 177.0571, 165.0555, 137.0257	39011-91-1
17	5.69	317.0785	$[M+H]^+$	$C_{16}H_{12}O_7$	317.0661	Isorhamnetin	302.0945	480-19-3
18	5.83	525.1642	$[M+HCOO]^-$	$C_{23}H_{28}O_{11}$	525.1608	Paeoniflorine isomer	479.1608, 449.1176	23180-57-6
19	5.94	337.0939	$[M-H]^-$	$C_{16}H_{18}O_8$	337.0923	5-p-coumaroylquinic-acid	191.0561, 173.0475, 163.0412, 137.0257	1899-30-5
20	6.03	353.0884	$[M-H]^-$	$C_{16}H_{18}O_9$	353.0873	Neochlorogenic-acid	191.0589, 179.0353, 135.0458	906-33-2
21	6.33	525.1688	$[M+HCOO]^-$	$C_{23}H_{28}O_{11}$	525.1608	Albiflorin	479.1608, 357.1246, 283.0859, 121,0305	39011-90-0
22	6.56	711.2562	$[M-H]^-$	$C_{33}H_{44}O_{17}$	711.2500	(-) - Syringol-4-o-β- D-carvacosyl -(1 → 2)- β- D-glucopyranoside	417.1605, 181.0521	136997-64-3
23	6.63	579.2137	$[M-H]^-$	$C_{28}H_{36}O_{13}$	579.2078	(-)-Syringaresinol4-O-β-D-glucopyranoside	449.1481, 417.1563, 181.0493	137038-13-2
24	6.68	525.1688	$[M+HCOO]^-$	$C_{23}H_{28}O_{11}$	525.1608	Paeoniflorine	449.1524, 327.1119, 165.0581, 121.0305	23180-57-6
25	6.79	475.1307	$[M+HCOO]^-$	$C_{22}H_{22}O_9$	475.1231	Ononin	267.0677	486-62-4
26	7.28	593.1547	$[M-H]^-$	$C_{27}H_{30}O_{15}$	593.1512	Quercetin3,7-O-rhamnopyranoside	447.0977, 301.0397	28638-13-3
27	7.29	181.0352	$[M+H]^+$	$C_9H_8O_4$	181.0501	Theobromine		83-67-0
28	7.53	447.0938	$[M+H]^+$	$C_{22}H_{22}O_{10}$	447.1291	Calycosin-7-O-β-	285.0525,	20633-67-4

						D-glucoside	270.0334, 213.0376	
29	7.53	285.0525	$[M+H]^+$	$C_{16}H_{13}O_5$	285.0763	Wogonin	270.0334, 183.0345	632-85-9
30	7.56	283.0618	$[M-H]^-$	$C_{16}H_{12}O_5$	283.0607	Calycosin	268.0391, 239.0355, 211.0403, 195.0477	20575-57-9
31	7.81	503.1127	$[M+Na]^+$	$C_{23}H_{28}O_{11}$	503.1529	Albiflorin isomer	341.0789	-
32	8.30	465.1453	$[M-H]^-$	$C_{22}H_{26}O_{11}$	465.1397	Curculigoside	327.1082, 204.9773, 123.0461	85643-19-2
33	8.38	631.1736	$[M-H]^-$	$C_{30}H_{32}O_{15}$	631.1663	Galloyl-paeoniflorin	613.1644, 509.1364, 491.1263, 463.1293	122965-41-7
34	8.87	711.2562	$[M-H]^-$	$C_{33}H_{44}O_{17}$	711.2500	(-)-Syringaresinol4-O-β-D-glucopyranoside isomer	417.1605, 181.0548	-
35	8.88	547.1693	$[M+Na]^+$	$C_{21}H_{32}O_{15}$	547.1639	Rehmannioside A isomer	347.1309	-
36	8.89	547.1693	$[M+Na]^+$	$C_{26}H_{36}O_{11}$	547.2155	Icariside E3	489.1534, 205.0713	137822-23-2
37	9.02	463.0896	$[M-H]^-$	$C_{21}H_{20}O_{12}$	463.0877	Hyperoside	300.0280, 271.0277, 255.0323, 151.0040	482-36-0
38	9.20	417.1564	$[M-H]^-$	$C_{18}H_{26}O_{11}$	417.1397	Orcinol-1-O-β-D-apiofuranosyl-(1→6)-β-D-glucopyranoside	109.0280	868557-54-4
39	9.22	547.1693	$[M+Na]^+$	$C_{21}H_{32}O_{15}$	547.1639	Rehmannioside A isomer	347.1042	-
40	9.24	547.1693	$[M+Na]^+$	$C_{26}H_{36}O_{11}$	547.2155	Icariside E3 isomer	489.1670, 205.0831	-
41	9.28	301.0006	$[M-H]^-$	$C_{14}H_6O_8$	300.9984	Ellagic acide	283.9997, 229.0150	476-66-4
42	9.48	623.2043	$[M-H]^-$	$C_{29}H_{36}O_{15}$	623.1976	Acteoside	461.1704, 161.0264	61276-17-3
43	9.48	623.2043	$[M-H]^-$	$C_{29}H_{36}O_{15}$	623.1976	Isoacteoside	461.1703, 161.0264	61303-13-7
44	9.48	623.2043	$[M-H]^-$	$C_{29}H_{36}O_{15}$	623.1976	Isoverproside	461.1703, 161.0264	61303-13-7
45	9.67	939.1226	$[M-H]^-$	$C_{41}H_{32}O_{26}$	939.1104	Pentagalloyl-glucose	617.0902, 465.0791, 295.0512, 169.0146	14937-32-7
46	9.73	503.1127	$[M+Na]^+$	$C_{23}H_{28}O_{11}$	503.1529	Albiflorin isomer	381.0847, 341.0751, 219.0484	-
47	9.93	631.1736	$[M-H]^-$	$C_{30}H_{32}O_{15}$	631.1663	Galloylpaeoni-florin isomer	509.1502, 463.1248, 271.0547	-
48	10.18	503.1127	$[M+Na]^+$	$C_{23}H_{28}O_{11}$	503.1529	Albiflorin isomer	381.0887, 341.0826, 219.0484	-
49	10.48	503.1127	$[M+Na]^+$	$C_{23}H_{28}O_{11}$	503.1529	Albiflorin isomer	487.1952, 341.1091	-
50	10.86	367.0872	$[M+Na]^+$	$C_{16}H_{24}O_8$	367.1369	Mudanpioside F	205.1124	172670-08-5
51	10.88	163.0412	$[M-H]^-$	$C_9H_8O_3$	163.0395	p-Hydroxy-cinnamic acid	119.0495	7400-08-0
52	11.03	677.2142	$[M-H]^-$	$C_{32}H_{38}O_{16}$	677.2082	Demethylicaritin-7-O-sophoroside	530.1925, 370.1111	101072-83-7
53	11.03	677.2142	$[M-H]^-$	$C_{32}H_{38}O_{16}$	677.2082	Hexandraside E	515.1606, 353.1076	139955-75-2
54	11.25	447.0977	$[M-H]^-$	$C_{21}H_{20}O_{11}$	447.0933	Quercetin 3-rhamnoside	301.0361, 284.0445,	522-12-3

							255.0323	
55	11.60	823.2723	$[M-H]^-$	$C_{38}H_{48}O_{20}$	823.2661	Rouhuoside	661.2167, 515.1606, 353.1076	131862-37-8
56	11.90	523.1858	$[M-H]^-$	$C_{21}H_{32}O_{15}$	523.2663	Rehmannioside A	323.1022, 199.1009	81720-05-0
57	12.07	793.2632	$[M-H]^-$	$C_{37}H_{46}O_{19}$	793.2555	Epimedoside E	631.2095, 352.0925	39049-19-9
58	12.10	431.1008	$[M+H]^+$	$C_{22}H_{22}O_9$	431.1342	Ononin	269.0623	486-62-4
59	12.11	269.0623	$[M+H]^+$	$C_{16}H_{13}O_4$	269.0814	Formononetin	254.0390, 237.0399	485-72-3
60	12.17	267.0677	$[M-H]^-$	$C_{16}H_{12}O_4$	267.0658	Formononetin isomer	252.0456, 223.0427, 195.0477	-
61	12.41	385.0988	$[M+Na]^+$	$C_{15}H_{22}O_{10}$	385.1111	Catalpol	355.1005, 223.0653, 203.0597	2415-24-9
62	12.61	807.2815	$[M-H]^-$	$C_{38}H_{48}O_{19}$	807.2712	Diphylloside B	661.2220, 645.2285, 499.1685, 514.1526, 353.1076	-
63	12.72	661.2167	$[M-H]^-$	$C_{32}H_{38}O_{15}$	661.2138	Epimedoside A	514.1526, 499.1685, 395.1136, 353.1037	39012-04-9
64	13.00	517.1289	$[M+Na]^+$	$C_{19}H_{26}O_{15}$	517.1169	Galloylsucrose	355.0927	-
65	13.07	807.2815	$[M-H]^-$	$C_{38}H_{48}O_{19}$	807.2712	Epimedin B	645.2285, 367.1232, 351.0903, 323.1022	110623-73-9
66	13.26	385.0988	$[M+Na]^+$	$C_{15}H_{22}O_{10}$	385.1111	Catalpol isomer	355.0889, 223.0683, 203.0656	-
67	13.68	629.1945	$[M-H]^-$	$C_{31}H_{34}O_{14}$	629.1876	Mudanpioside J	599.1794, 507.1469, 477.1548, 461.2407	262350-52-7
68	13.81	485.1012	$[M+Na]^+$	$C_{23}H_{26}O_{10}$	485.1424	Lactiflorin isomer	105.0306	-
69	13.88	167.0588	$[M+H]^+$	$C_9H_{10}O_3$	167.0708	Paeonol	149.0061, 124.8925, 121.0297	552-41-0
70	13.90	485.1012	$[M+Na]^+$	$C_{23}H_{26}O_{10}$	485.1424	Lactiflorin	105.0285	1361049-59-3
71	13.95	983.3507	$[M-H]^-$	$C_{45}H_{60}O_{24}$	983.3402	Acuminatoside	675.2385, 367.1350, 211.0670	142735-71-5
72	13.98	485.1103	$[M+Na]^+$	$C_{23}H_{26}O_{10}$	485.1424	Lactiflorin isomer	105.0285	-
73	14.56	465.2160	$[M-H]^-$	$C_{21}H_{22}O_{12}$	465.2130	Taxifolin-7-O-glucoside	285.1534, 259.1725, 241.1591	14292-40-1
74	14.76	477.1047	$[M+HCOO]^-$	$C_{21}H_{20}O_{10}$	477.1028	Genistin	477.1422, 431.0998, 301.0376, 269.0456, 167.7634	529-59-9
75	14.85	599.1844	$[M-H]^-$	$C_{30}H_{32}O_{13}$	599.1770	Mudanpioside C	569.1843, 477.1458, 281.0706, 165.0581, 137.0257	172760-03-1
76	15.34	529.1746	$[M-H]^-$	$C_{27}H_{30}O_{11}$	529.1710	Icariside I	367.1232, 309.0795, 297.0454	56725-99-6
77	16.24	285.0525	$[M+H]^+$	$C_{16}H_{13}O_5$	285.0763	Calycosin	270.0334, 253.0319	20575-57-9
78	16.72	839.2284	$[M+H]^+$	$C_{39}H_{50}O_{20}$	839.2974	Epimedin A	677.1909, 531.1440, 369.1056, 313.0495	110623-72-8

79	16.76	675.2385	$[M-H]^-$	$C_{33}H_{40}O_{15}$	675.2289	Sagittatoside A	367.1193, 351.0492, 323,0949	118525-35-2
80	17.54	839.2284	$[M+H]^+$	$C_{39}H_{50}O_{20}$	839.2974	Epimedin A isomer	677.1855, 531.1440, 369.1056, 313.0458	-
81	17.61	675.2385	[M-H]-	$C_{33}H_{40}O_{15}$	675.2289	Icariin	529.1887, 513.1673, 367.1232	489-32-7
82	18.27	645.2285	$[M-H]^-$	$C_{32}H_{38}O_{14}$	645.2183	Sagittatoside B	366.1156, 351.0903, 323.0986	118525-36-3
83	19.00	823.2366	$[M+Na]^+$	$C_{36}H_{48}O_{20}$	823.2637	Jionoside A1	677.1909	120444-60-2
84	19.05	823.2366	$[M+Na]^+$	$C_{36}H_{48}O_{20}$	823.2637	Jionoside A1 isomer	677.1909	-
85	19.12	867.3004	$[M+HCOO]^-$	$C_{39}H_{50}O_{19}$	867.2928	Baohuoside VI	659.2437, 366.1156, 351.0903, 323.0949	119760-73-5
86	19.14	659.2437	$[M-H]^-$	$C_{33}H_{40}O_{14}$	659.2345	2″-O-rhamnosylicariside II	366.1156, 351.0903, 323.0949	135293-13-9
87	19.48	369.1135	$[M+H]^+$	$C_{21}H_{20}O_6$	369.1338	Icaritin	369.1056, 313.0495, 243.0459, 135.0337	118525-40-9
88	19.58	513.1826	$[M-H]^-$	$C_{27}H_{30}O_{10}$	513.1761	Baohuoside I isomer	366.1156, 351.0903, 323.0949, 217.0528	-
89	5.99	269.0824	$[M-H]^-$	$C_{16}H_{14}O_4$	269.0808	Echinatin	269.1403, 225.1494, 181.1594, 125.0961	34221-41-5
90	20.56	879.3061	$[M-H]^-$	$C_{41}H_{52}O_{21}$	879.2923	Epimedin I	717.2432, 367.1232	205445-00-7
91	22.01	531.1926	$[M-H]^-$	$C_{27}H_{32}O_{11}$	531.1872	Icaritin-3-O-rhamnopyranoside isomer	384.1239, 367.1232, 341.1021, 311.0591	-
92	22.43	717.2488	$[M-H]^-$	$C_{35}H_{42}O_{16}$	717.2395	Sagittatoside C isomer	513.1873, 367.1232	-
93	22.90	687.2371	$[M+HCOO]^-$	$C_{29}H_{38}O_{16}$	687.2142	Isomalto-paeoniflorin	611.2189, 593.1946, 283.0583, 121.0305	262350-54-9
94	23.14	819.2820	$[M-H]^-$	$C_{39}H_{48}O_{19}$	819.2712	Anhydroicaritin-3-O-rhamnoside (1-2)-furanacid-7-O-glucoside	529.1793, 367.1232, 289.0951	-
95	23.95	385.0988	$[M+Na]^+$	$C_{15}H_{22}O_{10}$	385.1111	Catalpol	355.0851, 223.0879, 203.0539	2415-24-9
96	24.09	269.0623	$[M+H]^+$	$C_{16}H_{13}O_4$	269.0814	Formononetin isomer	254.0390, 237.0399	-
97	24.24	829.4650	$[M+HCOO]^-$	$C_{45}H_{56}O_{23}$	829.4586	Astragaloside IV	783.3661	84687-43-4
98	24.369	821.2583	$[M-H]^-$	$C_{39}H_{50}O_{19}$	821.2868	Epimedin C	659.2068, 366.1078, 351.0942, 323.0654, 311.0627	110642-44-9
99	24.43	631.2095	$[M-H]^-$	$C_{31}H_{36}O_{14}$	631.2027	Demethylanhydroicaritin-3-O-rhamnopyranosyl-xylopyranoside	352.1002	-
100	24.90	499.1639	$[M-H]^-$	$C_{26}H_{28}O_{10}$	499.1604	Baohuoside II	353.1037, 352.0963, 295.1040	55395-07-8

101	25.12	675.2385	[M-H]-	$C_{33}H_{40}O_{15}$	675.2289	Icariin isomer	529.1887, 513.1673, 367.1232	-
102	25.12	675.2385	$[M-H]^-$	$C_{33}H_{40}O_{15}$	675.2289	Baohuoside VII	367.1232, 352.1002	119730-89-1
103	25.14	659.2340	$[M-H]^-$	$C_{33}H_{40}O_{14}$	659.2340	2″-O-rhamnosyl-icariside II	366.1156, 351.0942, 323.0912	135293-13-9
104	25.54	659.2345	$[M-H]^-$	$C_{33}H_{40}O_{14}$	659.2437	2″-O-rhamnosyl-icariside II isomer	366.1156, 351.0903, 323.0949	-
105	25.54	479.1330	$[M+H]^+$	$C_{23}H_{26}O_{11}$	479.1553	Curculigoside D	179.0633, 161.0503	-
106	25.71	871.4845	$[M+HCOO]^-$	$C_{43}H_{70}O_{15}$	871.4692	Astragaloside II	825.4771	84676-89-1
107	25.71	871.4845	$[M+HCOO]^-$	$C_{43}H_{70}O_{15}$	871.4692	AstragalosideII isomer	825.4713	-
108	25.74	659.2437	$[M-H]^-$	$C_{33}H_{40}O_{14}$	659.2340	2″-O-rhamnosyl-Icariside II isomer	366.1156, 351.0903, 323.0949	-
109	25.99	717.2488	$[M-H]^-$	$C_{35}H_{42}O_{16}$	717.2395	Sagittatoside C isomer	513.1826, 367.1232	-
110	26.20	313.0495	$[M+H]^+$	$C_{17}H_{12}O_6$	313.0712	Curculigoside A	151.0317	85643-19-2
111	26.21	513.1826	$[M-H]^-$	$C_{27}H_{30}O_{10}$	513.1761	Baohuoside I	366.1156, 351.0903, 323.0949, 217.0528	113558-15-9
112	26.28	913.4961	$[M+HCOO]^{-−}$	$C_{45}H_{72}O_{16}$	913.4797	Astragaloside I	867.4873	84680-75-1
113	26.58	913.4900	$[M+HCOO]^{-−}$	$C_{45}H_{72}O_{16}$	913.4797	Astragaloside I isomer	867.4813	-
114	27.03	657.2263	$[M-H]^-$	$C_{33}H_{38}O_{14}$	657.2183	Anhydroicaritin-3-O-rhamnopyranosyl-furanacid isomer	513.1826, 367.1193, 352.0963, 289.1229	-
115	27.05	913.4961	$[M+HCOO]^-$	$C_{45}H_{72}O_{16}$	913.4797	Astragaloside I isomer	867.4873	-

Mass spectrums of chemical components of KXN based on UPLC-Q/TOF-MS

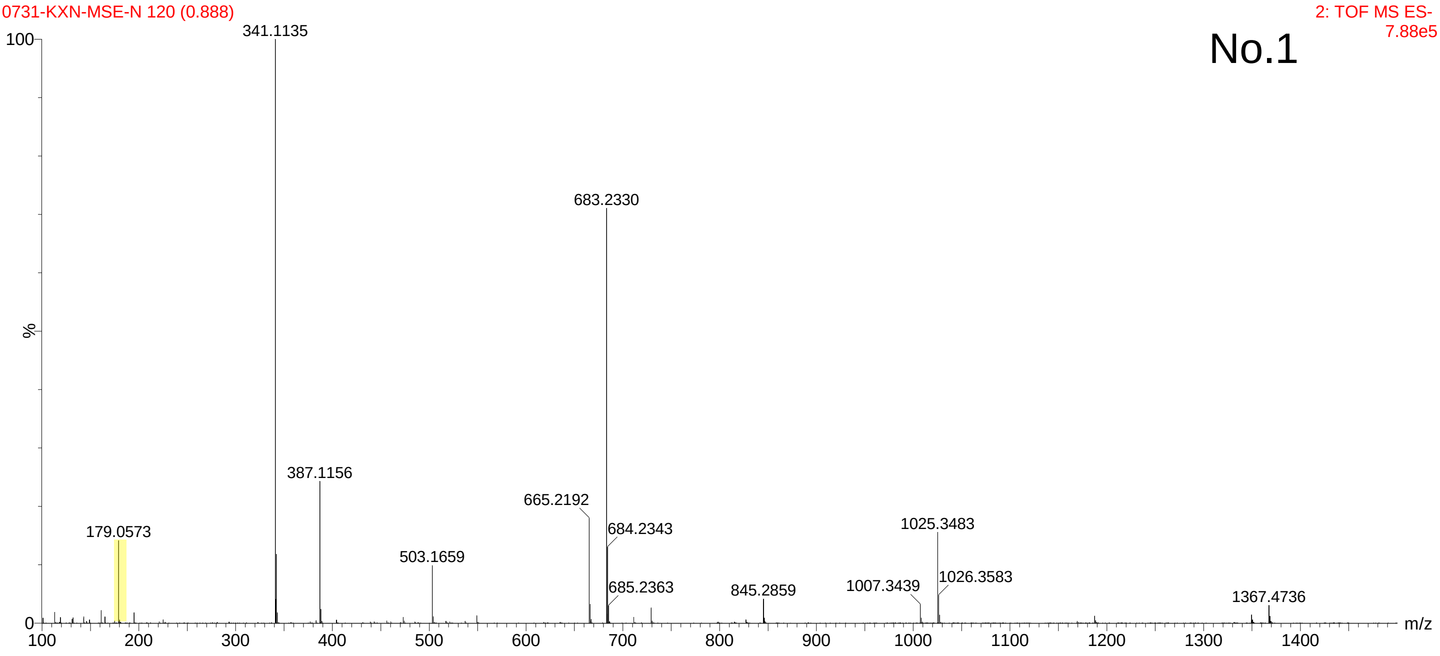

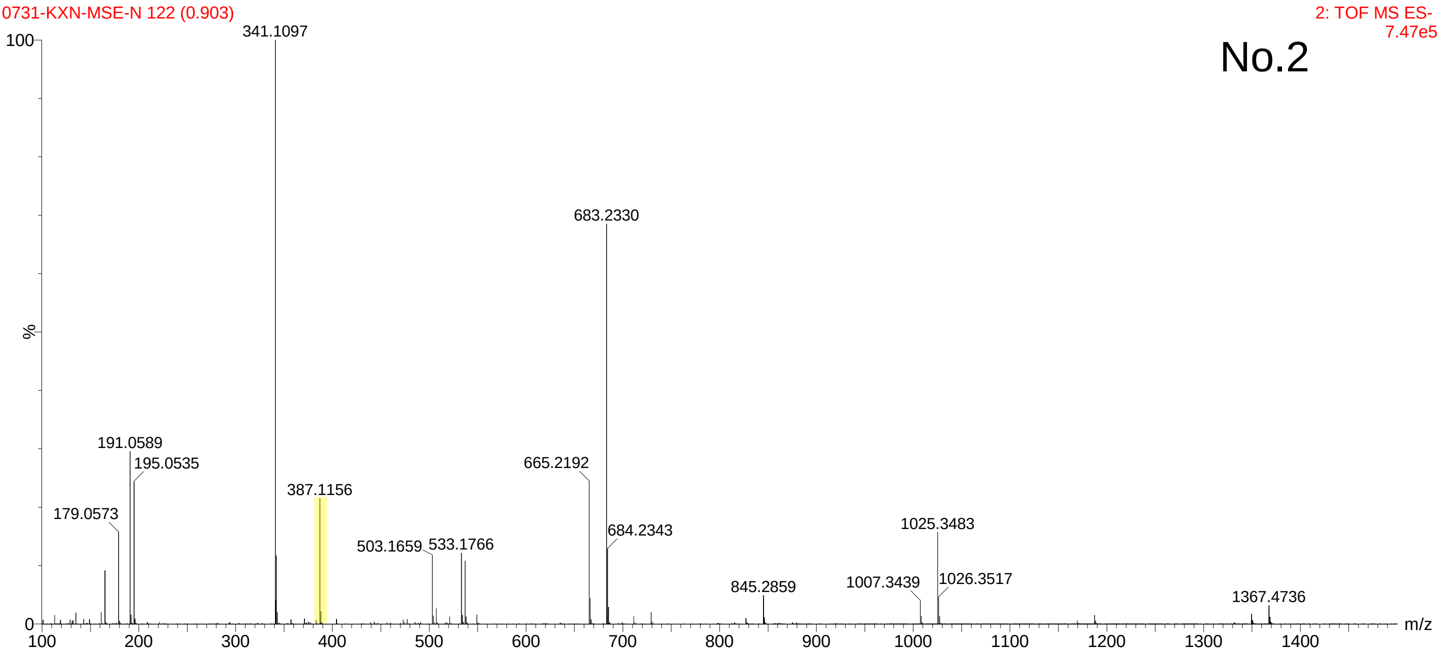

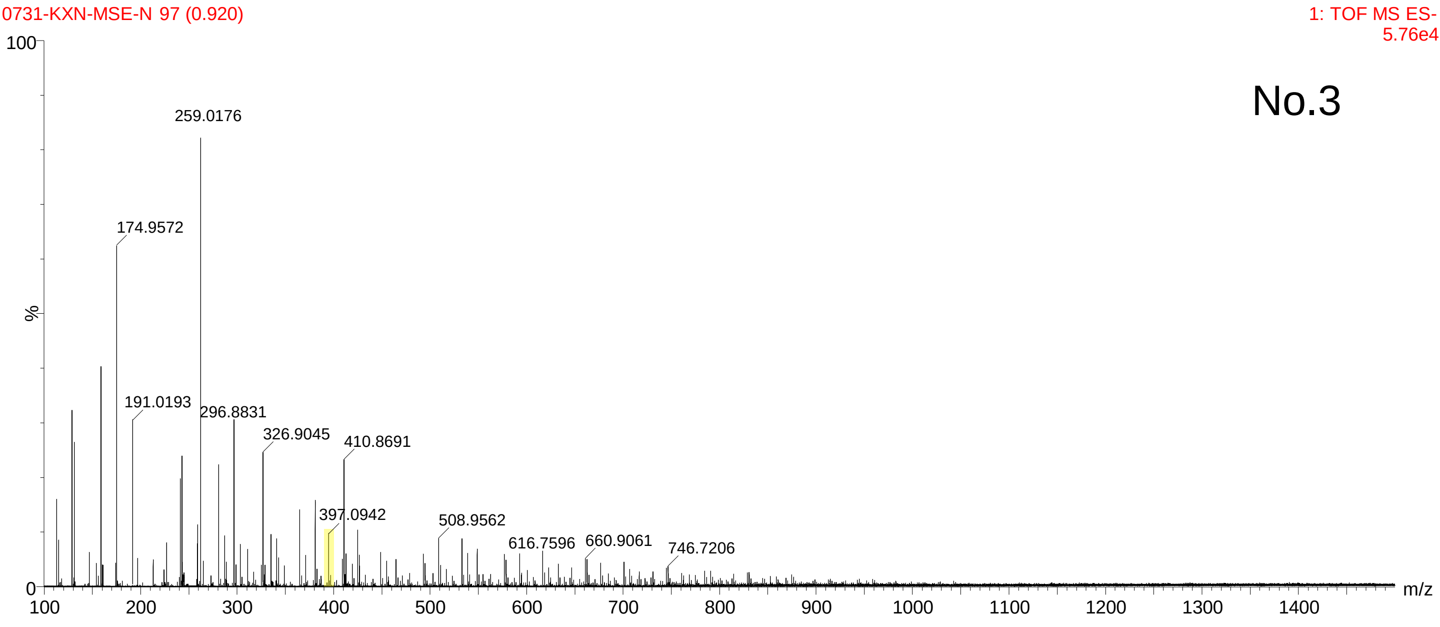

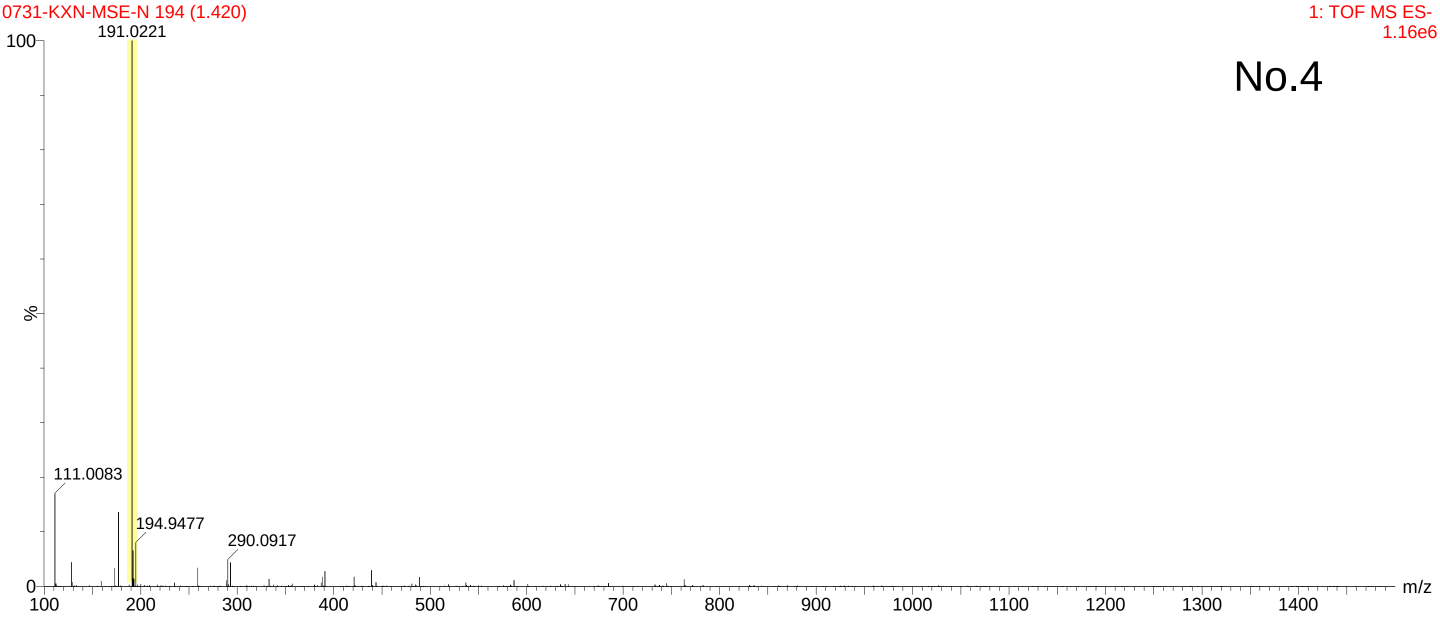
0731-KXN-MSE-N 194 (1.420)
1: TOF MS ES-
1.16e6
No.4
191.0221
111.0083
194.9477
290.0917
100
%
0
100
200
300
400
500
600
700
800
900
1000
1100
1200
1300
1400
m/z

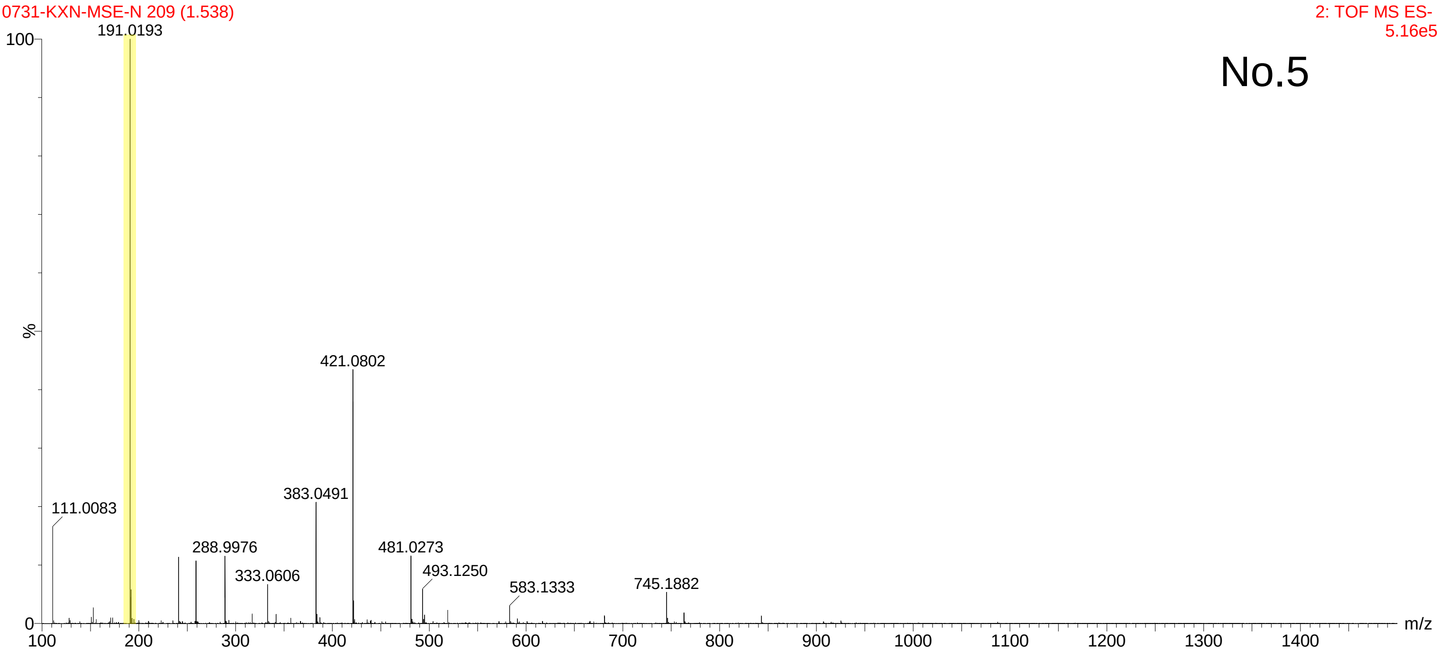
0731-KXN-MSE-N 209 (1.538)
2: TOF MS ES-
5.16e5
No.5
191.0193
421.0802
383.0491
111.0083
288.9976
333.0606
481.0273
493.1250
583.1333
745.1882
100
%
0
100
200
300
400
500
600
700
800
900
1000
1100
1200
1300
1400
m/z

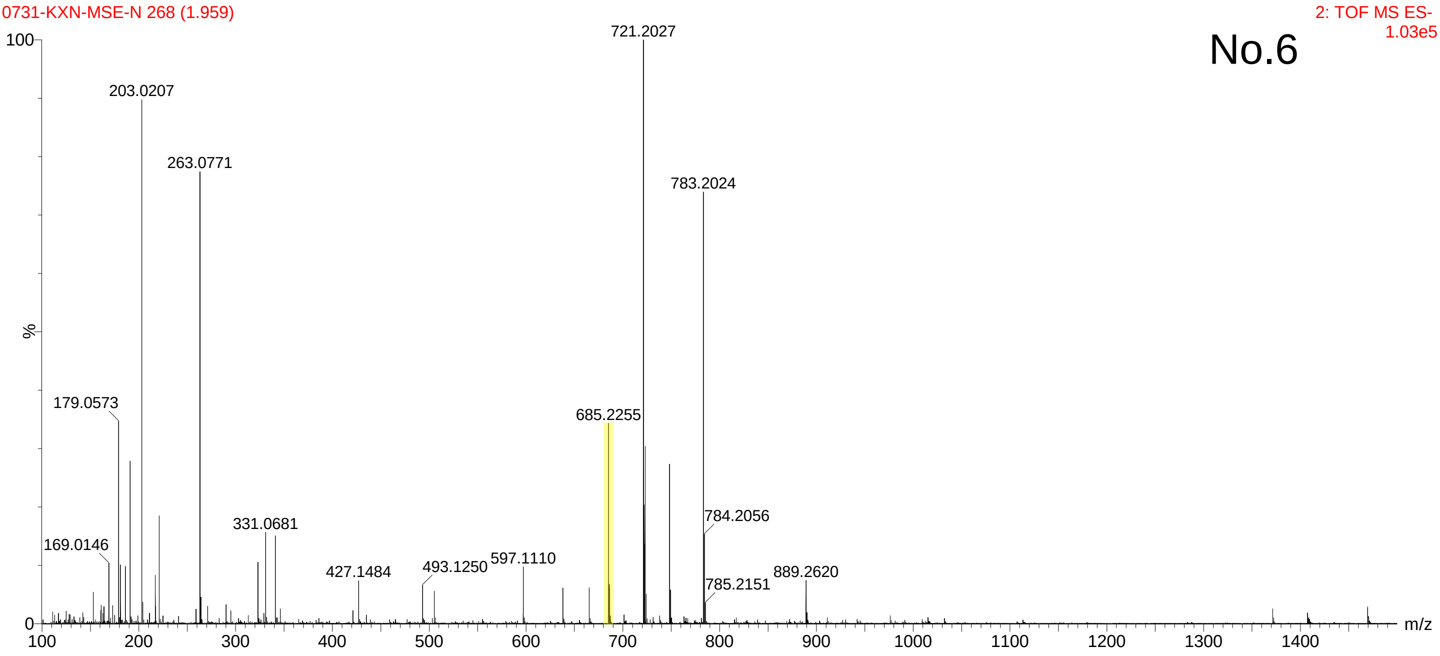
0731-KXN-MSE-N 268 (1.959)
2: TOF MS ES-
1.03e5
No.6
721.2027
203.0207
263.0771
783.2024
179.0573
685.2255
784.2056
331.0681
169.0146
427.1484
493.1250
597.1110
785.2151
889.2620
100
%
0
100
200
300
400
500
600
700
800
900
1000
1100
1200
1300
1400
m/z

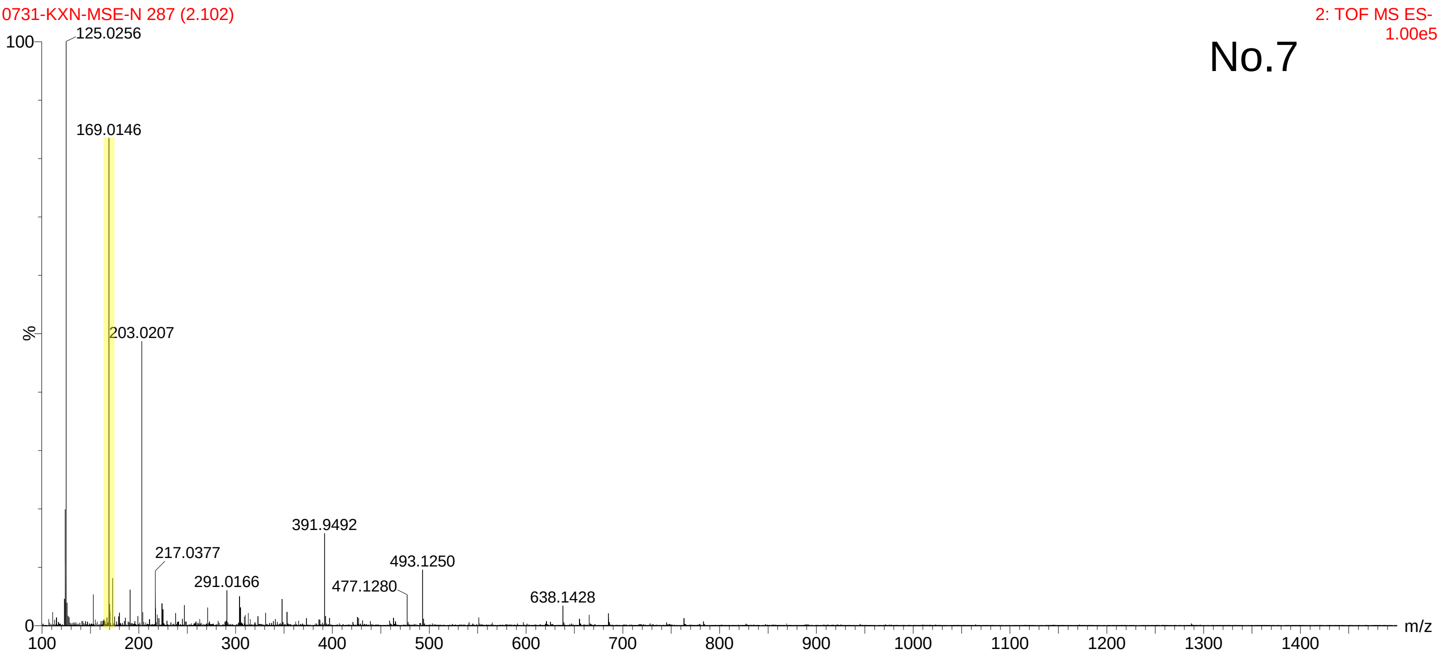
0731-KXN-MSE-N 287 (2.102)
2: TOF MS ES-
1.00e5
No.7
125.0256
169.0146
203.0207
217.0377
291.0166
391.9492
477.1280
493.1250
638.1428
%
m/z

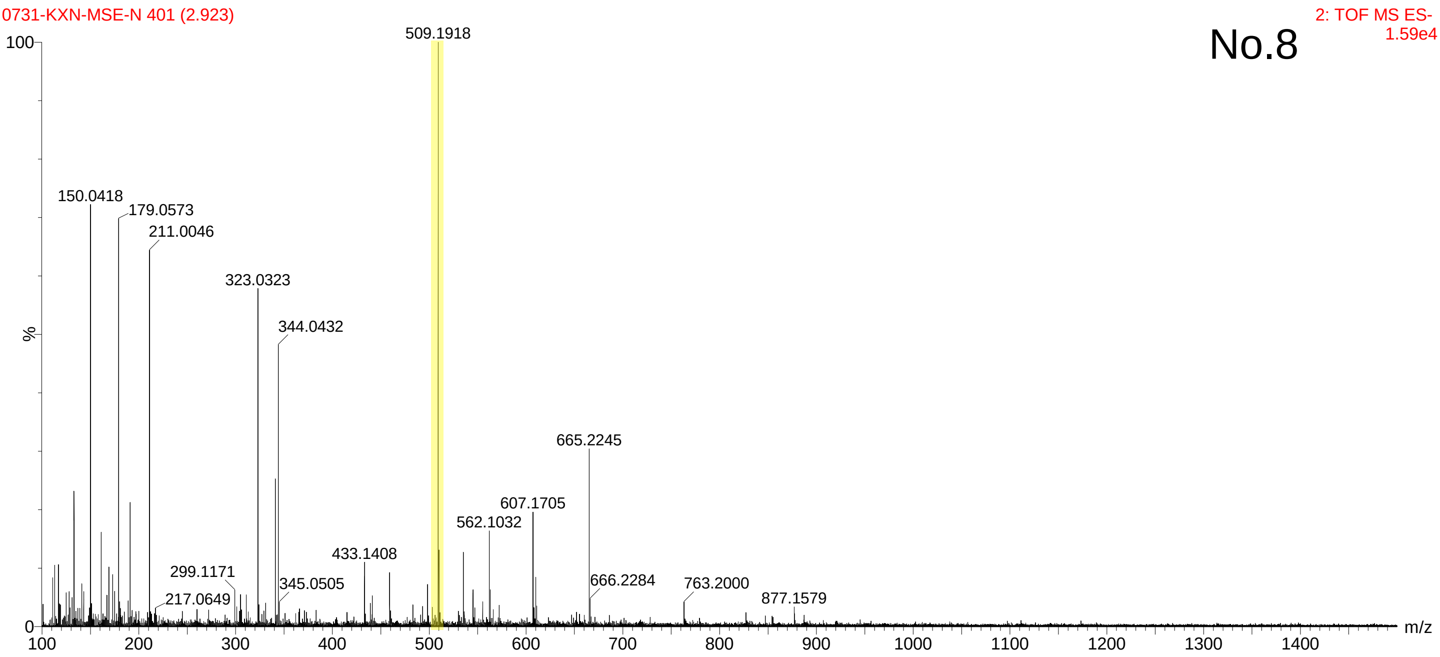
0731-KXN-MSE-N 401 (2.923)
2: TOF MS ES-
1.59e4
No.8
509.1918
150.0418
179.0573
211.0046
323.0323
344.0432
665.2245
607.1705
562.1032
433.1408
299.1171
345.0505
217.0649
666.2284
763.2000
877.1579
%
m/z

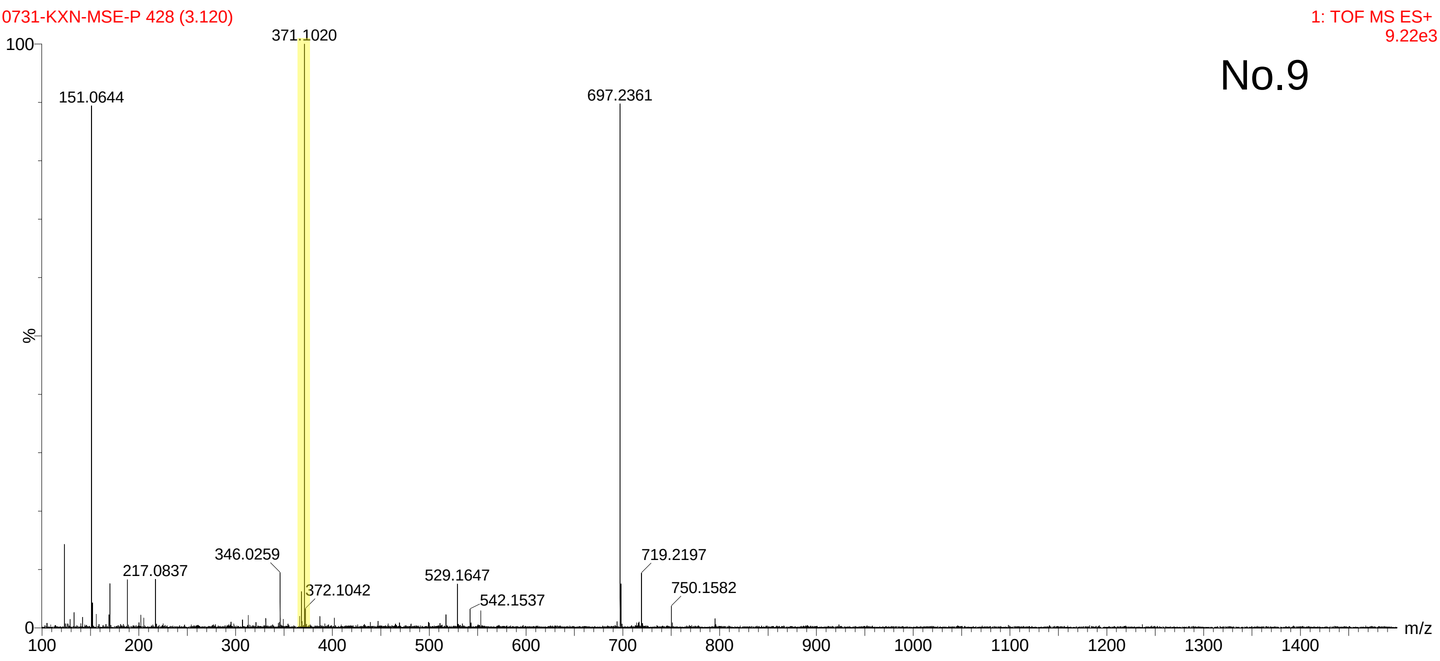
0731-KXN-MSE-P 428 (3.120)
1: TOF MS ES+
9.22e3
No.9
371.1020
151.0644
697.2361
346.0259
217.0837
372.1042
529.1647
542.1537
719.2197
750.1582
%
m/z

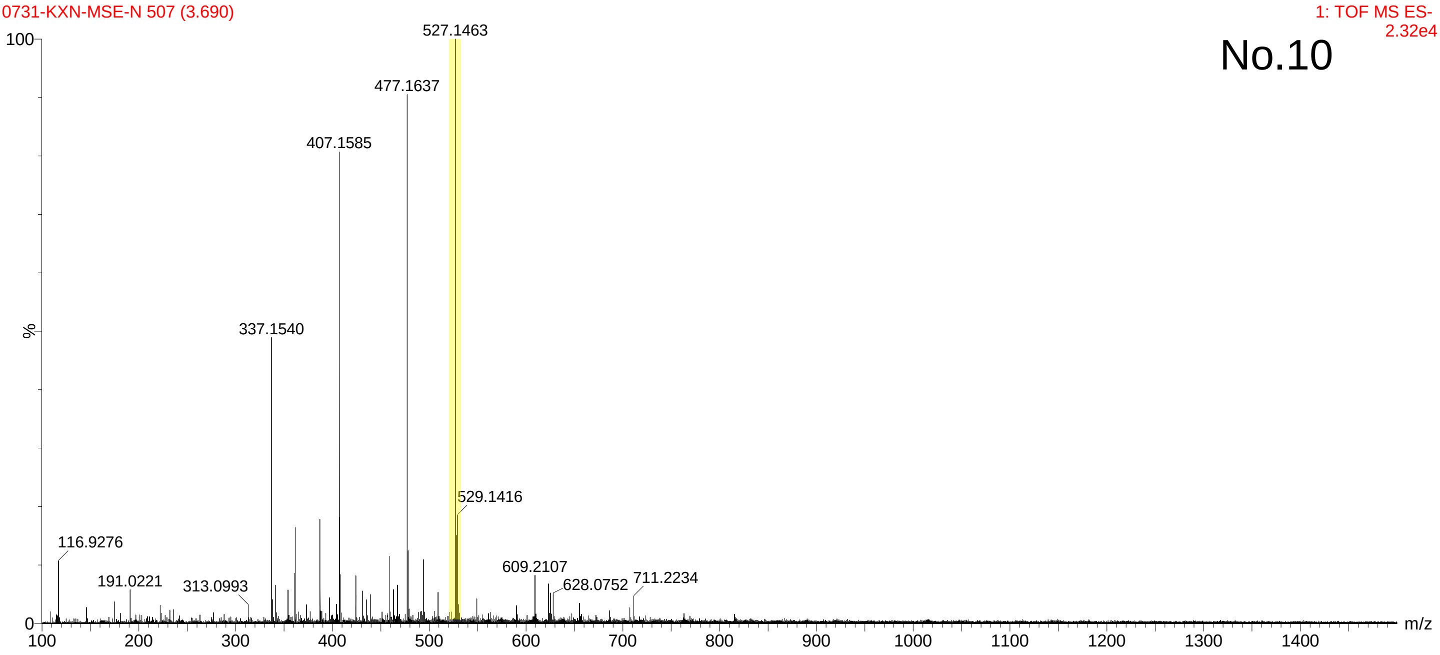

0731-KXN-MSE-N 507 (3.690)
1: TOF MS ES-
2.32e4
No.10
527.1463
477.1637
407.1585
337.1540
529.1416
116.9276
191.0221
313.0993
609.2107
628.0752
711.2234
%
m/z

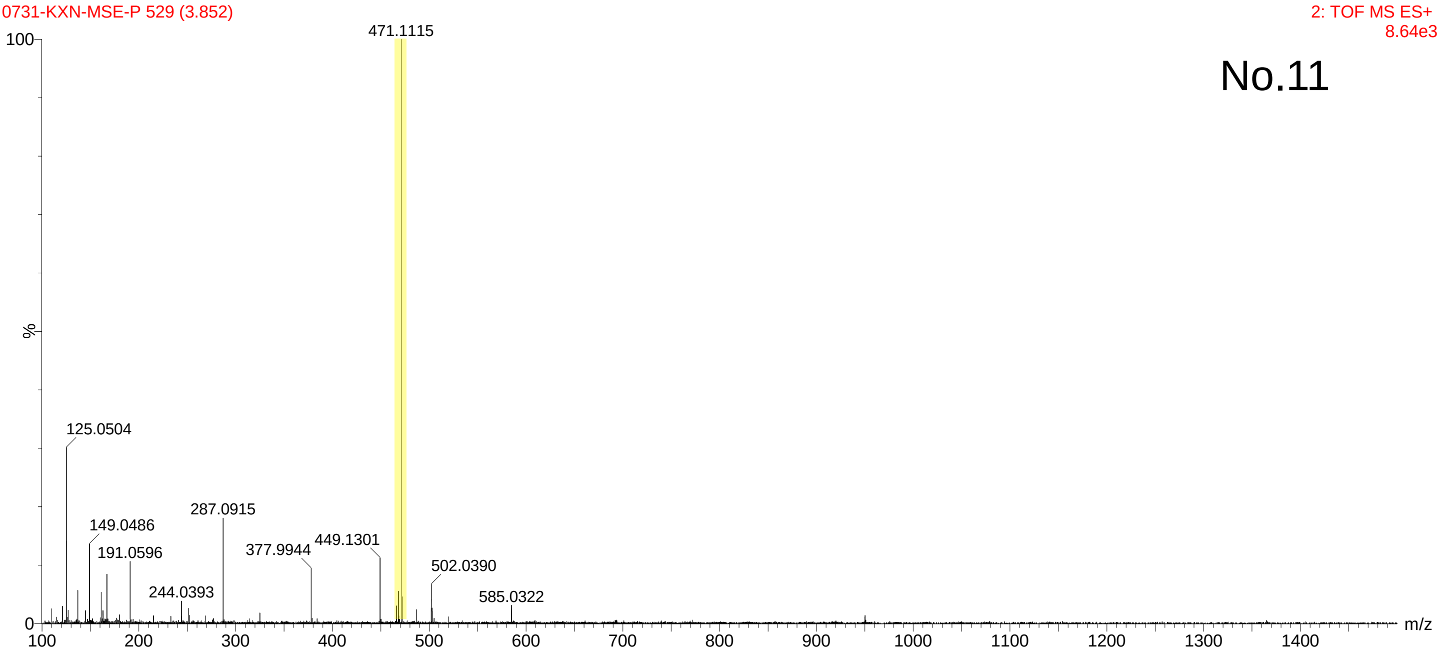

0731-KXN-MSE-P 529 (3.852)
2: TOF MS ES+
8.64e3
No.11
471.1115
125.0504
287.0915
149.0486
191.0596
377.9944
449.1301
502.0390
244.0393
585.0322
%
m/z

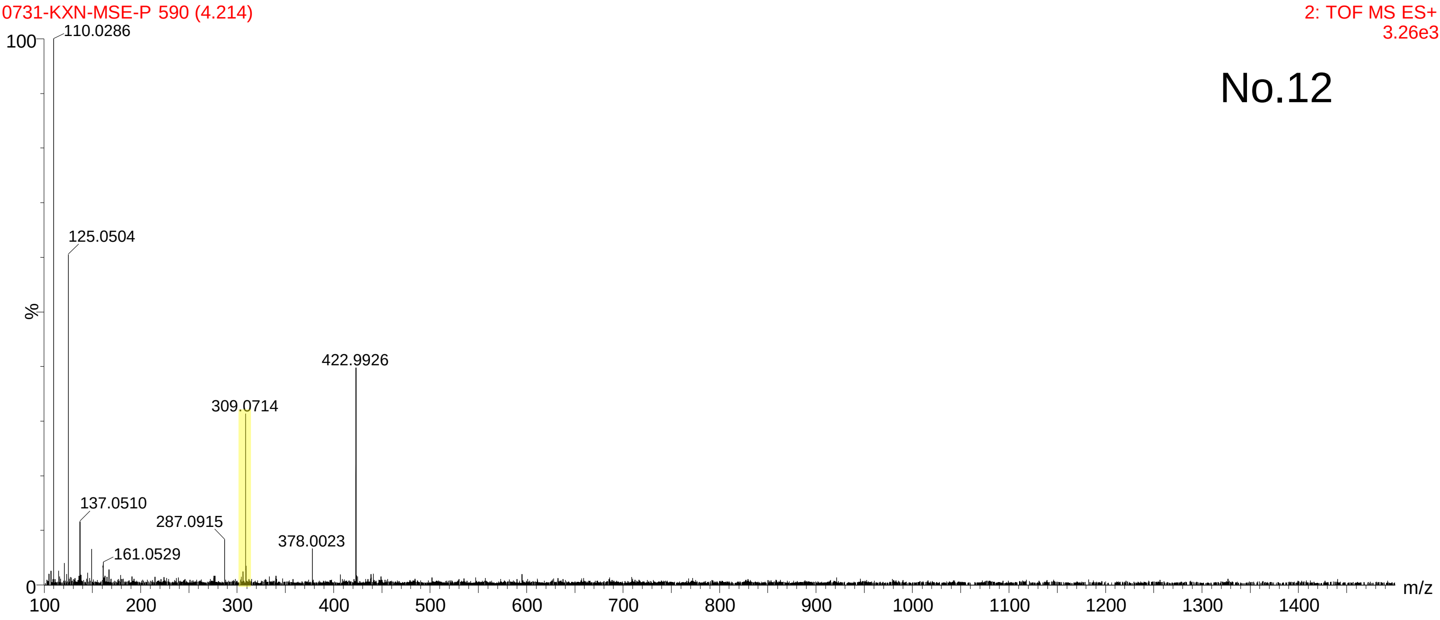

0731-KXN-MSE-P 590 (4.214)
2: TOF MS ES+
3.26e3
No.12
110.0286
125.0504
422.9926
309.0714
137.0510
287.0915
378.0023
161.0529
%
m/z

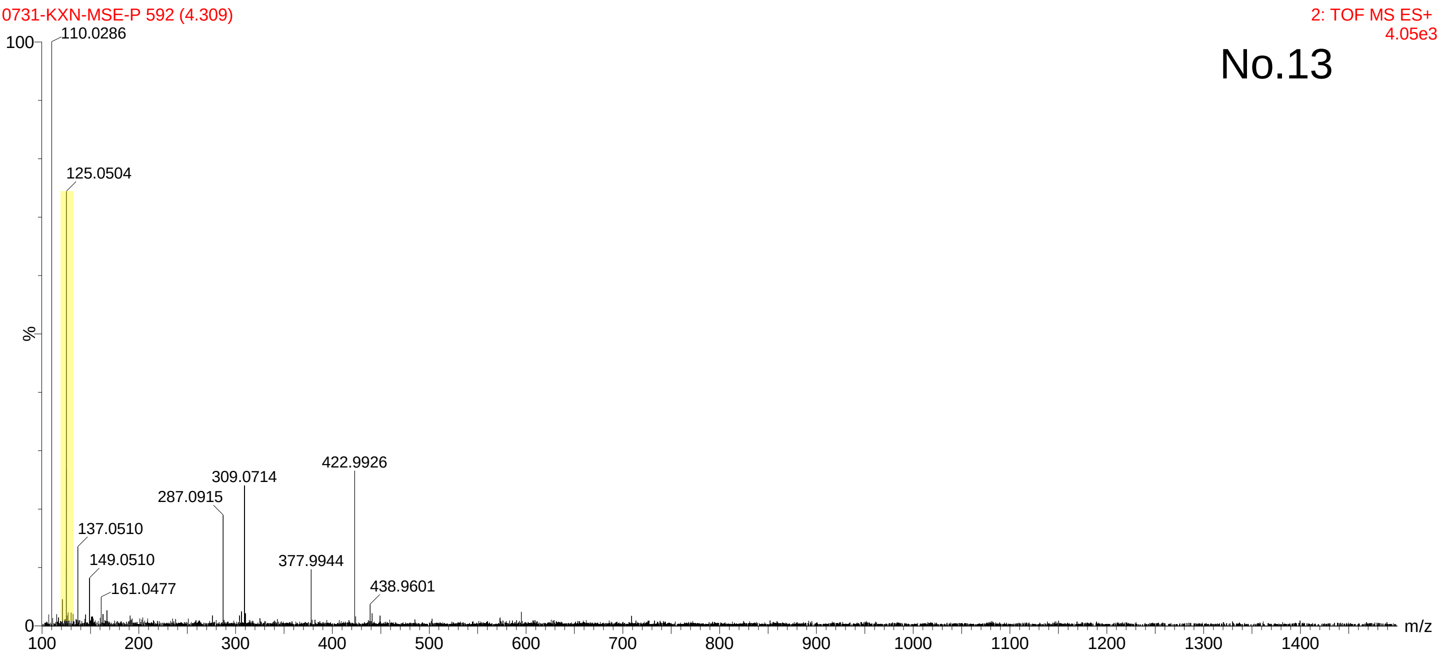
0731-KXN-MSE-P 592 (4.309)
2: TOF MS ES+
4.05e3
No.13
110.0286
125.0504
137.0510
149.0510
161.0477
287.0915
309.0714
377.9944
422.9926
438.9601
100
%
0
m/z
100 200 300 400 500 600 700 800 900 1000 1100 1200 1300 1400

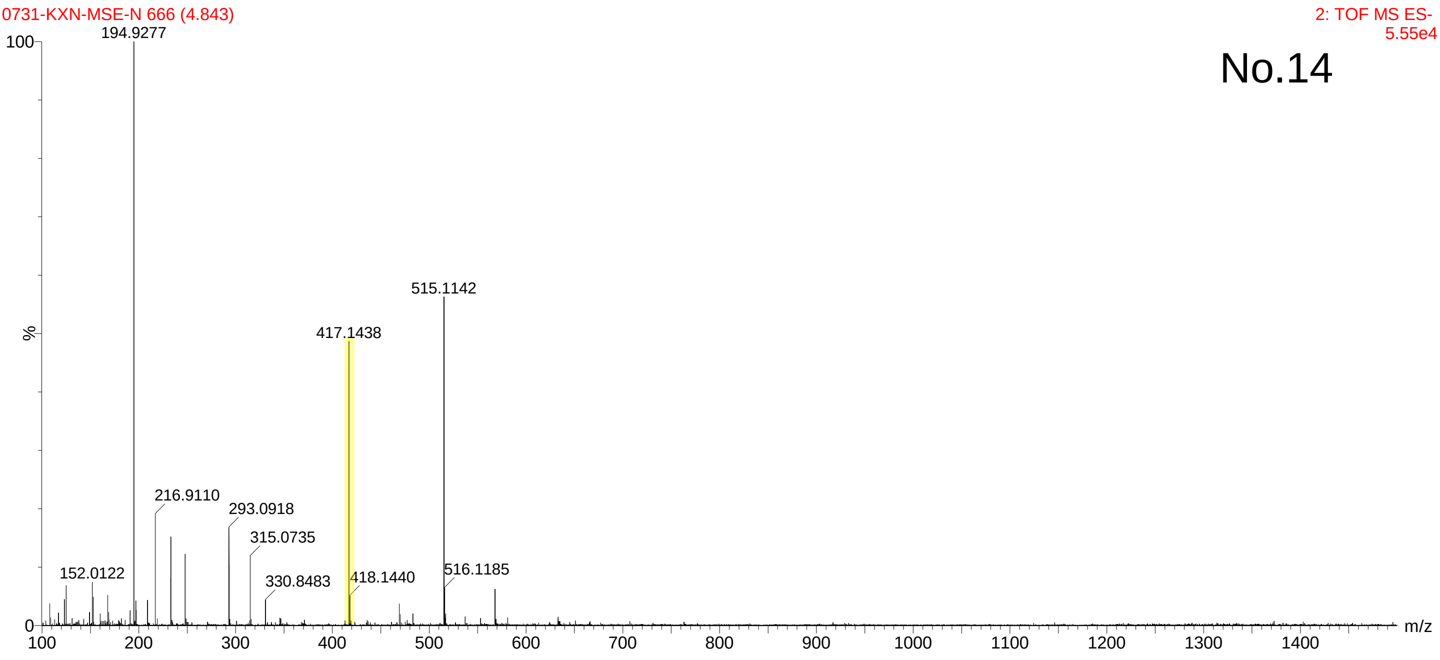
0731-KXN-MSE-N 666 (4.843)
2: TOF MS ES-
5.55e4
No.14
194.9277
515.1142
417.1438
216.9110
293.0918
315.0735
152.0122
330.8483
418.1440
516.1185
100
%
0
m/z
100 200 300 400 500 600 700 800 900 1000 1100 1200 1300 1400

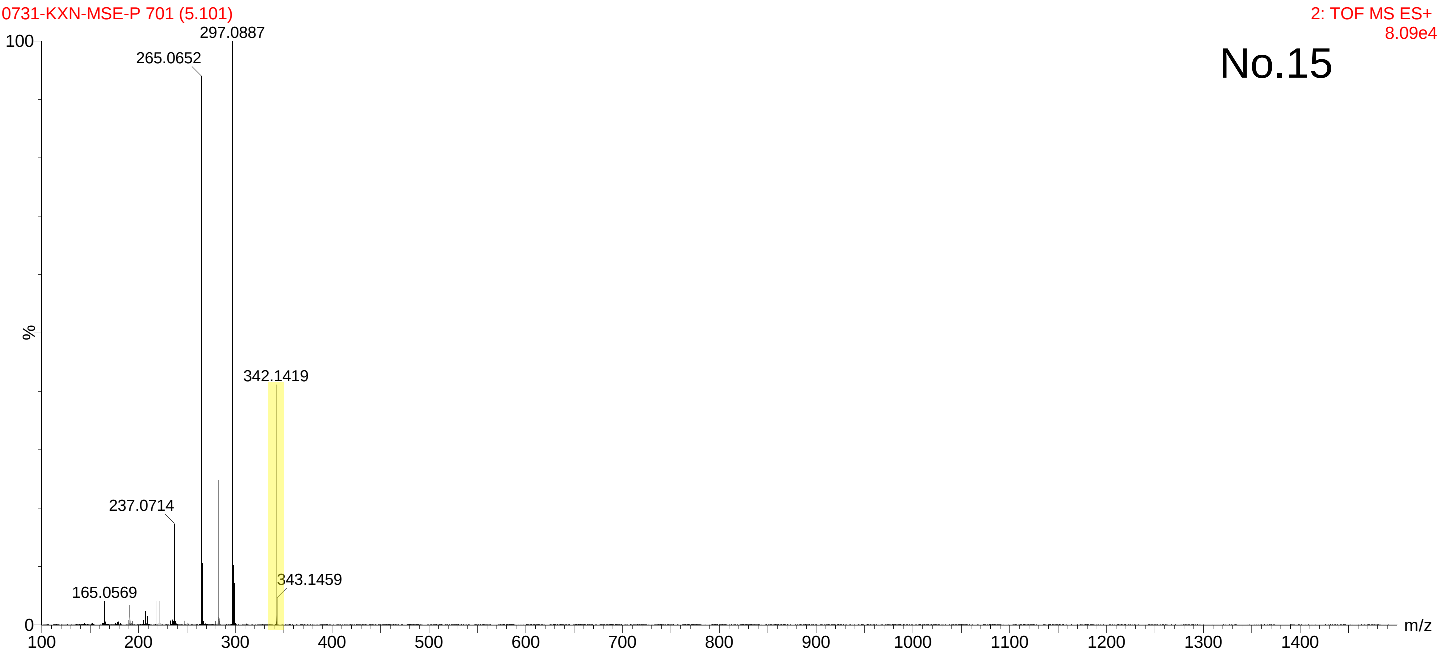
0731-KXN-MSE-P 701 (5.101)
2: TOF MS ES+
8.09e4
No.15
297.0887
265.0652
342.1419
237.0714
165.0569
343.1459
100
%
0
m/z
100 200 300 400 500 600 700 800 900 1000 1100 1200 1300 1400

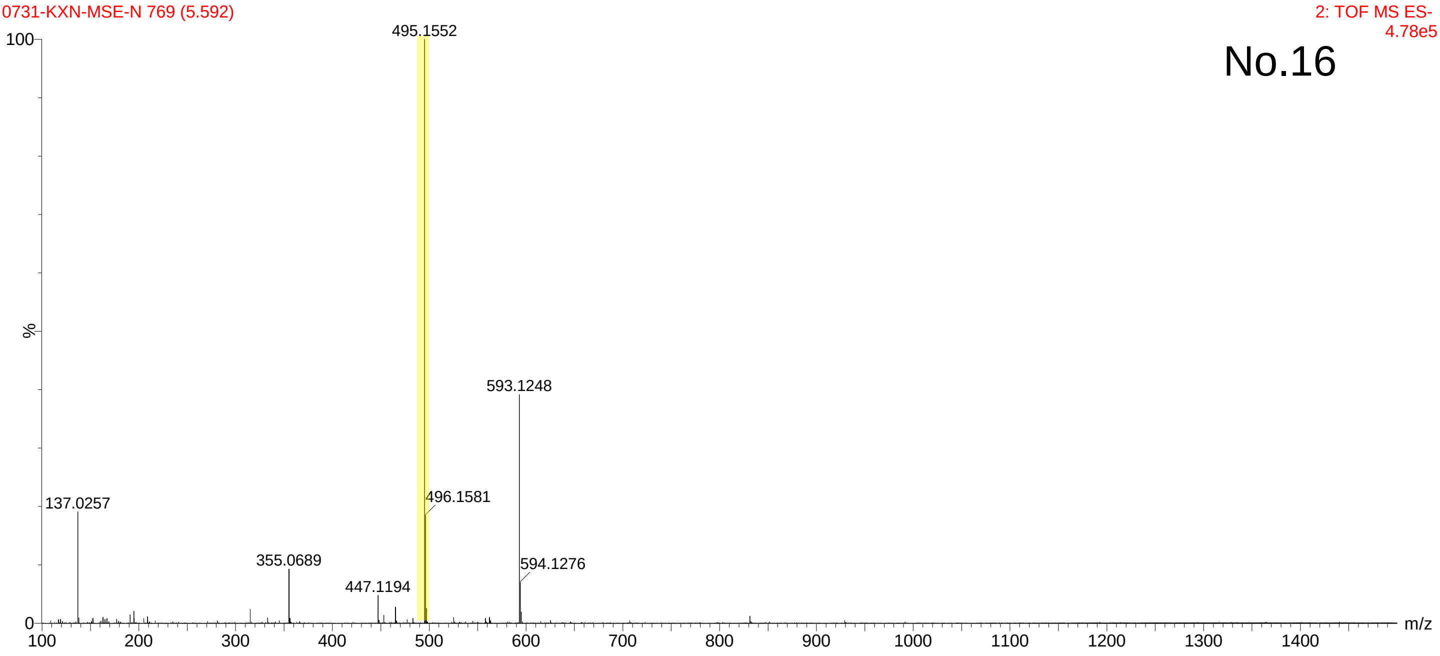
0731-KXN-MSE-N 769 (5.592)
2: TOF MS ES-
4.78e5
No.16
495.1552
593.1248
137.0257
496.1581
355.0689
447.1194
594.1276
100
%
0
100 200 300 400 500 600 700 800 900 1000 1100 1200 1300 1400
m/z

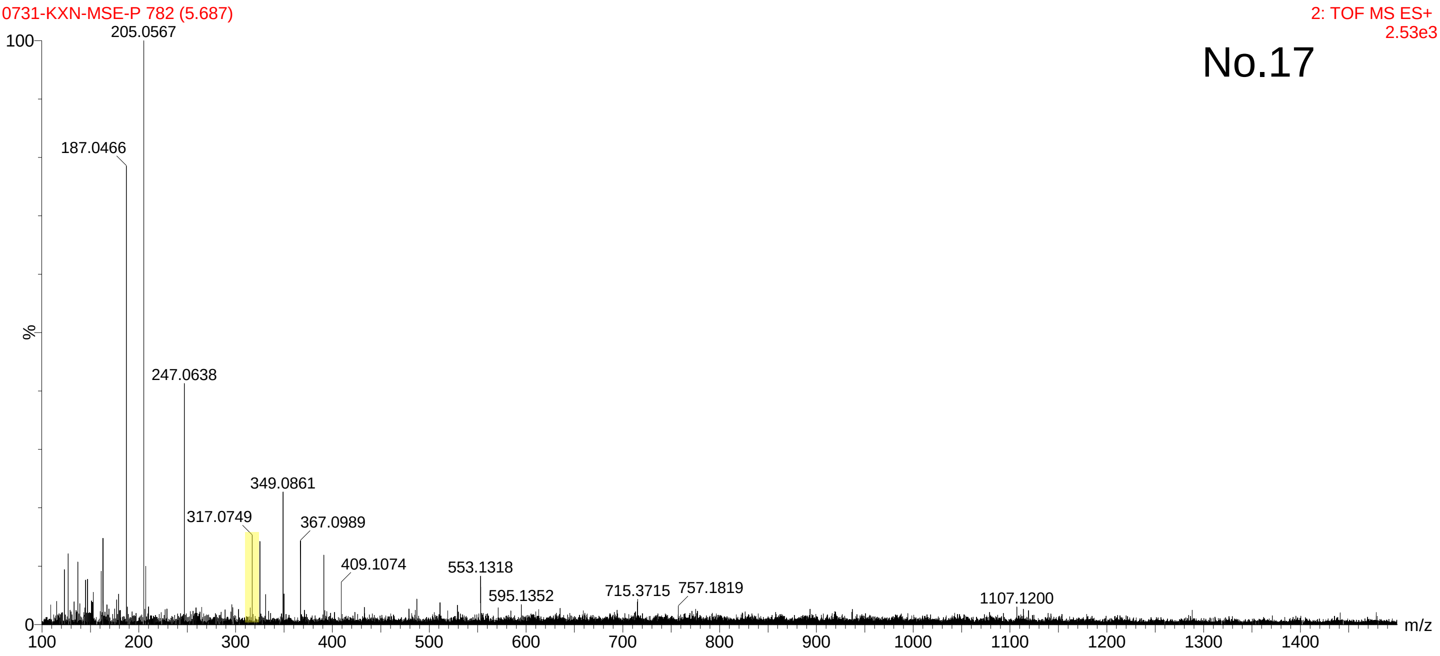
0731-KXN-MSE-P 782 (5.687)
2: TOF MS ES+
2.53e3
No.17
205.0567
187.0466
247.0638
349.0861
317.0749
367.0989
409.1074
553.1318
595.1352
715.3715
757.1819
1107.1200
100
%
0
100 200 300 400 500 600 700 800 900 1000 1100 1200 1300 1400
m/z

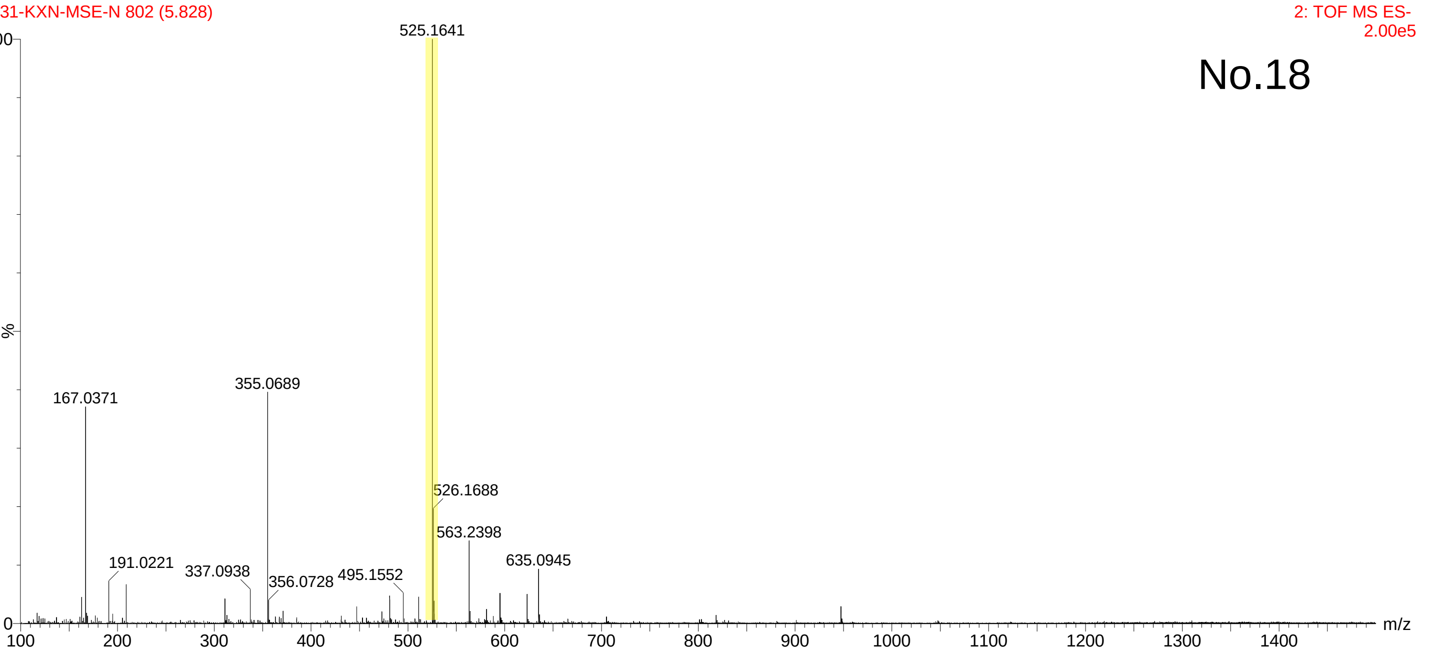
31-KXN-MSE-N 802 (5.828)
2: TOF MS ES-
2.00e5
No.18
525.1641
167.0371
355.0689
526.1688
563.2398
191.0221
337.0938
356.0728
495.1552
635.0945
%
0
100 200 300 400 500 600 700 800 900 1000 1100 1200 1300 1400
m/z

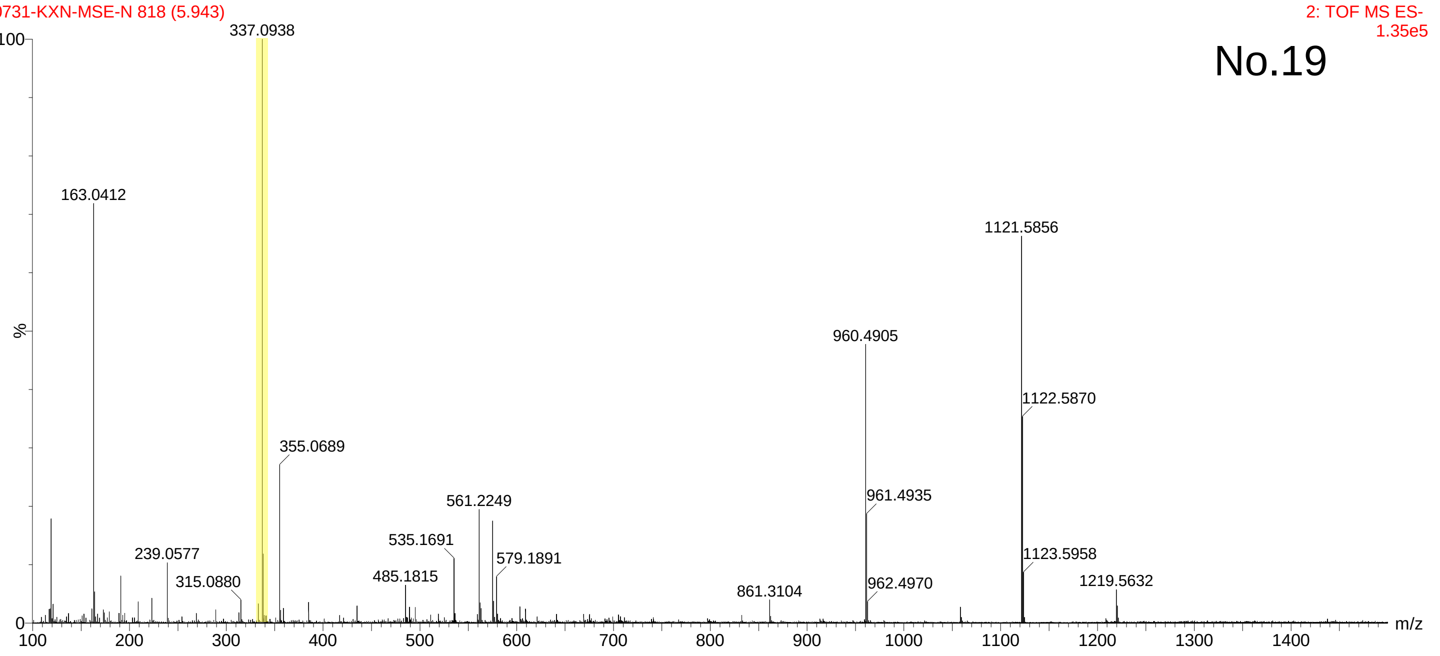
0731-KXN-MSE-N 818 (5.943)
2: TOF MS ES-
1.35e5
No.19
337.0938
163.0412
1121.5856
960.4905
1122.5870
355.0689
561.2249
961.4935
535.1691
239.0577
579.1891
1123.5958
315.0880
485.1815
1219.5632
861.3104
962.4970
%
m/z

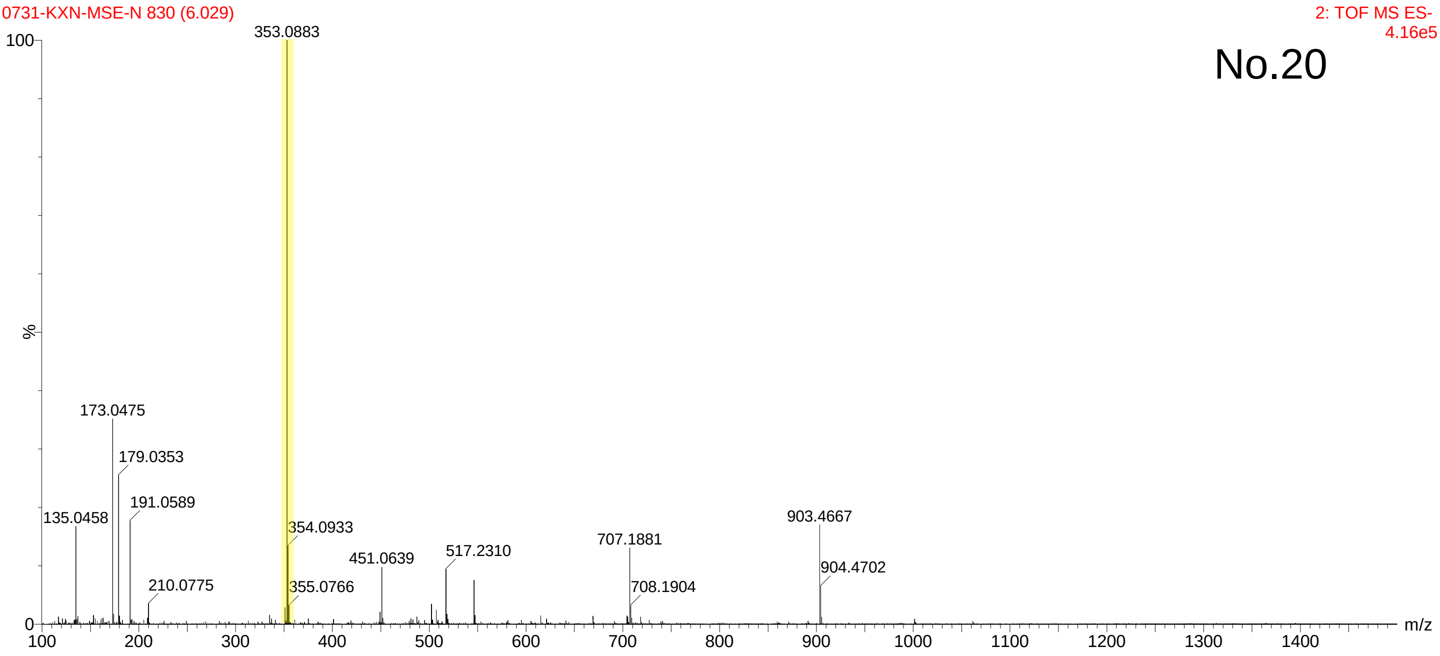
0731-KXN-MSE-N 830 (6.029)
2: TOF MS ES-
4.16e5
No.20
353.0883
173.0475
179.0353
191.0589
135.0458
354.0933
903.4667
451.0639
517.2310
707.1881
904.4702
210.0775
355.0766
708.1904
%
m/z

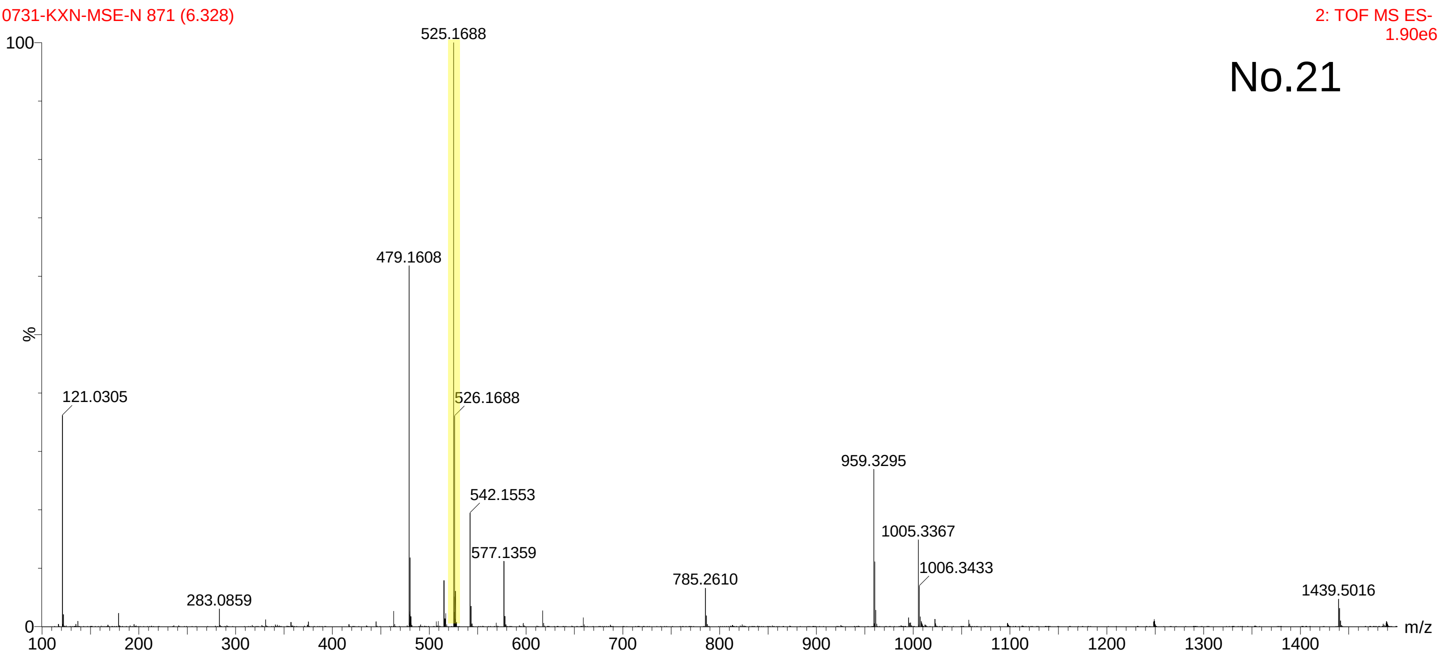
0731-KXN-MSE-N 871 (6.328)
2: TOF MS ES-
1.90e6
No.21
525.1688
479.1608
121.0305
526.1688
959.3295
542.1553
1005.3367
577.1359
1006.3433
785.2610
1439.5016
283.0859
%
m/z

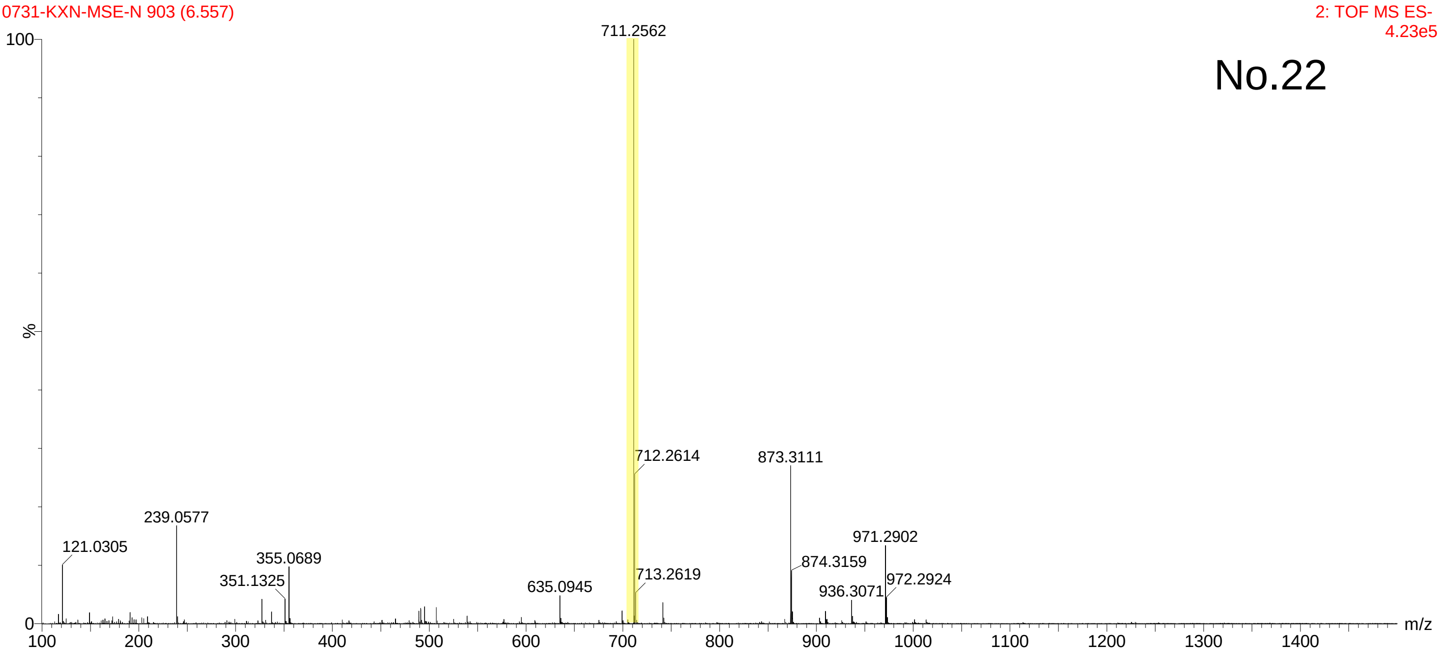
0731-KXN-MSE-N 903 (6.557)
2: TOF MS ES-
4.23e5
No.22
711.2562
712.2614
873.3111
971.2902
874.3159
972.2924
936.3071
713.2619
239.0577
121.0305
355.0689
351.1325
635.0945
%
100
0
m/z
100 200 300 400 500 600 700 800 900 1000 1100 1200 1300 1400

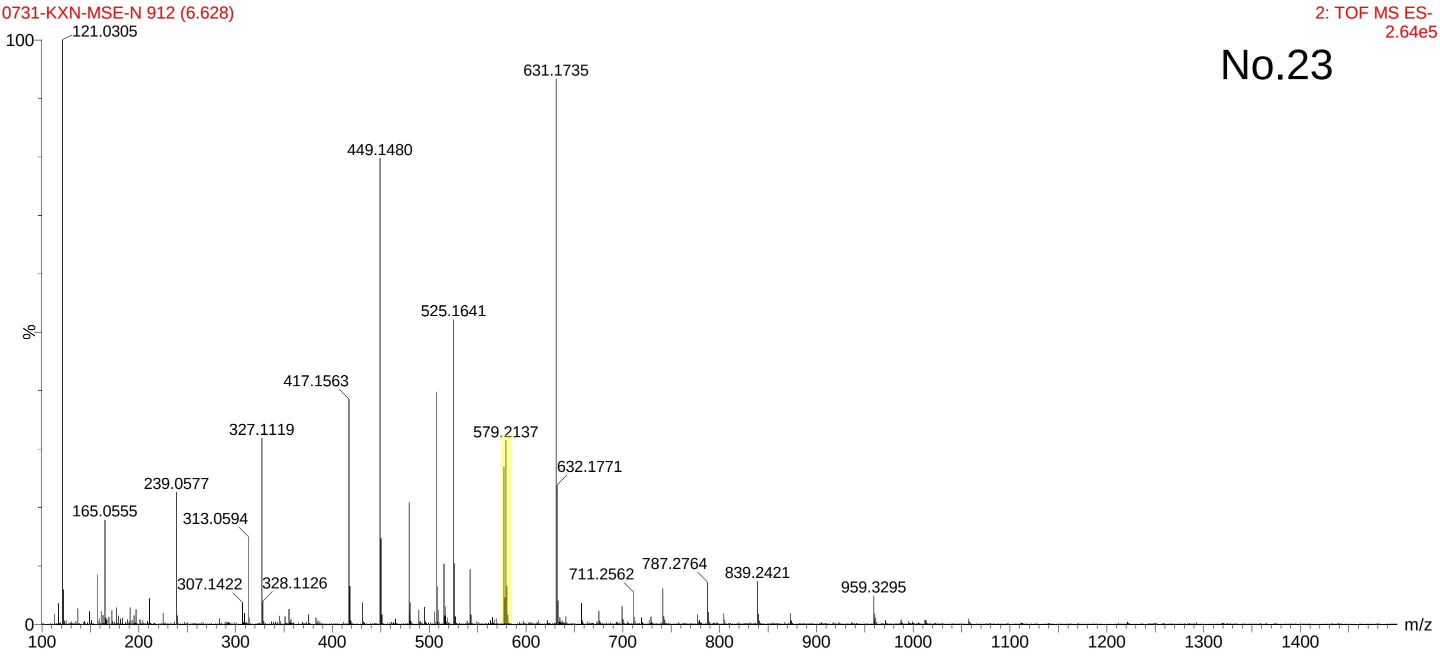
0731-KXN-MSE-N 912 (6.628)
2: TOF MS ES-
2.64e5
No.23
121.0305
631.1735
449.1480
525.1641
417.1563
327.1119
579.2137
632.1771
239.0577
165.0555
313.0594
307.1422
328.1126
711.2562
787.2764
839.2421
959.3295
%
100
0
m/z
100 200 300 400 500 600 700 800 900 1000 1100 1200 1300 1400

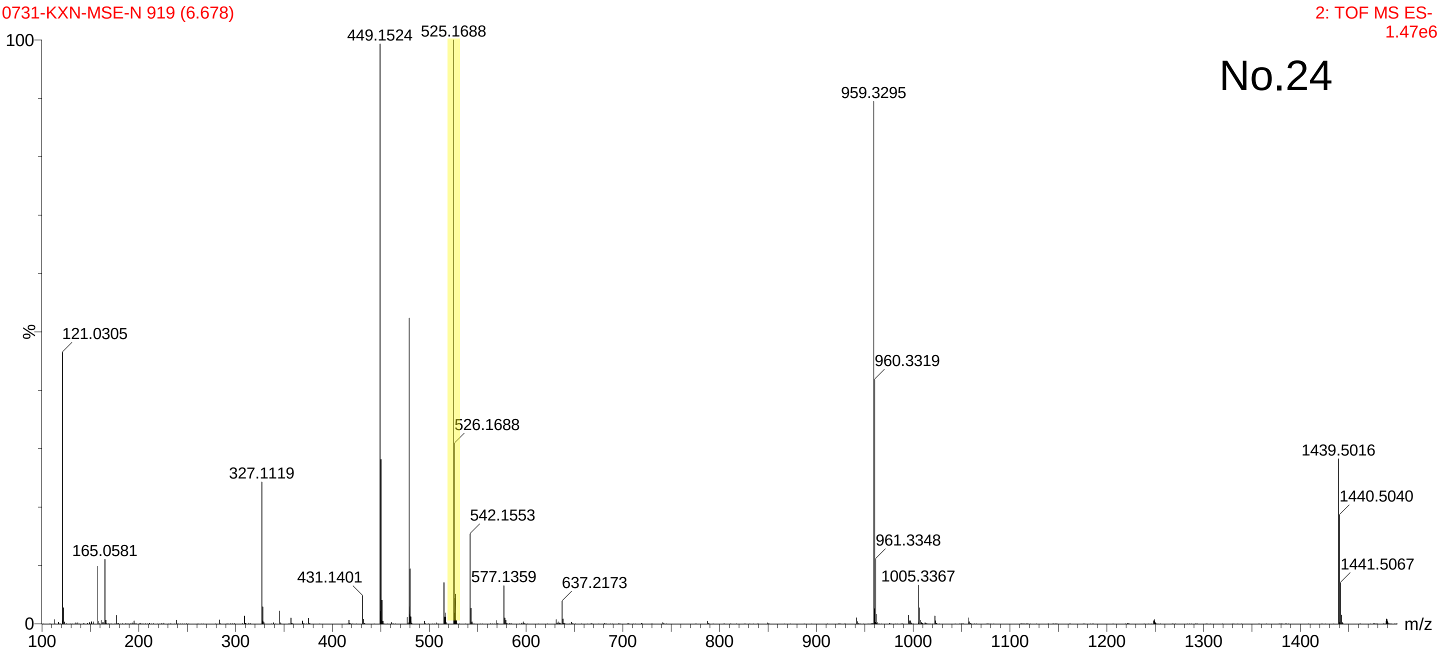
0731-KXN-MSE-N 919 (6.678)
2: TOF MS ES-
1.47e6
No.24
449.1524
525.1688
959.3295
121.0305
960.3319
526.1688
1439.5016
327.1119
1440.5040
542.1553
961.3348
165.0581
1441.5067
431.1401
577.1359
1005.3367
637.2173
%
100
0
m/z
100 200 300 400 500 600 700 800 900 1000 1100 1200 1300 1400

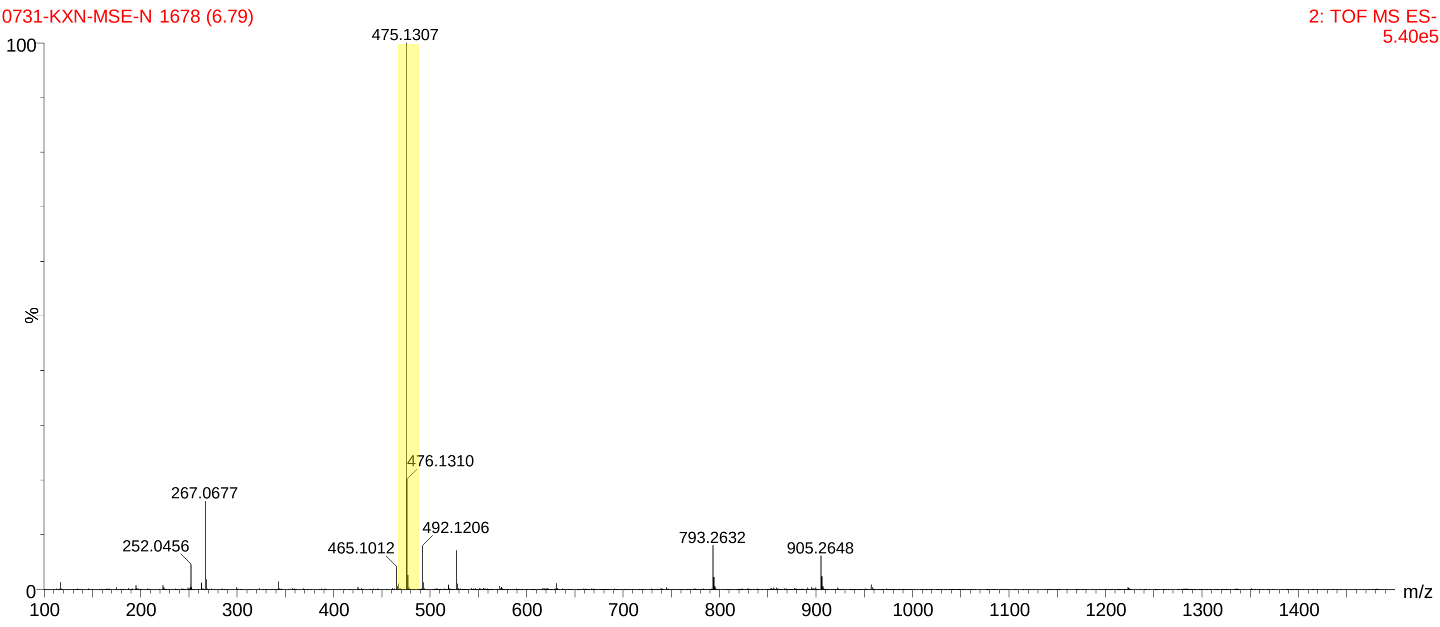
0731-KXN-MSE-N 1678 (6.79)
2: TOF MS ES-
5.40e5
475.1307
476.1310
267.0677
252.0456
465.1012
492.1206
793.2632
905.2648
%
m/z

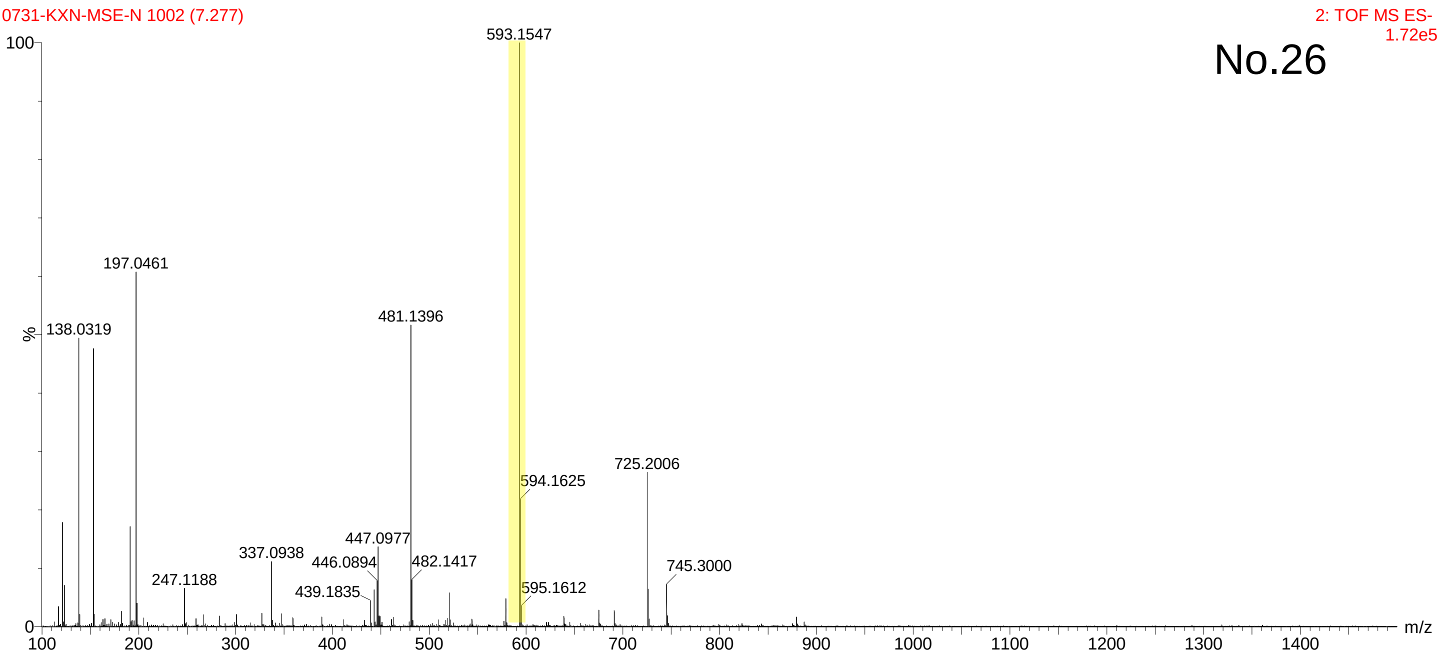
0731-KXN-MSE-N 1002 (7.277)
2: TOF MS ES-
1.72e5
593.1547
197.0461
138.0319
481.1396
594.1625
725.2006
447.0977
337.0938
446.0894
482.1417
745.3000
247.1188
439.1835
595.1612
%
m/z

No.26

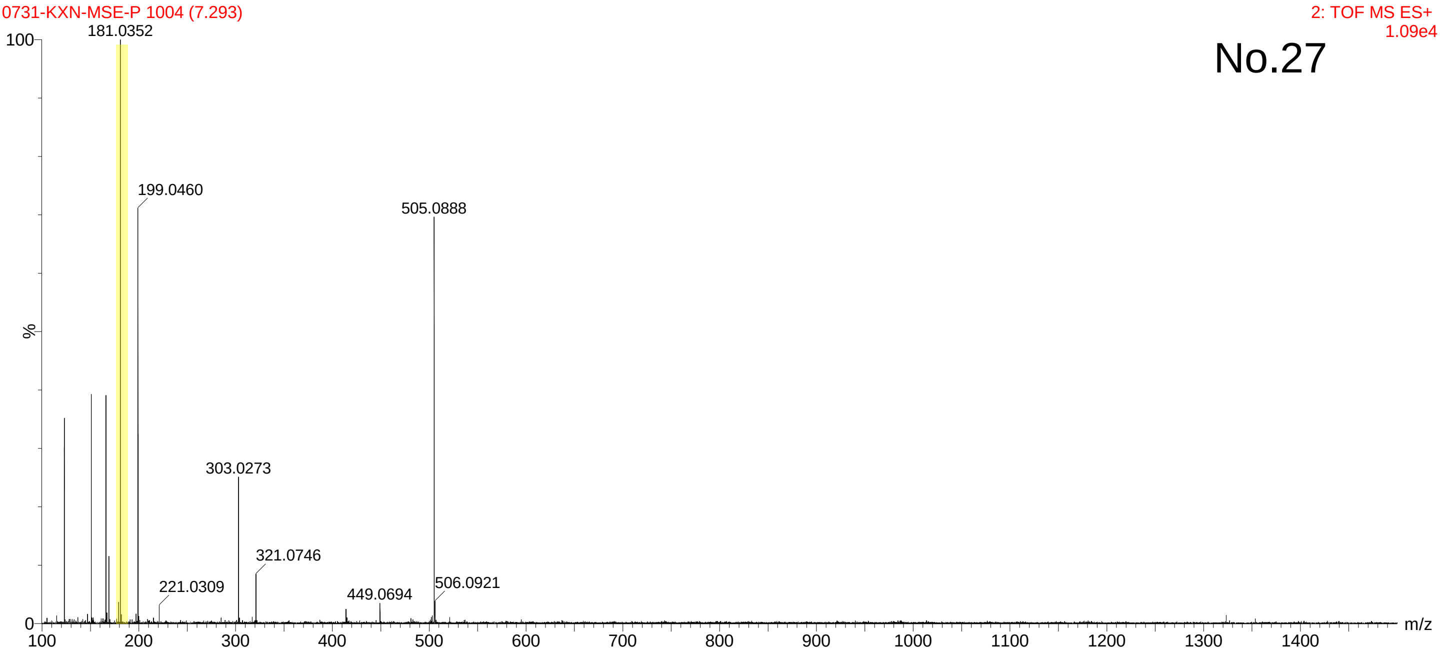
0731-KXN-MSE-P 1004 (7.293)
2: TOF MS ES+
1.09e4
181.0352
199.0460
505.0888
303.0273
321.0746
221.0309
449.0694
506.0921
%
m/z

No.27

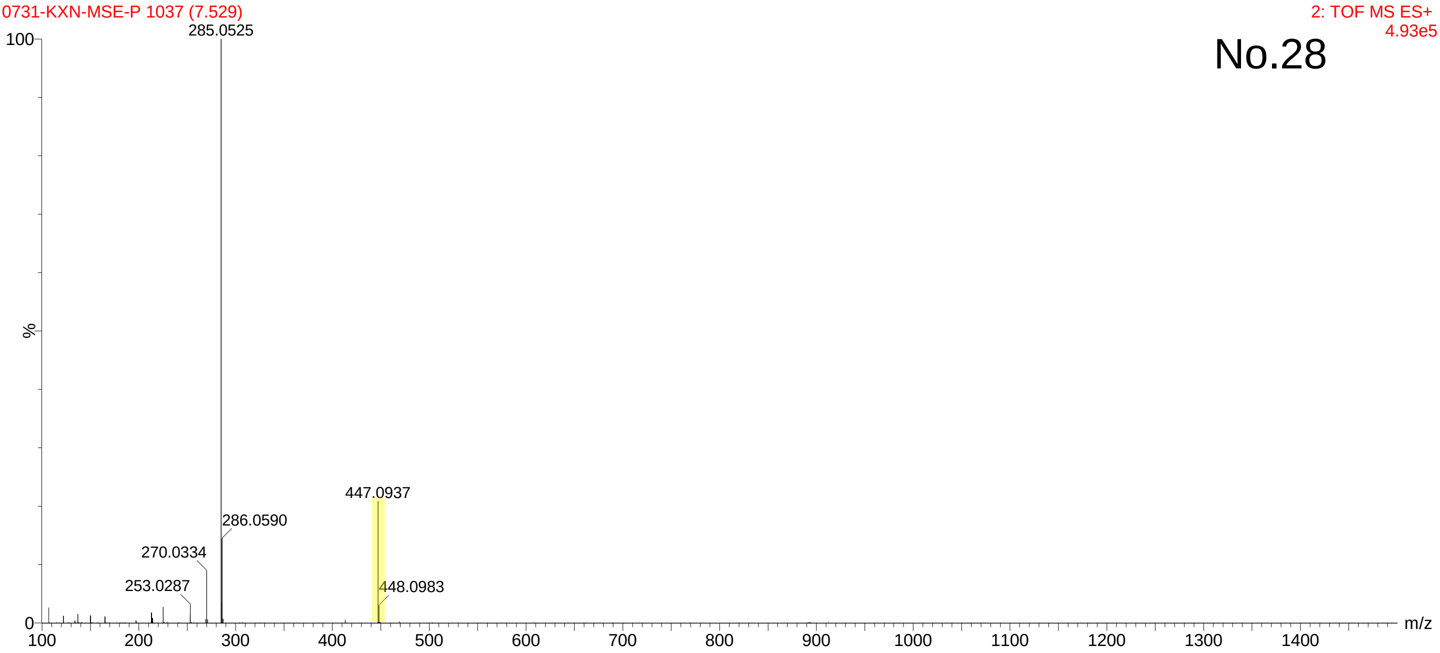
0731-KXN-MSE-P 1037 (7.529)
2: TOF MS ES+
4.93e5
No.28
285.0525
447.0937
286.0590
270.0334
253.0287
448.0983
%
m/z

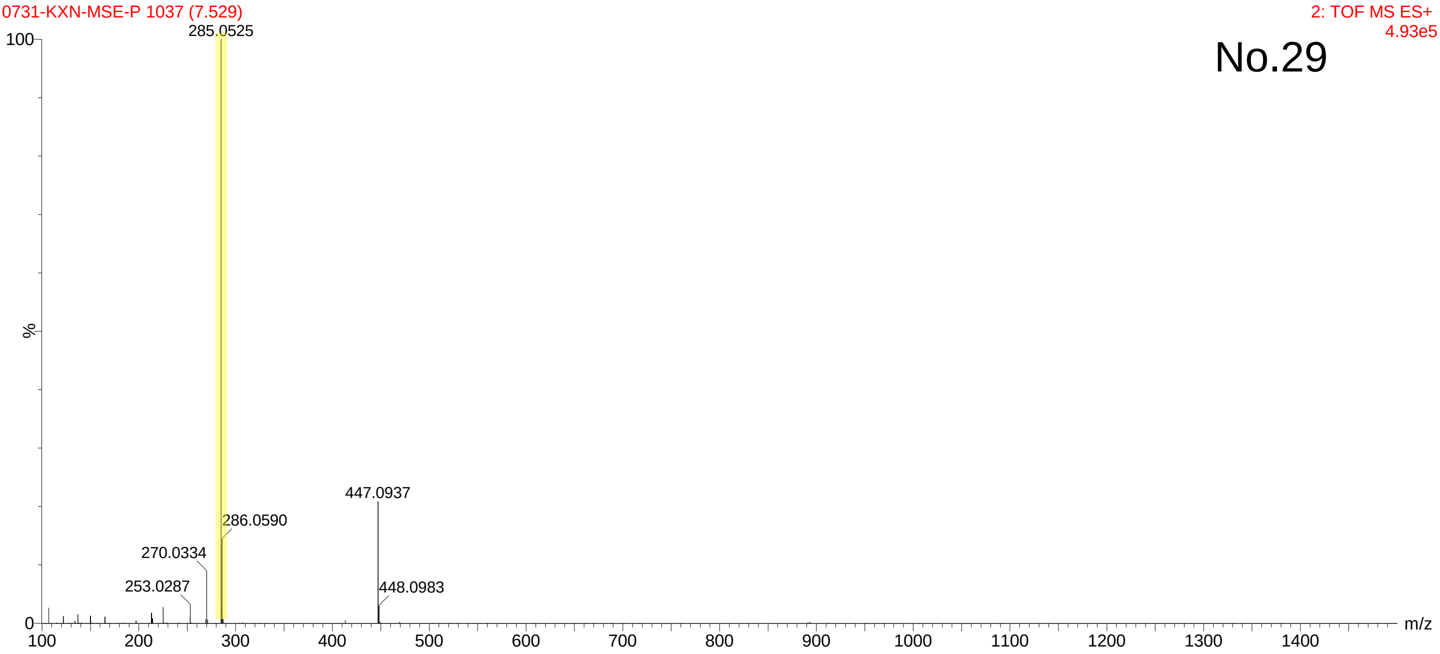
0731-KXN-MSE-P 1037 (7.529)
2: TOF MS ES+
4.93e5
No.29
285.0525
447.0937
286.0590
270.0334
253.0287
448.0983
%
m/z

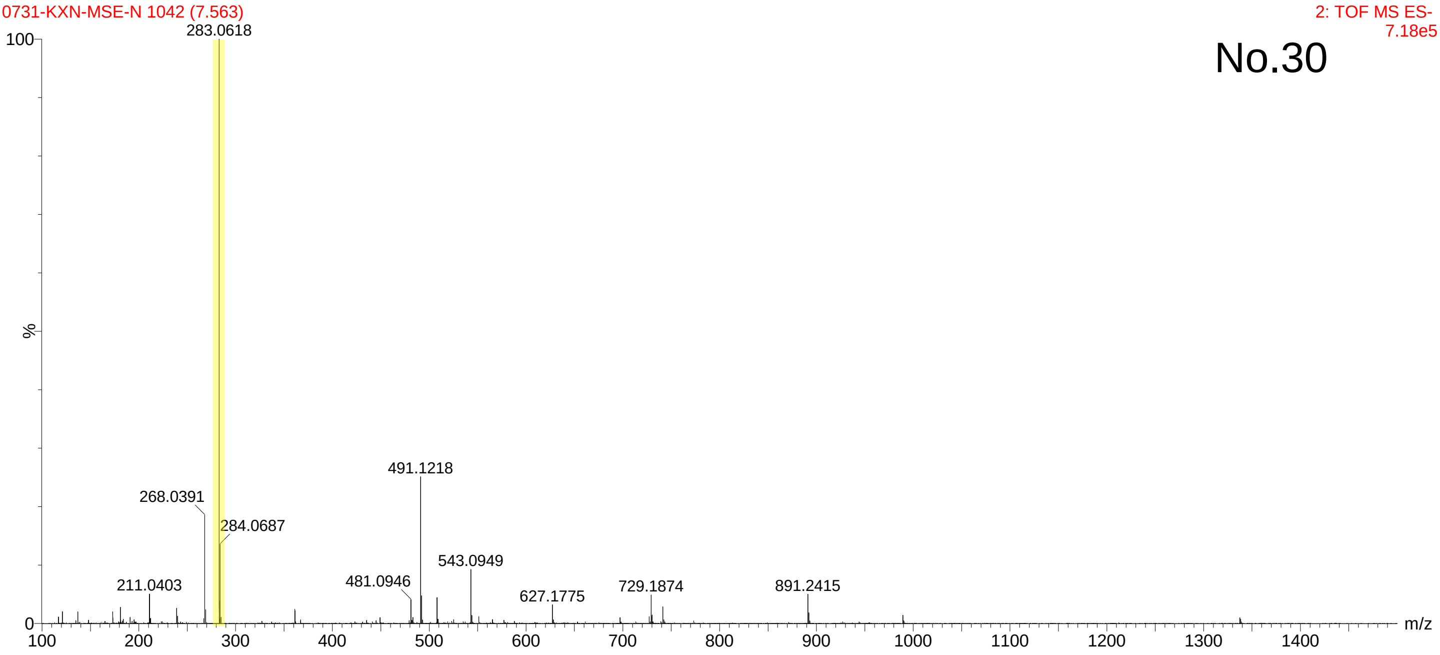
0731-KXN-MSE-N 1042 (7.563)
2: TOF MS ES-
7.18e5
No.30
283.0618
491.1218
268.0391
284.0687
543.0949
481.0946
211.0403
627.1775
729.1874
891.2415
%
m/z

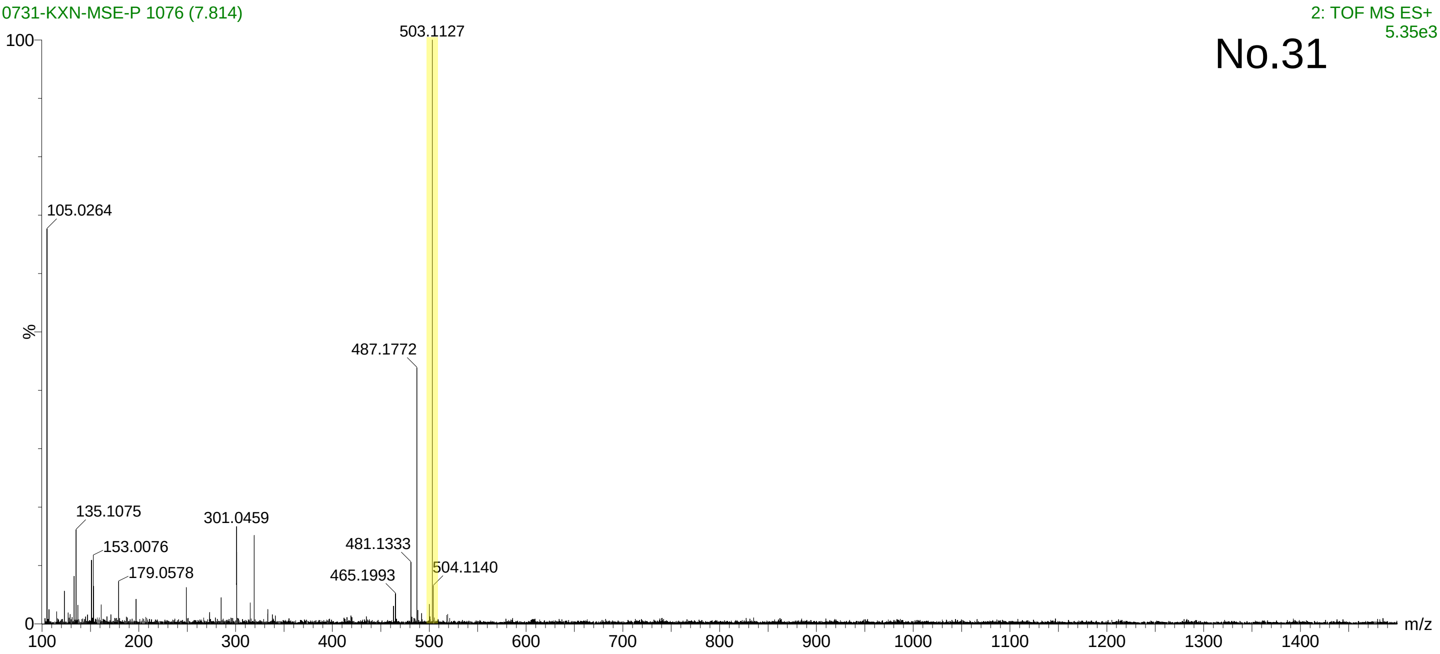
0731-KXN-MSE-P 1076 (7.814)
2: TOF MS ES+
5.35e3
No.31
503.1127
105.0264
487.1772
135.1075
301.0459
153.0076
481.1333
179.0578
504.1140
465.1993
%
m/z

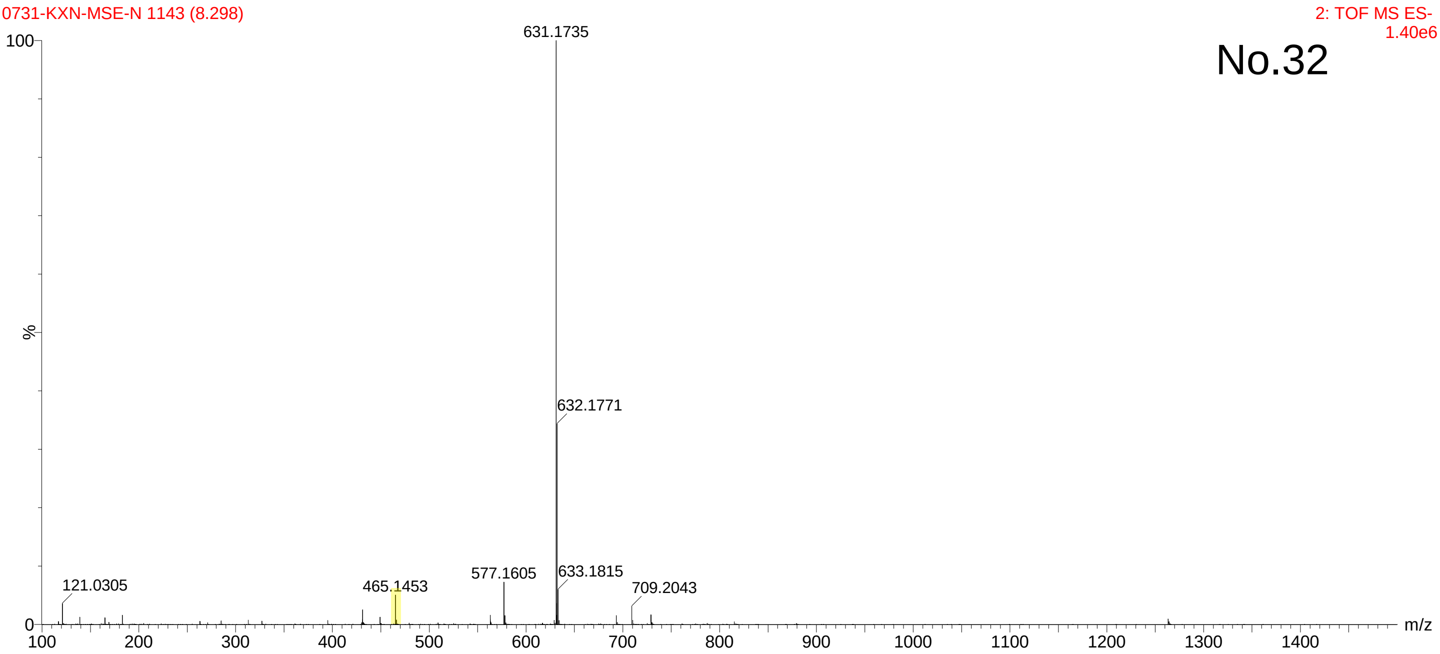
0731-KXN-MSE-N 1143 (8.298)
2: TOF MS ES-
1.40e6
No.32
631.1735
632.1771
121.0305
465.1453
577.1605
633.1815
709.2043
%
m/z

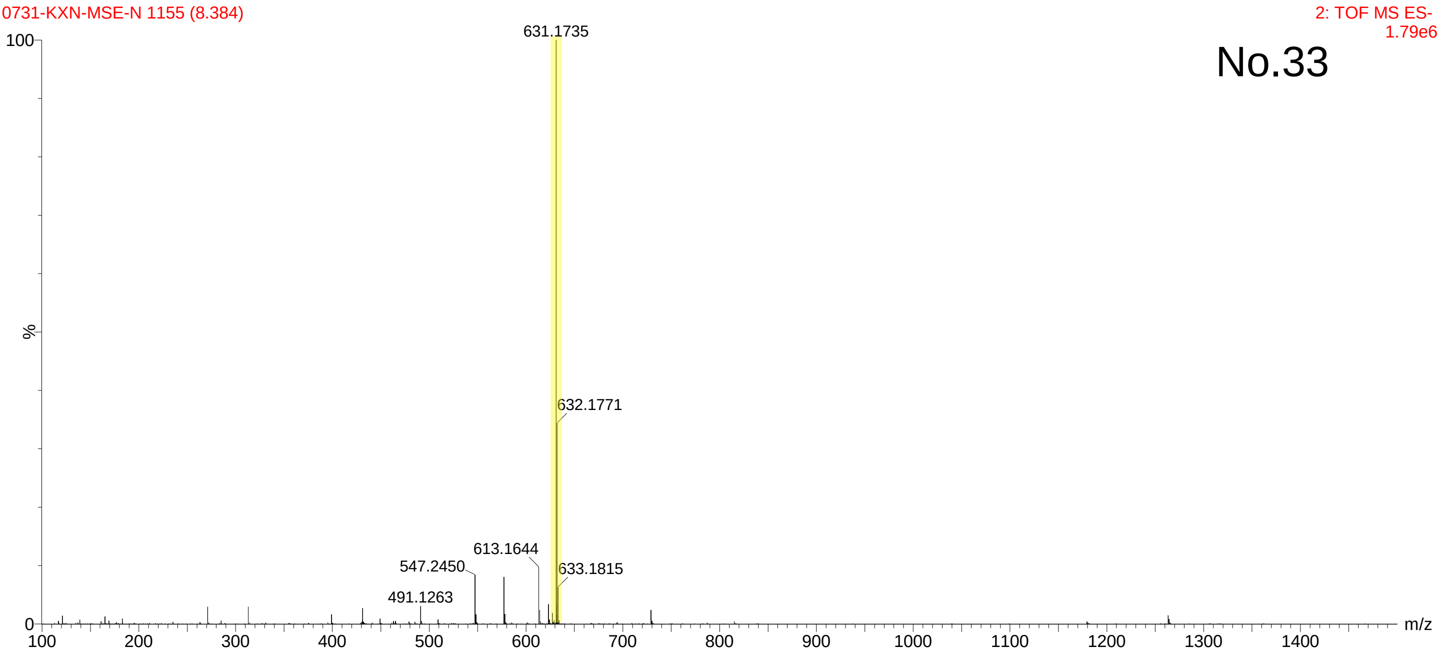
0731-KXN-MSE-N 1155 (8.384)
2: TOF MS ES-
1.79e6
No.33
631.1735
632.1771
613.1644
547.2450
633.1815
491.1263
%
m/z

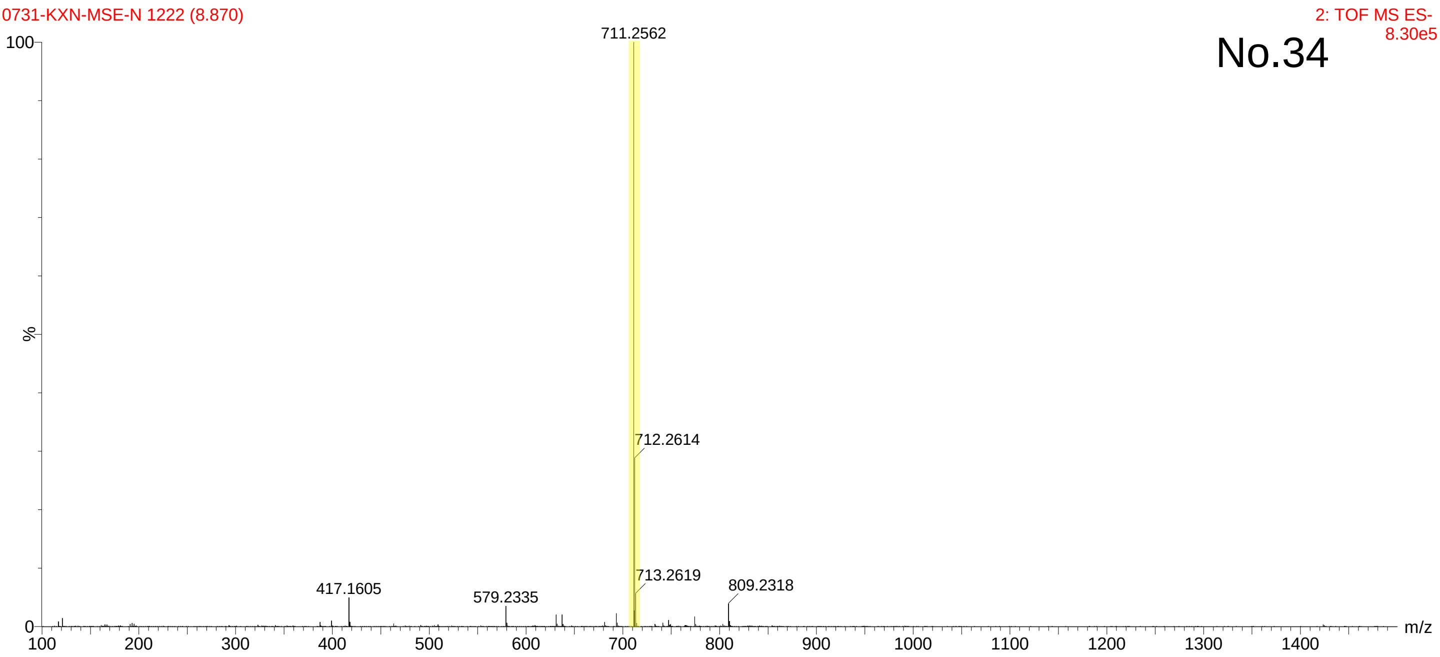
0731-KXN-MSE-N 1222 (8.870)
2: TOF MS ES-
8.30e5
No.34
711.2562
712.2614
713.2619
809.2318
417.1605
579.2335
100
%
0
m/z
100 200 300 400 500 600 700 800 900 1000 1100 1200 1300 1400

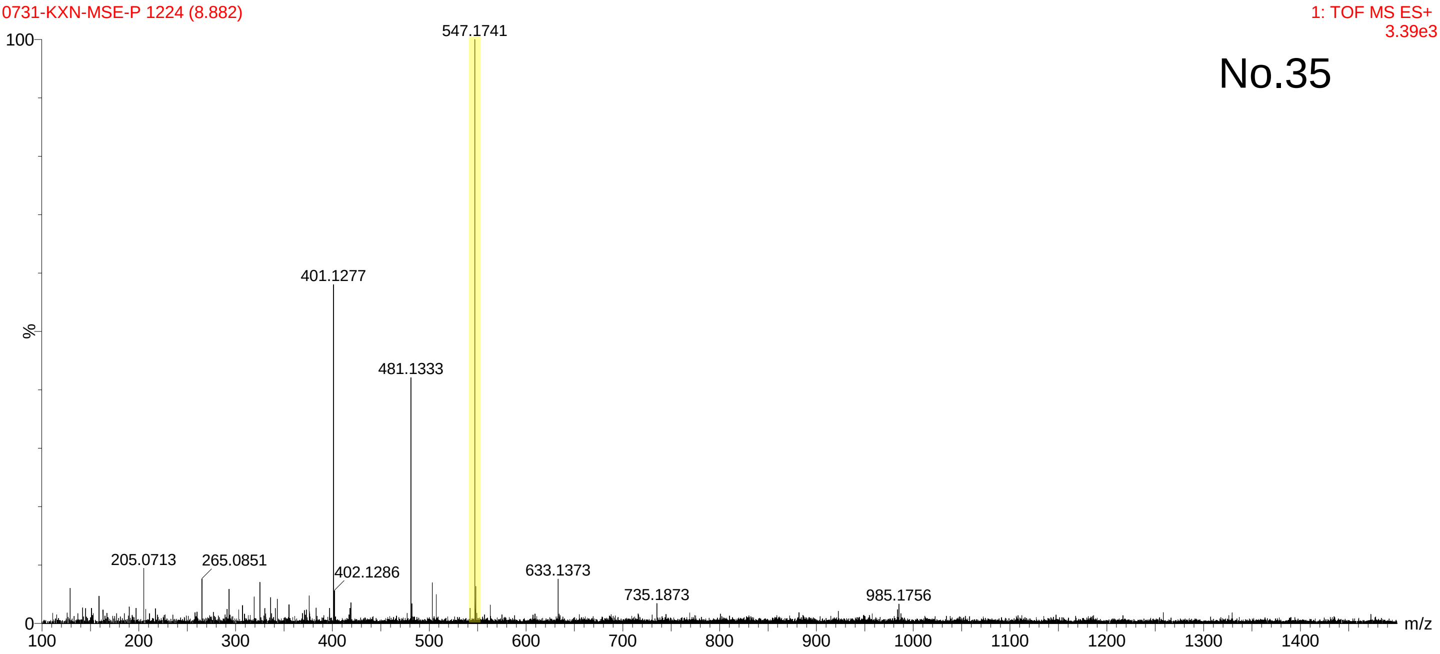
0731-KXN-MSE-P 1224 (8.882)
1: TOF MS ES+
3.39e3
No.35
547.1741
401.1277
481.1333
205.0713
265.0851
402.1286
633.1373
735.1873
985.1756
100
%
0
m/z
100 200 300 400 500 600 700 800 900 1000 1100 1200 1300 1400

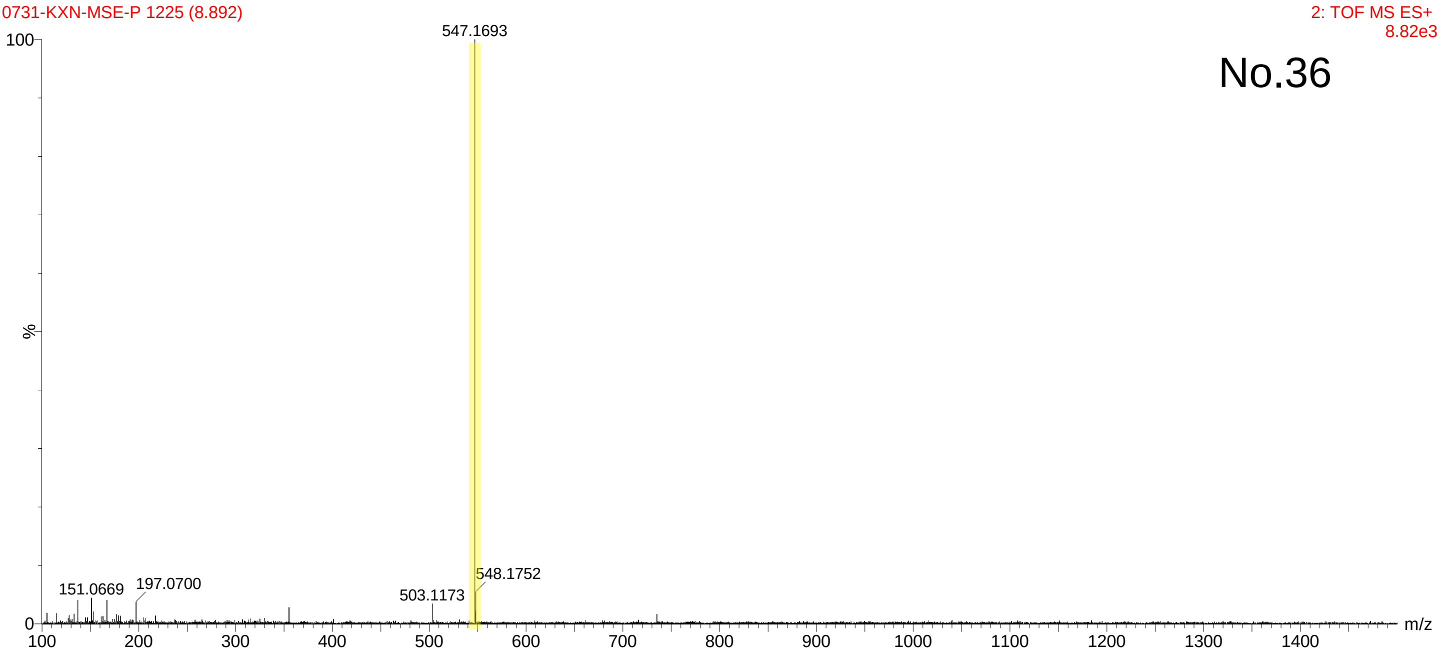
0731-KXN-MSE-P 1225 (8.892)
2: TOF MS ES+
8.82e3
No.36
547.1693
548.1752
151.0669
197.0700
503.1173
100
%
0
m/z
100 200 300 400 500 600 700 800 900 1000 1100 1200 1300 1400

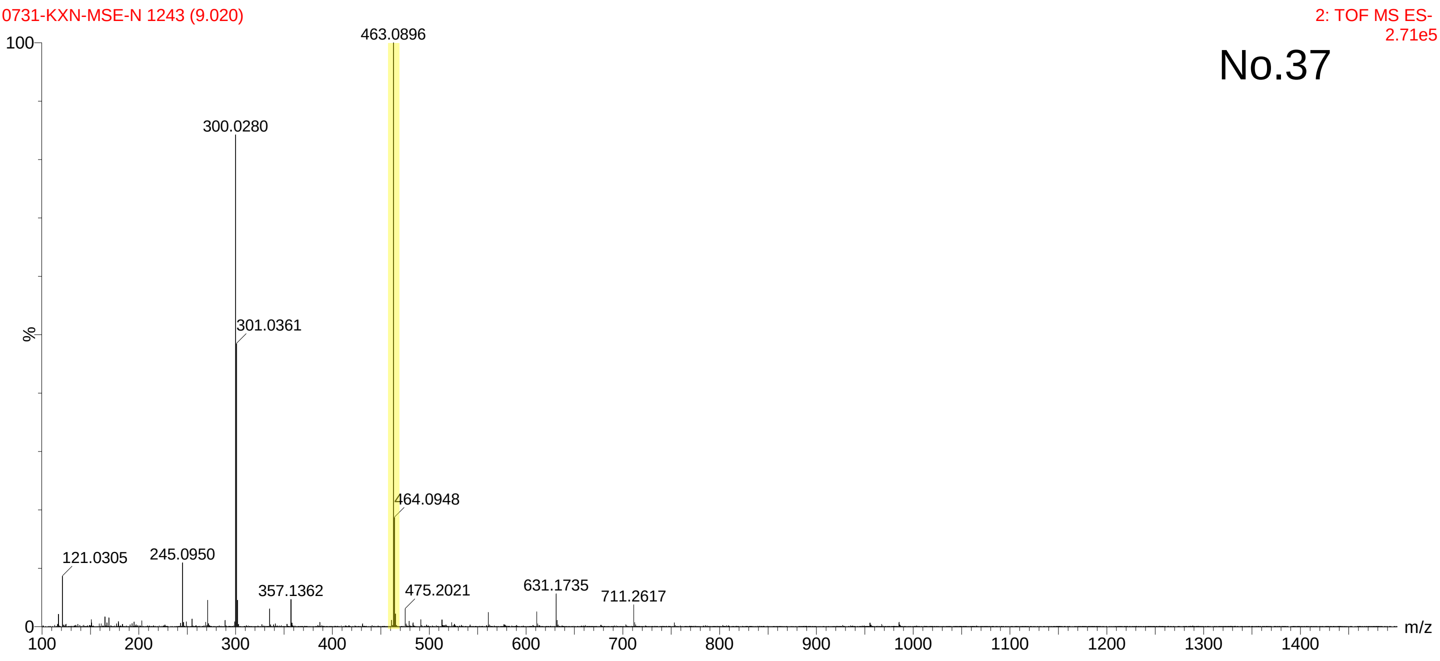
0731-KXN-MSE-N 1243 (9.020)
2: TOF MS ES-
2.71e5
No.37
463.0896
300.0280
301.0361
464.0948
121.0305
245.0950
357.1362
475.2021
631.1735
711.2617
100
%
0
m/z
100 200 300 400 500 600 700 800 900 1000 1100 1200 1300 1400

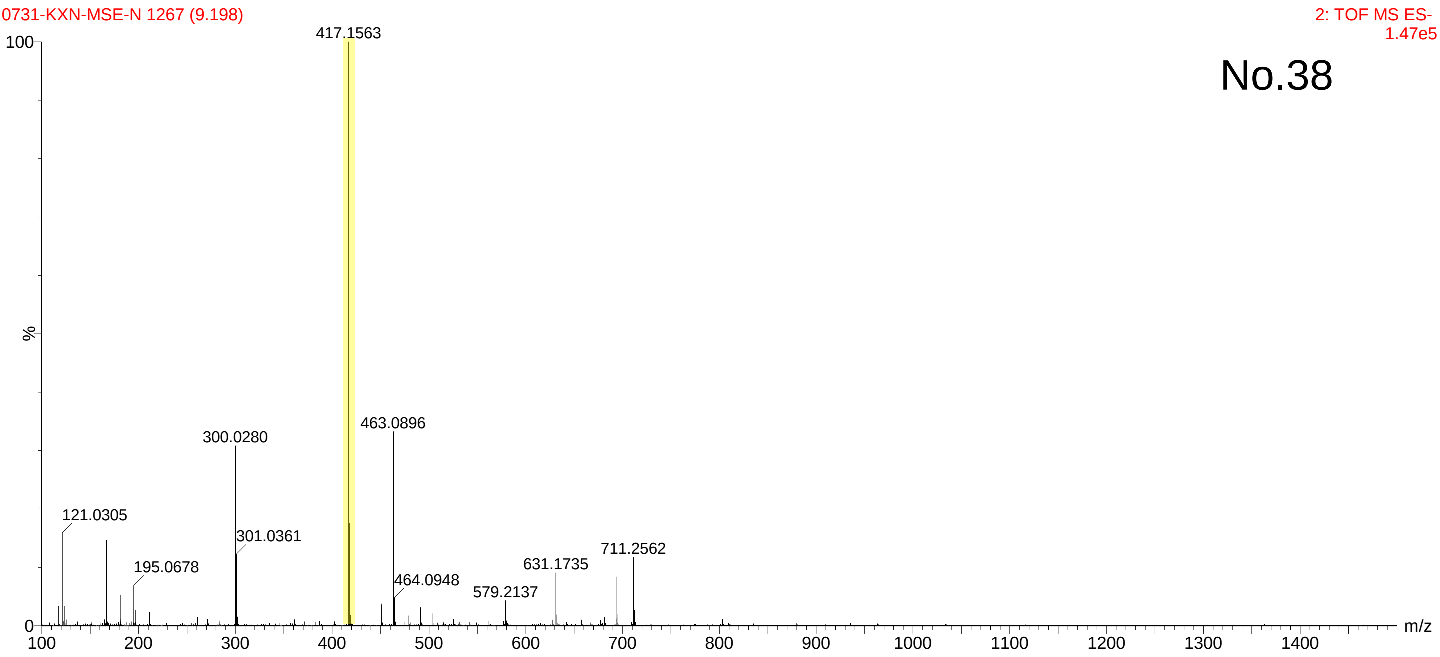
0731-KXN-MSE-N 1267 (9.198)
2: TOF MS ES-
1.47e5
No.38
417.1563
463.0896
300.0280
121.0305
301.0361
195.0678
711.2562
631.1735
464.0948
579.2137
100
%
0
m/z
100 200 300 400 500 600 700 800 900 1000 1100 1200 1300 1400

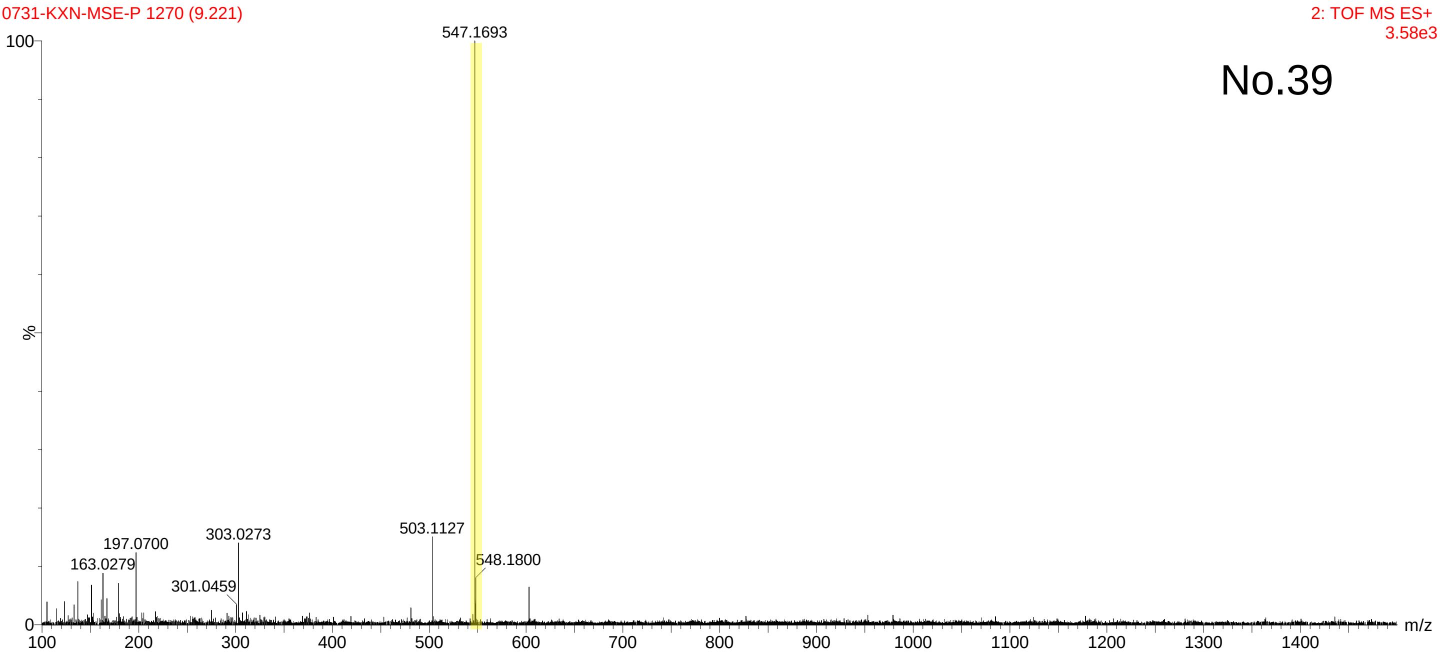
0731-KXN-MSE-P 1270 (9.221)
2: TOF MS ES+
3.58e3
No.39
547.1693
503.1127
303.0273
197.0700
163.0279
548.1800
301.0459
100
%
0
m/z
100 200 300 400 500 600 700 800 900 1000 1100 1200 1300 1400

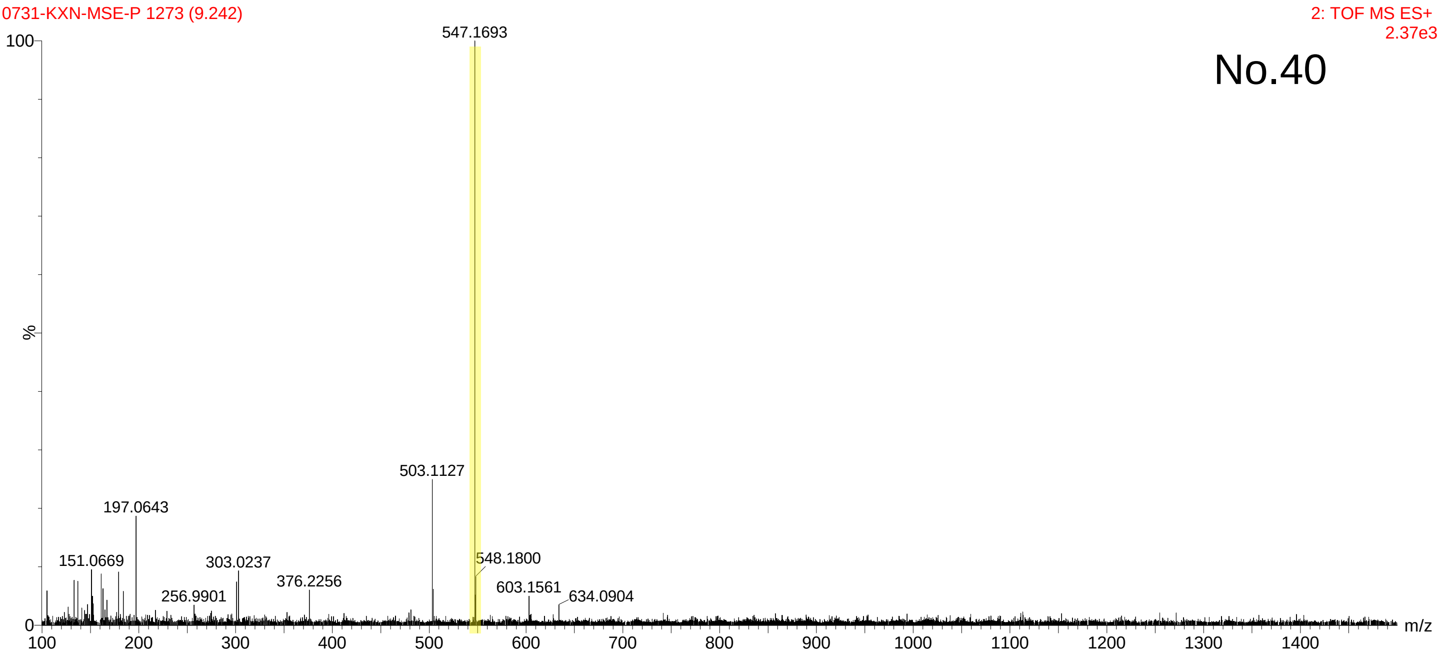
0731-KXN-MSE-P 1273 (9.242)
2: TOF MS ES+
2.37e3
No.40
547.1693
503.1127
197.0643
151.0669
303.0237
376.2256
256.9901
548.1800
603.1561
634.0904
100
%
0
m/z
100 200 300 400 500 600 700 800 900 1000 1100 1200 1300 1400

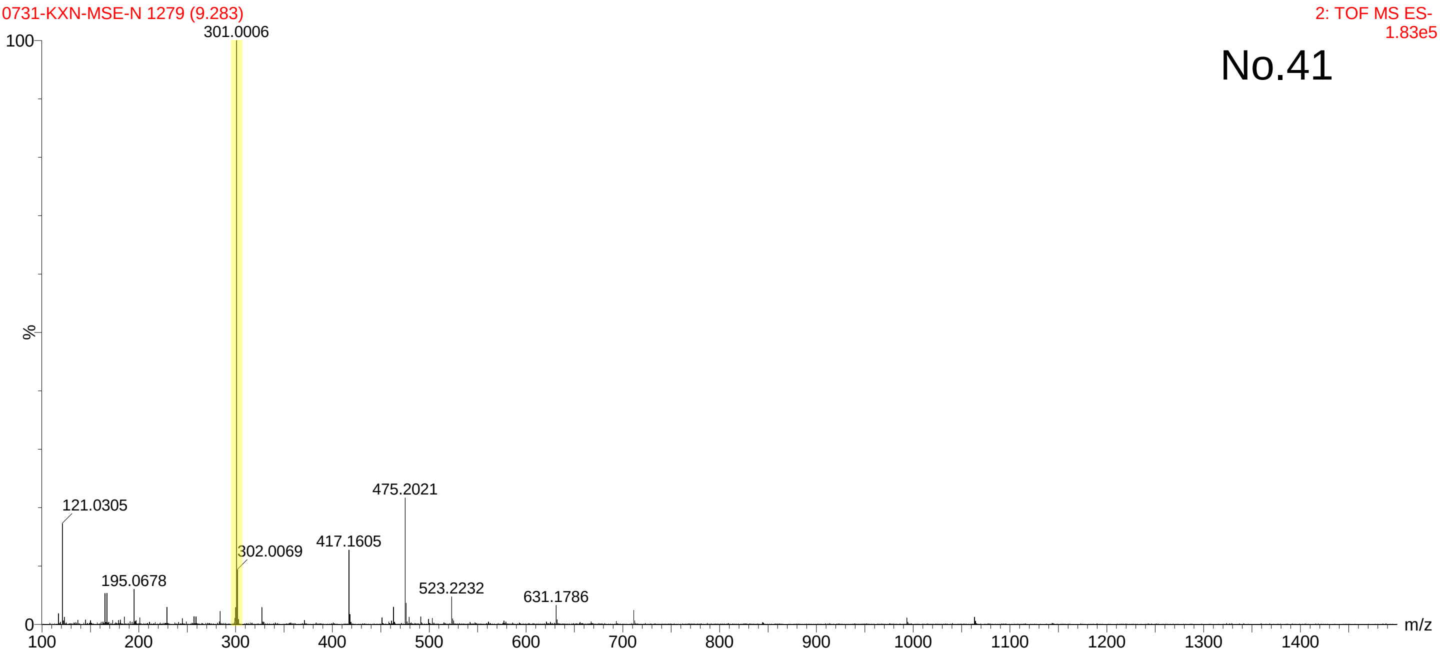
0731-KXN-MSE-N 1279 (9.283)
2: TOF MS ES-
1.83e5
No.41
301.0006
121.0305
475.2021
417.1605
302.0069
195.0678
523.2232
631.1786
100
%
0
m/z
100 200 300 400 500 600 700 800 900 1000 1100 1200 1300 1400

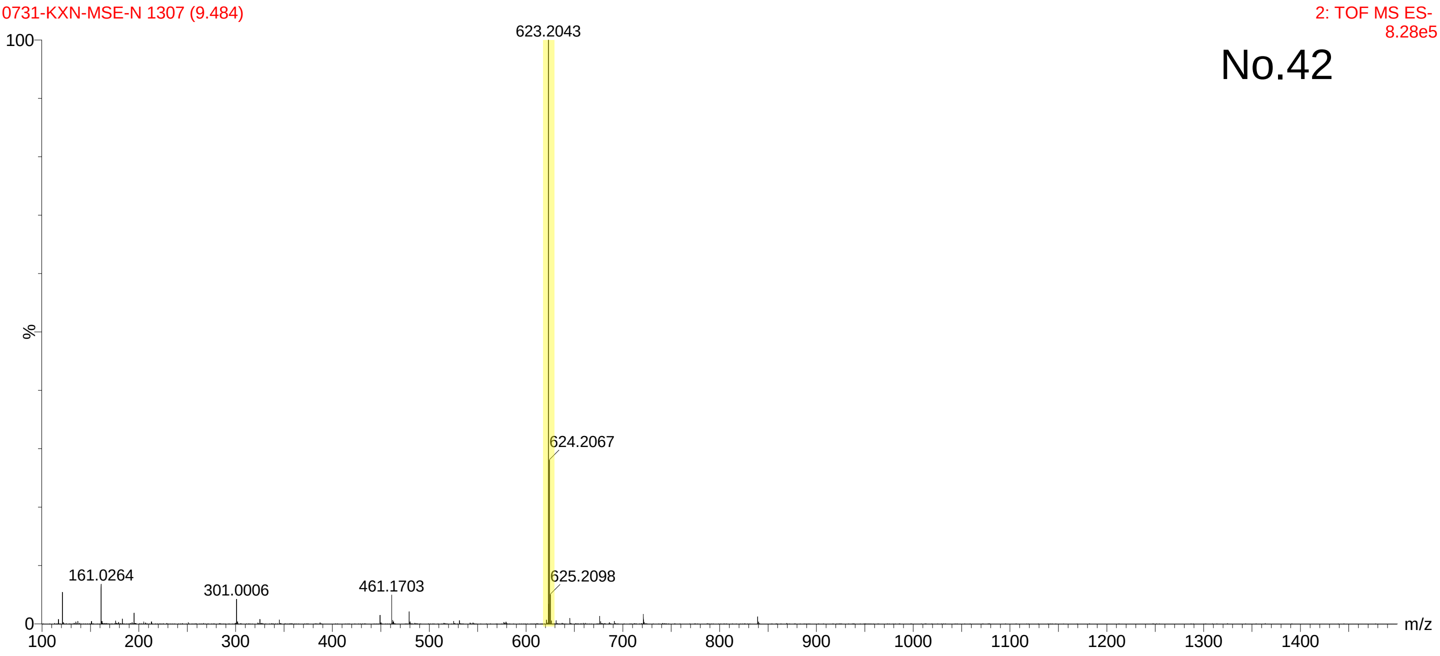
0731-KXN-MSE-N 1307 (9.484)
2: TOF MS ES-
8.28e5
No.42
623.2043
624.2067
161.0264
301.0006
461.1703
625.2098
100
%
0
m/z
100 200 300 400 500 600 700 800 900 1000 1100 1200 1300 1400

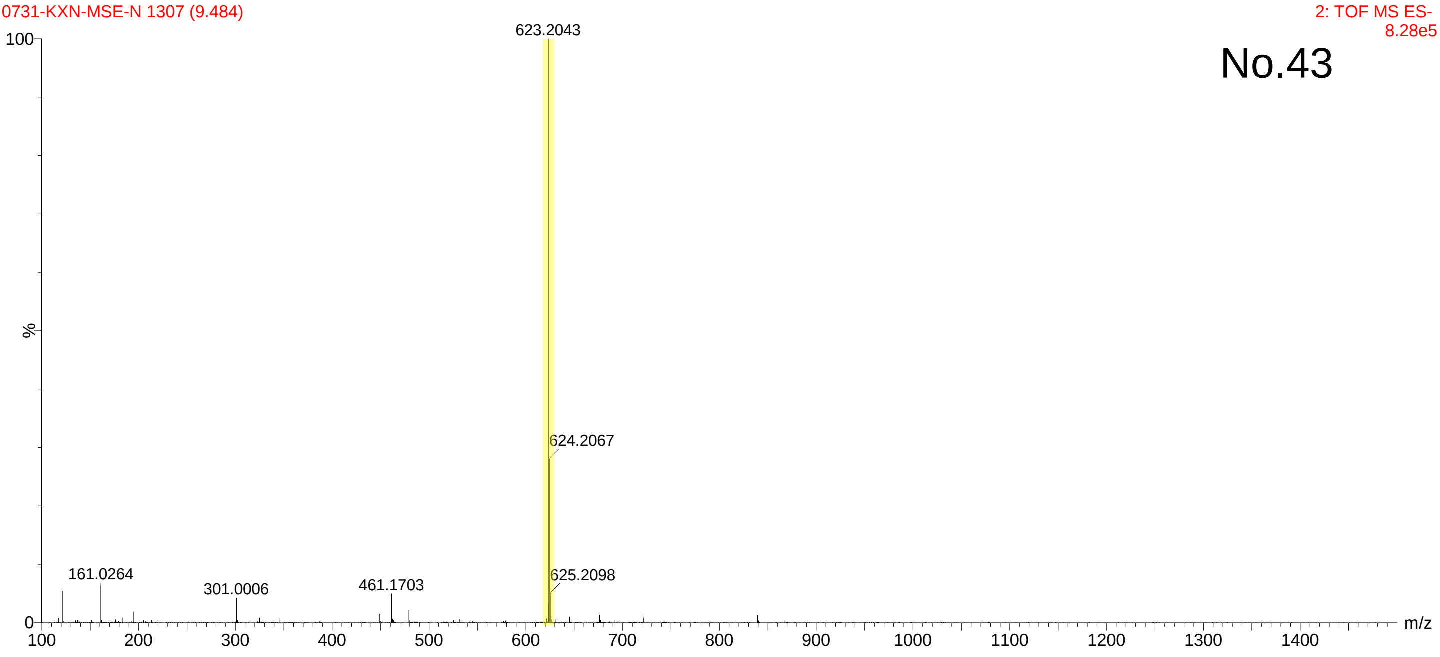

0731-KXN-MSE-N 1307 (9.484)
2: TOF MS ES-
8.28e5
No.43
623.2043
624.2067
625.2098
161.0264
301.0006
461.1703
100
%
0
m/z
100 200 300 400 500 600 700 800 900 1000 1100 1200 1300 1400

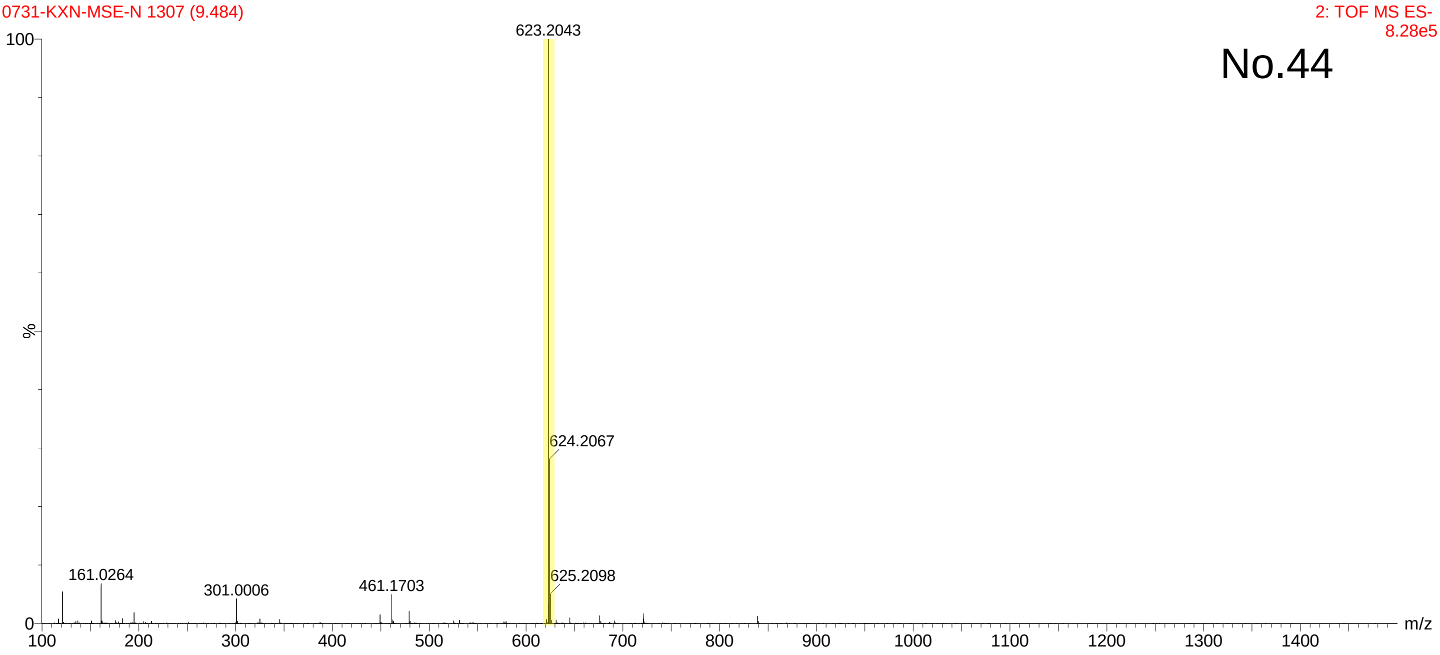

0731-KXN-MSE-N 1307 (9.484)
2: TOF MS ES-
8.28e5
No.44
623.2043
624.2067
625.2098
161.0264
301.0006
461.1703
100
%
0
m/z
100 200 300 400 500 600 700 800 900 1000 1100 1200 1300 1400

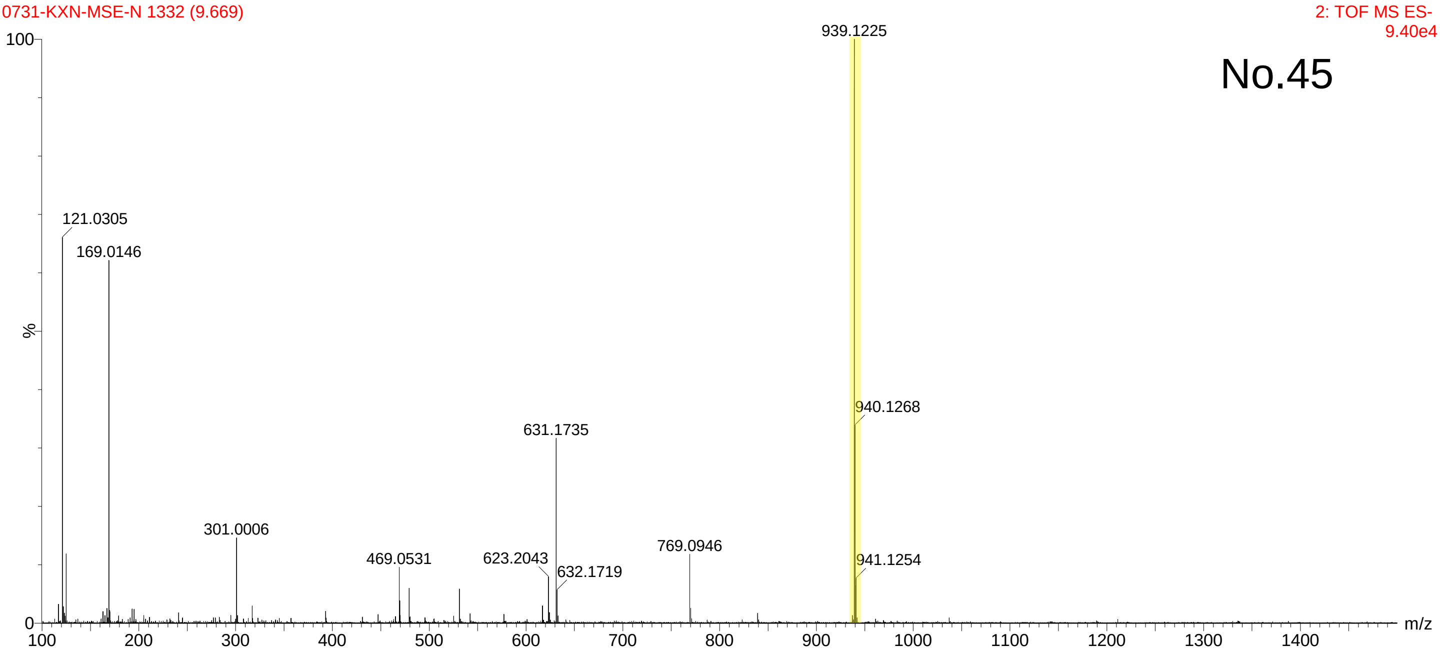

0731-KXN-MSE-N 1332 (9.669)
2: TOF MS ES-
9.40e4
No.45
939.1225
121.0305
169.0146
940.1268
631.1735
301.0006
769.0946
469.0531
623.2043
632.1719
941.1254
100
%
0
m/z
100 200 300 400 500 600 700 800 900 1000 1100 1200 1300 1400

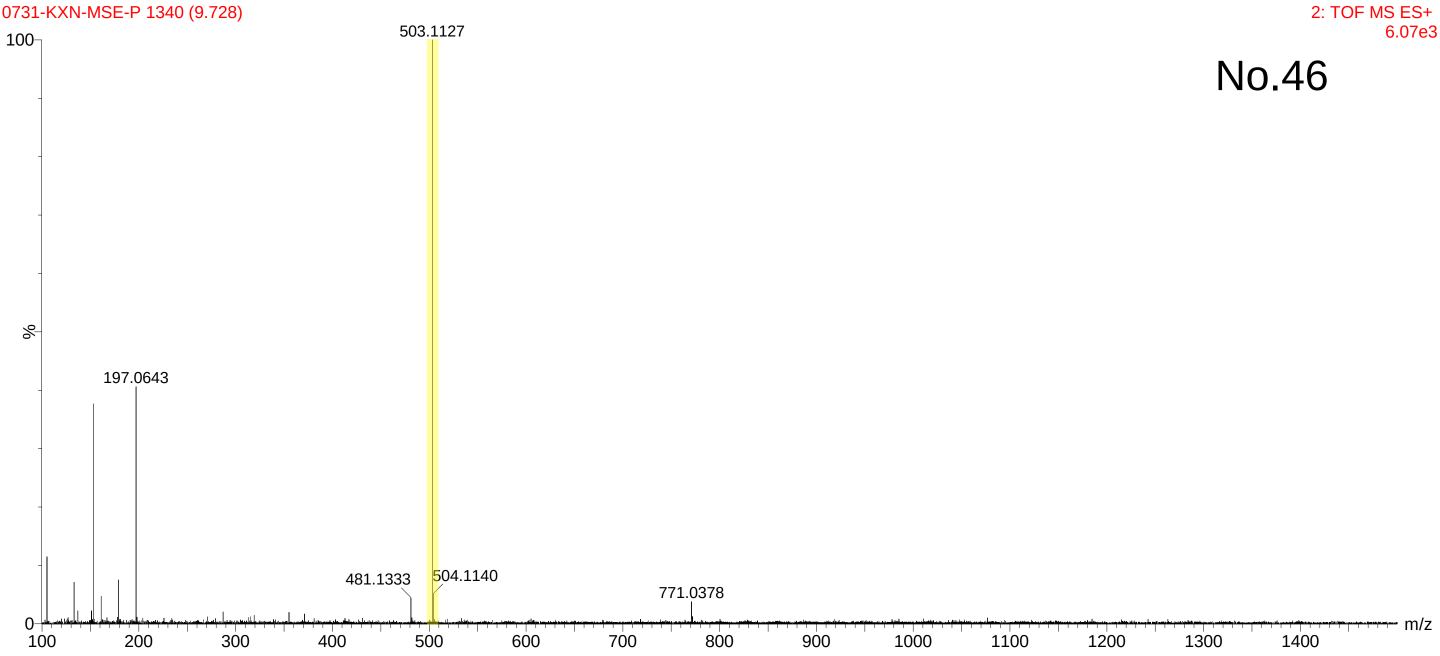
0731-KXN-MSE-P 1340 (9.728)
2: TOF MS ES+
6.07e3
No.46
503.1127
197.0643
481.1333
504.1140
771.0378
100
%
0
m/z

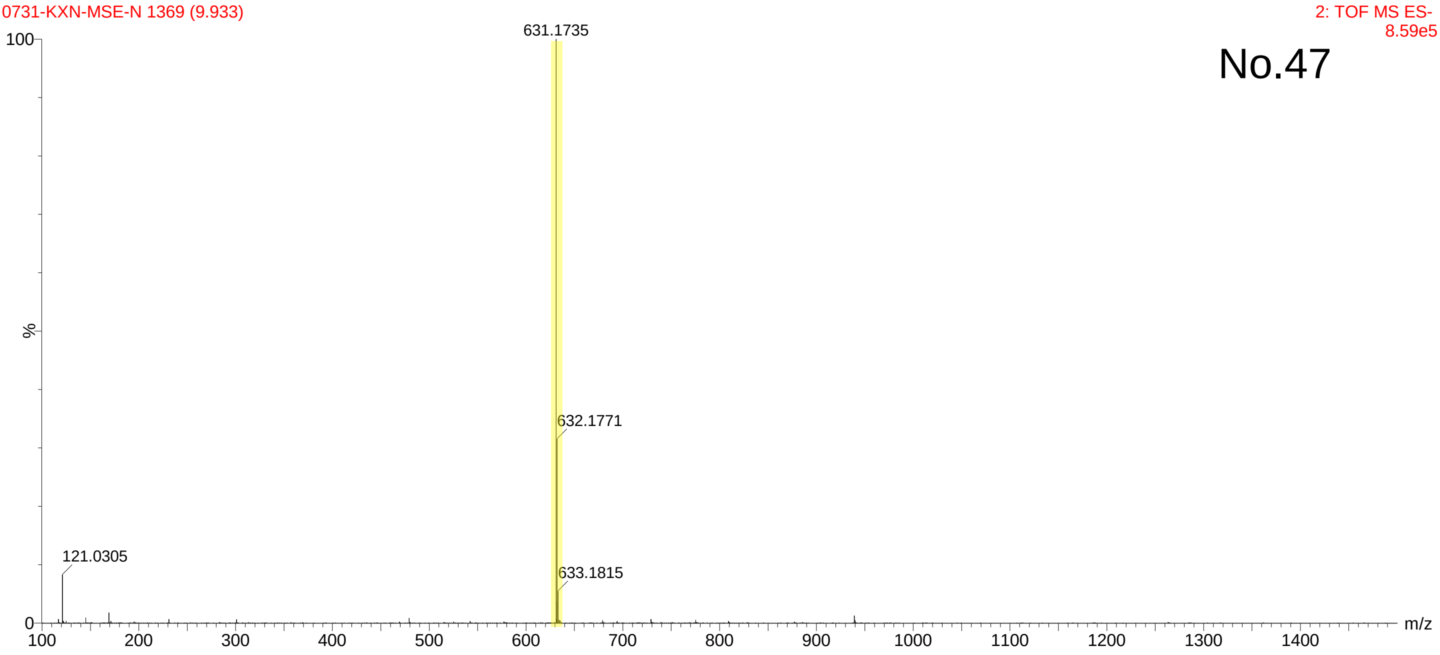
0731-KXN-MSE-N 1369 (9.933)
2: TOF MS ES-
8.59e5
No.47
631.1735
632.1771
121.0305
633.1815
100
%
0
m/z

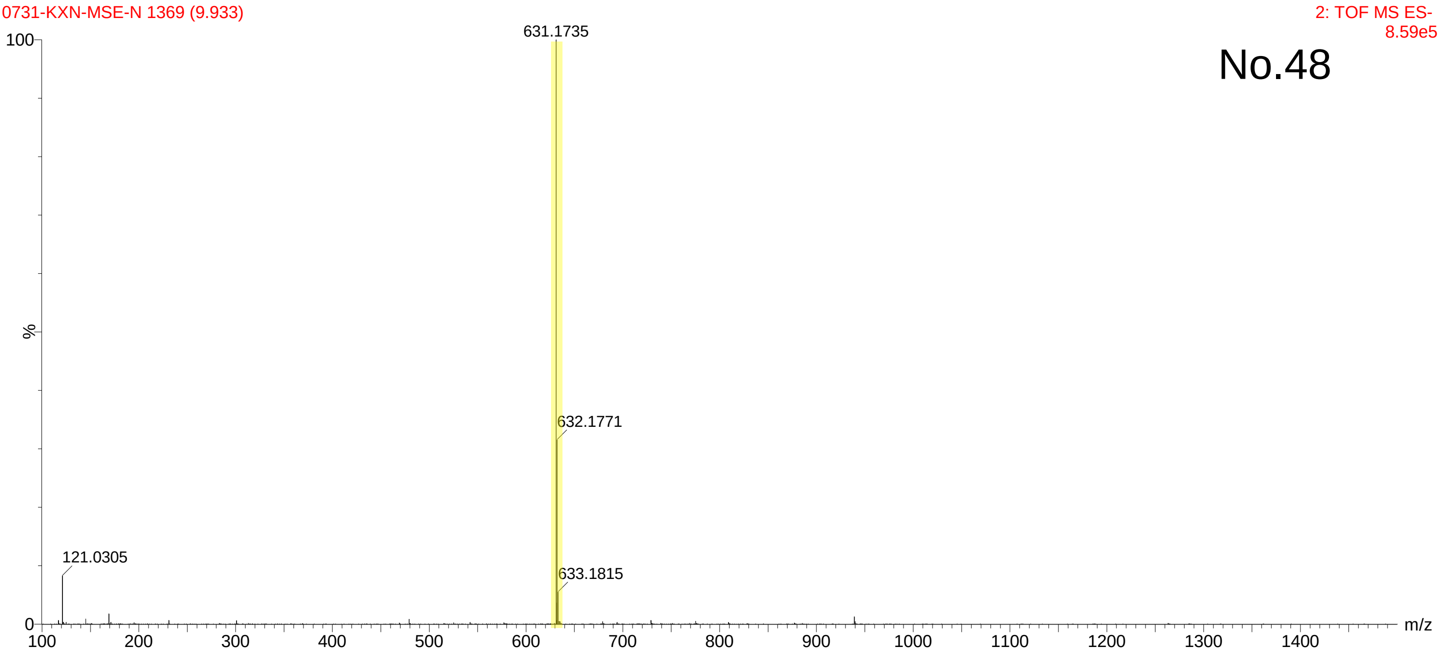
0731-KXN-MSE-N 1369 (9.933)
2: TOF MS ES-
8.59e5
No.48
631.1735
632.1771
121.0305
633.1815
100
%
0
m/z

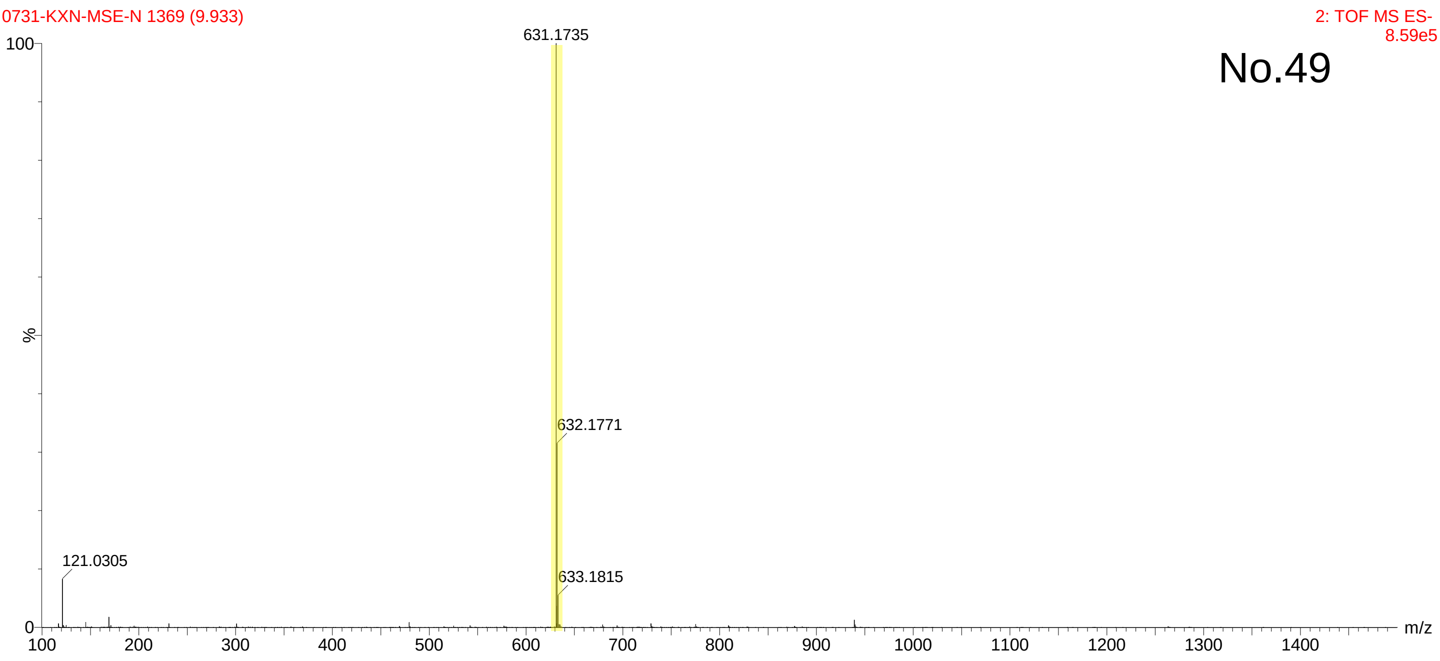
0731-KXN-MSE-N 1369 (9.933)
2: TOF MS ES-
8.59e5
No.49
631.1735
632.1771
633.1815
121.0305
%
m/z

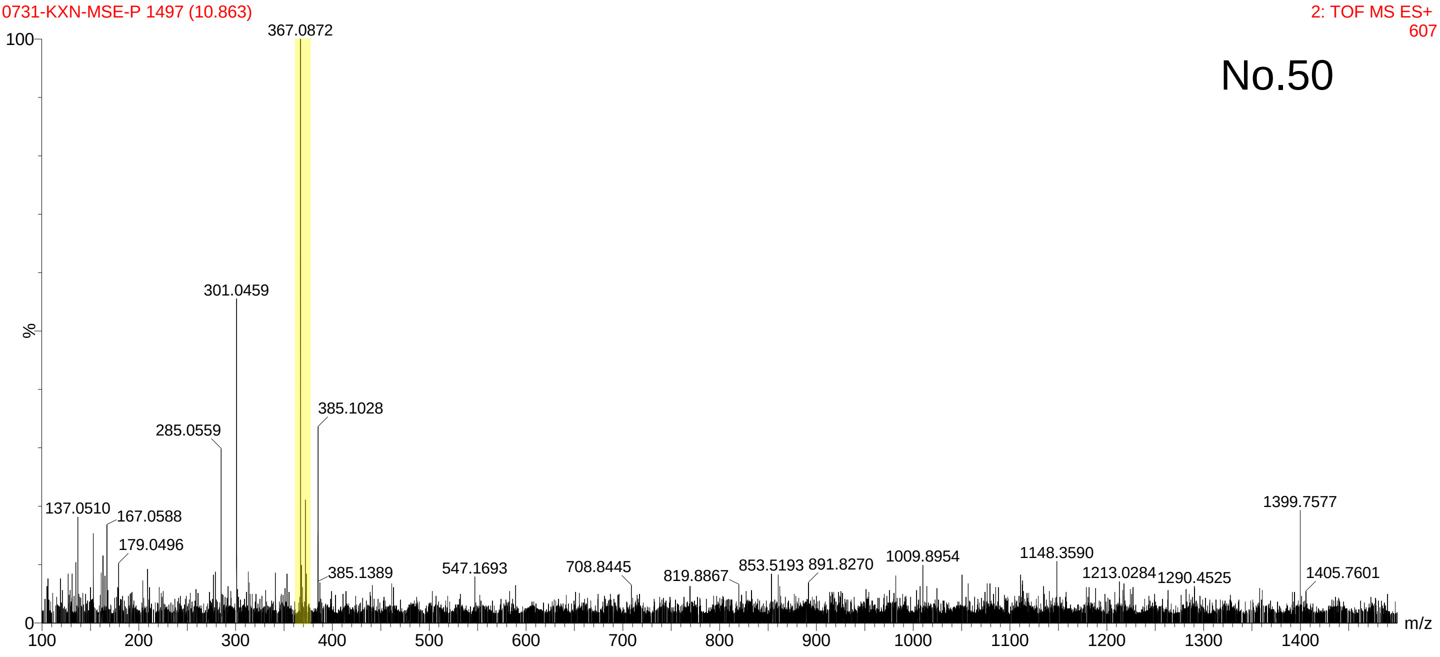
0731-KXN-MSE-P 1497 (10.863)
2: TOF MS ES+
607
No.50
367.0872
301.0459
385.1028
285.0559
137.0510
167.0588
179.0496
385.1389
547.1693
708.8445
819.8867
853.5193
891.8270
1009.8954
1148.3590
1213.0284
1290.4525
1399.7577
1405.7601
%
m/z

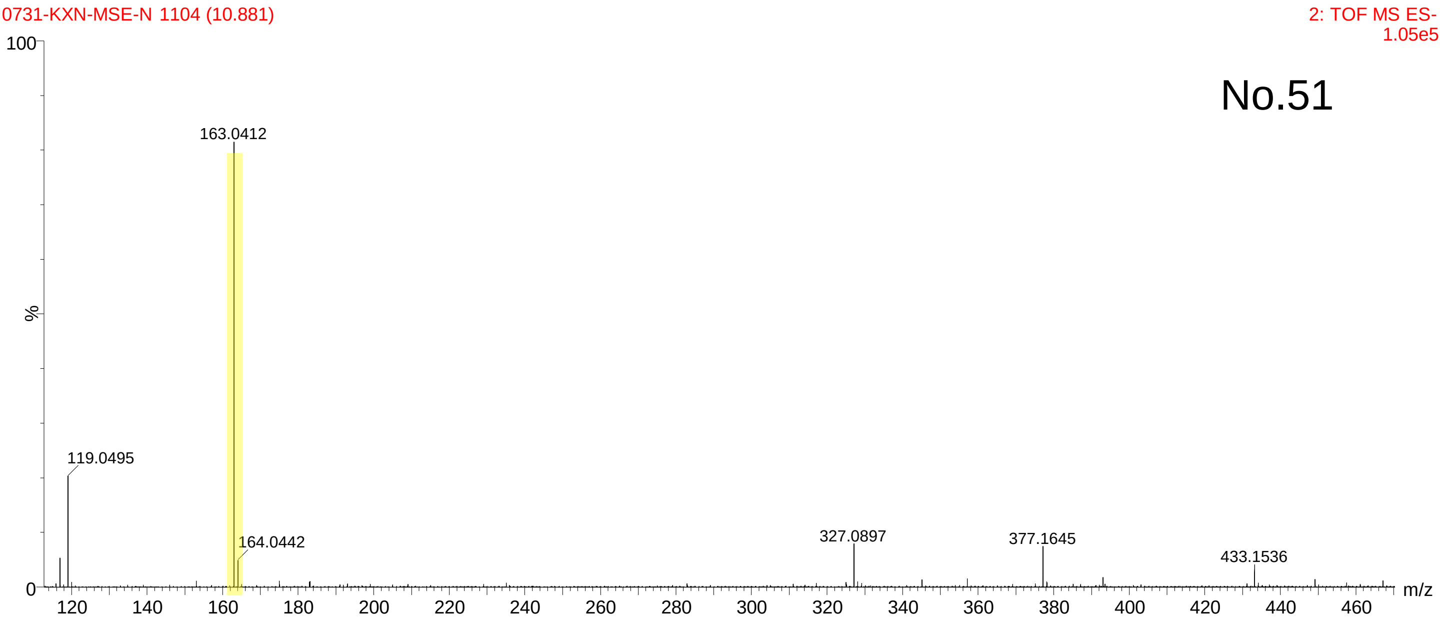
0731-KXN-MSE-N 1104 (10.881)
2: TOF MS ES-
1.05e5
No.51
163.0412
119.0495
164.0442
327.0897
377.1645
433.1536
%
m/z

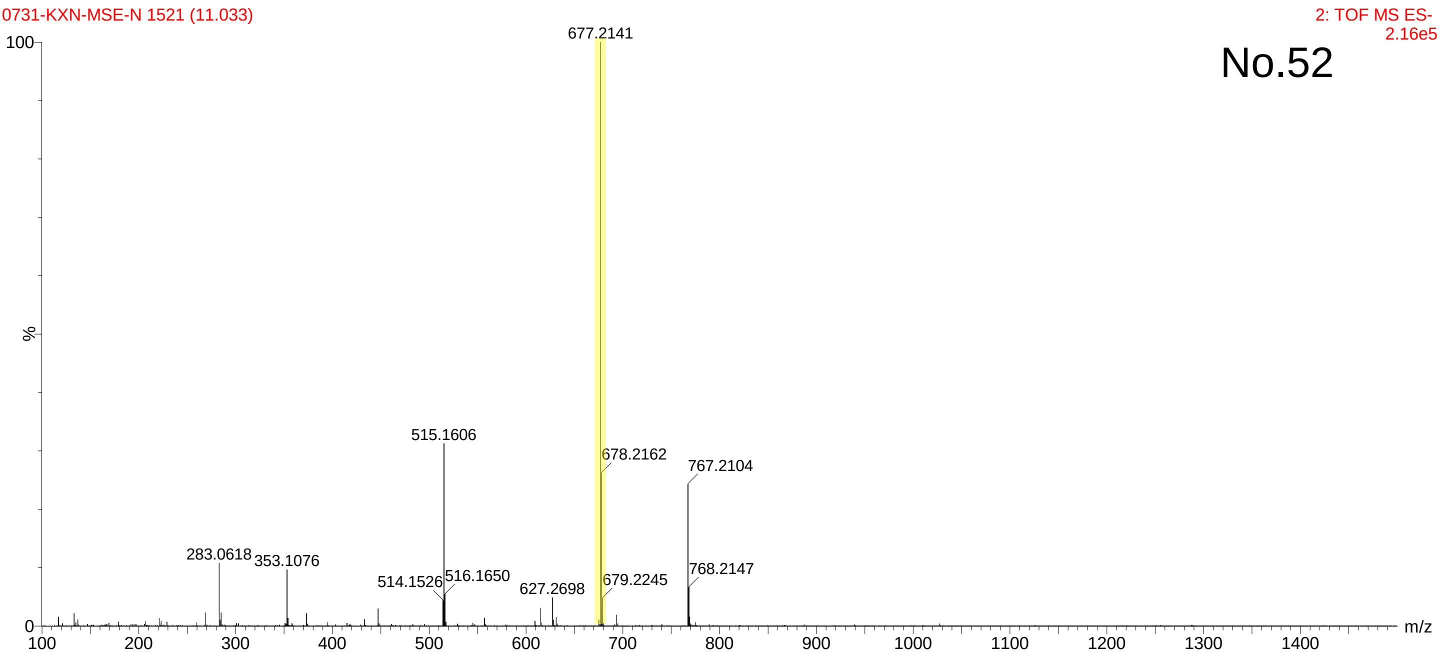
0731-KXN-MSE-N 1521 (11.033)
2: TOF MS ES-
2.16e5
No.52
677.2141
515.1606
678.2162
767.2104
283.0618
353.1076
514.1526
516.1650
627.2698
679.2245
768.2147
%
m/z

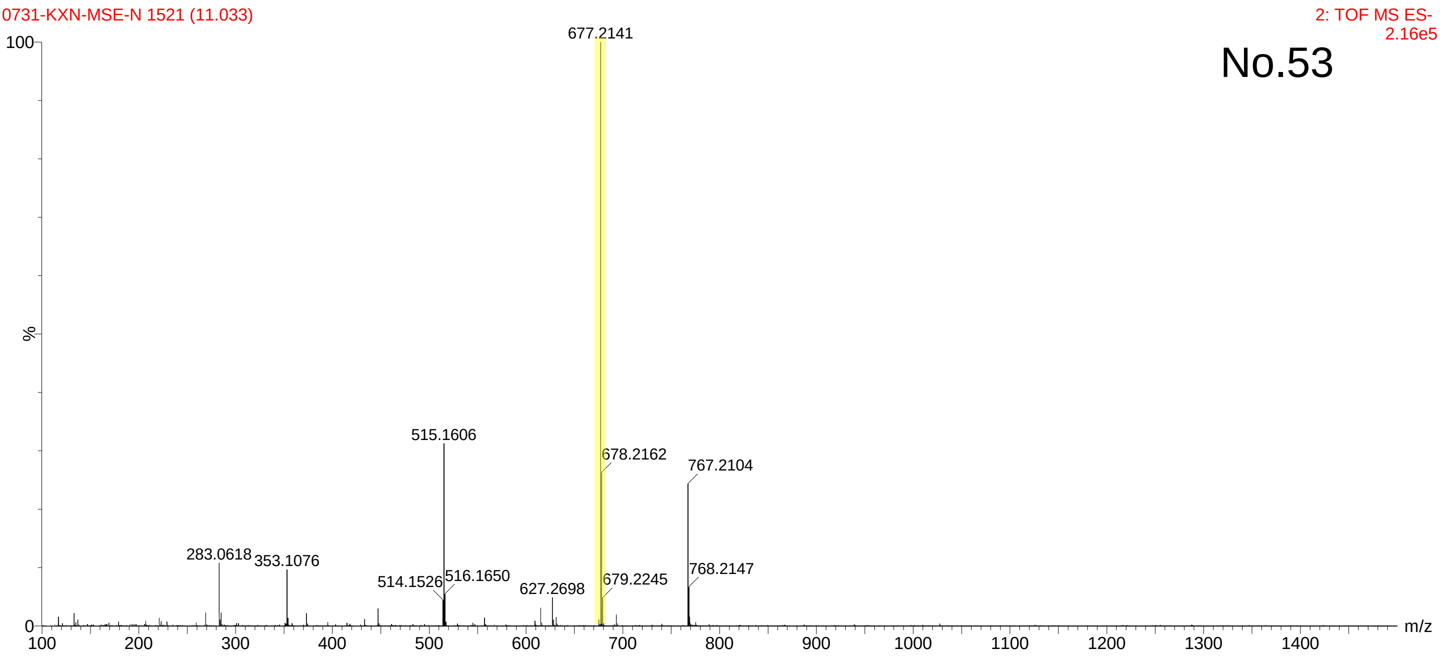
0731-KXN-MSE-N 1521 (11.033)
2: TOF MS ES-
2.16e5
No.53
677.2141
515.1606
678.2162
767.2104
283.0618
353.1076
514.1526
516.1650
627.2698
679.2245
768.2147
%
m/z

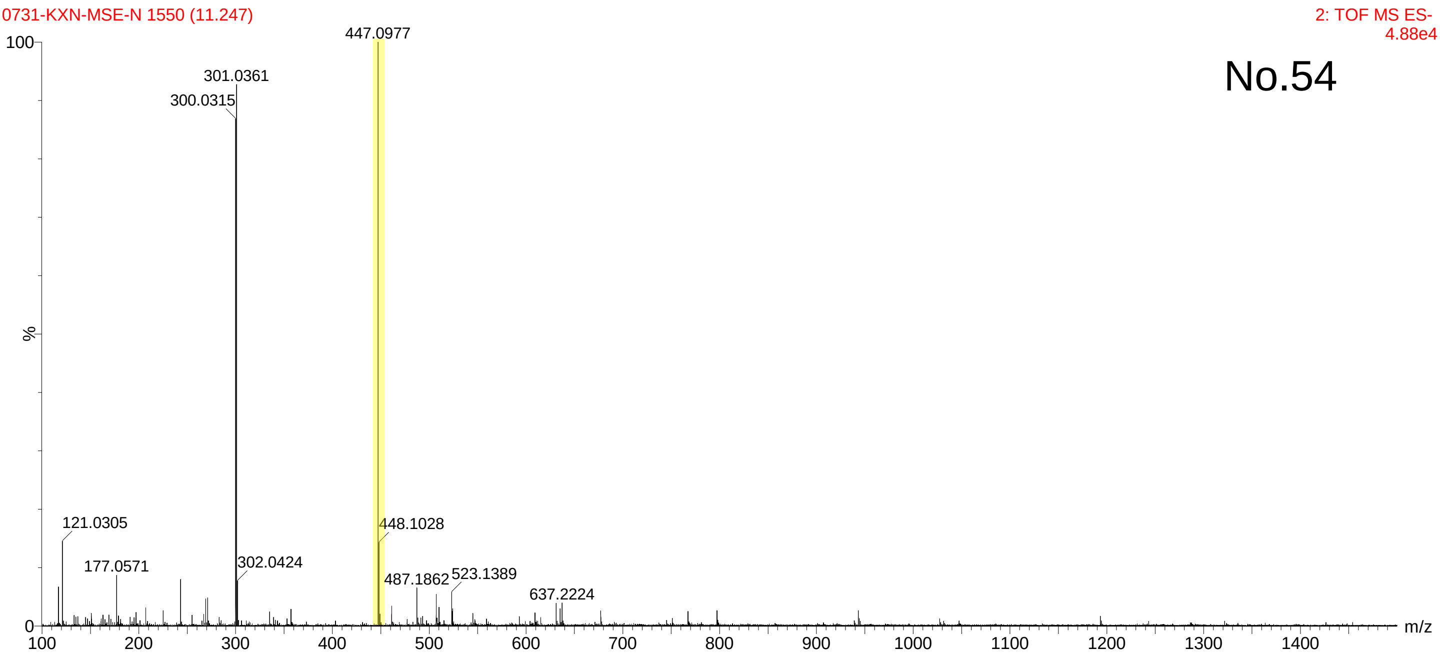
0731-KXN-MSE-N 1550 (11.247)
2: TOF MS ES-
4.88e4
No.54
447.0977
301.0361
300.0315
121.0305
448.1028
177.0571
302.0424
487.1862
523.1389
637.2224
%
m/z

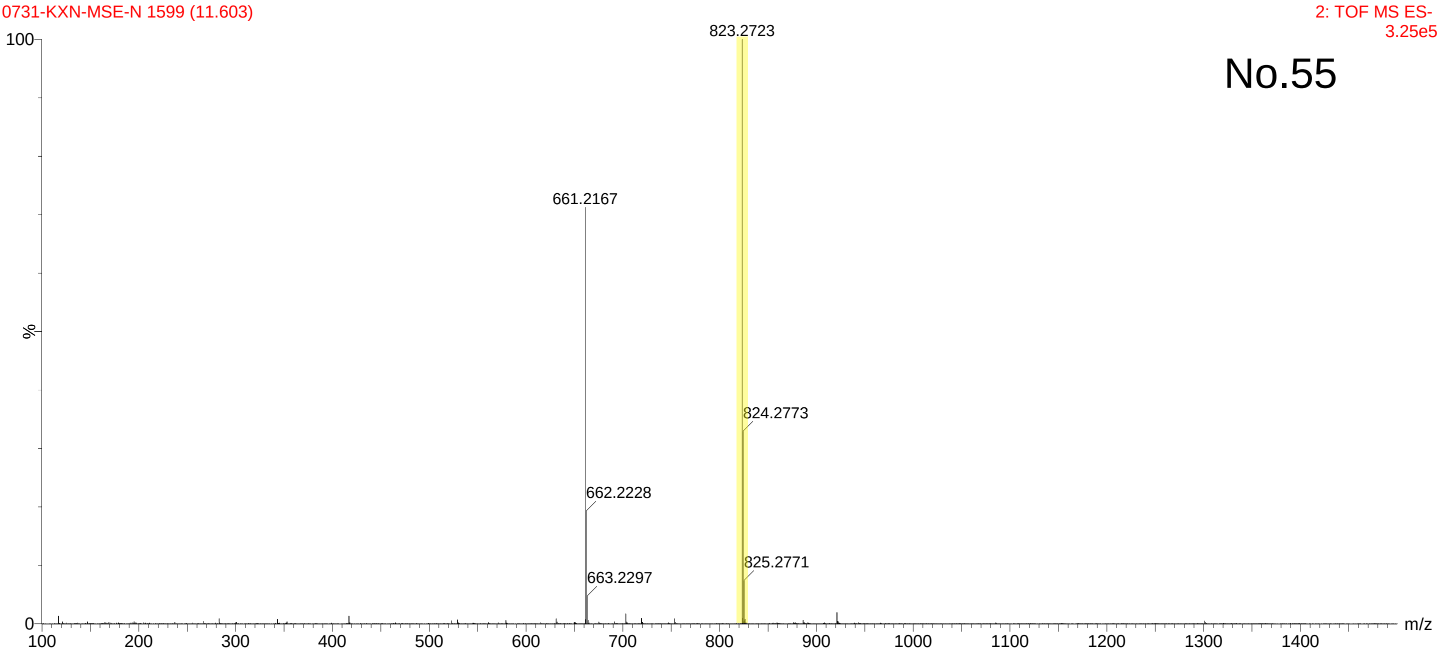
0731-KXN-MSE-N 1599 (11.603)
2: TOF MS ES-
3.25e5
No.55
823.2723
661.2167
824.2773
662.2228
825.2771
663.2297
100
%
0
100
200
300
400
500
600
700
800
900
1000
1100
1200
1300
1400
m/z

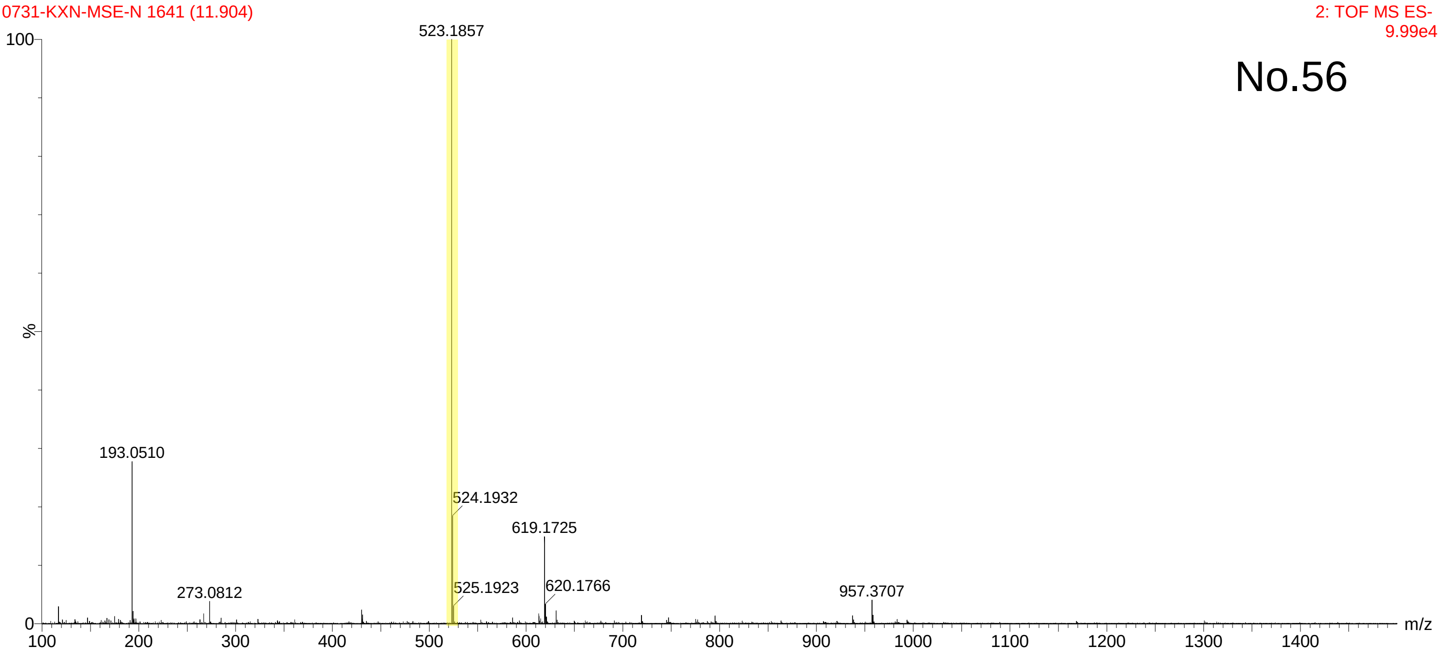
0731-KXN-MSE-N 1641 (11.904)
2: TOF MS ES-
9.99e4
No.56
523.1857
193.0510
524.1932
619.1725
273.0812
525.1923
620.1766
957.3707
100
%
0
100
200
300
400
500
600
700
800
900
1000
1100
1200
1300
1400
m/z

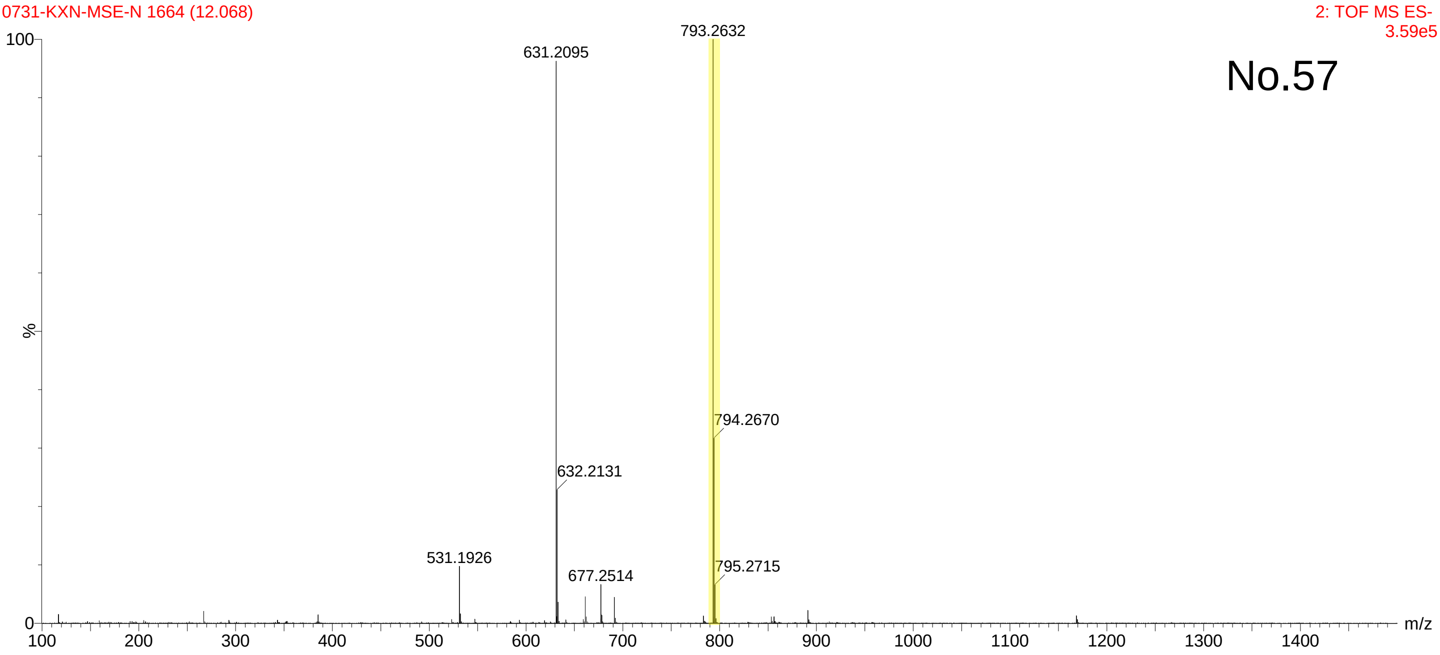
0731-KXN-MSE-N 1664 (12.068)
2: TOF MS ES-
3.59e5
No.57
793.2632
631.2095
794.2670
632.2131
531.1926
677.2514
795.2715
100
%
0
100
200
300
400
500
600
700
800
900
1000
1100
1200
1300
1400
m/z

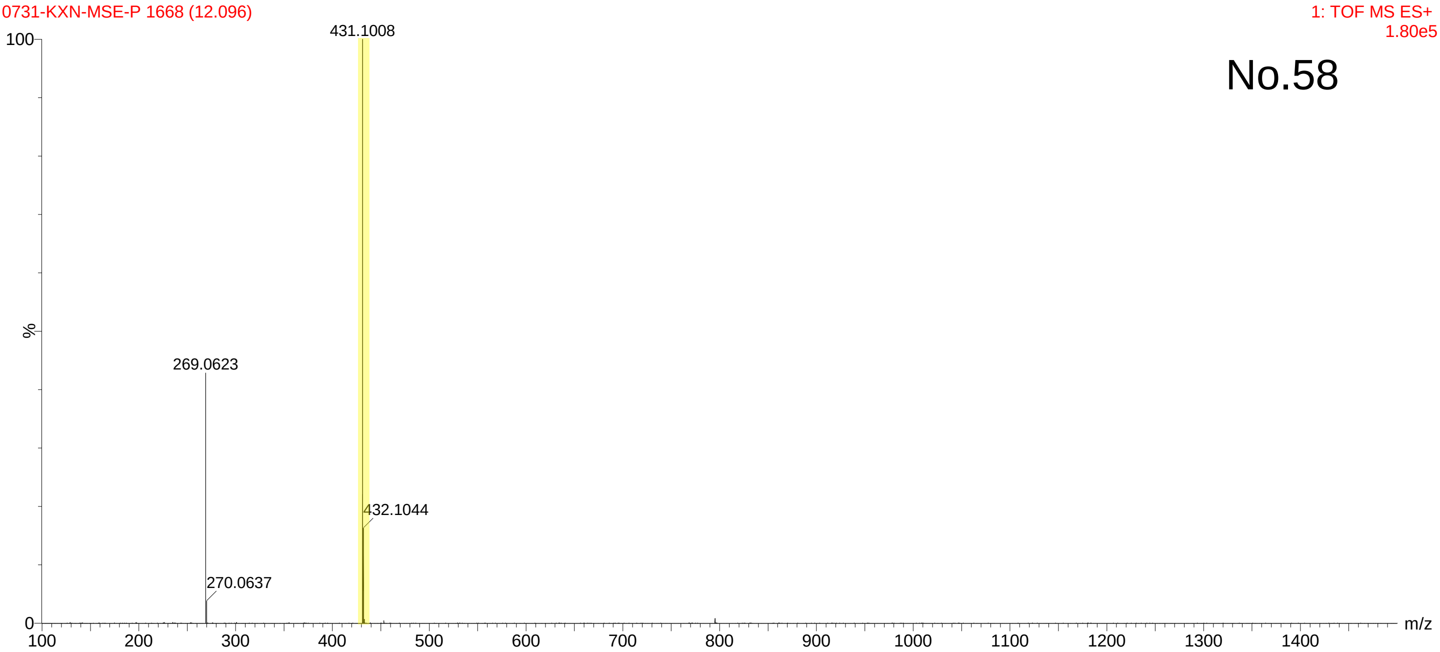
0731-KXN-MSE-P 1668 (12.096)
1: TOF MS ES+
1.80e5
No.58
431.1008
269.0623
432.1044
270.0637
%
m/z

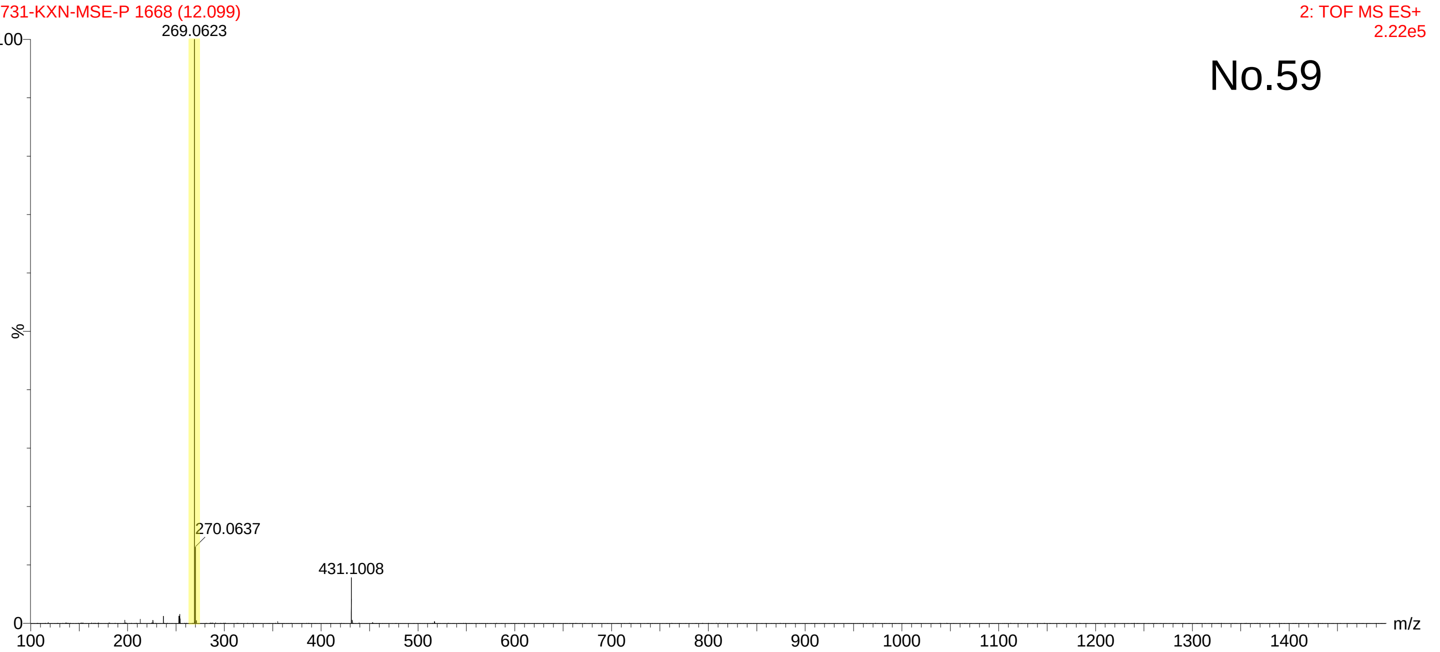
731-KXN-MSE-P 1668 (12.099)
2: TOF MS ES+
2.22e5
No.59
269.0623
270.0637
431.1008
%
m/z

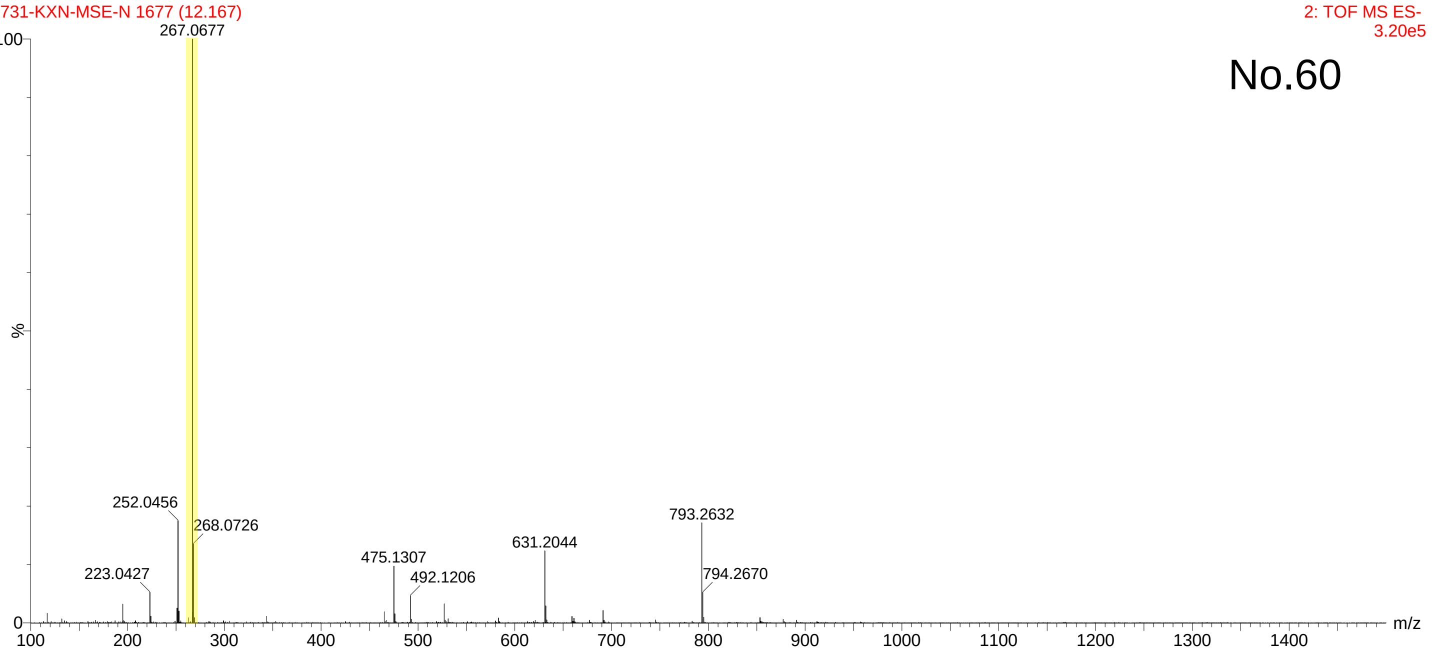
731-KXN-MSE-N 1677 (12.167)
2: TOF MS ES-
3.20e5
No.60
267.0677
252.0456
268.0726
223.0427
475.1307
492.1206
631.2044
793.2632
794.2670
%
m/z

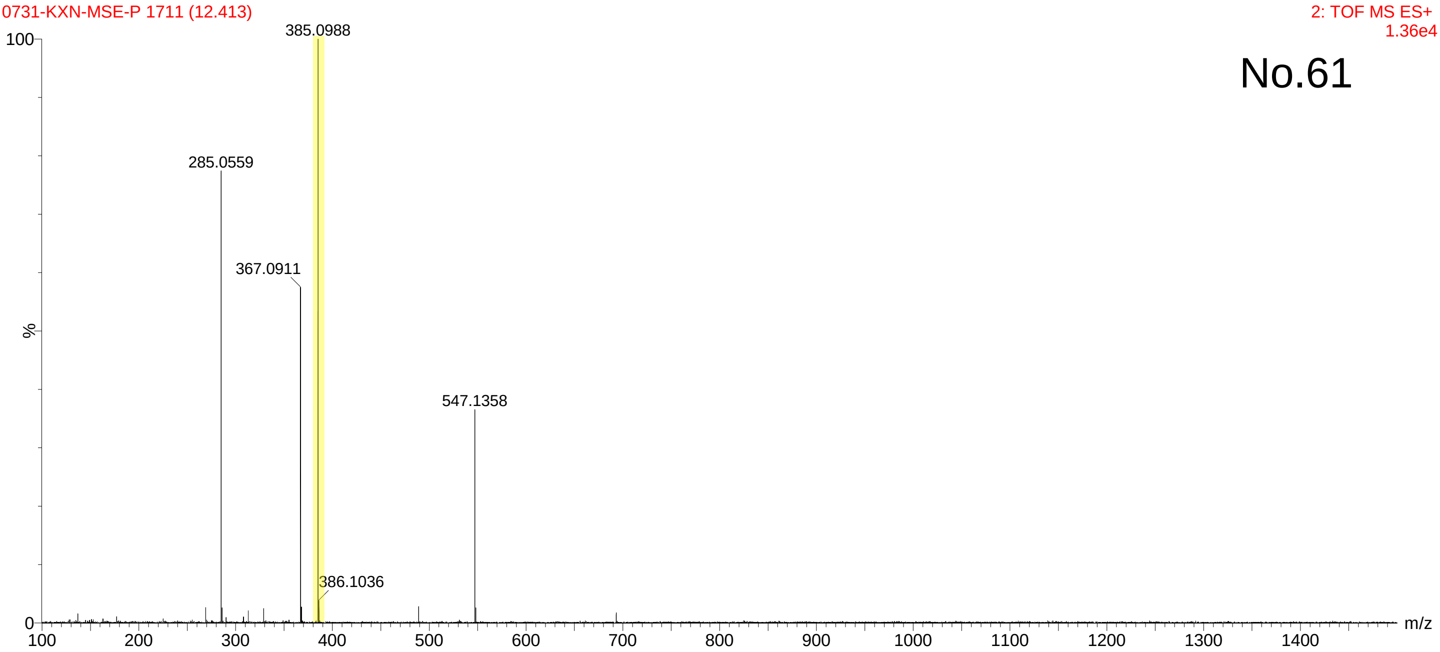
0731-KXN-MSE-P 1711 (12.413)
2: TOF MS ES+
1.36e4
No.61
385.0988
285.0559
367.0911
547.1358
386.1036
%
m/z

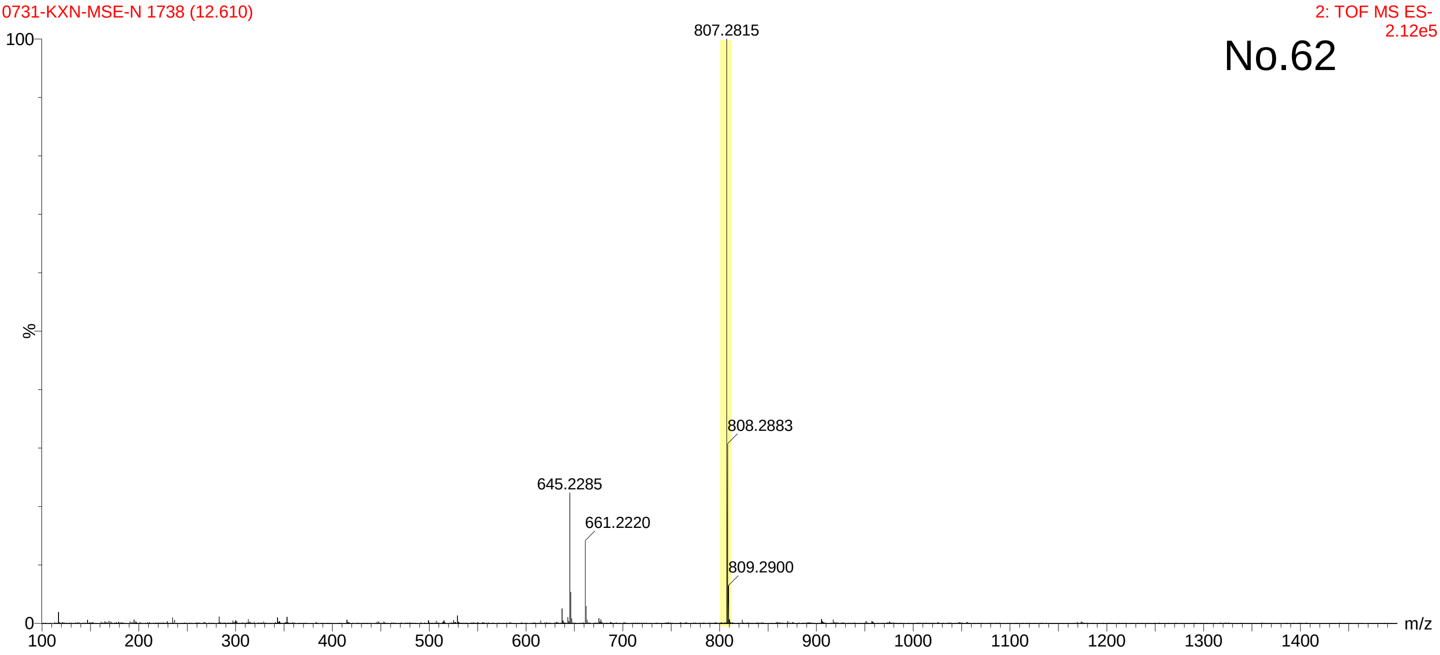
0731-KXN-MSE-N 1738 (12.610)
2: TOF MS ES-
2.12e5
No.62
807.2815
808.2883
645.2285
661.2220
809.2900
%
m/z

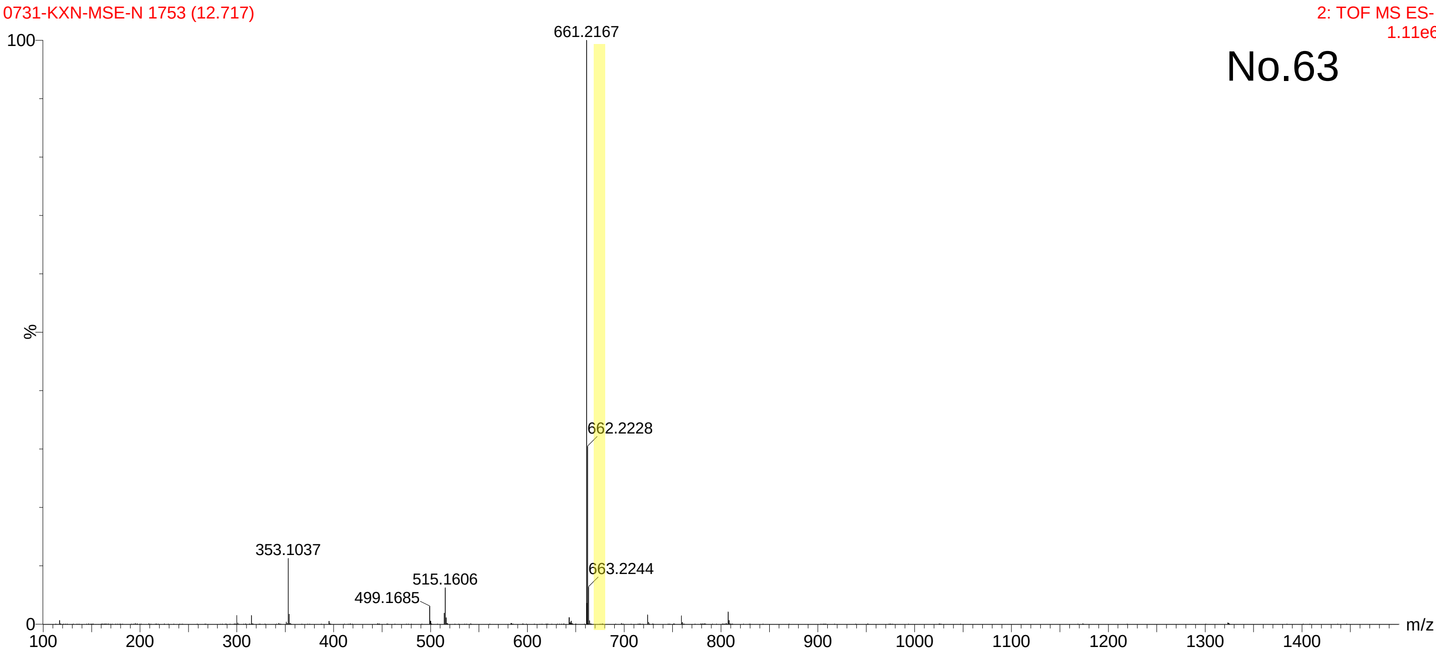
0731-KXN-MSE-N 1753 (12.717)
2: TOF MS ES-
No.63
661.2167
662.2228
353.1037
515.1606
499.1685
663.2244
%
m/z

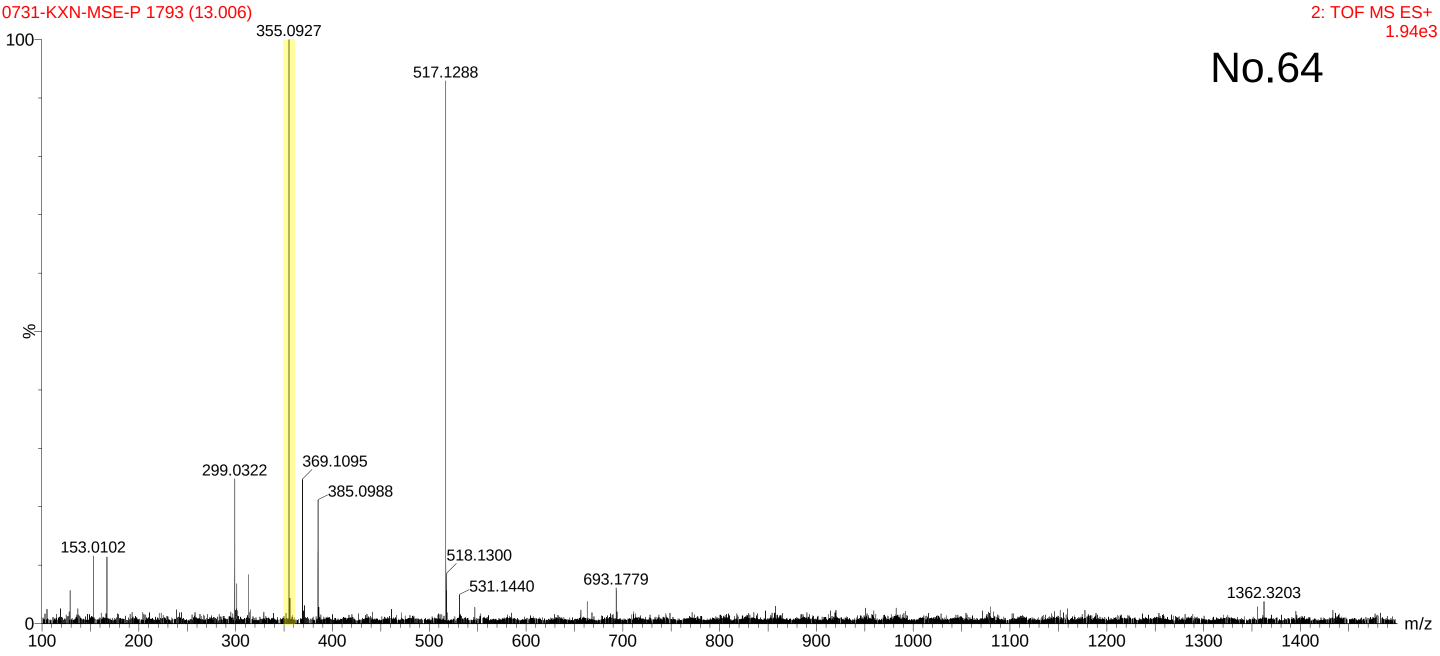
0731-KXN-MSE-P 1793 (13.006)
2: TOF MS ES+
1.94e3
No.64
355.0927
517.1288
299.0322
369.1095
385.0988
153.0102
518.1300
531.1440
693.1779
1362.3203
100
%
0
m/z
100 200 300 400 500 600 700 800 900 1000 1100 1200 1300 1400

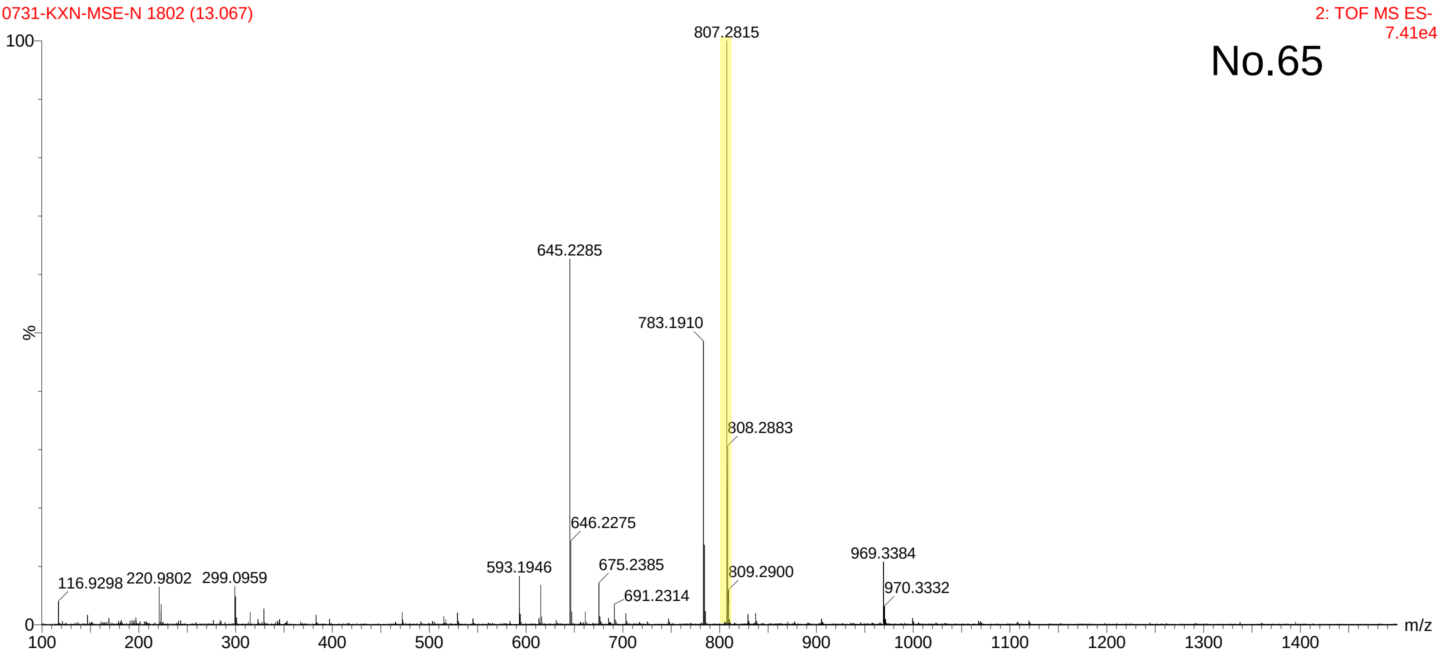
0731-KXN-MSE-N 1802 (13.067)
2: TOF MS ES-
7.41e4
No.65
807.2815
645.2285
783.1910
808.2883
646.2275
675.2385
593.1946
691.2314
809.2900
969.3384
970.3332
116.9298
220.9802
299.0959
100
%
0
m/z
100 200 300 400 500 600 700 800 900 1000 1100 1200 1300 1400

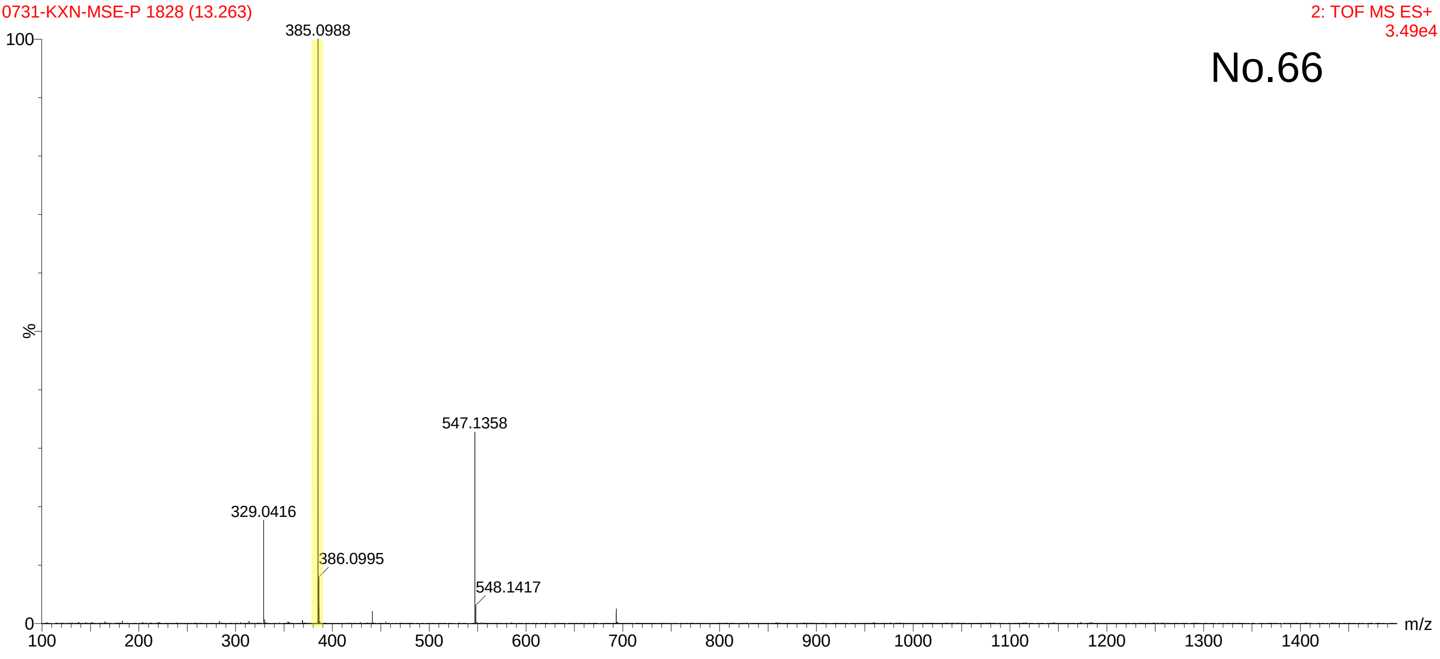
0731-KXN-MSE-P 1828 (13.263)
2: TOF MS ES+
3.49e4
No.66
385.0988
547.1358
329.0416
386.0995
548.1417
100
%
0
m/z
100 200 300 400 500 600 700 800 900 1000 1100 1200 1300 1400

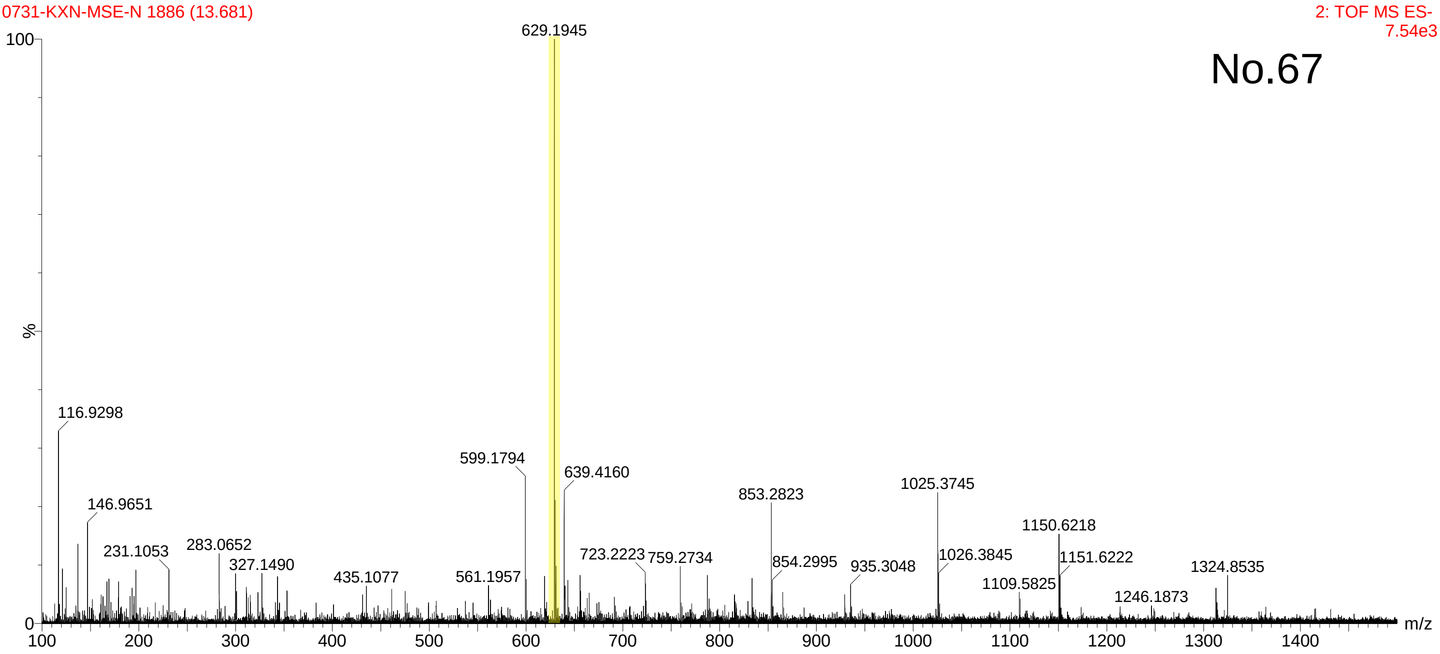
0731-KXN-MSE-N 1886 (13.681)
2: TOF MS ES-
7.54e3
No.67
629.1945
116.9298
599.1794
639.4160
146.9651
853.2823
1025.3745
1150.6218
231.1053
283.0652
327.1490
723.2223
759.2734
854.2995
935.3048
1026.3845
1151.6222
1324.8535
435.1077
561.1957
1109.5825
1246.1873
100
%
0
m/z
100 200 300 400 500 600 700 800 900 1000 1100 1200 1300 1400

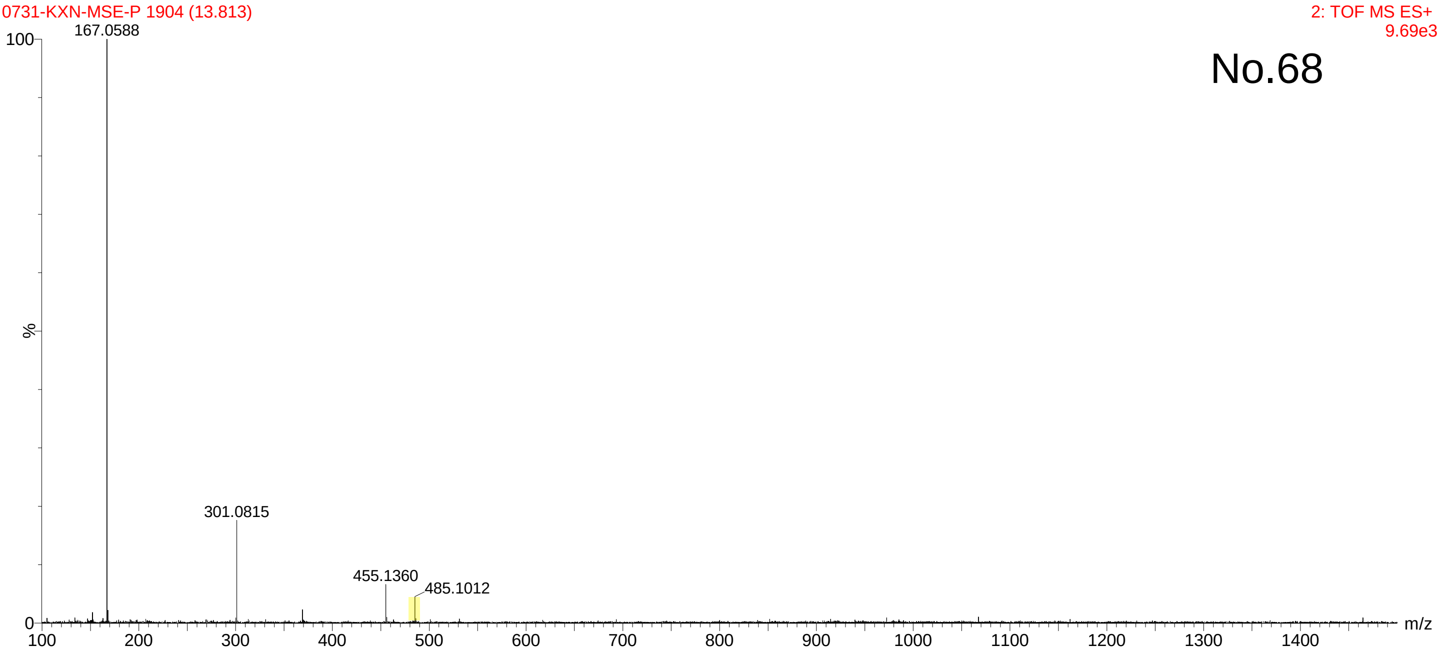
0731-KXN-MSE-P 1904 (13.813)
2: TOF MS ES+
9.69e3
No.68
167.0588
301.0815
455.1360
485.1012
100
%
0
m/z
100 200 300 400 500 600 700 800 900 1000 1100 1200 1300 1400

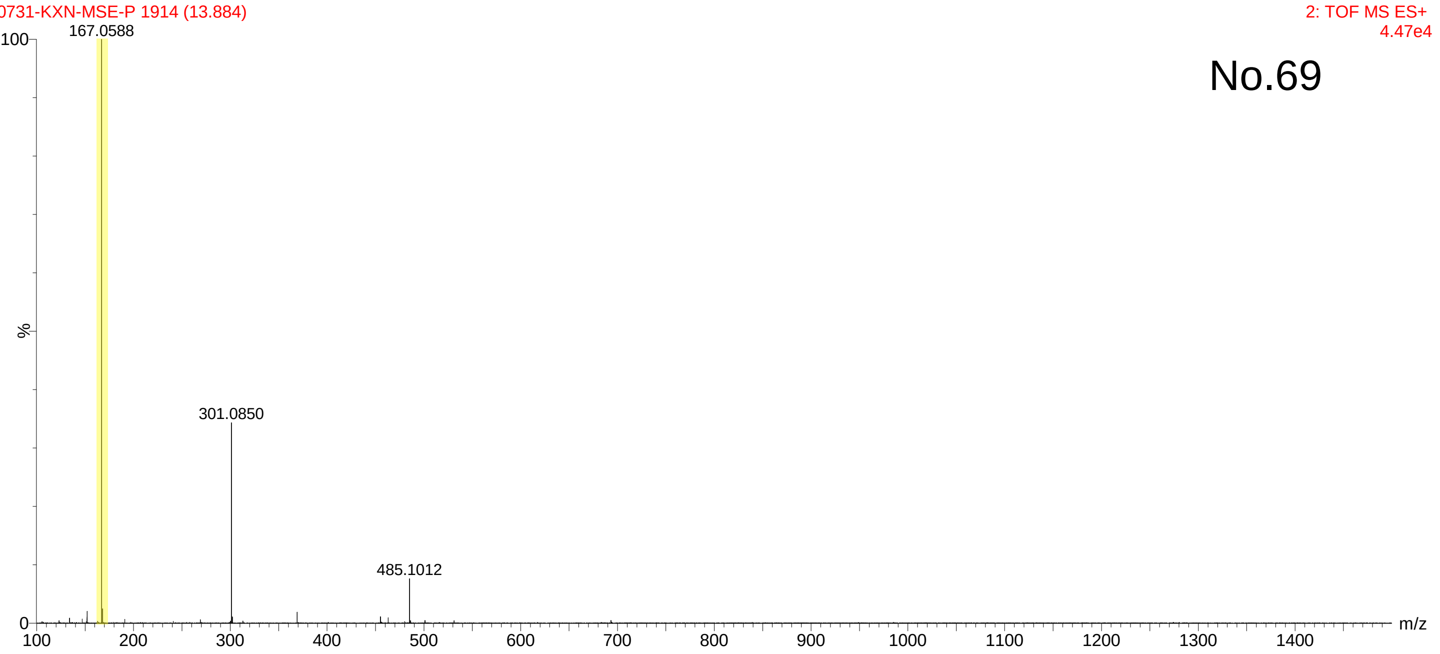
0731-KXN-MSE-P 1914 (13.884)
2: TOF MS ES+
4.47e4
No.69
167.0588
301.0850
485.1012
100
%
0
m/z
100 200 300 400 500 600 700 800 900 1000 1100 1200 1300 1400

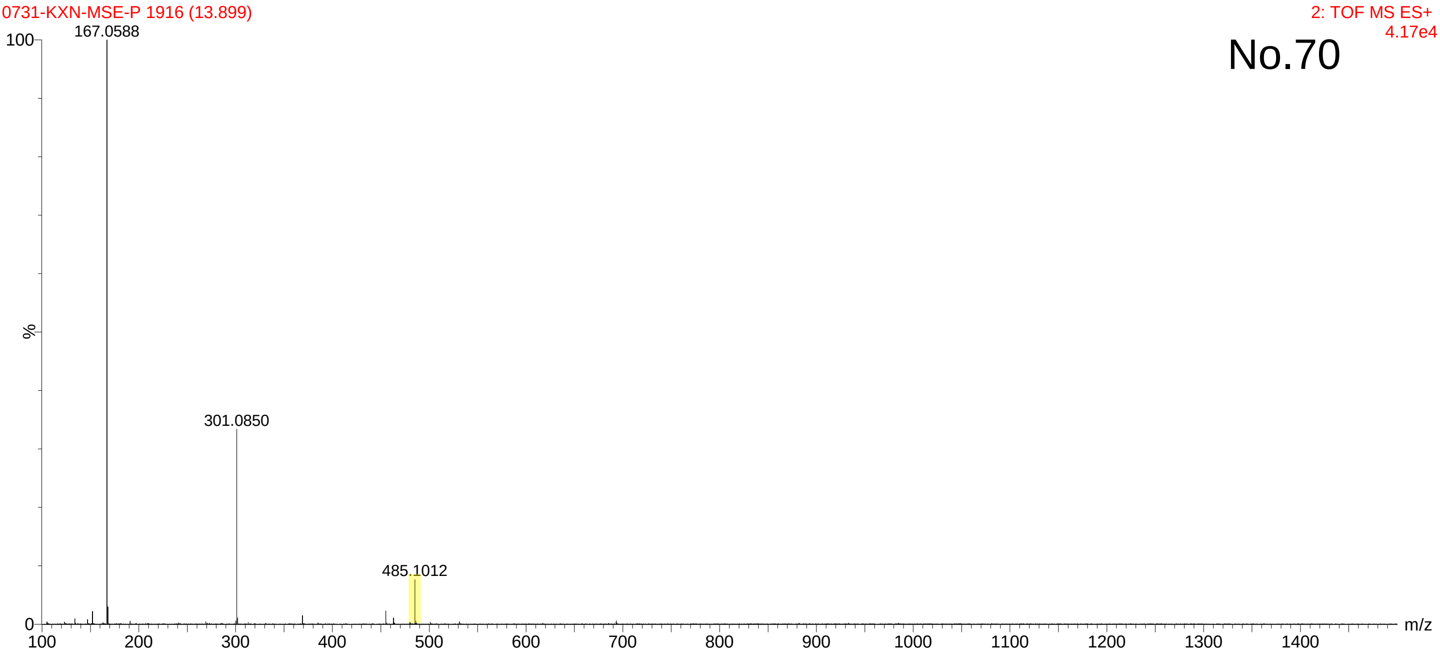
0731-KXN-MSE-P 1916 (13.899)
2: TOF MS ES+
4.17e4
No.70
167.0588
301.0850
485.1012
100
%
0
m/z
100
200
300
400
500
600
700
800
900
1000
1100
1200
1300
1400

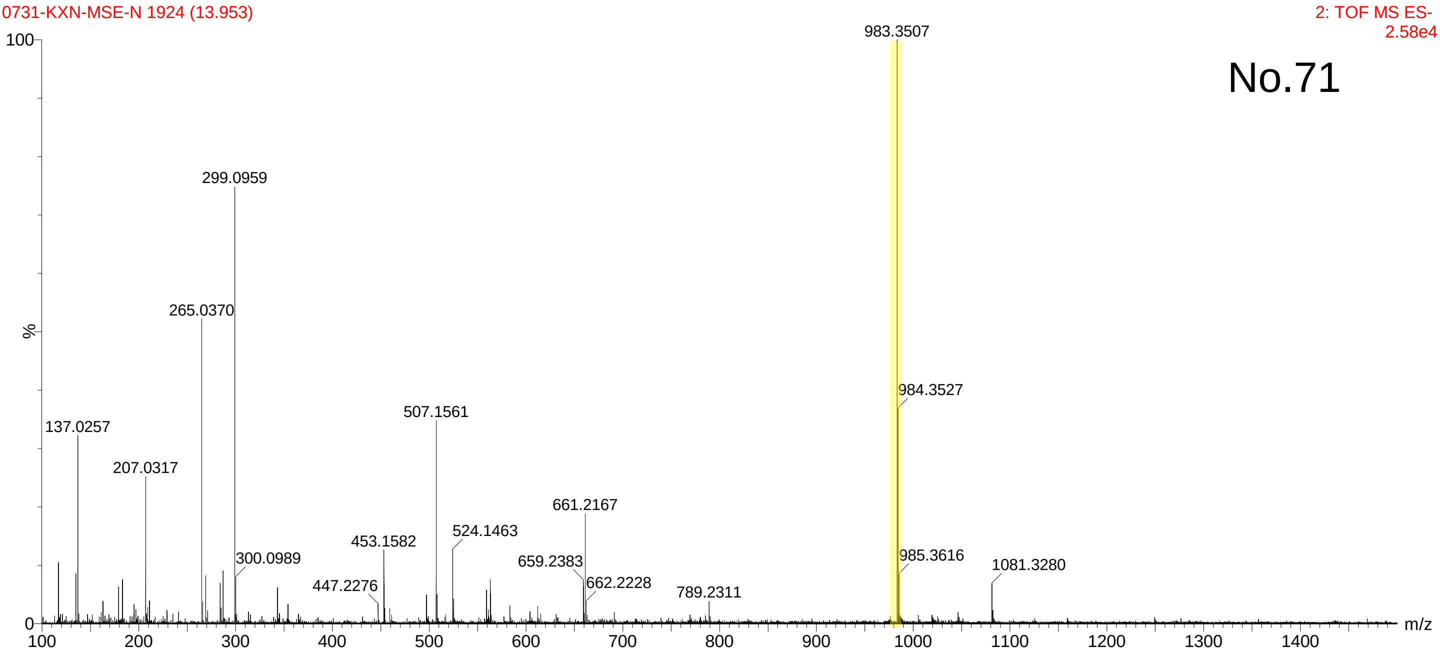
0731-KXN-MSE-N 1924 (13.953)
2: TOF MS ES-
2.58e4
No.71
983.3507
299.0959
265.0370
984.3527
137.0257
507.1561
207.0317
661.2167
524.1463
453.1582
985.3616
659.2383
300.0989
1081.3280
447.2276
662.2228
789.2311
100
%
0
m/z
100
200
300
400
500
600
700
800
900
1000
1100
1200
1300
1400

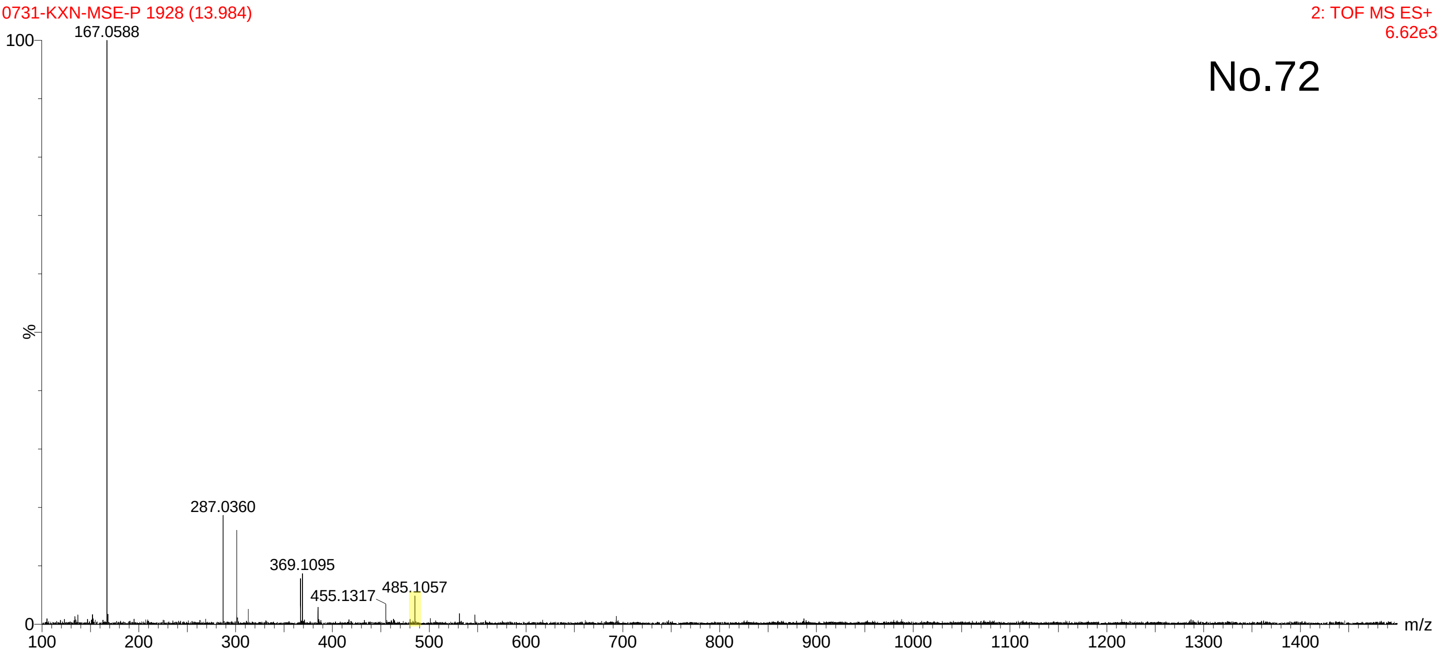
0731-KXN-MSE-P 1928 (13.984)
2: TOF MS ES+
6.62e3
No.72
167.0588
287.0360
369.1095
455.1317
485.1057
100
%
0
m/z
100
200
300
400
500
600
700
800
900
1000
1100
1200
1300
1400

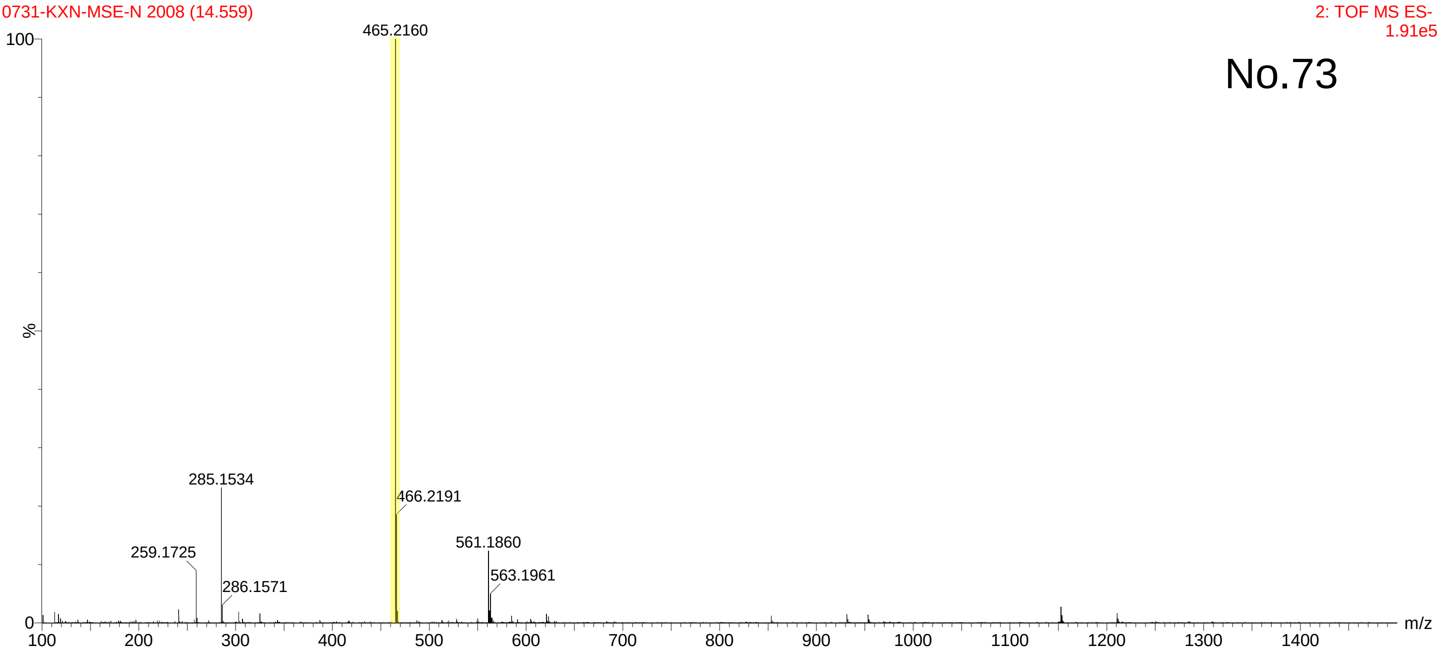
0731-KXN-MSE-N 2008 (14.559)
2: TOF MS ES-
1.91e5
No.73
465.2160
285.1534
466.2191
259.1725
561.1860
286.1571
563.1961
%
m/z

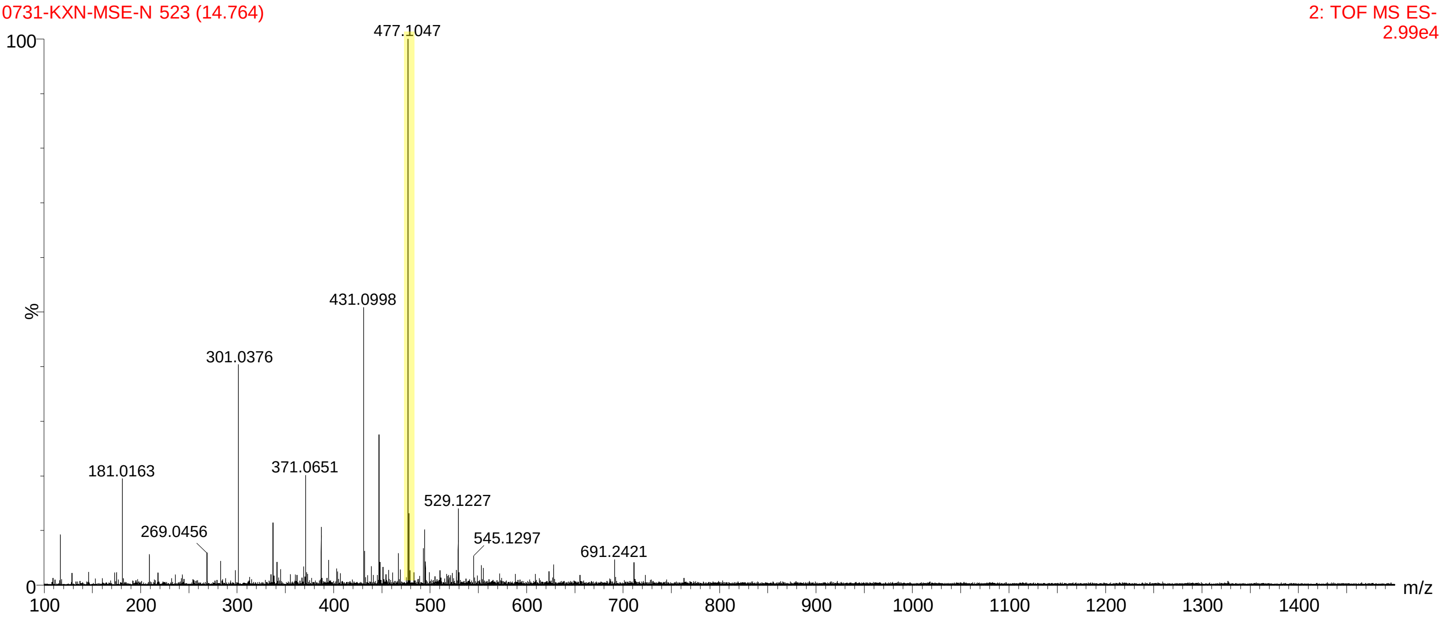
0731-KXN-MSE-N 523 (14.764)
2: TOF MS ES-
2.99e4
477.1047
431.0998
301.0376
181.0163
371.0651
529.1227
269.0456
545.1297
691.2421
%
m/z

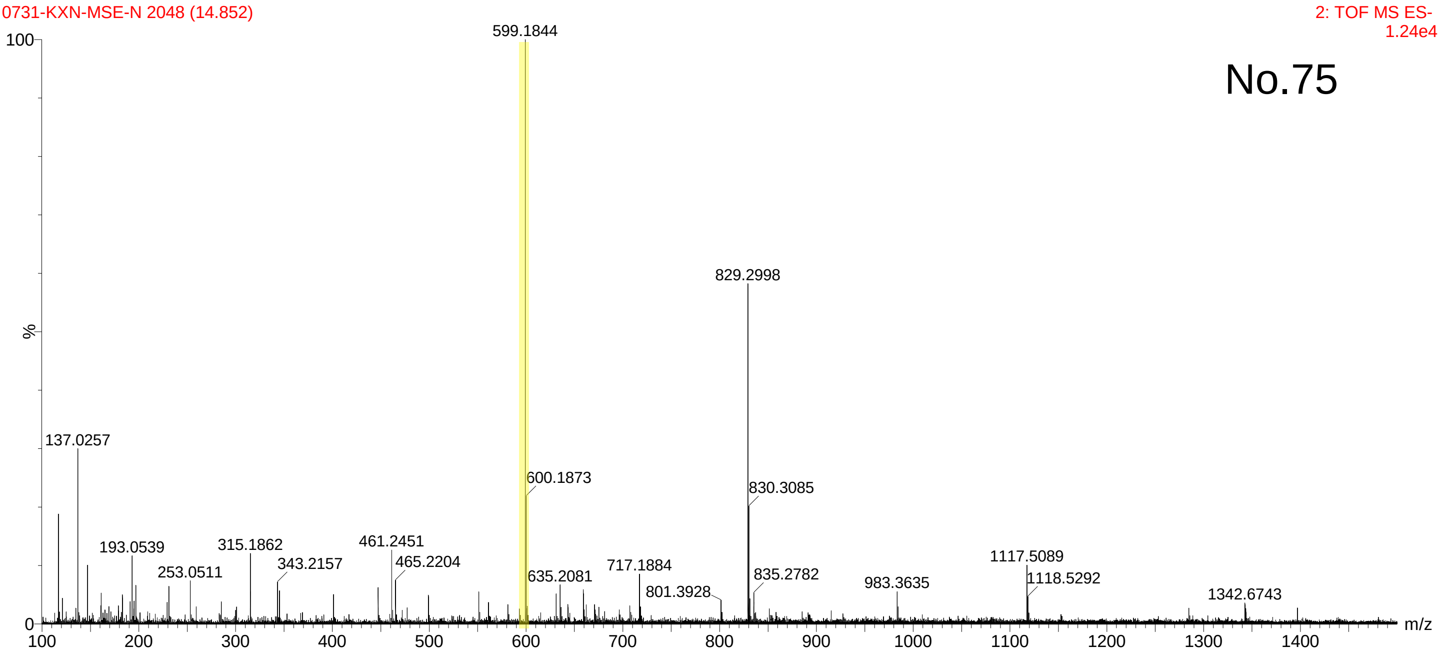
0731-KXN-MSE-N 2048 (14.852)
2: TOF MS ES-
1.24e4
No.75
599.1844
829.2998
137.0257
600.1873
830.3085
193.0539
315.1862
461.2451
253.0511
343.2157
465.2204
635.2081
717.1884
801.3928
835.2782
983.3635
1117.5089
1118.5292
1342.6743
%
m/z

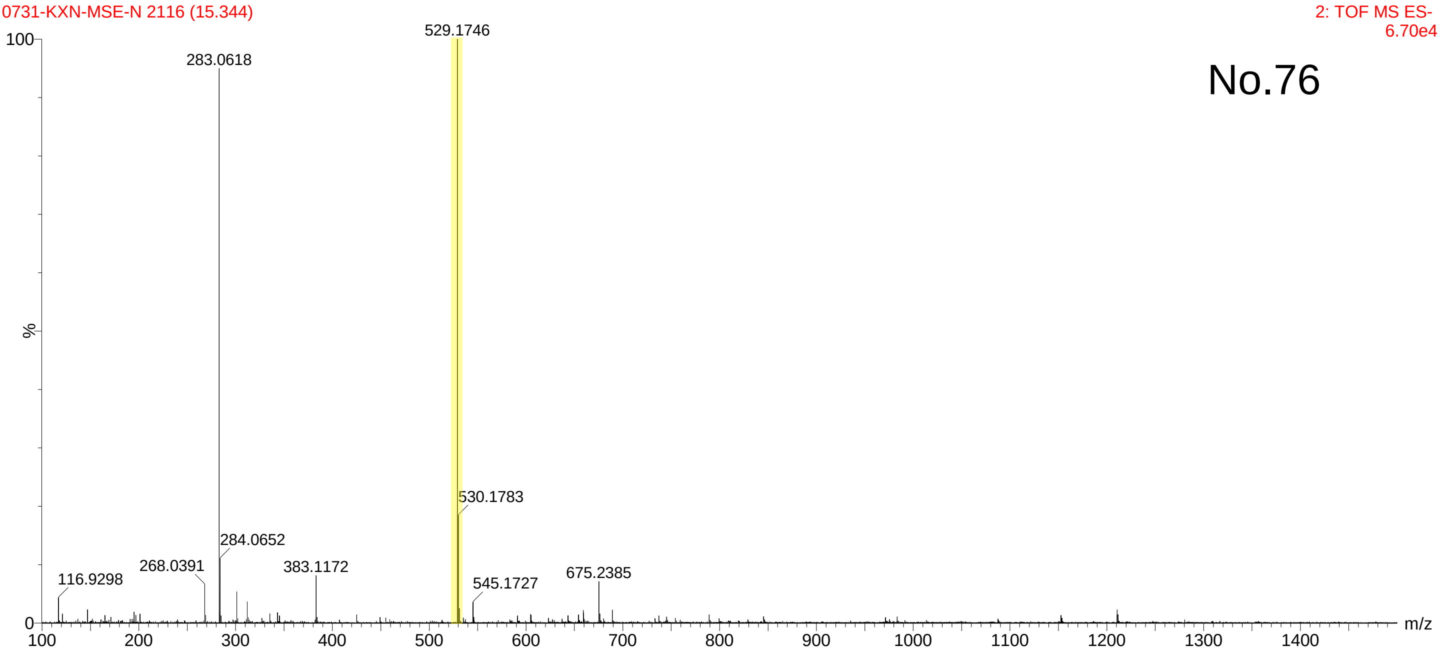
0731-KXN-MSE-N 2116 (15.344)
2: TOF MS ES-
6.70e4
No.76
529.1746
283.0618
530.1783
284.0652
268.0391
383.1172
116.9298
545.1727
675.2385
%
m/z
100
0
100 200 300 400 500 600 700 800 900 1000 1100 1200 1300 1400

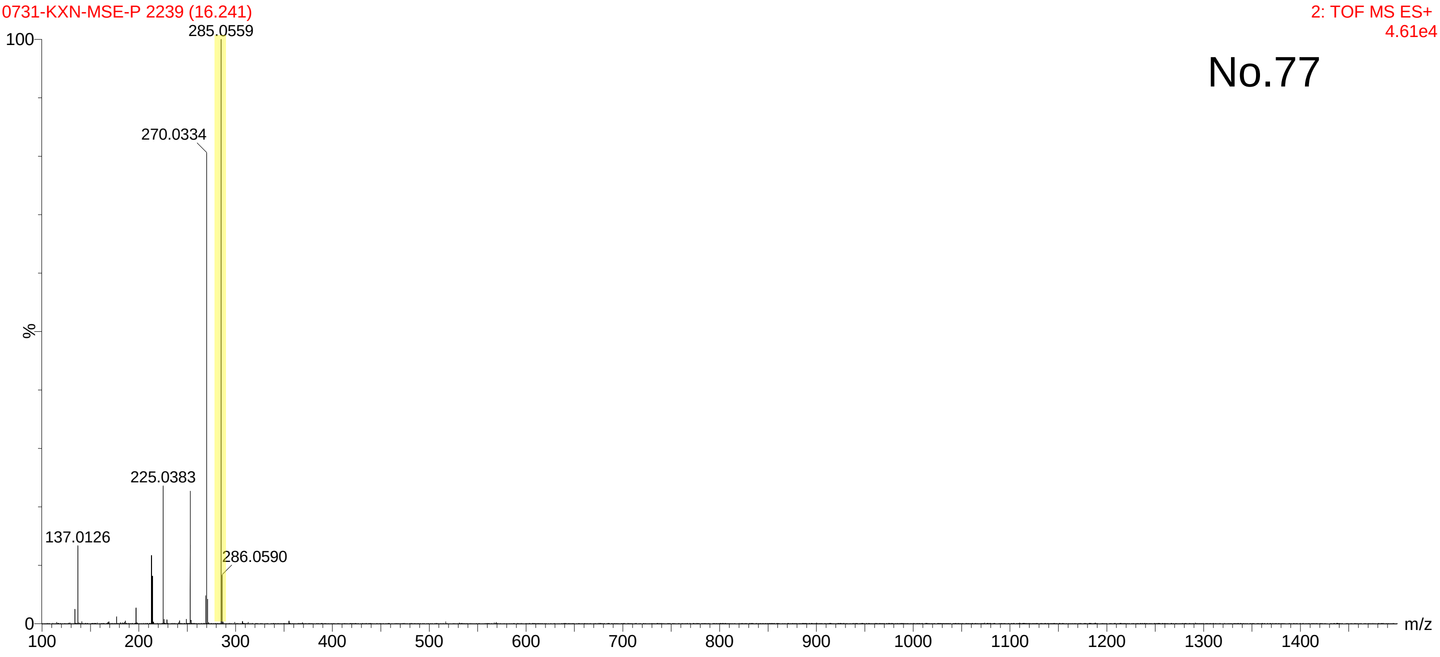
0731-KXN-MSE-P 2239 (16.241)
2: TOF MS ES+
4.61e4
No.77
285.0559
270.0334
225.0383
137.0126
286.0590
%
m/z
100
0
100 200 300 400 500 600 700 800 900 1000 1100 1200 1300 1400

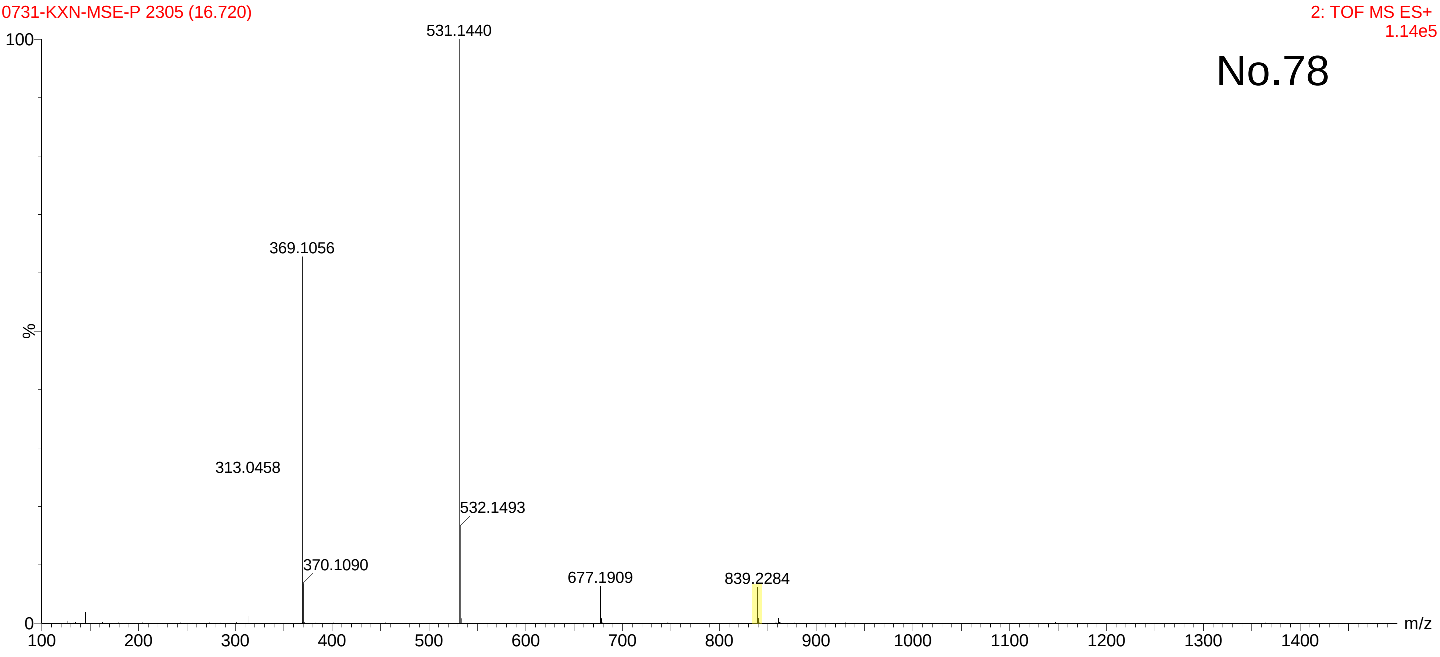
0731-KXN-MSE-P 2305 (16.720)
2: TOF MS ES+
1.14e5
No.78
531.1440
369.1056
313.0458
532.1493
370.1090
677.1909
839.2284
%
m/z
100
0
100 200 300 400 500 600 700 800 900 1000 1100 1200 1300 1400

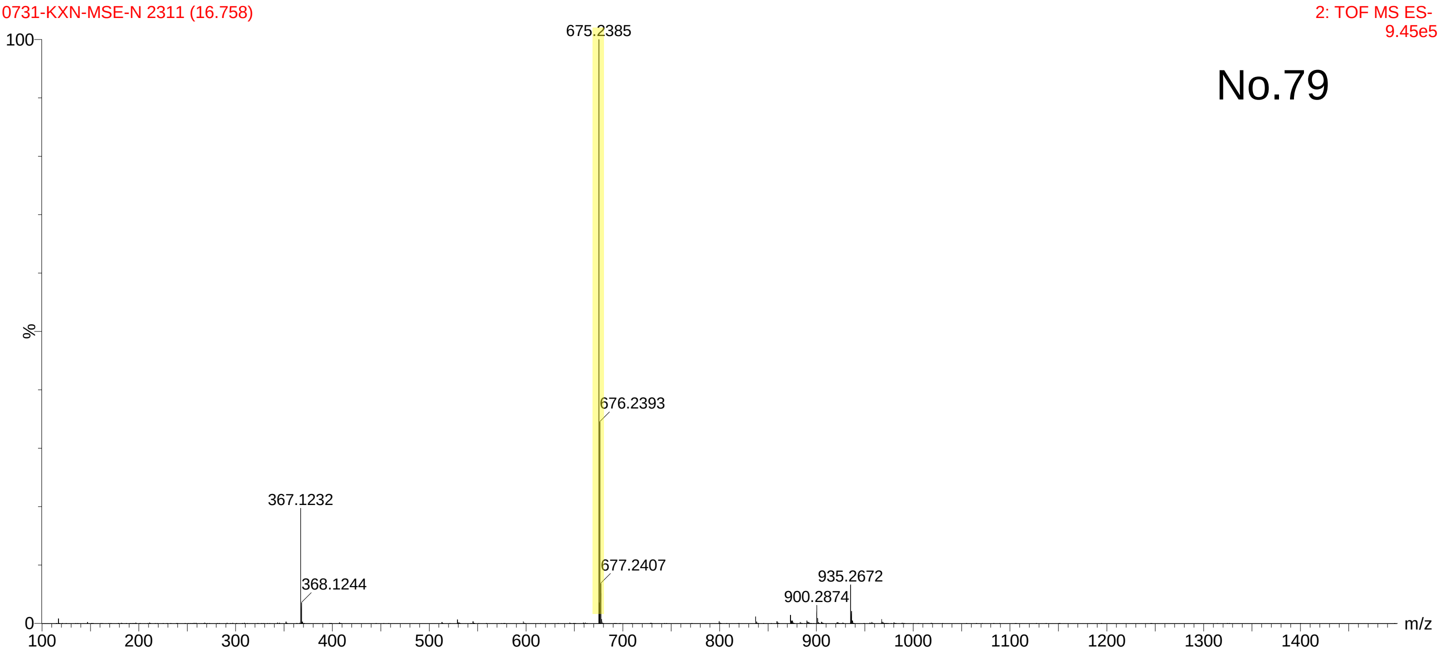
0731-KXN-MSE-N 2311 (16.758)
2: TOF MS ES-
9.45e5
No.79
675.2385
676.2393
367.1232
368.1244
677.2407
935.2672
900.2874
100
%
0
100 200 300 400 500 600 700 800 900 1000 1100 1200 1300 1400
m/z

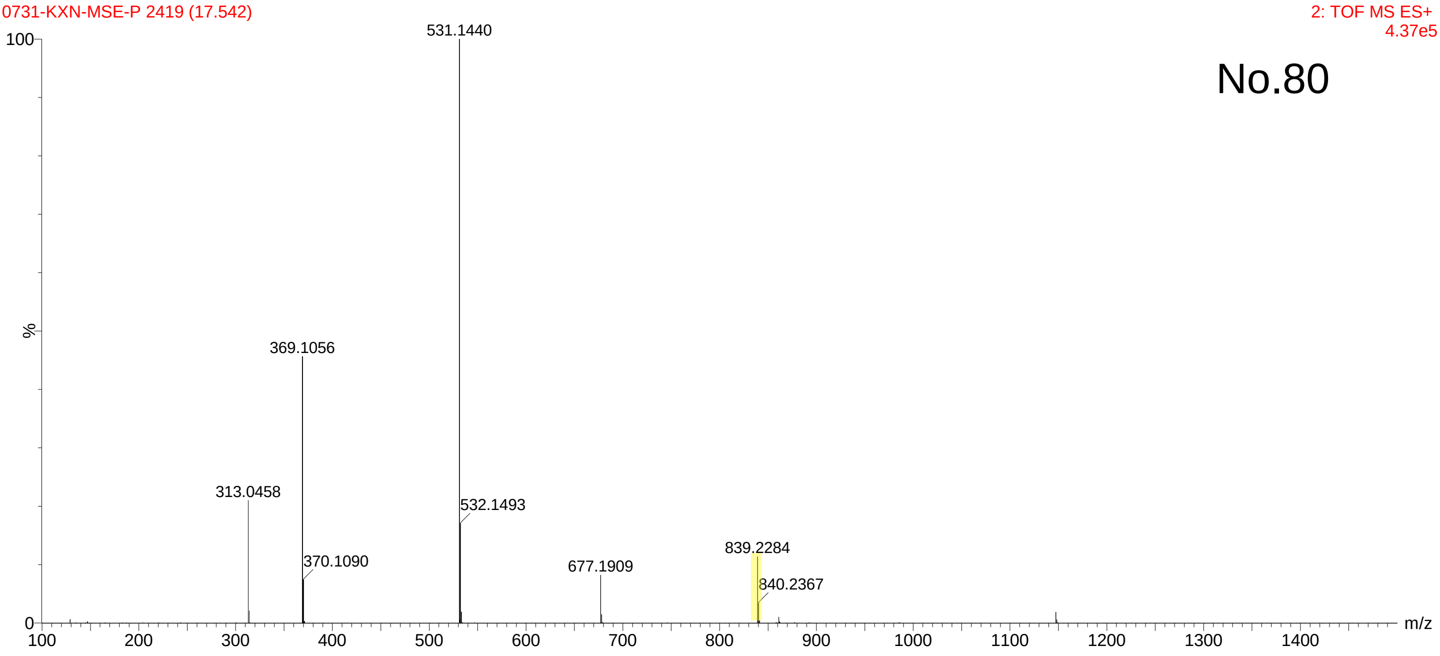
0731-KXN-MSE-P 2419 (17.542)
2: TOF MS ES+
4.37e5
No.80
531.1440
369.1056
313.0458
532.1493
370.1090
677.1909
839.2284
840.2367
100
%
0
100 200 300 400 500 600 700 800 900 1000 1100 1200 1300 1400
m/z

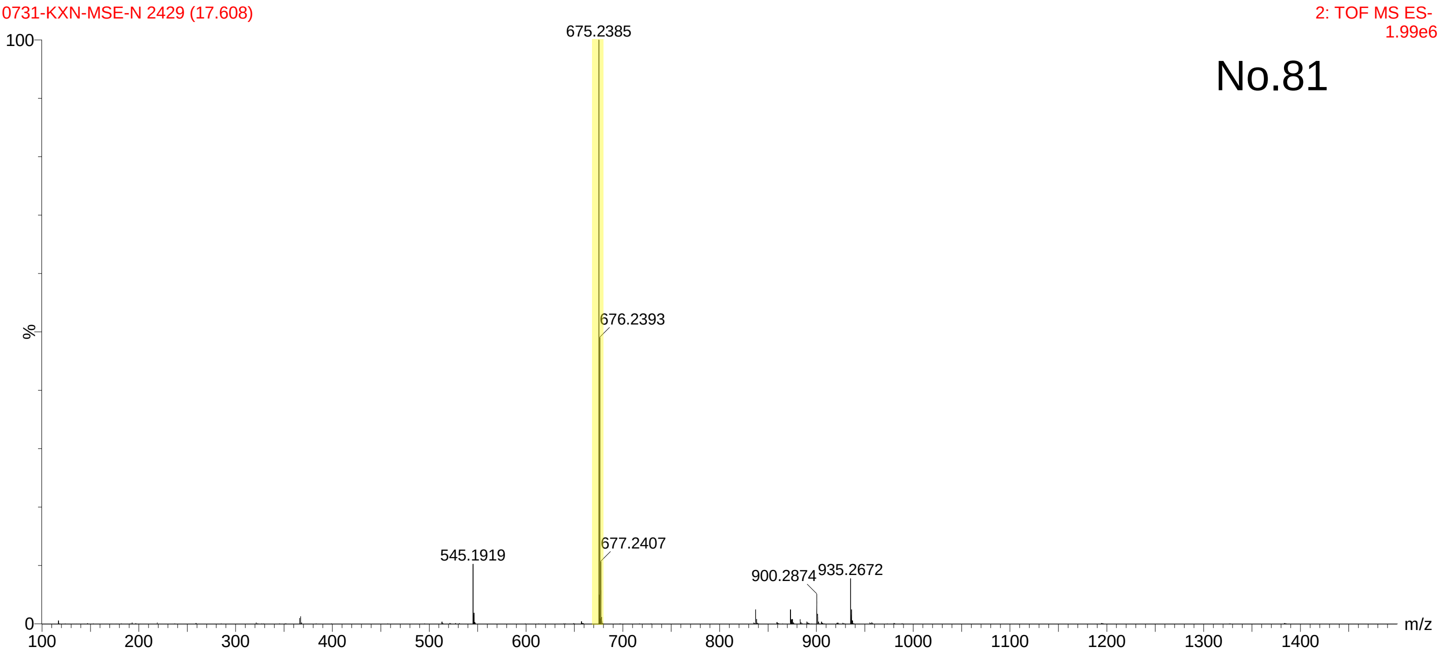
0731-KXN-MSE-N 2429 (17.608)
2: TOF MS ES-
1.99e6
No.81
675.2385
676.2393
545.1919
677.2407
900.2874
935.2672
100
%
0
100 200 300 400 500 600 700 800 900 1000 1100 1200 1300 1400
m/z

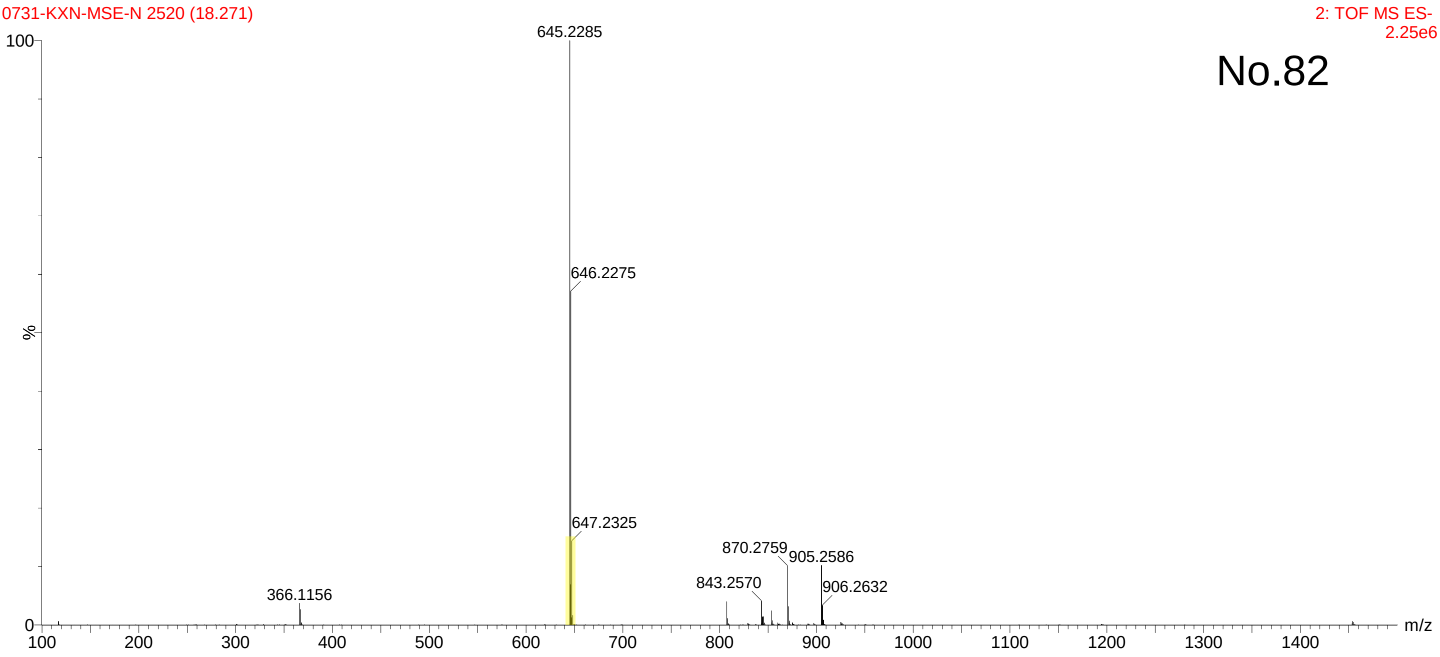
0731-KXN-MSE-N 2520 (18.271)
2: TOF MS ES-
2.25e6
No.82
100
%
0
645.2285
646.2275
647.2325
870.2759
905.2586
843.2570
906.2632
366.1156
m/z
100
200
300
400
500
600
700
800
900
1000
1100
1200
1300
1400

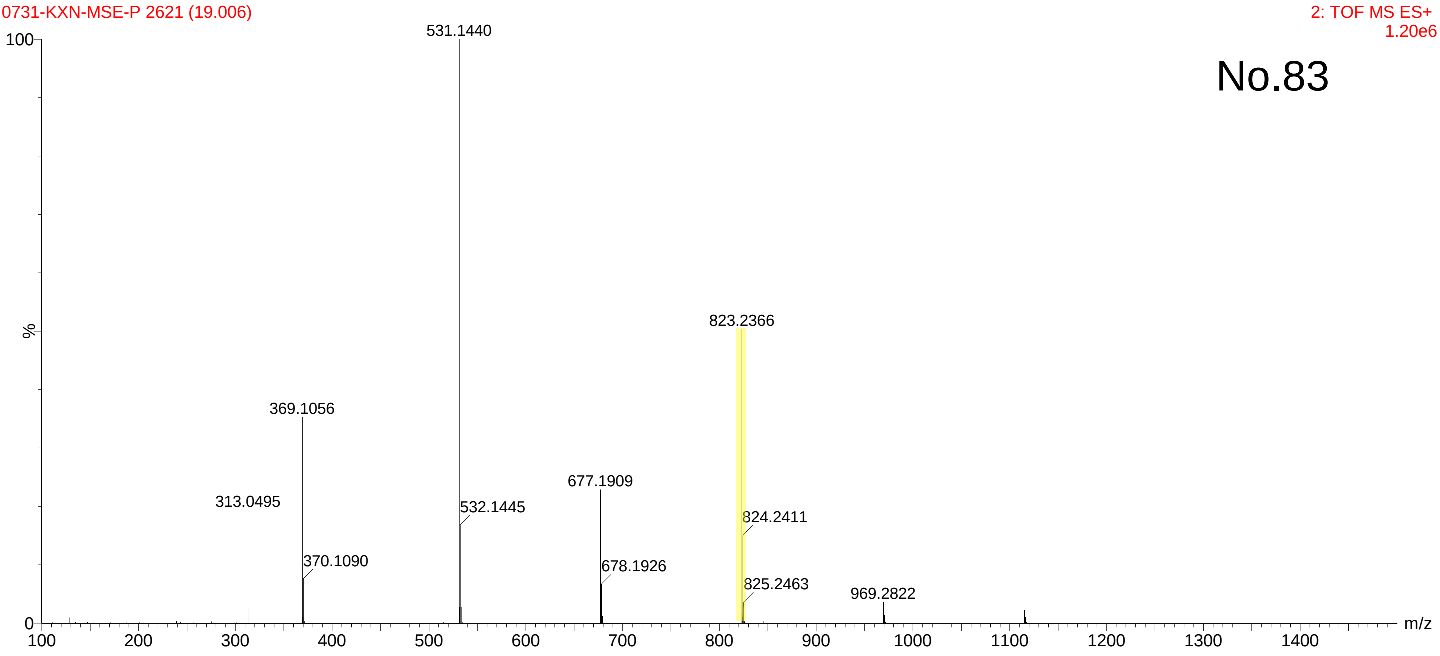
0731-KXN-MSE-P 2621 (19.006)
2: TOF MS ES+
1.20e6
No.83
100
%
0
531.1440
823.2366
369.1056
677.1909
313.0495
532.1445
824.2411
370.1090
678.1926
825.2463
969.2822
m/z
100
200
300
400
500
600
700
800
900
1000
1100
1200
1300
1400

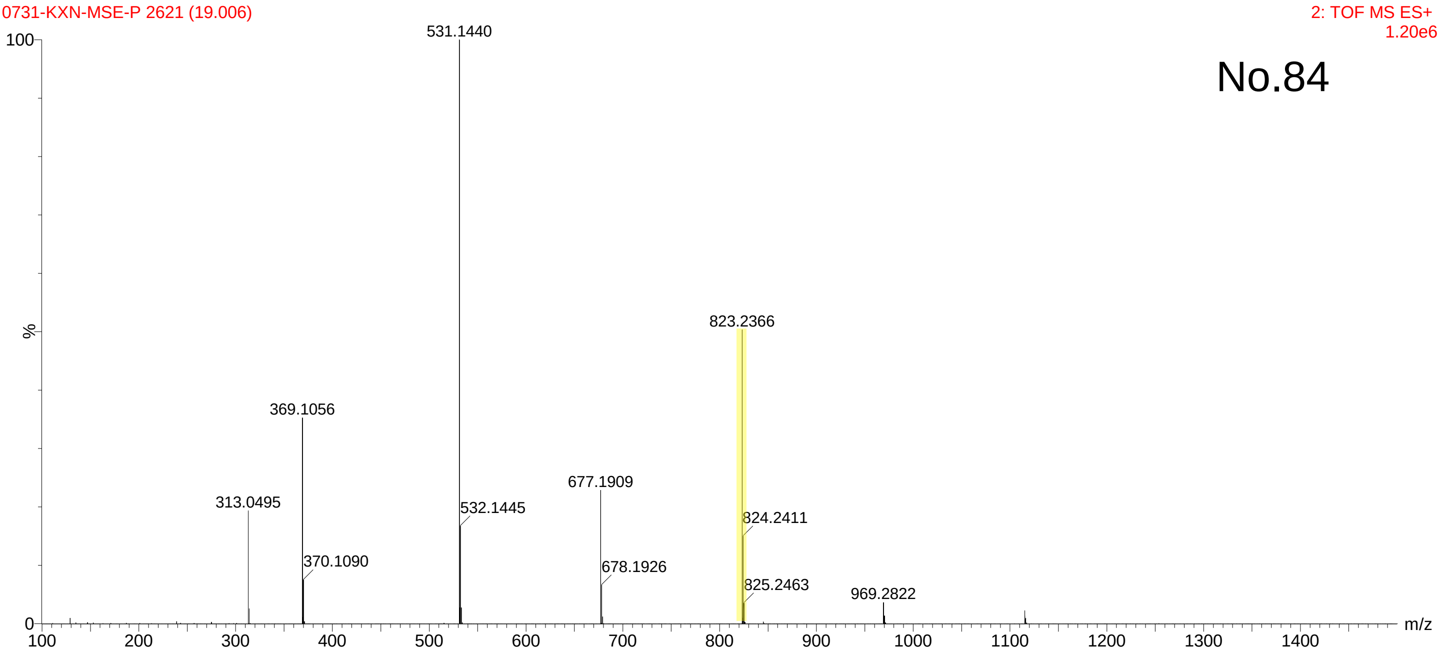
0731-KXN-MSE-P 2621 (19.006)
2: TOF MS ES+
1.20e6
No.84
100
%
0
531.1440
823.2366
369.1056
677.1909
313.0495
532.1445
824.2411
370.1090
678.1926
825.2463
969.2822
m/z
100
200
300
400
500
600
700
800
900
1000
1100
1200
1300
1400

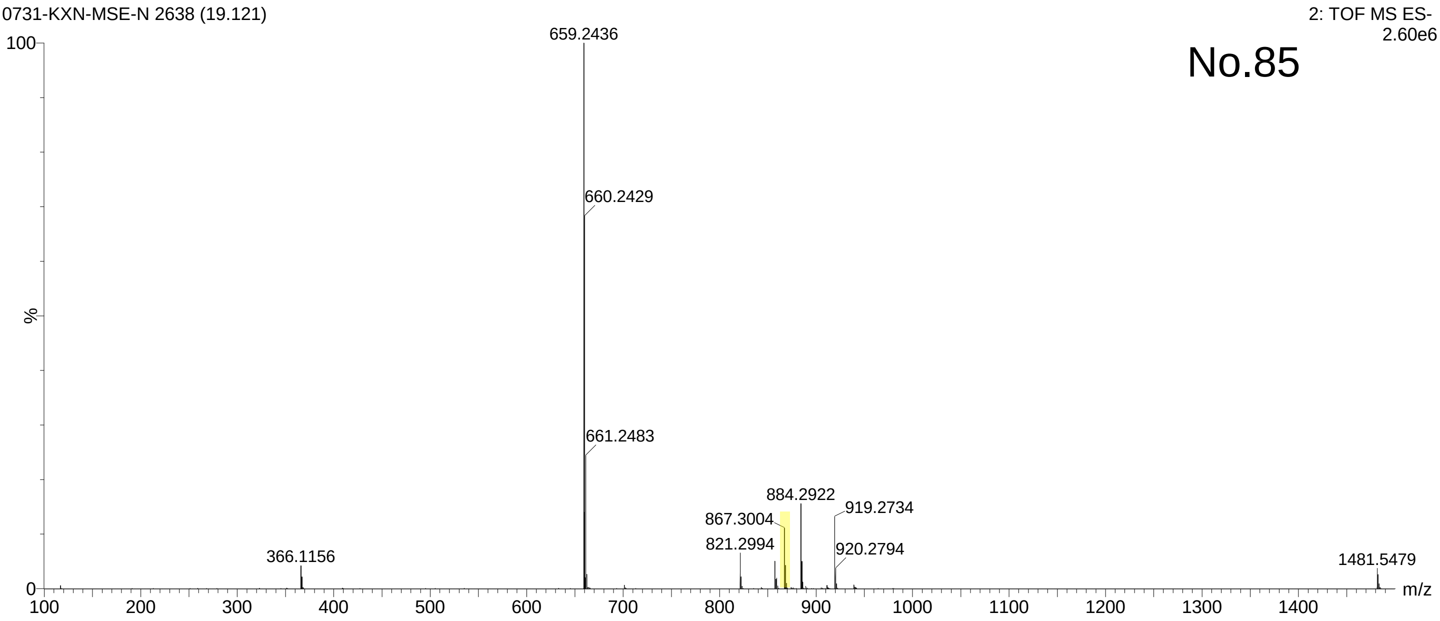
0731-KXN-MSE-N 2638 (19.121)
2: TOF MS ES-
2.60e6
No.85
659.2436
660.2429
661.2483
884.2922
919.2734
867.3004
821.2994
920.2794
366.1156
1481.5479
100
%
0
m/z
100 200 300 400 500 600 700 800 900 1000 1100 1200 1300 1400

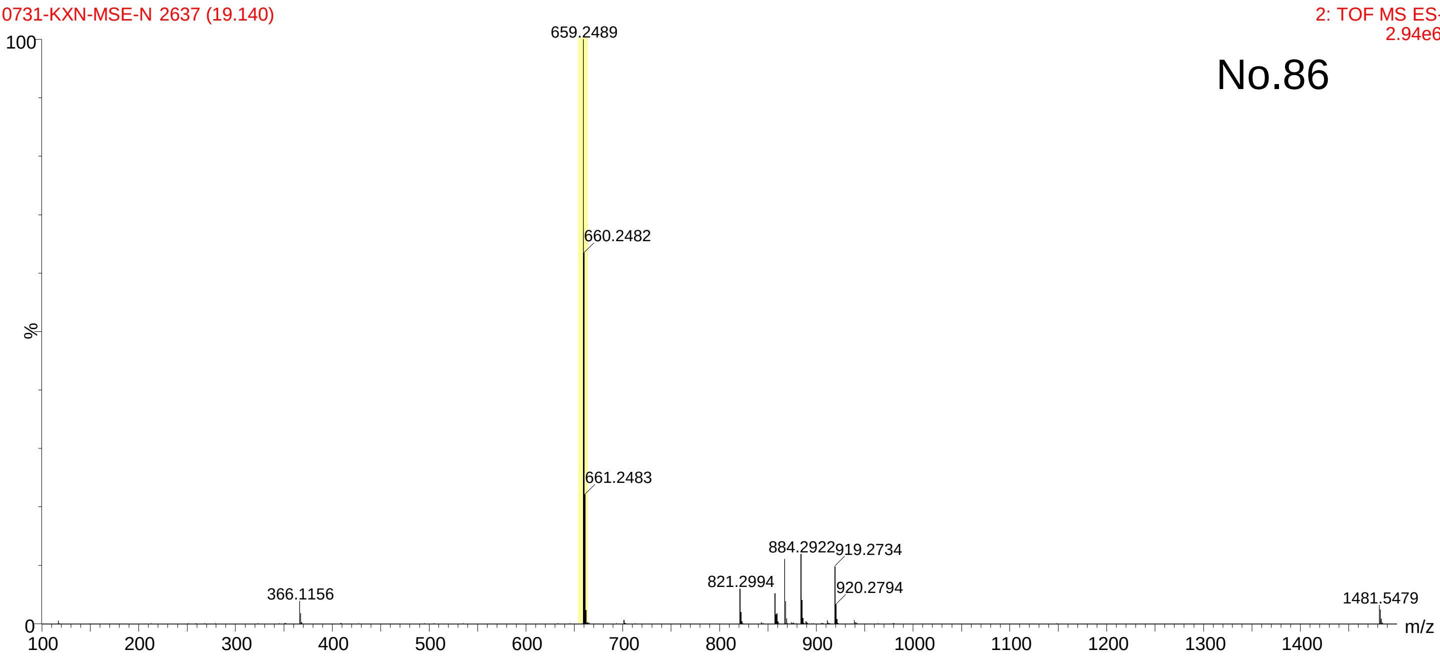
0731-KXN-MSE-N 2637 (19.140)
2: TOF MS ES-
2.94e6
No.86
659.2489
660.2482
661.2483
884.2922
919.2734
821.2994
920.2794
366.1156
1481.5479
100
%
0
m/z
100 200 300 400 500 600 700 800 900 1000 1100 1200 1300 1400

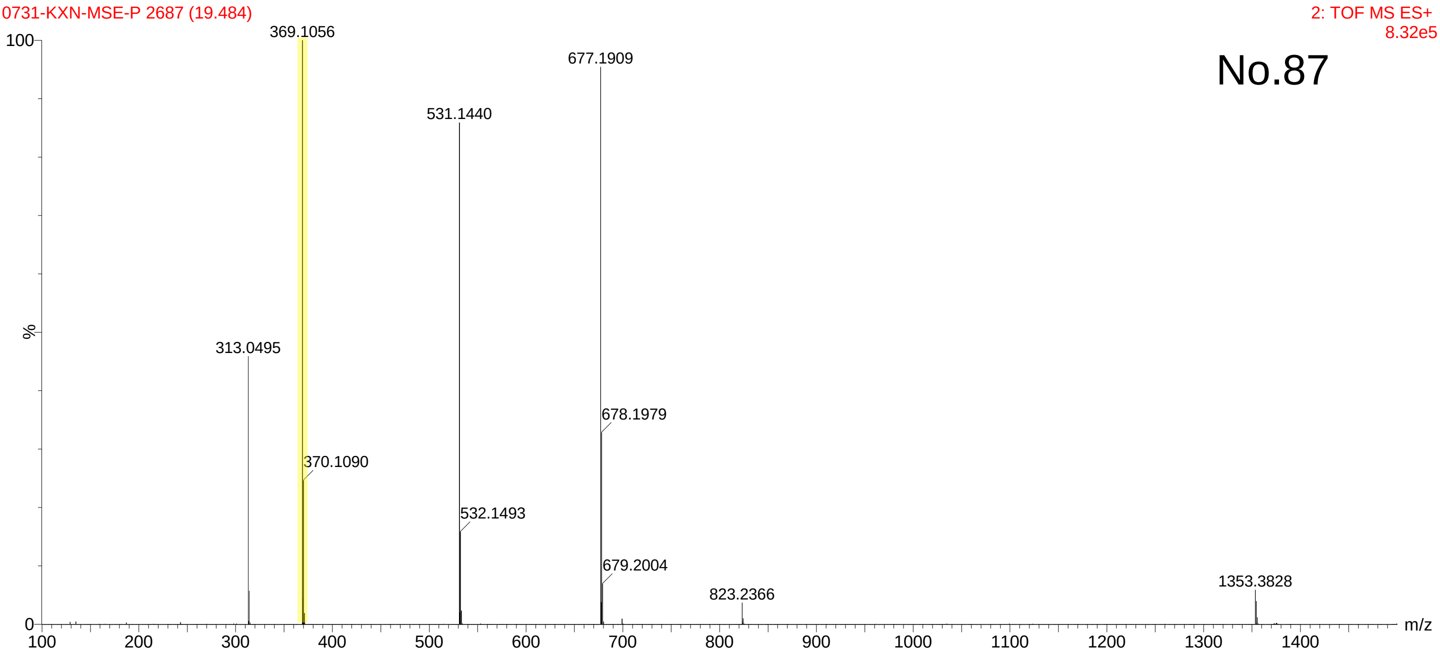
0731-KXN-MSE-P 2687 (19.484)
2: TOF MS ES+
8.32e5
No.87
369.1056
677.1909
531.1440
313.0495
678.1979
370.1090
532.1493
679.2004
823.2366
1353.3828
100
%
0
m/z
100 200 300 400 500 600 700 800 900 1000 1100 1200 1300 1400

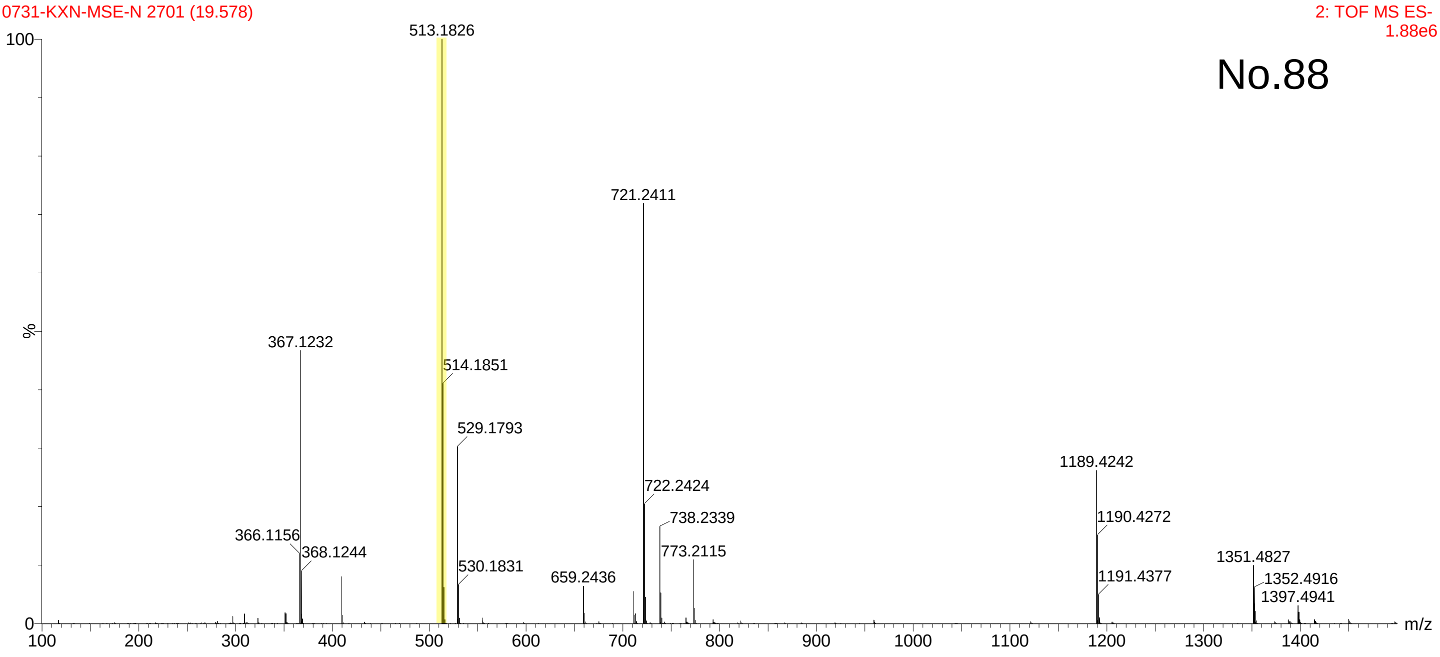
0731-KXN-MSE-N 2701 (19.578)
2: TOF MS ES-
1.88e6
No.88
513.1826
721.2411
367.1232
514.1851
529.1793
1189.4242
722.2424
738.2339
366.1156
1190.4272
368.1244
773.2115
1351.4827
530.1831
1191.4377
659.2436
1352.4916
1397.4941
%
m/z

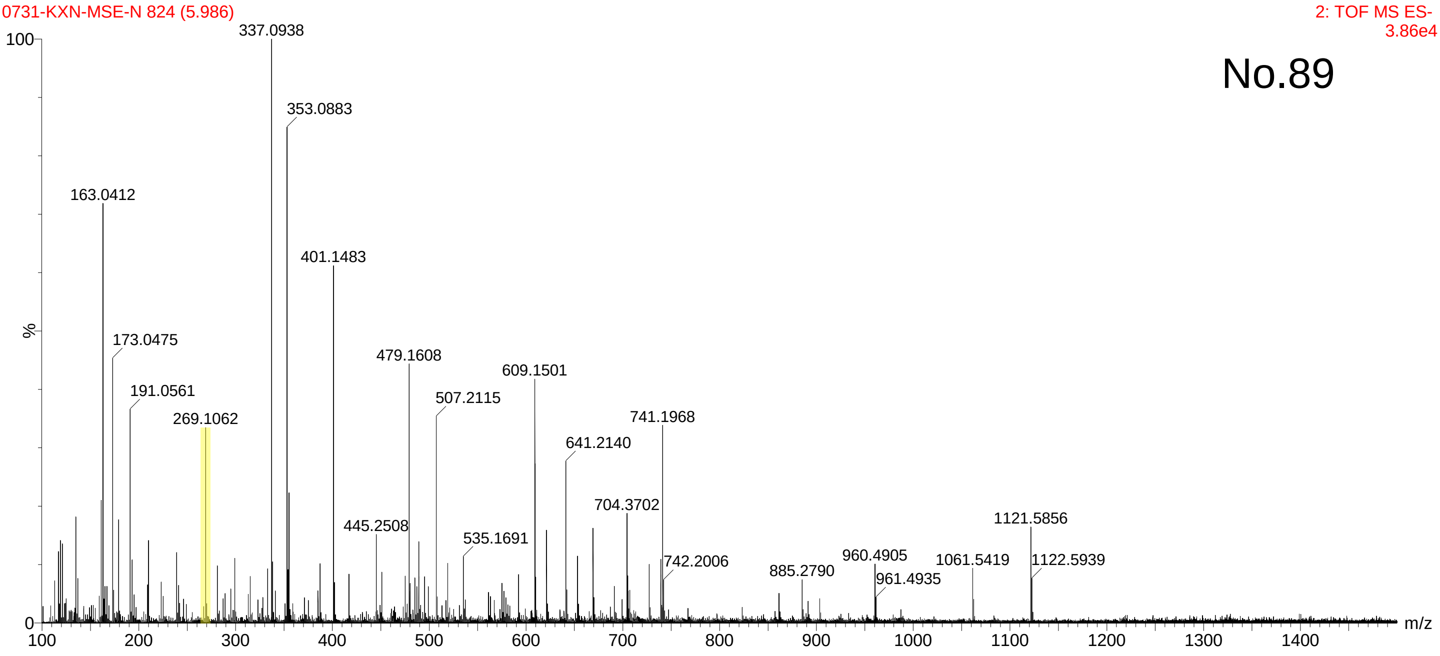
0731-KXN-MSE-N 824 (5.986)
2: TOF MS ES-
3.86e4
No.89
337.0938
353.0883
163.0412
401.1483
173.0475
479.1608
609.1501
191.0561
507.2115
269.1062
741.1968
641.2140
704.3702
445.2508
1121.5856
535.1691
742.2006
960.4905
1061.5419
1122.5939
885.2790
961.4935
%
m/z

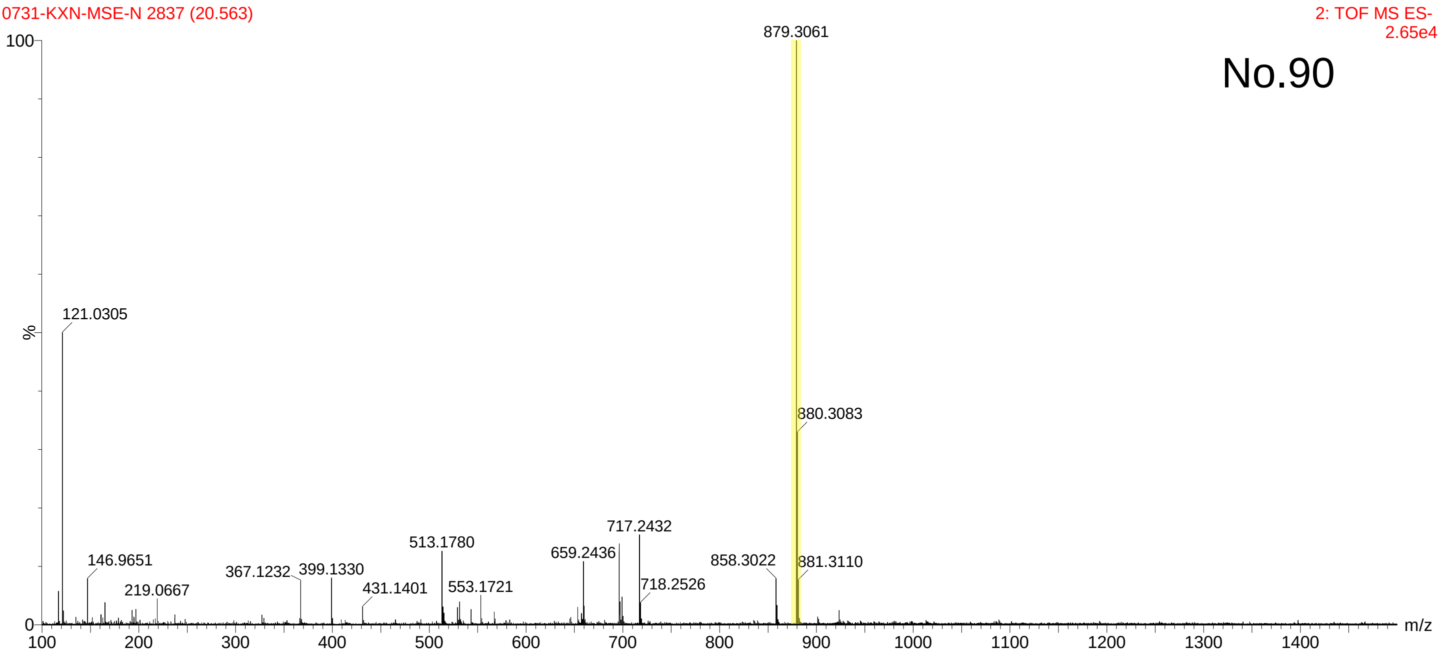
0731-KXN-MSE-N 2837 (20.563)
2: TOF MS ES-
2.65e4
879.3061
No.90
121.0305
880.3083
717.2432
513.1780
659.2436
146.9651
858.3022
881.3110
367.1232
399.1330
718.2526
219.0667
431.1401
553.1721
%
m/z

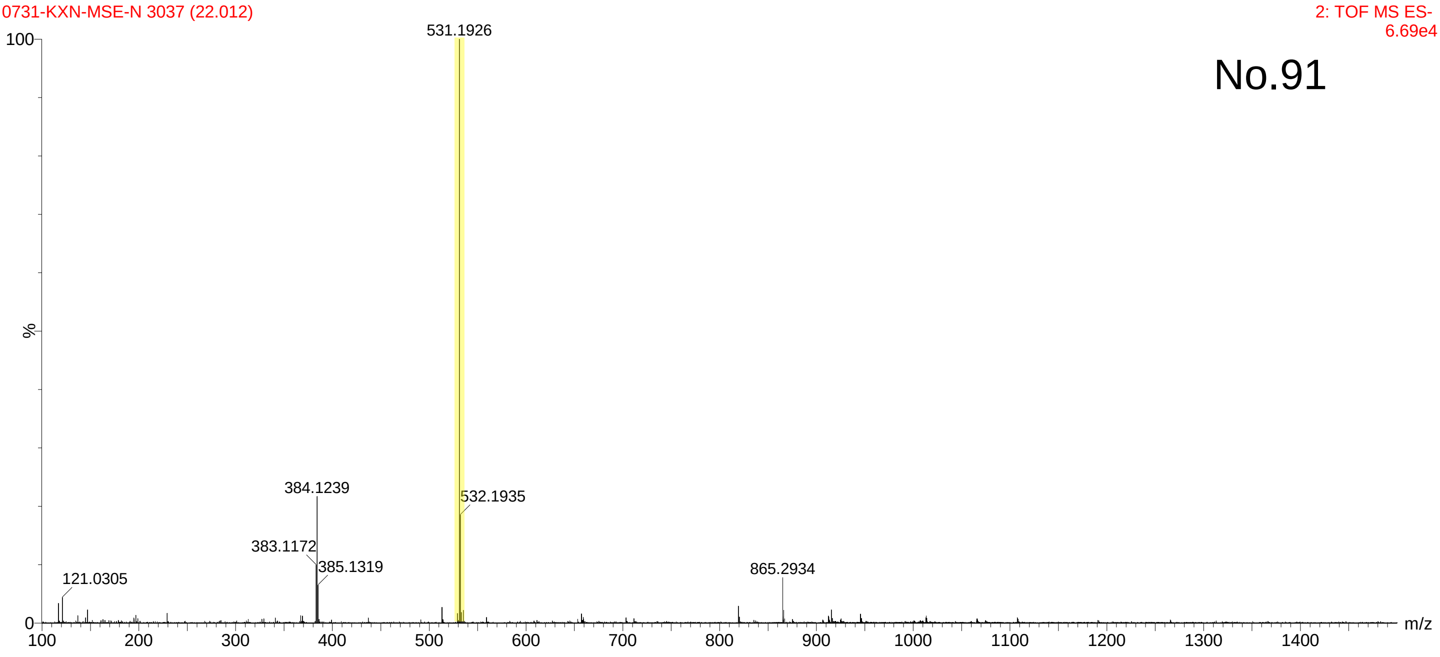

0731-KXN-MSE-N 3037 (22.012)
2: TOF MS ES-
6.69e4
No.91
531.1926
384.1239
532.1935
383.1172
385.1319
121.0305
865.2934
%
m/z

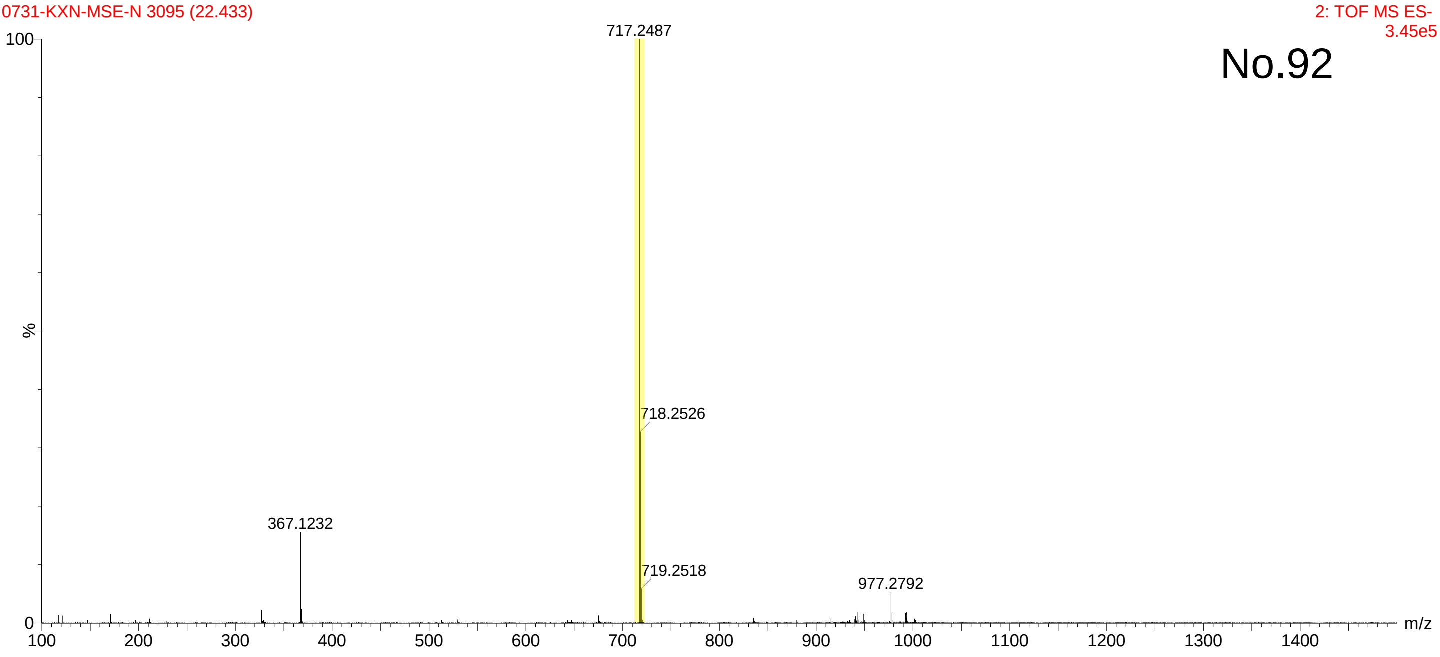

0731-KXN-MSE-N 3095 (22.433)
2: TOF MS ES-
3.45e5
No.92
717.2487
718.2526
367.1232
719.2518
977.2792
%
m/z

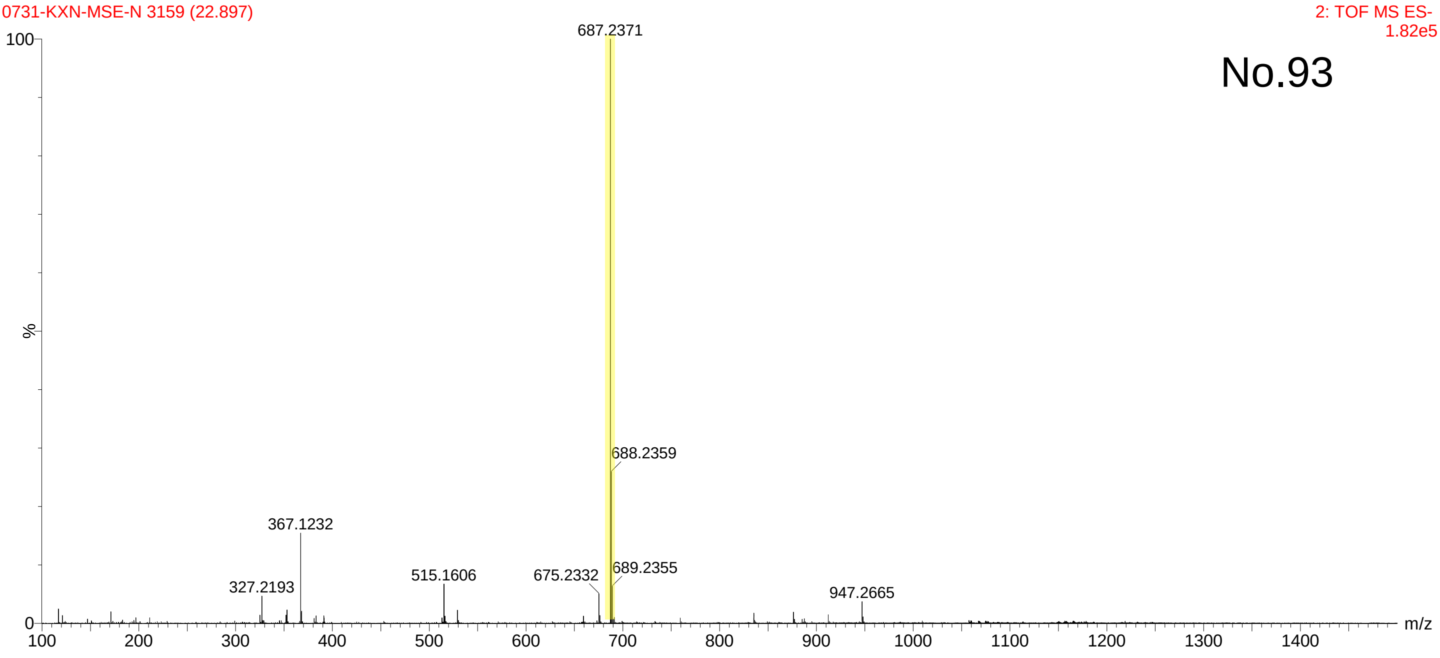

0731-KXN-MSE-N 3159 (22.897)
2: TOF MS ES-
1.82e5
No.93
687.2371
688.2359
367.1232
515.1606
675.2332
689.2355
327.2193
947.2665
%
m/z

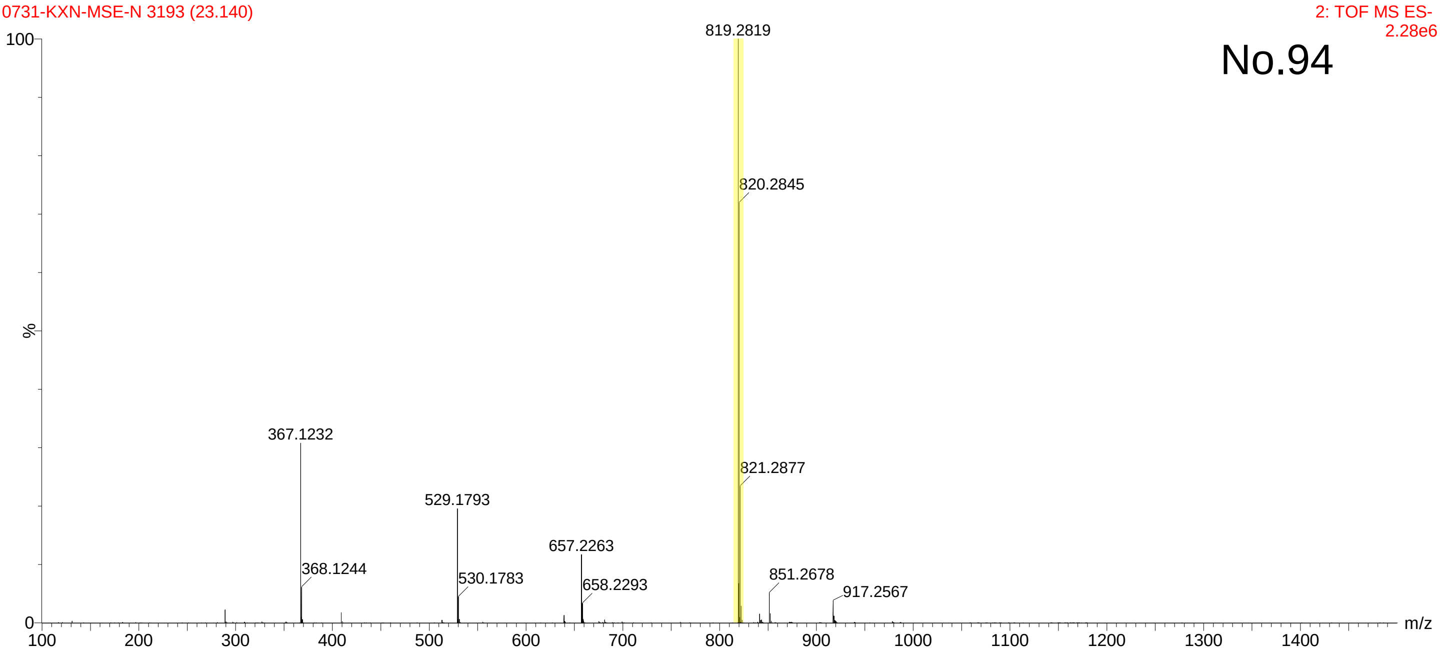

0731-KXN-MSE-N 3193 (23.140)
2: TOF MS ES-
2.28e6
No.94
819.2819
820.2845
367.1232
821.2877
529.1793
657.2263
368.1244
530.1783
851.2678
658.2293
917.2567
%
m/z

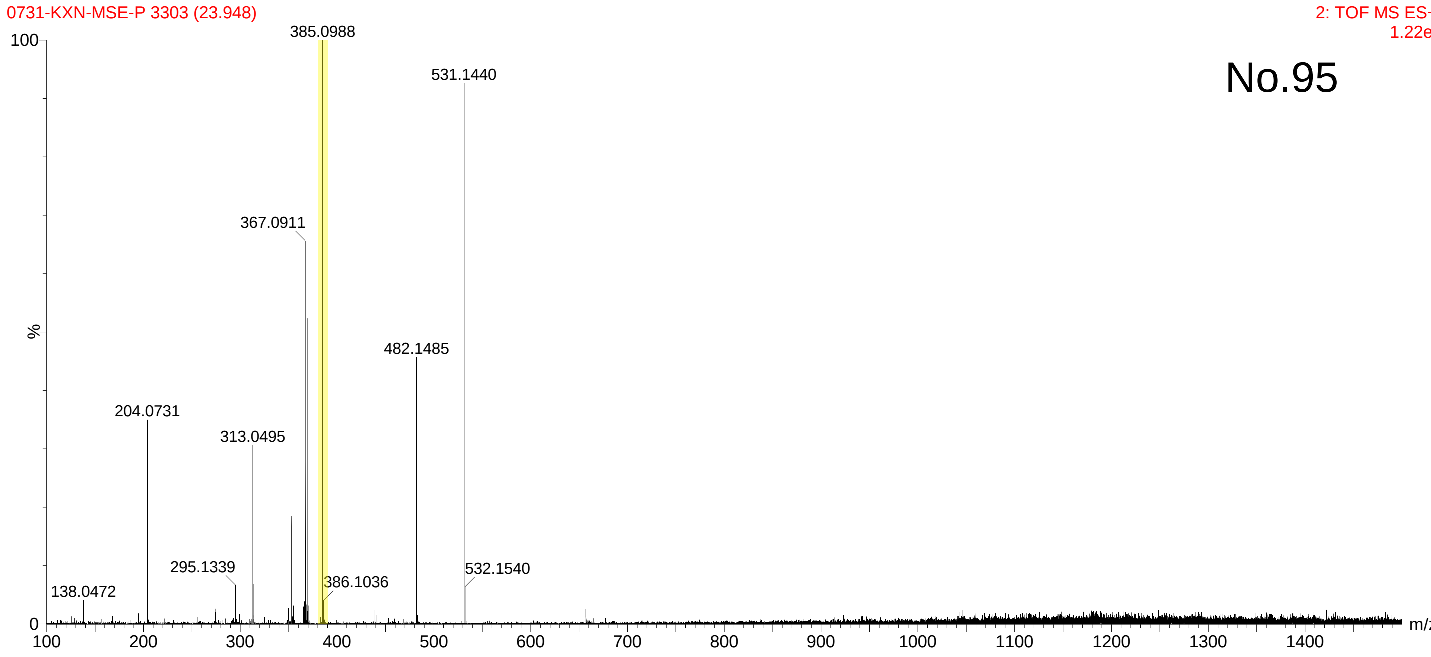

0731-KXN-MSE-P 3303 (23.948)
2: TOF MS ES
1.22e
No.95
385.0988
531.1440
367.0911
482.1485
204.0731
313.0495
295.1339
386.1036
532.1540
138.0472
%

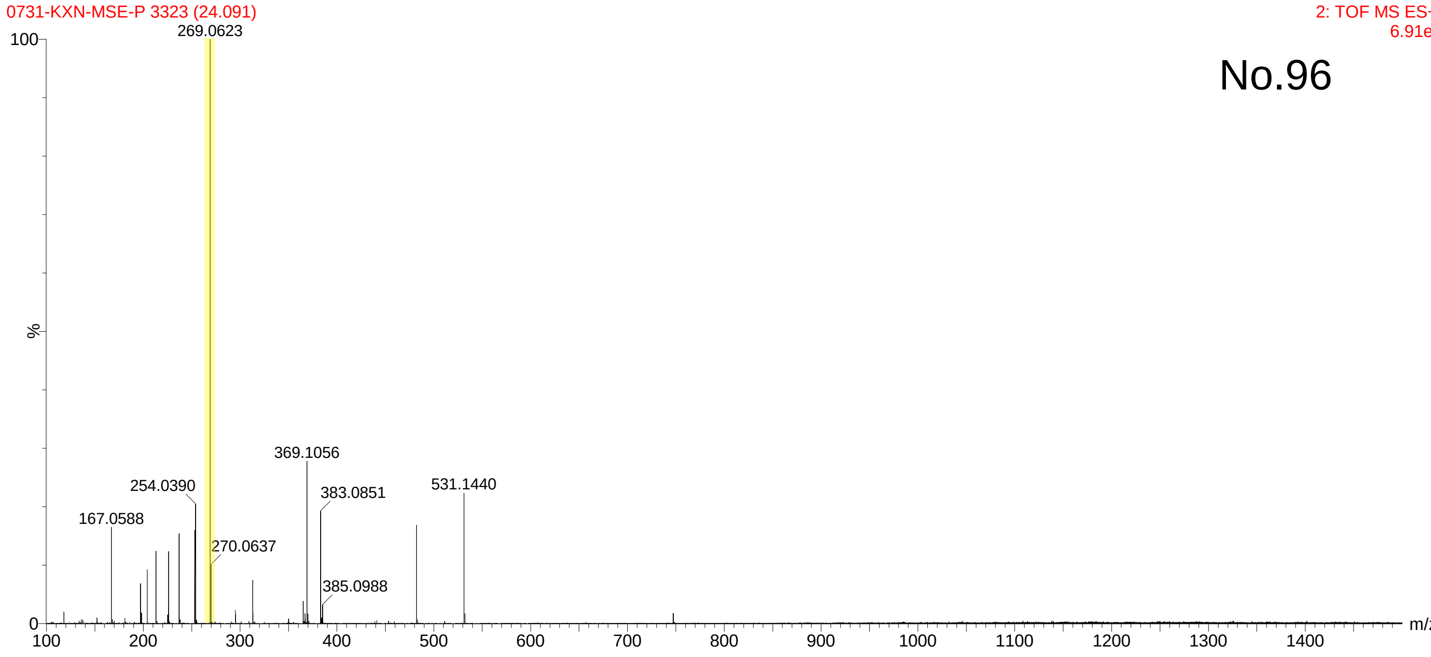

0731-KXN-MSE-P 3323 (24.091)
2: TOF MS ES
6.91e
No.96
269.0623
369.1056
254.0390
383.0851
531.1440
167.0588
270.0637
385.0988
%

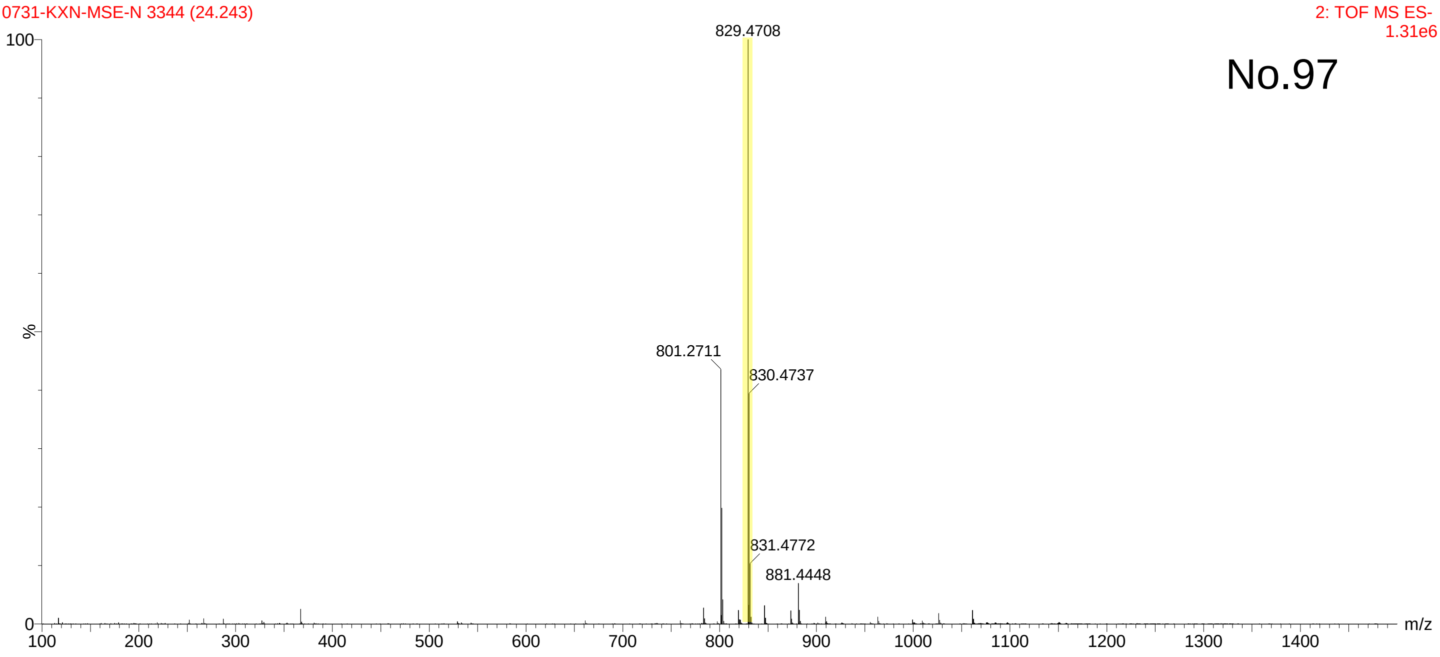

0731-KXN-MSE-N 3344 (24.243)
2: TOF MS ES-
1.31e6
No.97
829.4708
801.2711
830.4737
831.4772
881.4448
100
%
0
m/z
100
200
300
400
500
600
700
800
900
1000
1100
1200
1300
1400

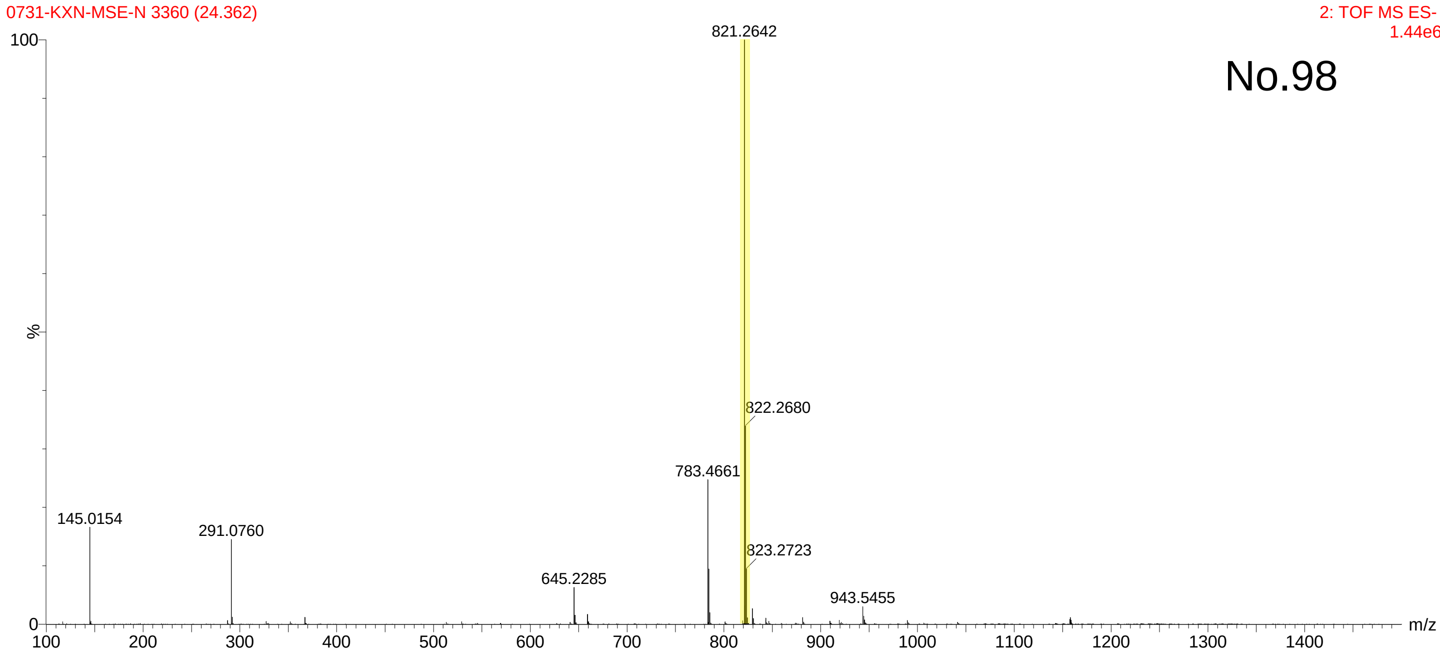

0731-KXN-MSE-N 3360 (24.362)
2: TOF MS ES-
1.44e6
No.98
821.2642
822.2680
783.4661
145.0154
291.0760
823.2723
645.2285
943.5455
100
%
0
m/z
100
200
300
400
500
600
700
800
900
1000
1100
1200
1300
1400

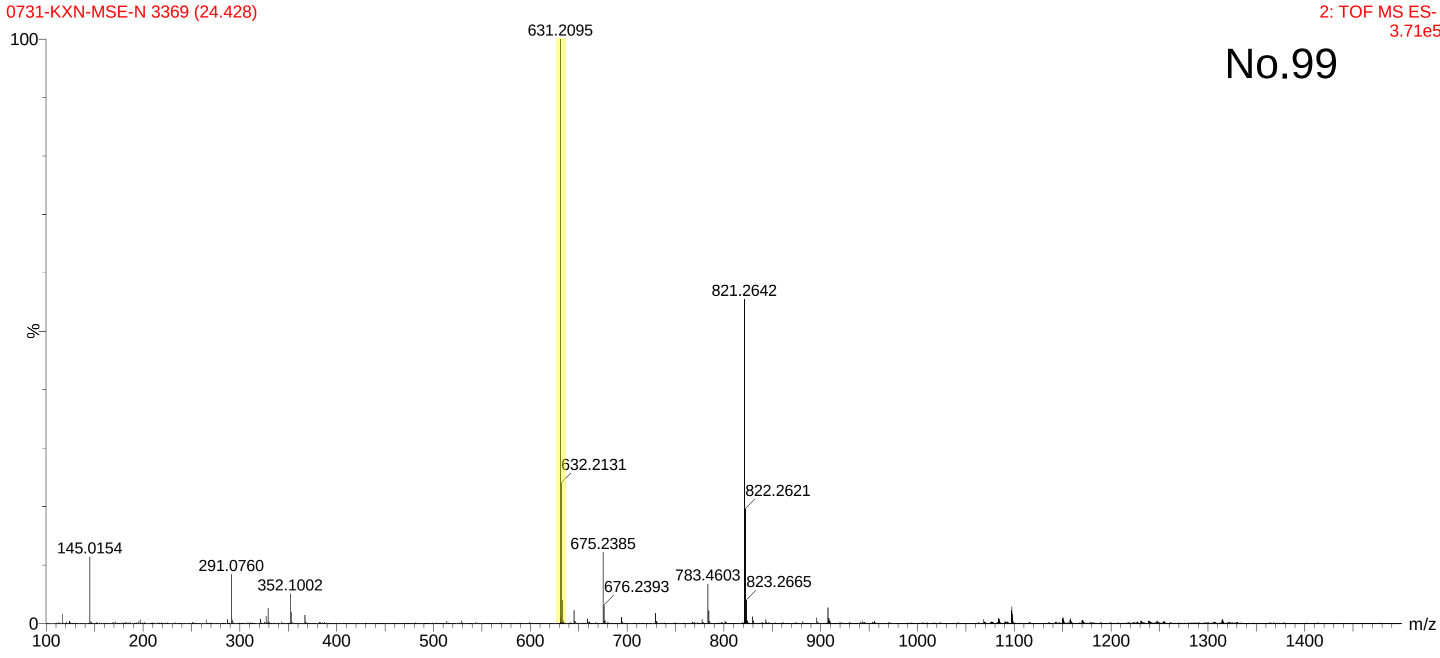

0731-KXN-MSE-N 3369 (24.428)
2: TOF MS ES-
3.71e5
No.99
631.2095
821.2642
632.2131
822.2621
145.0154
675.2385
291.0760
352.1002
676.2393
783.4603
823.2665
100
%
0
m/z
100
200
300
400
500
600
700
800
900
1000
1100
1200
1300
1400

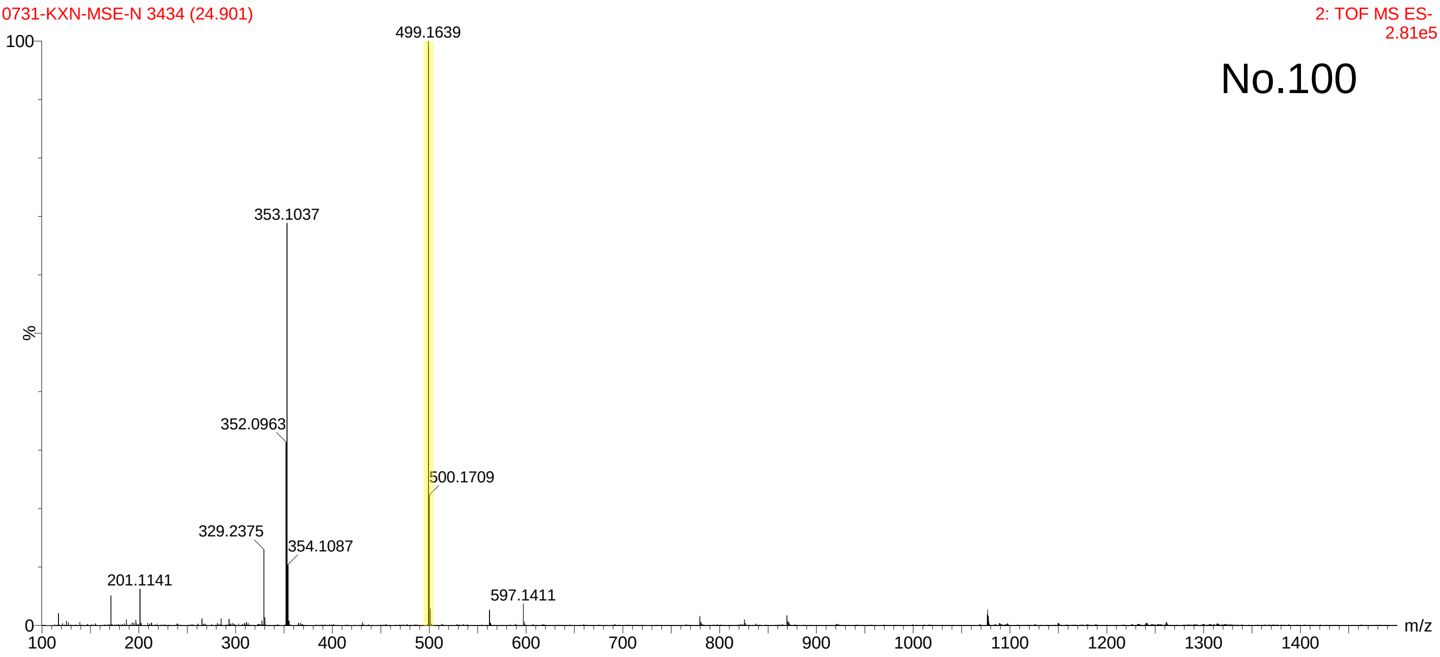
0731-KXN-MSE-N 3434 (24.901)
2: TOF MS ES-
2.81e5
No.100
499.1639
353.1037
352.0963
500.1709
329.2375
354.1087
201.1141
597.1411
100
%
0
m/z
100
200
300
400
500
600
700
800
900
1000
1100
1200
1300
1400

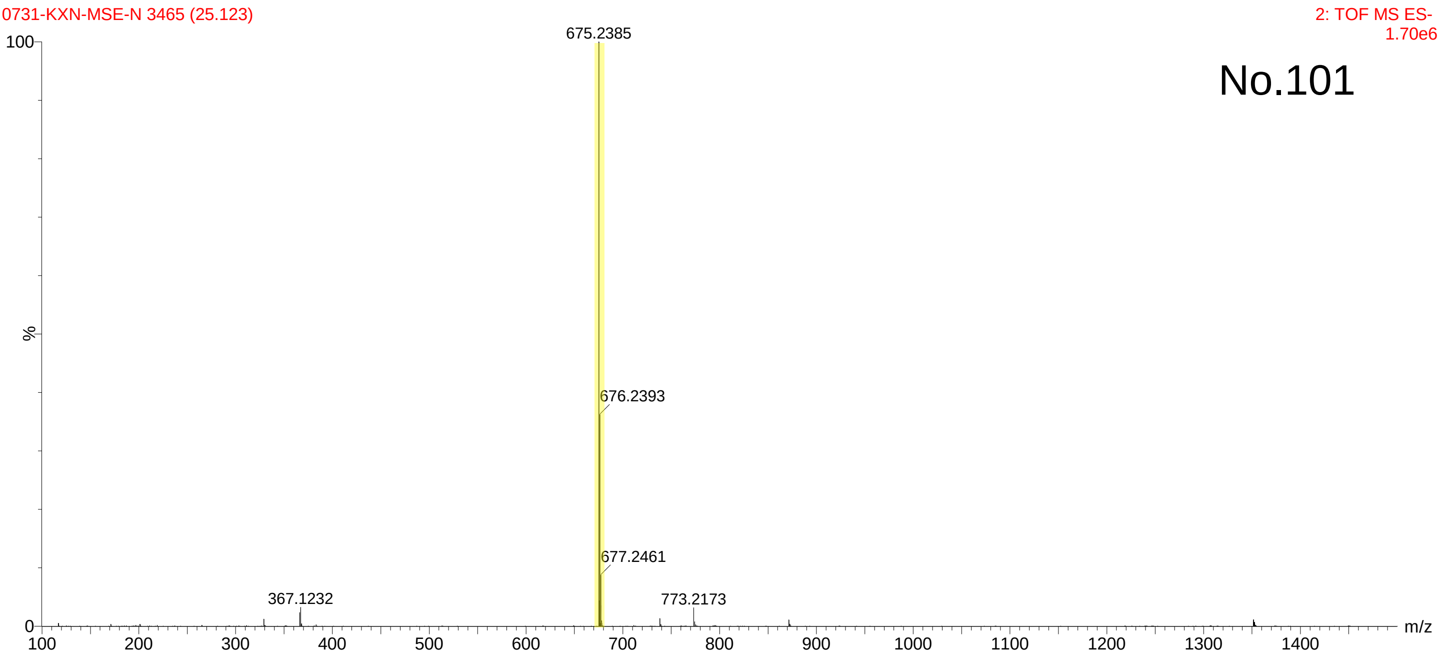
0731-KXN-MSE-N 3465 (25.123)
2: TOF MS ES-
1.70e6
No.101
675.2385
676.2393
677.2461
367.1232
773.2173
100
%
0
m/z
100
200
300
400
500
600
700
800
900
1000
1100
1200
1300
1400

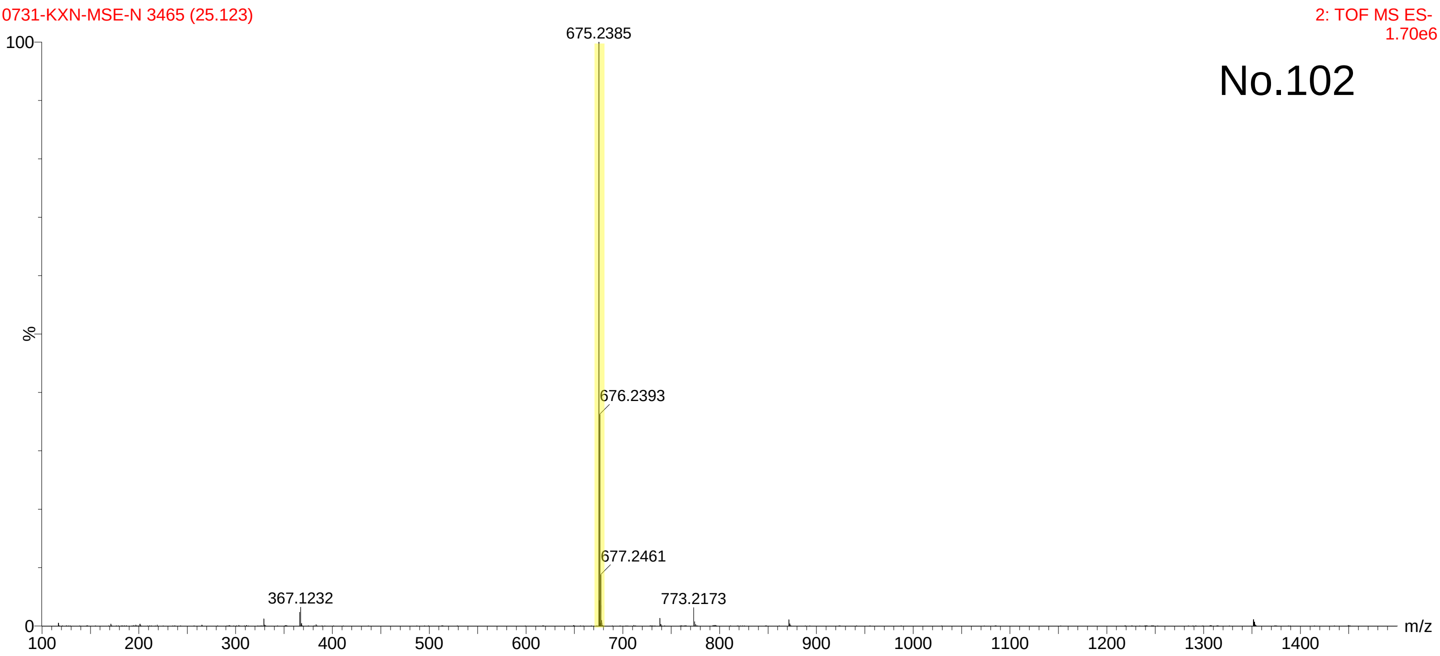
0731-KXN-MSE-N 3465 (25.123)
2: TOF MS ES-
1.70e6
No.102
675.2385
676.2393
677.2461
367.1232
773.2173
100
%
0
m/z
100
200
300
400
500
600
700
800
900
1000
1100
1200
1300
1400

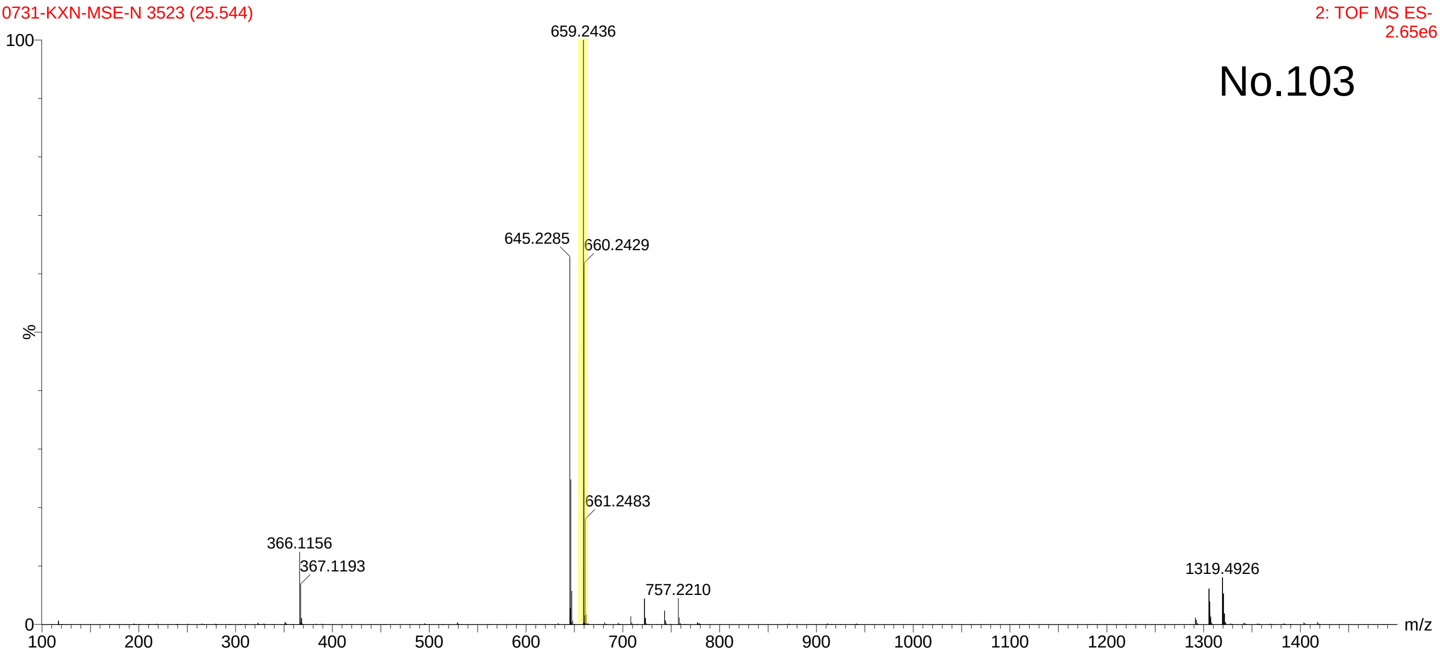
0731-KXN-MSE-N 3523 (25.544)
2: TOF MS ES-
2.65e6
No.103
659.2436
645.2285
660.2429
661.2483
366.1156
367.1193
757.2210
1319.4926
%
m/z

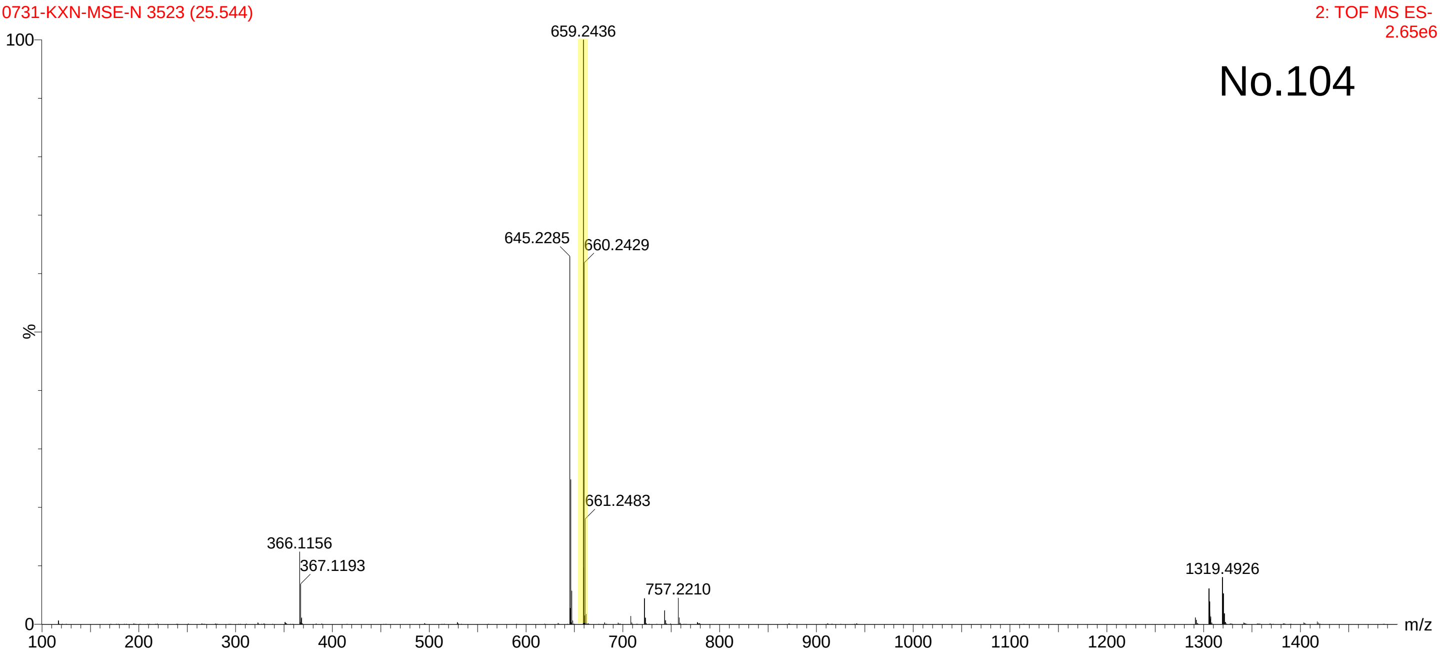
0731-KXN-MSE-N 3523 (25.544)
2: TOF MS ES-
2.65e6
No.104
659.2436
645.2285
660.2429
661.2483
366.1156
367.1193
757.2210
1319.4926
%
m/z

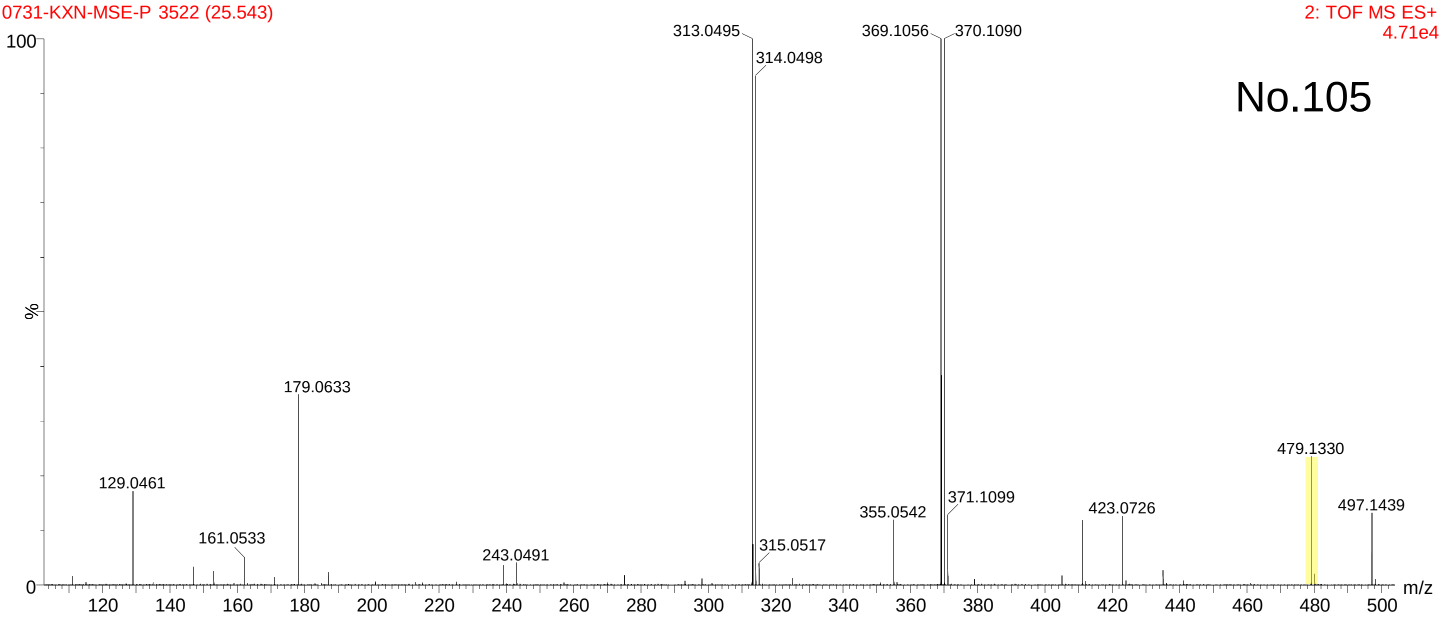
0731-KXN-MSE-P 3522 (25.543)
2: TOF MS ES+
4.71e4
No.105
313.0495
314.0498
369.1056
370.1090
179.0633
129.0461
161.0533
243.0491
315.0517
355.0542
371.1099
423.0726
479.1330
497.1439
%
m/z

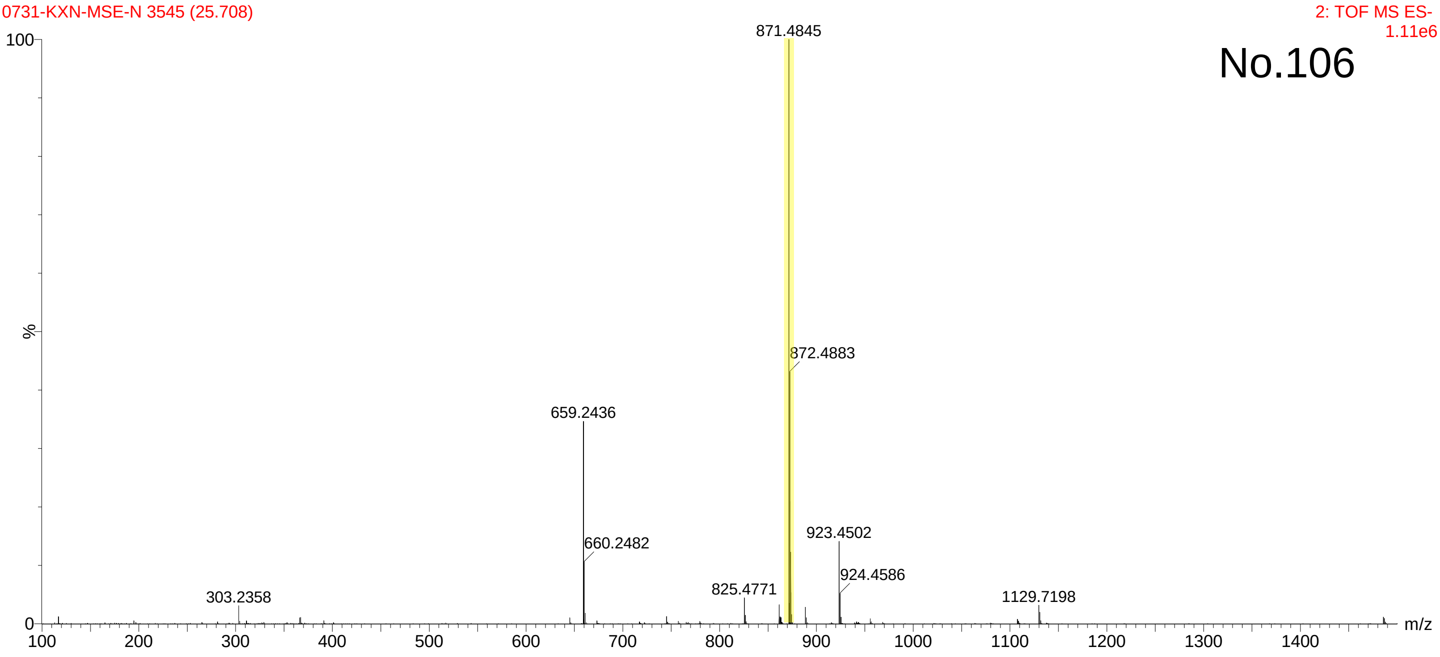

0731-KXN-MSE-N 3545 (25.708)
2: TOF MS ES-
1.11e6
No.106
100
%
0
871.4845
872.4883
659.2436
660.2482
923.4502
924.4586
825.4771
303.2358
1129.7198
100
200
300
400
500
600
700
800
900
1000
1100
1200
1300
1400
m/z

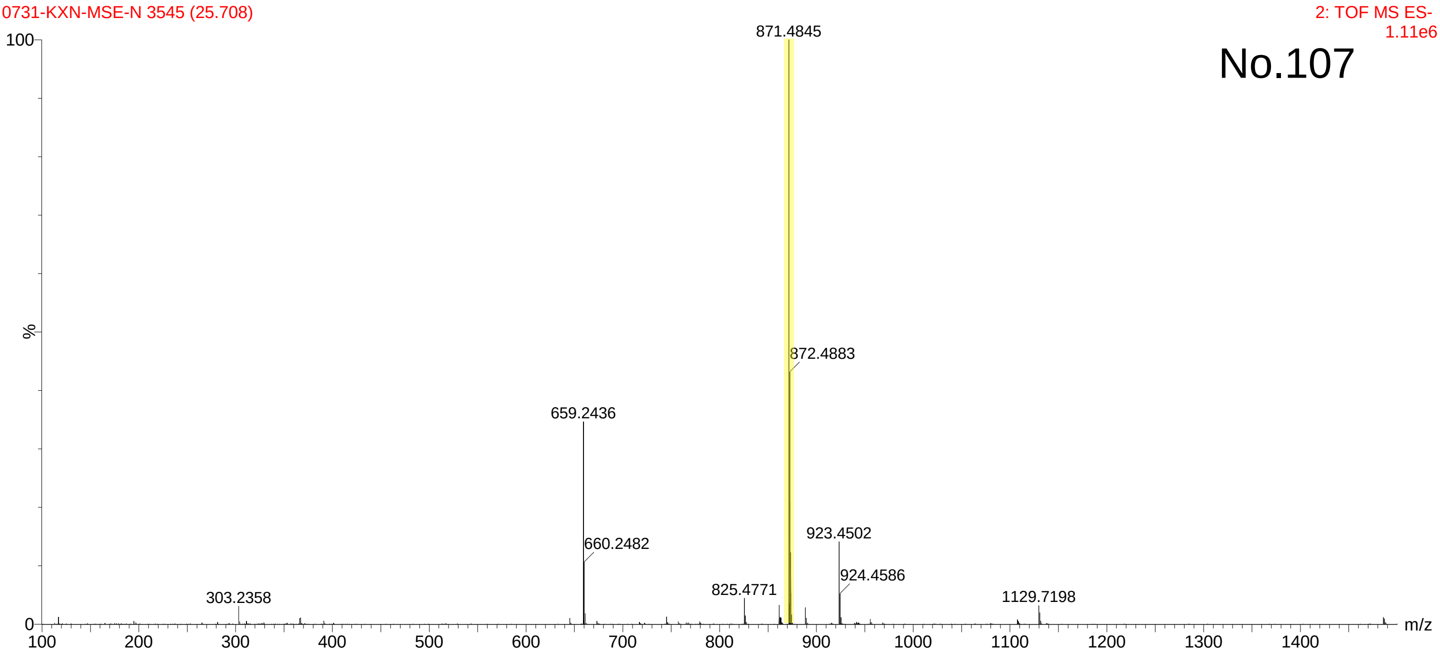

0731-KXN-MSE-N 3545 (25.708)
2: TOF MS ES-
1.11e6
No.107
100
%
0
871.4845
872.4883
659.2436
660.2482
923.4502
924.4586
825.4771
303.2358
1129.7198
100
200
300
400
500
600
700
800
900
1000
1100
1200
1300
1400
m/z

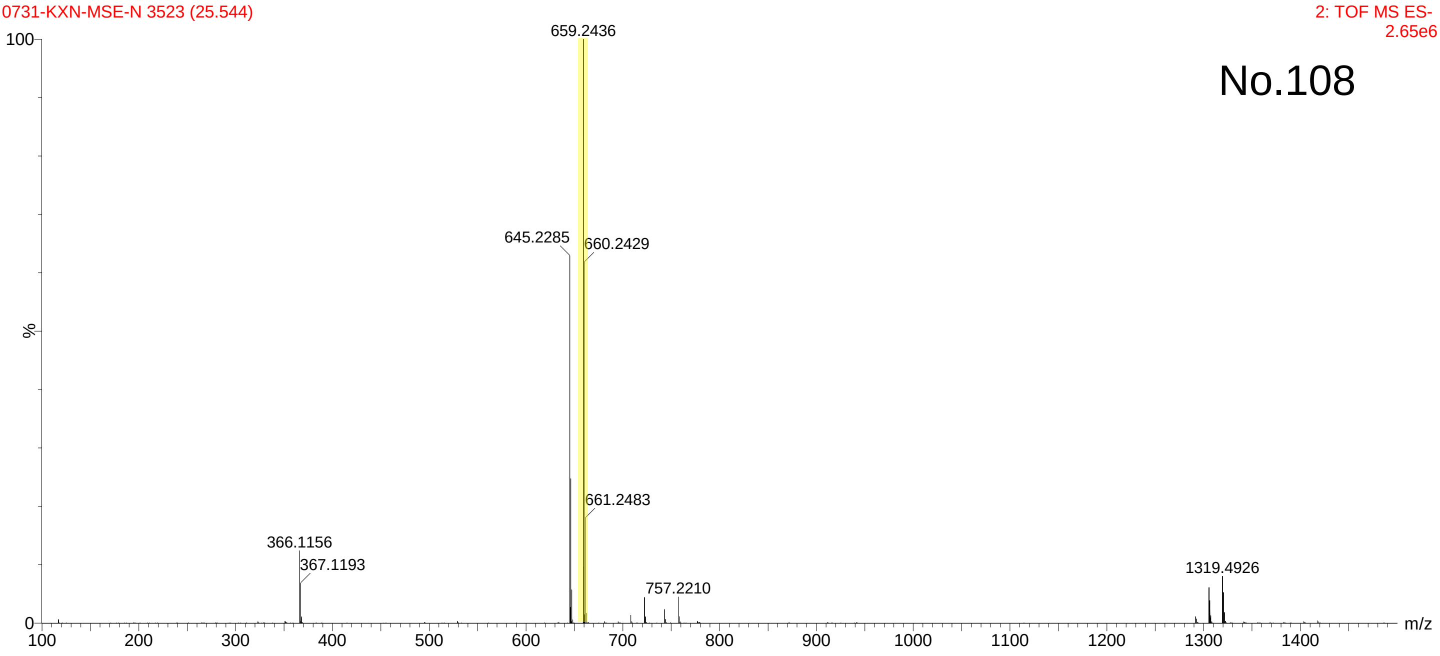

0731-KXN-MSE-N 3523 (25.544)
2: TOF MS ES-
2.65e6
No.108
100
%
0
659.2436
645.2285
660.2429
661.2483
366.1156
367.1193
757.2210
1319.4926
100
200
300
400
500
600
700
800
900
1000
1100
1200
1300
1400
m/z

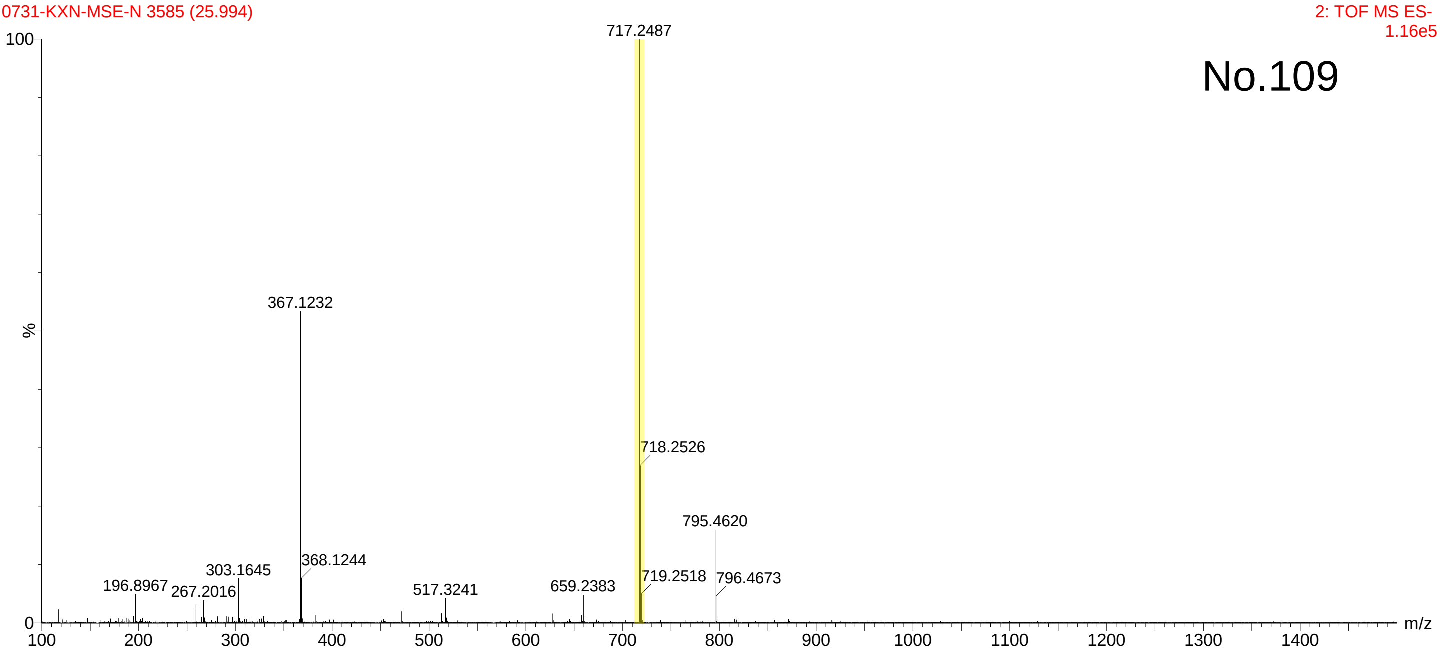
0731-KXN-MSE-N 3585 (25.994)
2: TOF MS ES-
1.16e5
No.109
717.2487
367.1232
718.2526
795.4620
368.1244
303.1645
196.8967
267.2016
517.3241
659.2383
719.2518
796.4673
%
m/z

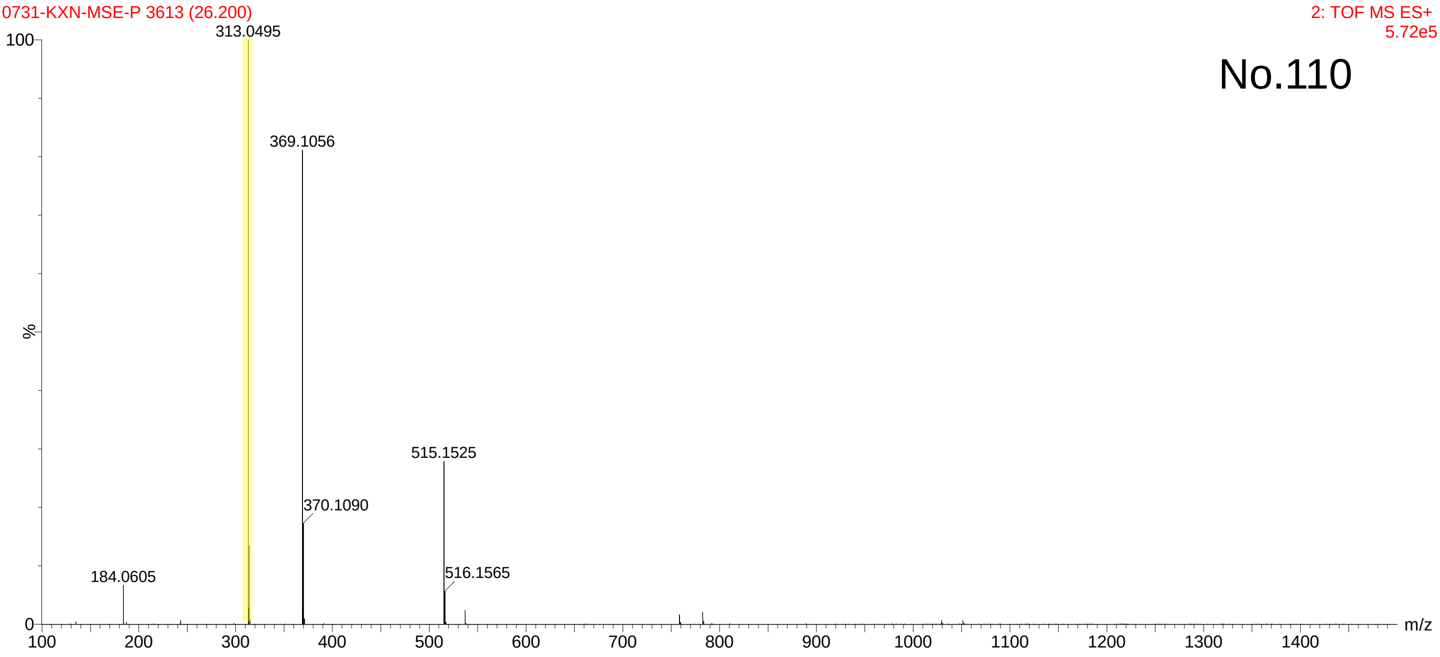
0731-KXN-MSE-P 3613 (26.200)
2: TOF MS ES+
5.72e5
No.110
313.0495
369.1056
515.1525
370.1090
184.0605
516.1565
%
m/z

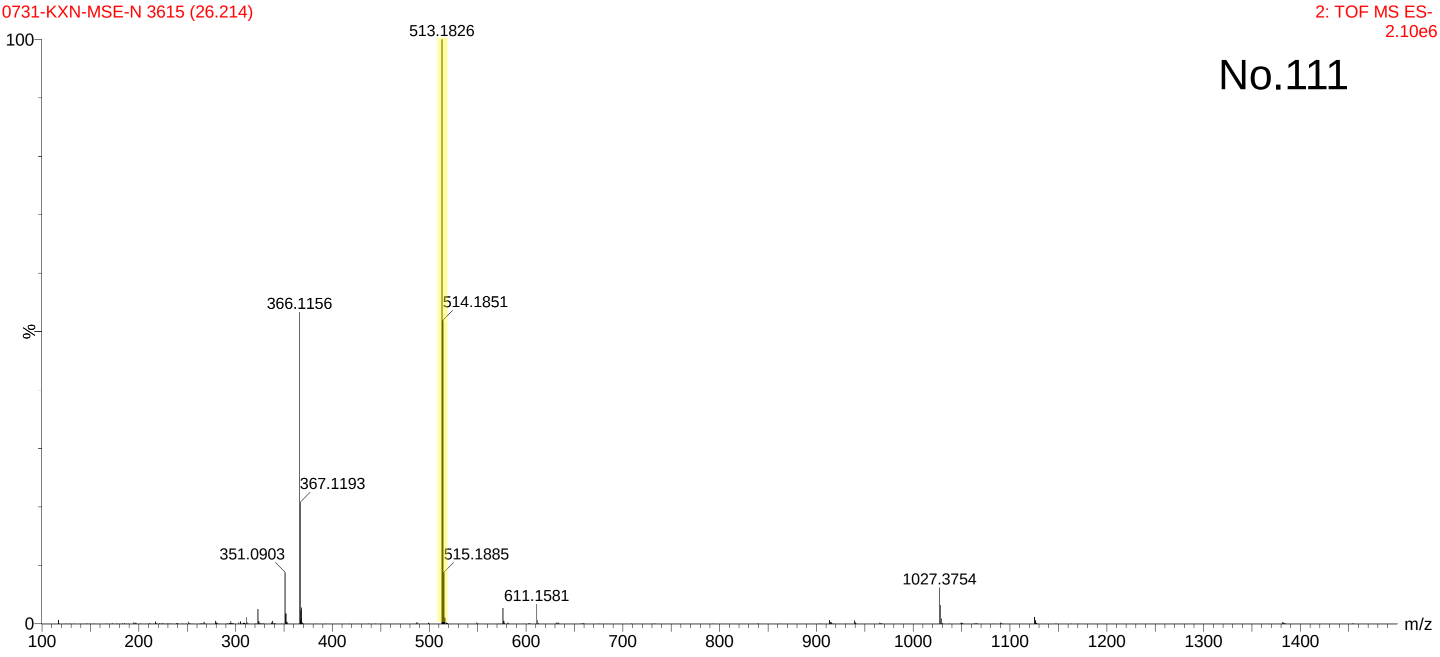
0731-KXN-MSE-N 3615 (26.214)
2: TOF MS ES-
2.10e6
No.111
513.1826
366.1156
514.1851
367.1193
351.0903
515.1885
611.1581
1027.3754
%
m/z

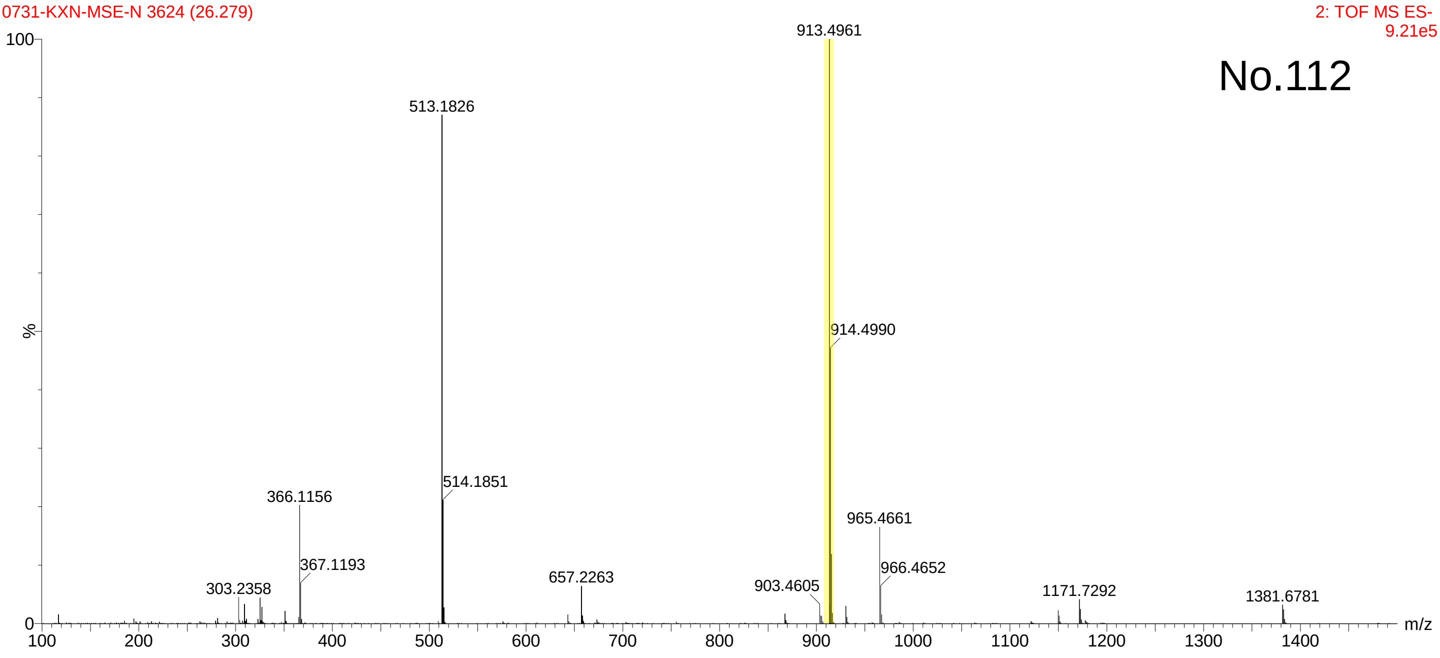
0731-KXN-MSE-N 3624 (26.279)
2: TOF MS ES-
9.21e5
No.112
913.4961
513.1826
914.4990
514.1851
366.1156
965.4661
367.1193
966.4652
303.2358
657.2263
903.4605
1171.7292
1381.6781
%
m/z

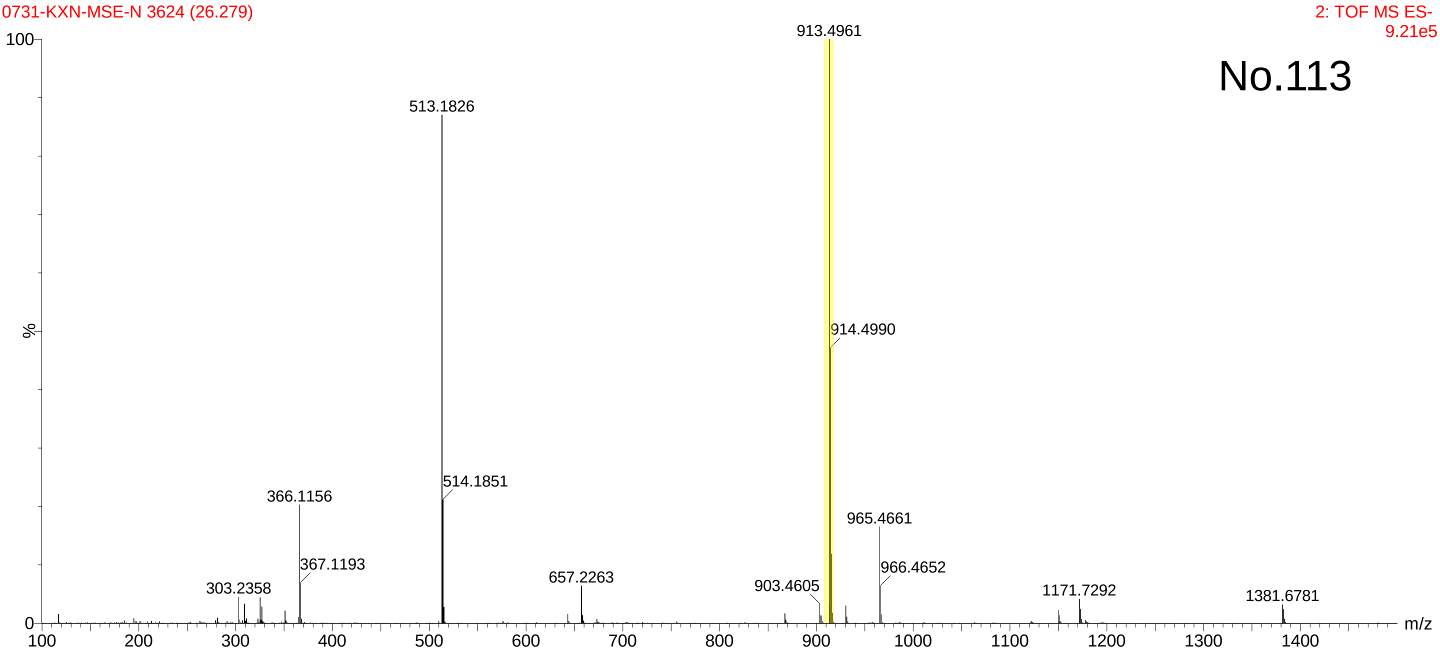
0731-KXN-MSE-N 3624 (26.279)
2: TOF MS ES-
9.21e5
No.113
913.4961
513.1826
914.4990
514.1851
366.1156
965.4661
367.1193
966.4652
303.2358
657.2263
903.4605
1171.7292
1381.6781
%
m/z

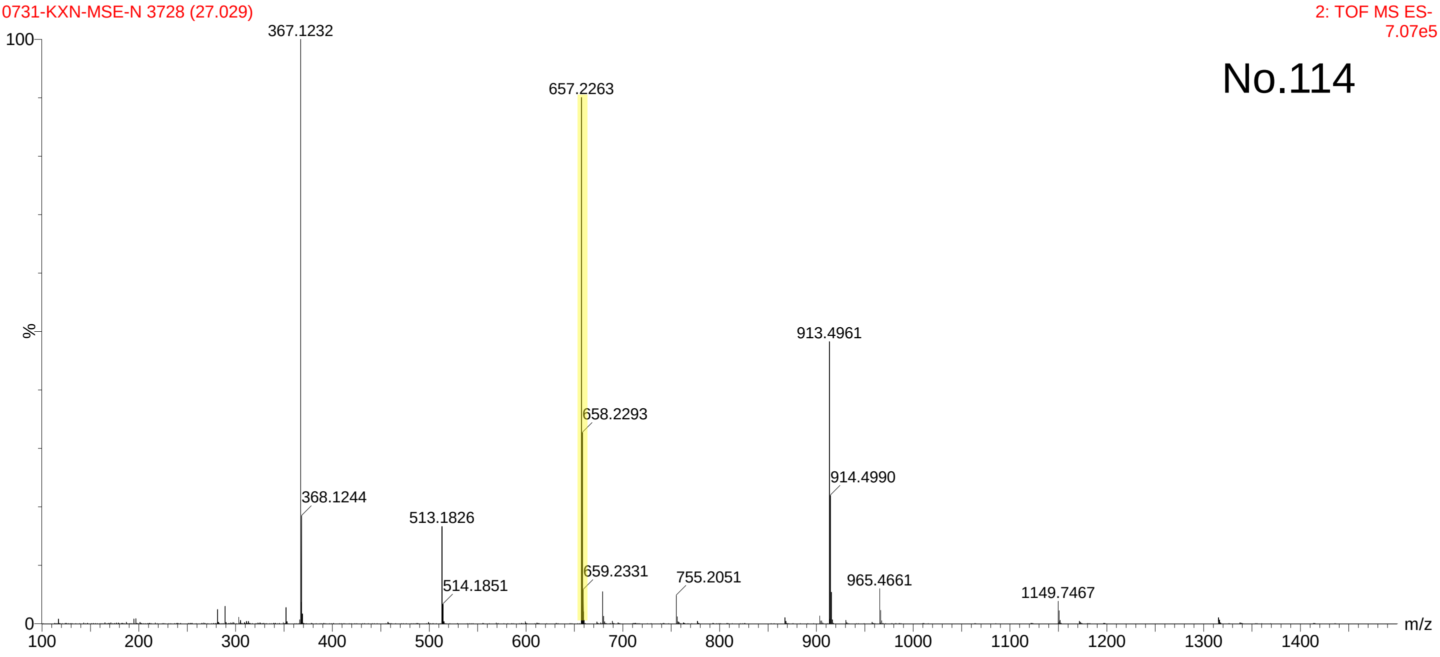
0731-KXN-MSE-N 3728 (27.029)
2: TOF MS ES-
7.07e5
No.114
367.1232
657.2263
913.4961
658.2293
914.4990
368.1244
513.1826
659.2331
755.2051
514.1851
965.4661
1149.7467
%
m/z

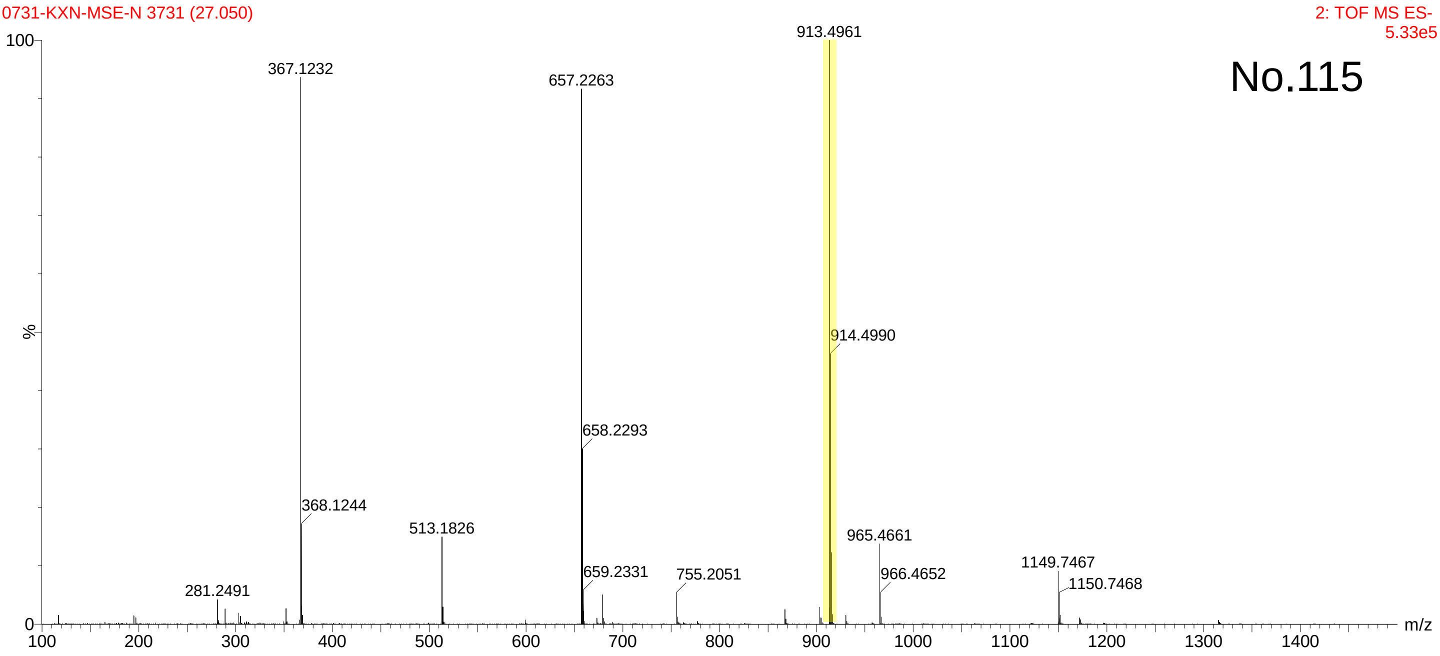

0731-KXN-MSE-N 3731 (27.050)
2: TOF MS ES-
5.33e5
No.115
100
%
0
913.4961
367.1232
657.2263
914.4990
658.2293
368.1244
513.1826
965.4661
1149.7467
1150.7468
659.2331
755.2051
966.4652
281.2491
100
200
300
400
500
600
700
800
900
1000
1100
1200
1300
1400
m/z

Table 2 16 absorbable components *in vivo* of KXN

No.	t_R/min	Measured value	Precursor ions	Formula	Theoretical value	Compound	Fragment ion	CAS No.
1	0.92	397.0942	$[M-H]^-$	$C_{22}H_{22}O_7$	397.1287	Baohuosu	268.8040, 259.0176, 191.0193	119730-90-4
2	4.30	309.0714	$[M+Na]^+$	$C_{13}H_{18}O_7$	309.0950	Sakakin	125.0573	21082-33-7
3	4.31	125.0504	$[M+H]^+$	$C_7H_8O_2$	125.0603	Guaiacol	110.0286	90-05-1
4	4.84	417.1438	$[M-H]^-$	$C_{22}H_{26}O_8$	417.1549	(-)-Syringaresinol	181.0548	6216-81-5
5	6.68	525.1688	$[M+HCOO]^-$	$C_{23}H_{28}O_{11}$	525.1608	Paeoniflorine	449.1524, 327.1119, 165.0581, 121.0305	23180-57-6
6	7.53	285.0525	$[M+H]^+$	$C_{16}H_{13}O_5$	285.0763	Wogonin	270.0334, 183.0345	632-85-9
7	7.56	283.0618	$[M-H]^-$	$C_{16}H_{12}O_5$	283.0607	Calycosin	268.0391, 239.0355, 211.0403, 195.0477	20575-57-9
8	9.02	463.0896	$[M-H]^-$	$C_{21}H_{20}O_{12}$	463.0877	Hyperoside	300.0280, 271.0277, 255.0323, 151.0040	482-36-0
9	12.11	269.0623	$[M+H]^+$	$C_{16}H_{13}O_4$	269.0814	Formononetin	254.0390, 237.0399	485-72-3
10	12.41	385.0988	$[M+Na]^+$	$C_{15}H_{22}O_{10}$	385.1111	Catalpol	355.0851, 223.0879, 203.0539	2415-24-9
11	15.94	167.0588	$[M+H]^+$	$C_9H_{10}O_3$	167.0708	Paeonol	149.0061, 124.8925, 121.0297	552-41-0
12	15.89	485.1012	$[M+Na]^+$	$C_{23}H_{26}O_{10}$	485.1424	Lactiflorin	105.0285	1361049-59-3
13	19.29	369.1135	$[M+H]^+$	$C_{21}H_{20}O_6$	369.1338	Icaritin	313.2378, 243.1672, 135.1075	118525-40-9
14	20.56	879.3061	$[M-H]^-$	$C_{41}H_{52}O_{21}$	879.2923	Epimedin I	717.2432, 367.1232	205445-00-7
15	24.24	829.4650	$[M+HCOO]^-$	$C_{45}H_{56}O_{23}$	829.4586	Astragaloside IV	783.3661	84687-43-4
16	26.21	513.1826	$[M-H]^-$	$C_{27}H_{30}O_{10}$	513.1761	Baohuoside I	366.1156, 351.0903, 323.0949, 217.0528	113558-15-9

Mass spectras of 16 absorbable components of KXN based on UPLC-Q/TOF-MS

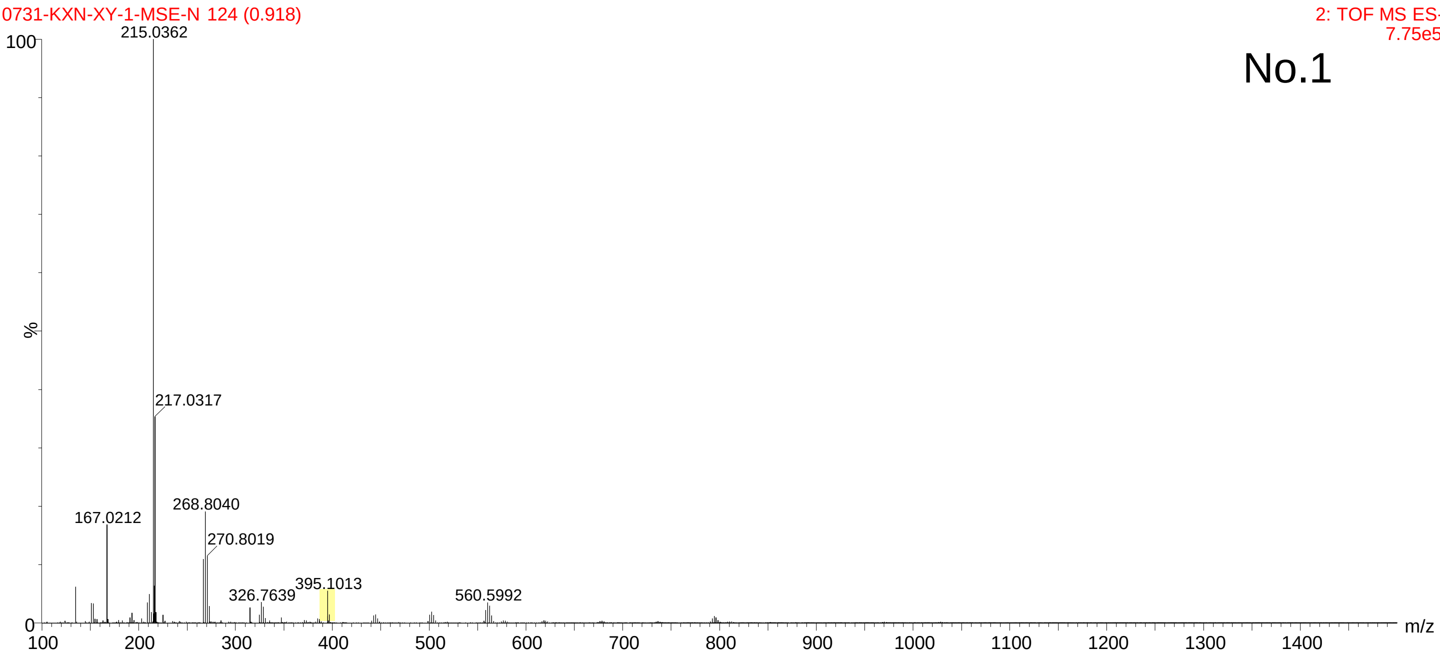

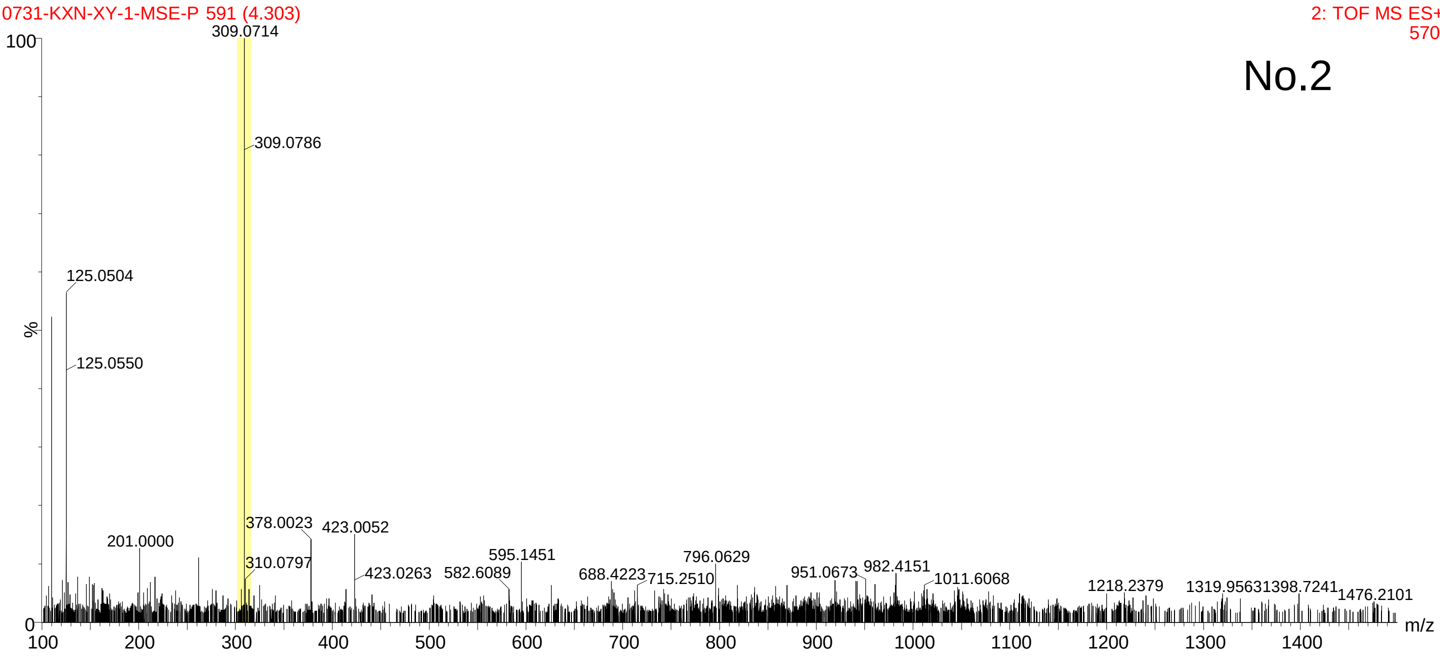

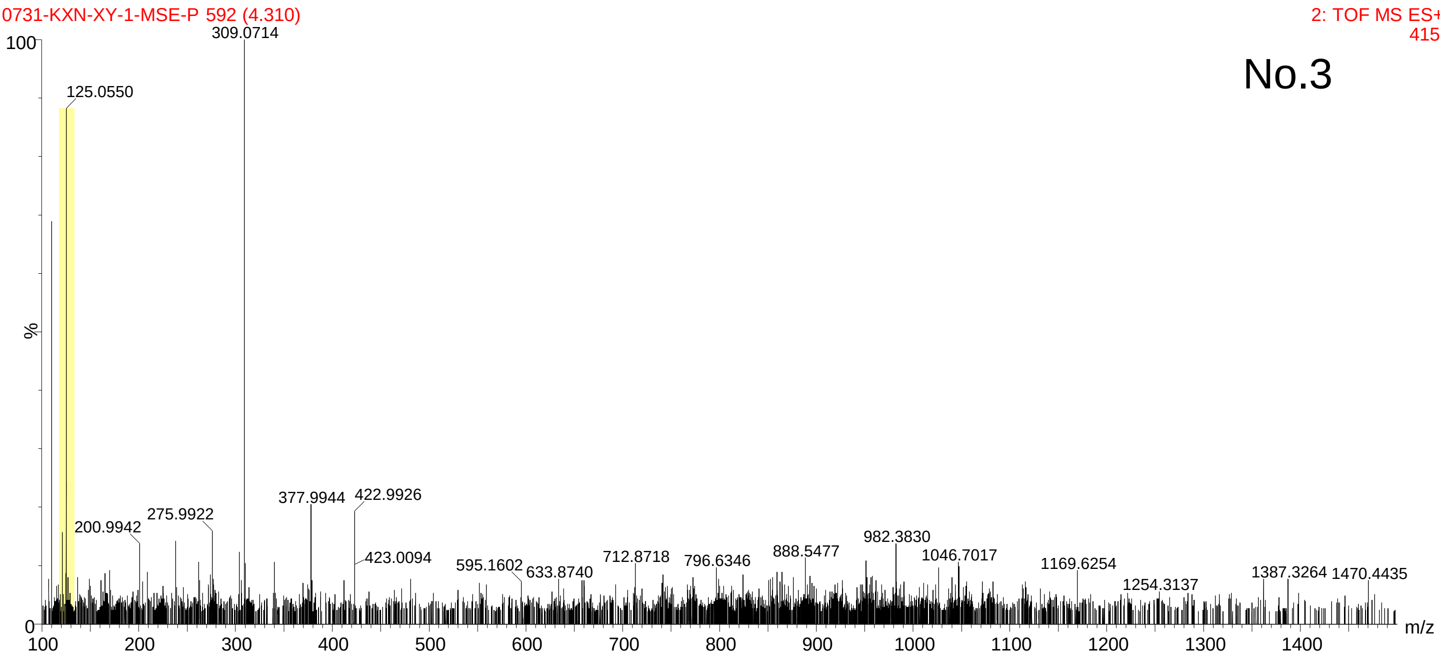

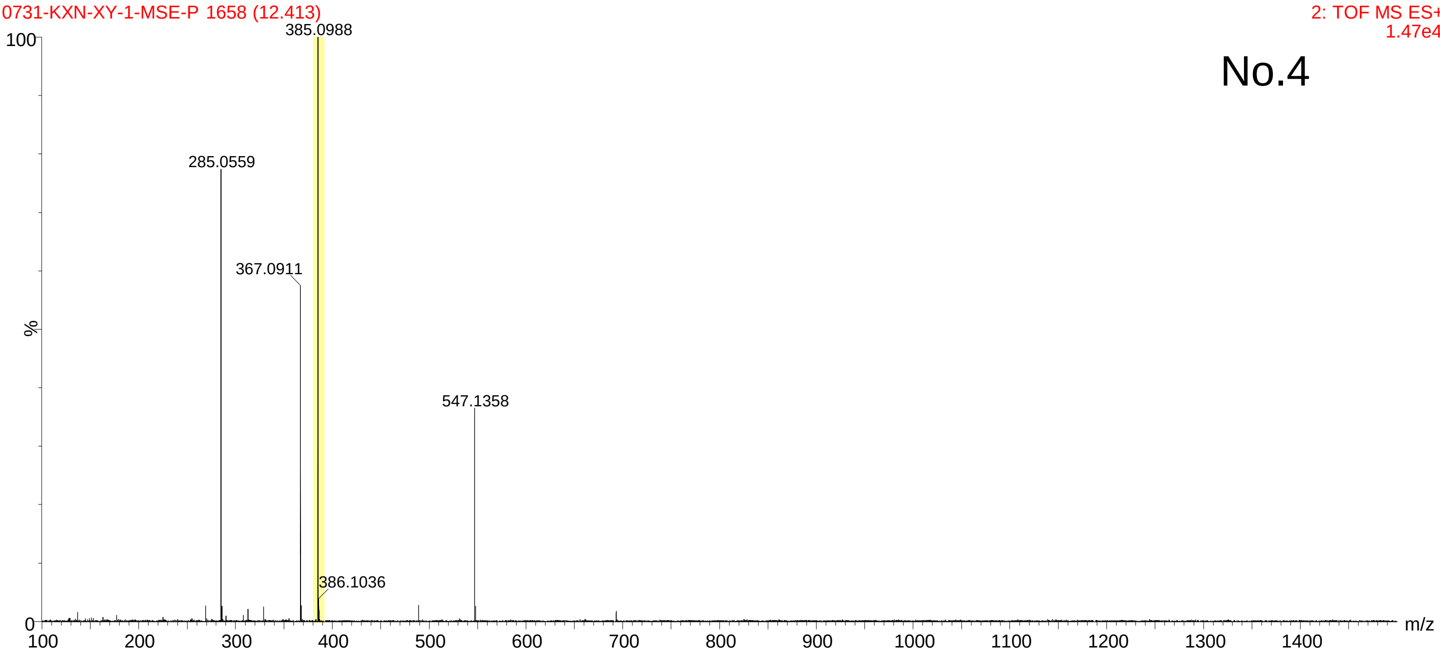

0731-KXN-XY-1-MSE-P 1658 (12.413)
2: TOF MS ES+
1.47e4
No.4
385.0988
285.0559
367.0911
547.1358
386.1036
%
m/z

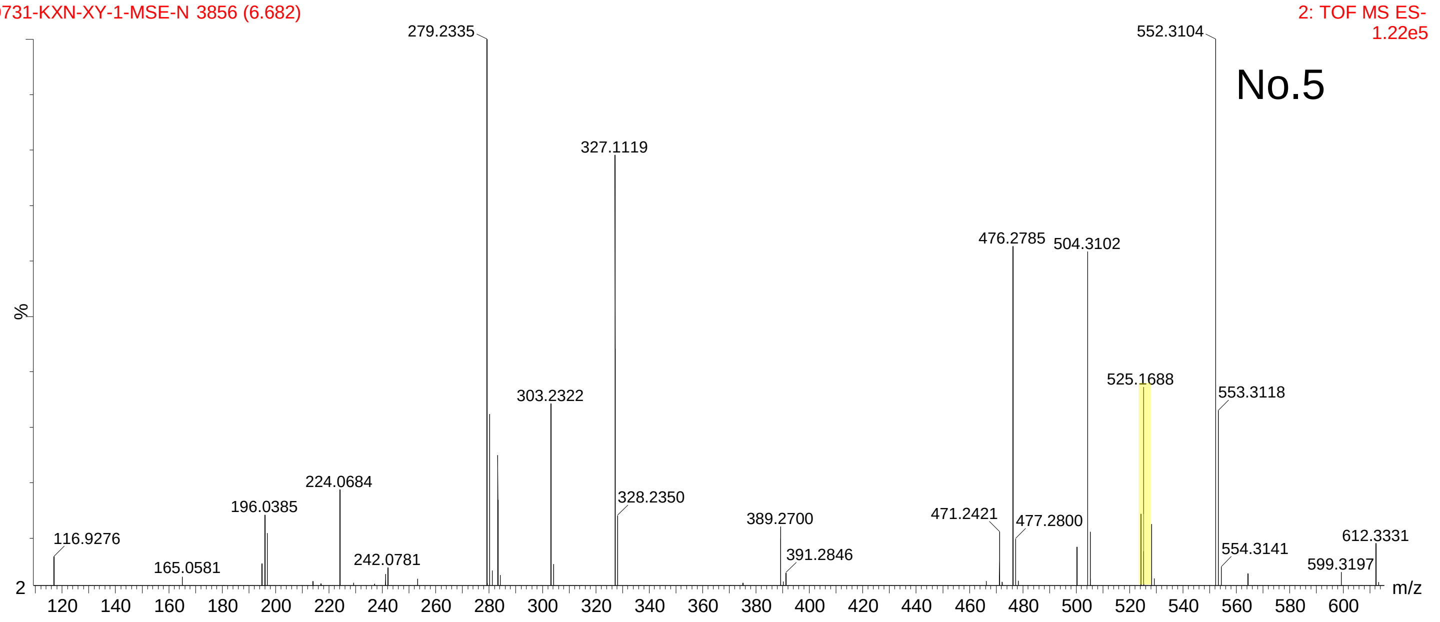

0731-KXN-XY-1-MSE-N 3856 (6.682)
2: TOF MS ES-
1.22e5
No.5
279.2335
552.3104
327.1119
476.2785
504.3102
525.1688
553.3118
303.2322
224.0684
196.0385
328.2350
389.2700
471.2421
477.2800
116.9276
612.3331
554.3141
391.2846
242.0781
165.0581
599.3197
%
m/z

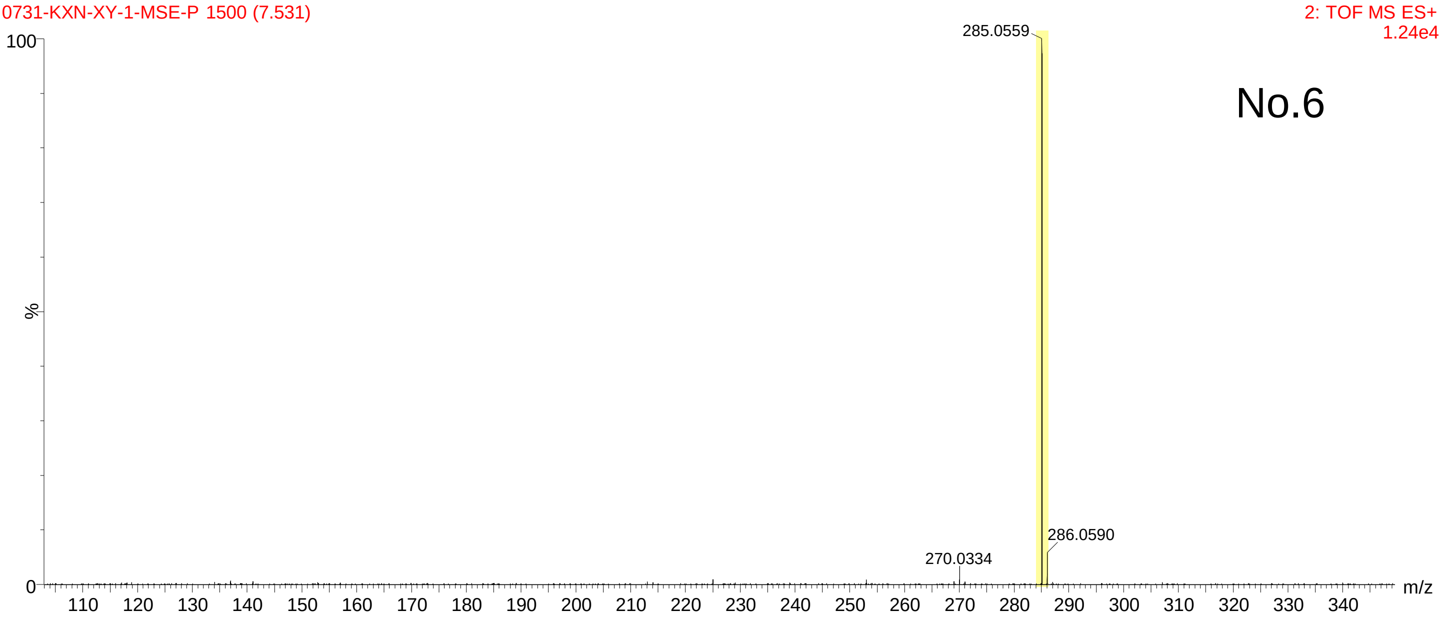

0731-KXN-XY-1-MSE-P 1500 (7.531)
2: TOF MS ES+
1.24e4
No.6
285.0559
286.0590
270.0334
%
m/z

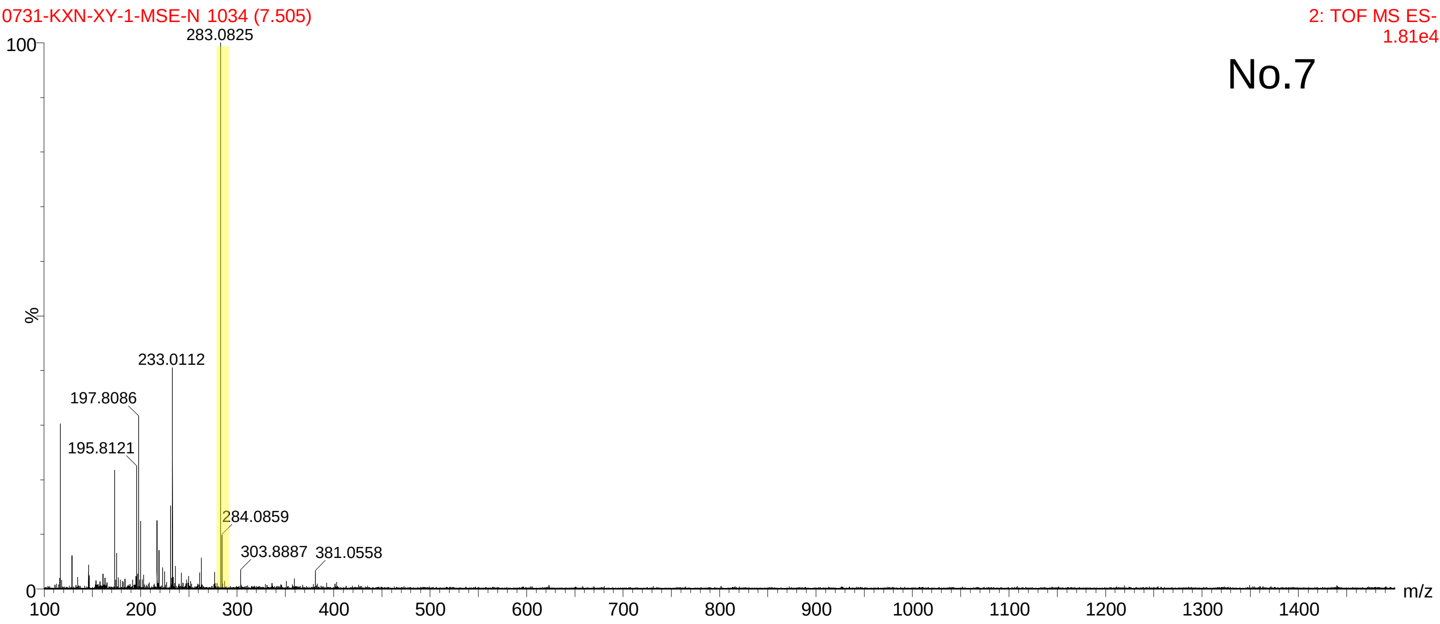
0731-KXN-XY-1-MSE-N 1034 (7.505)
2: TOF MS ES-
1.81e4
No.7
283.0825
233.0112
197.8086
195.8121
284.0859
303.8887
381.0558
100
%
0
m/z
100
200
300
400
500
600
700
800
900
1000
1100
1200
1300
1400

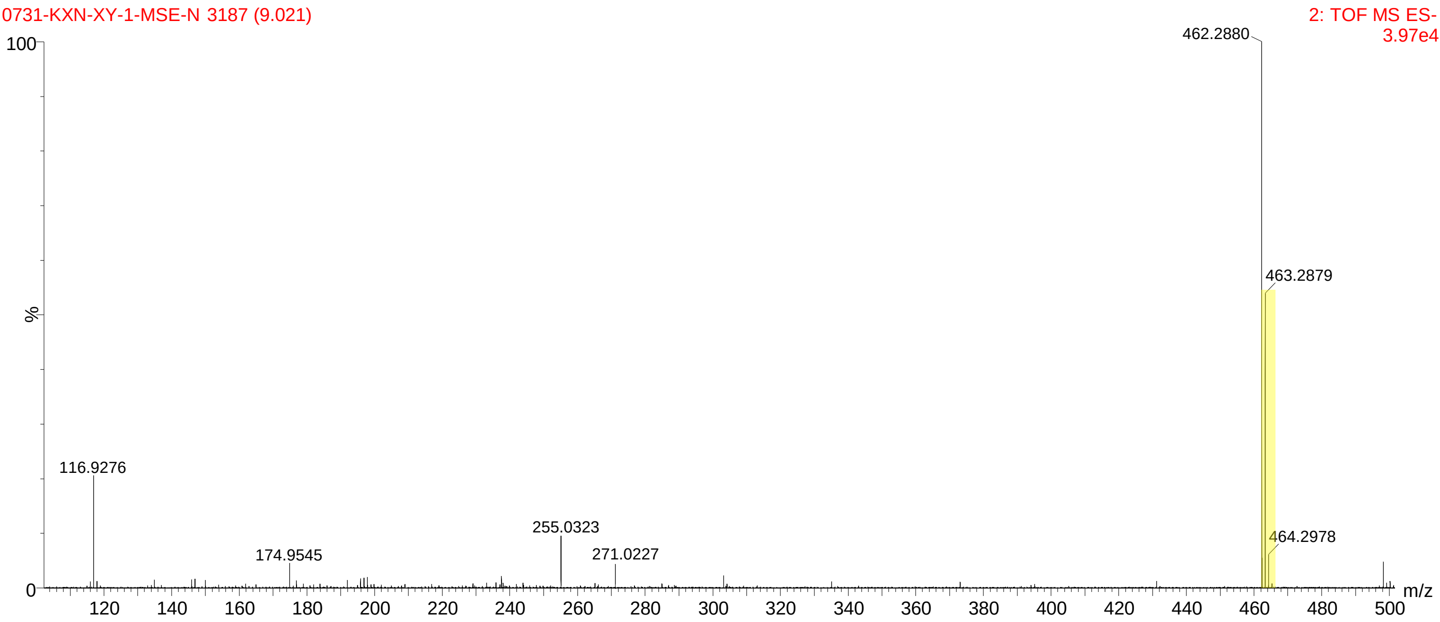
0731-KXN-XY-1-MSE-N 3187 (9.021)
2: TOF MS ES-
3.97e4
462.2880
463.2879
116.9276
255.0323
174.9545
271.0227
464.2978
100
%
0
m/z
120
140
160
180
200
220
240
260
280
300
320
340
360
380
400
420
440
460
480
500

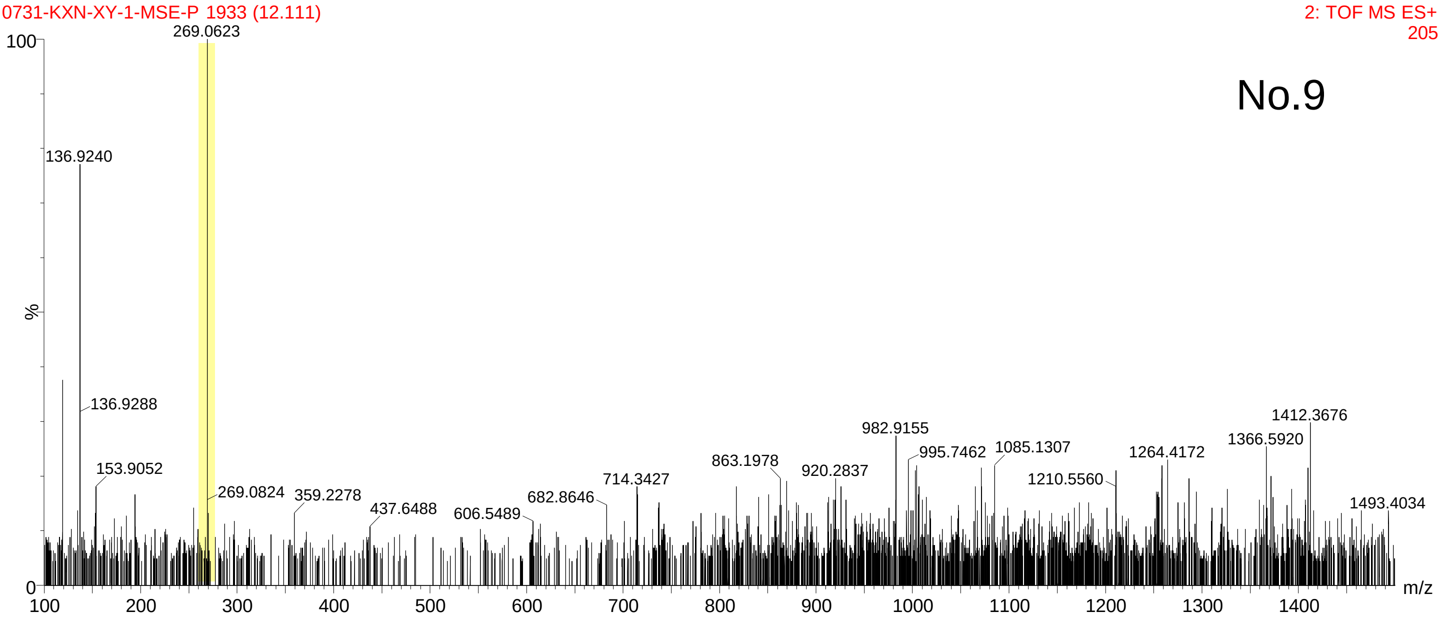
0731-KXN-XY-1-MSE-P 1933 (12.111)
2: TOF MS ES+
205
No.9
269.0623
136.9240
136.9288
153.9052
269.0824
359.2278
437.6488
606.5489
682.8646
714.3427
863.1978
920.2837
982.9155
995.7462
1085.1307
1210.5560
1264.4172
1366.5920
1412.3676
1493.4034
100
%
0
m/z
100
200
300
400
500
600
700
800
900
1000
1100
1200
1300
1400

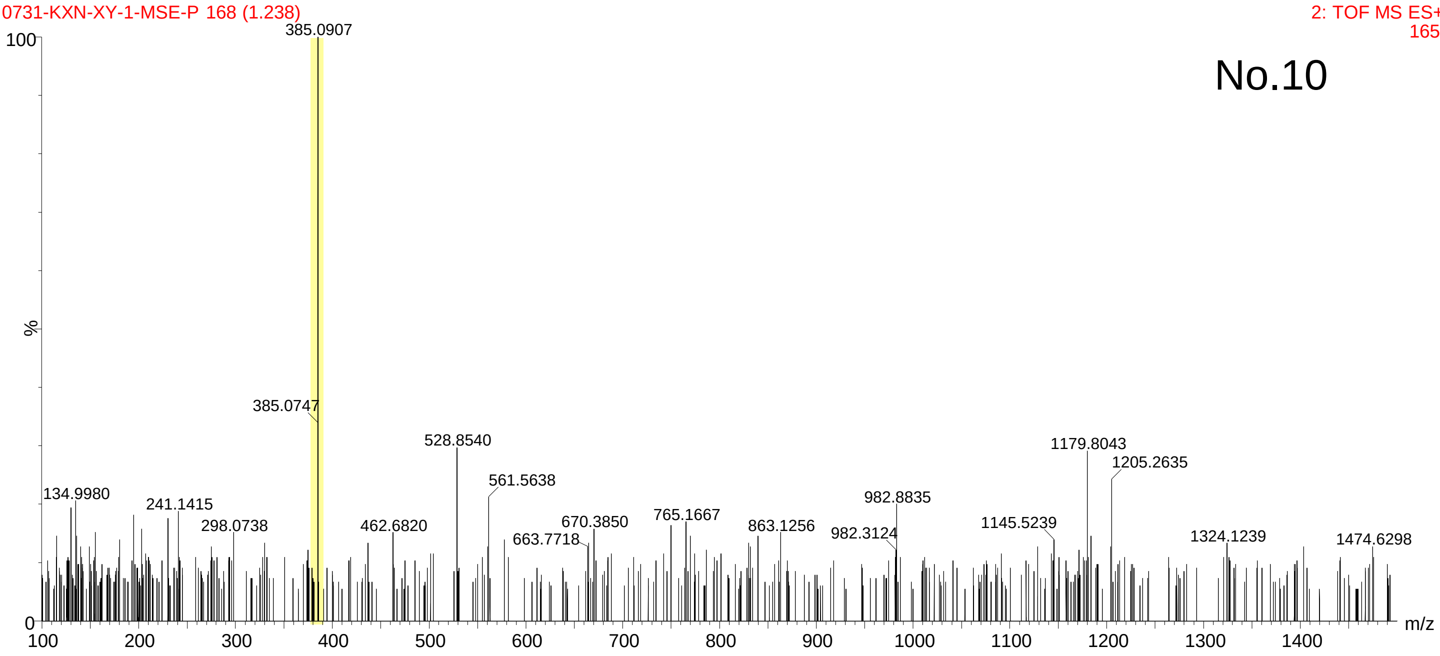
0731-KXN-XY-1-MSE-P 168 (1.238)
2: TOF MS ES+
165
No.10
385.0907
385.0747
528.8540
561.5638
134.9980
241.1415
298.0738
462.6820
670.3850
663.7718
765.1667
863.1256
982.8835
982.3124
1145.5239
1179.8043
1205.2635
1324.1239
1474.6298
100
%
0
m/z

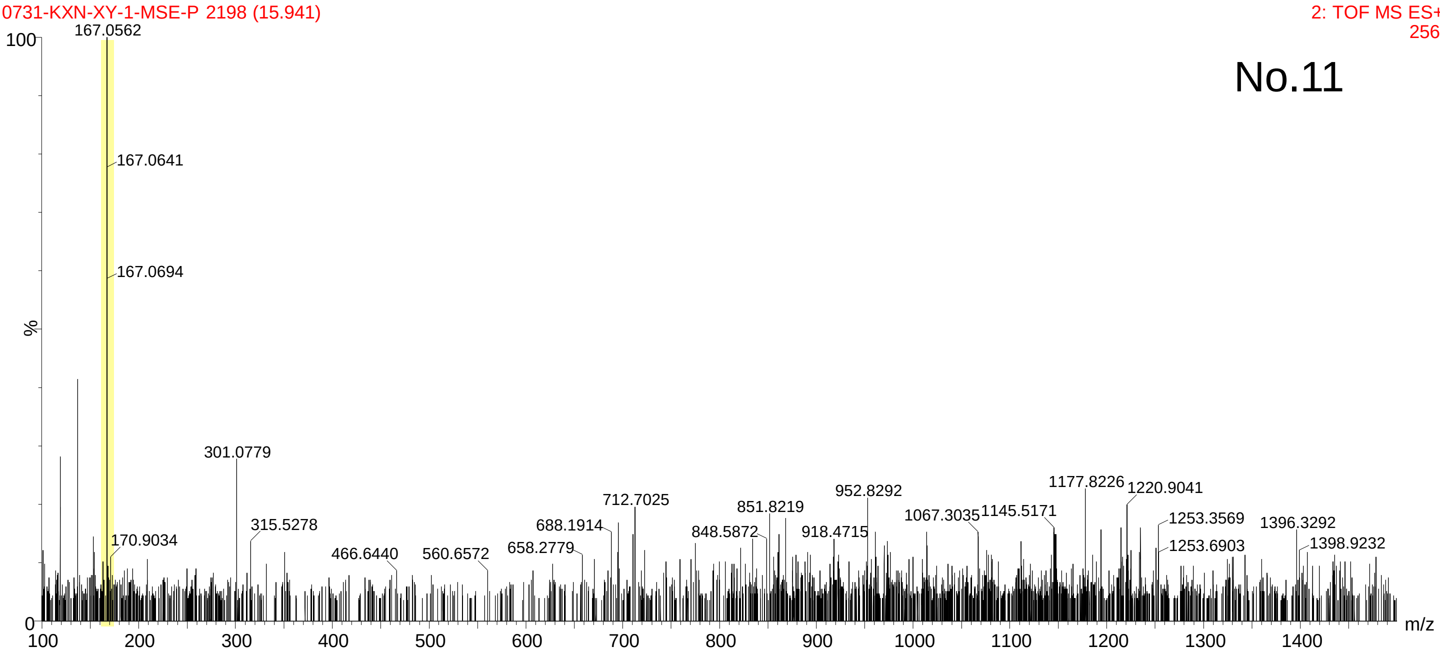
0731-KXN-XY-1-MSE-P 2198 (15.941)
2: TOF MS ES+
256
No.11
167.0562
167.0641
167.0694
301.0779
170.9034
315.5278
466.6440
560.6572
658.2779
688.1914
712.7025
848.5872
851.8219
918.4715
952.8292
1067.3035
1145.5171
1177.8226
1220.9041
1253.3569
1253.6903
1396.3292
1398.9232
100
%
0
m/z

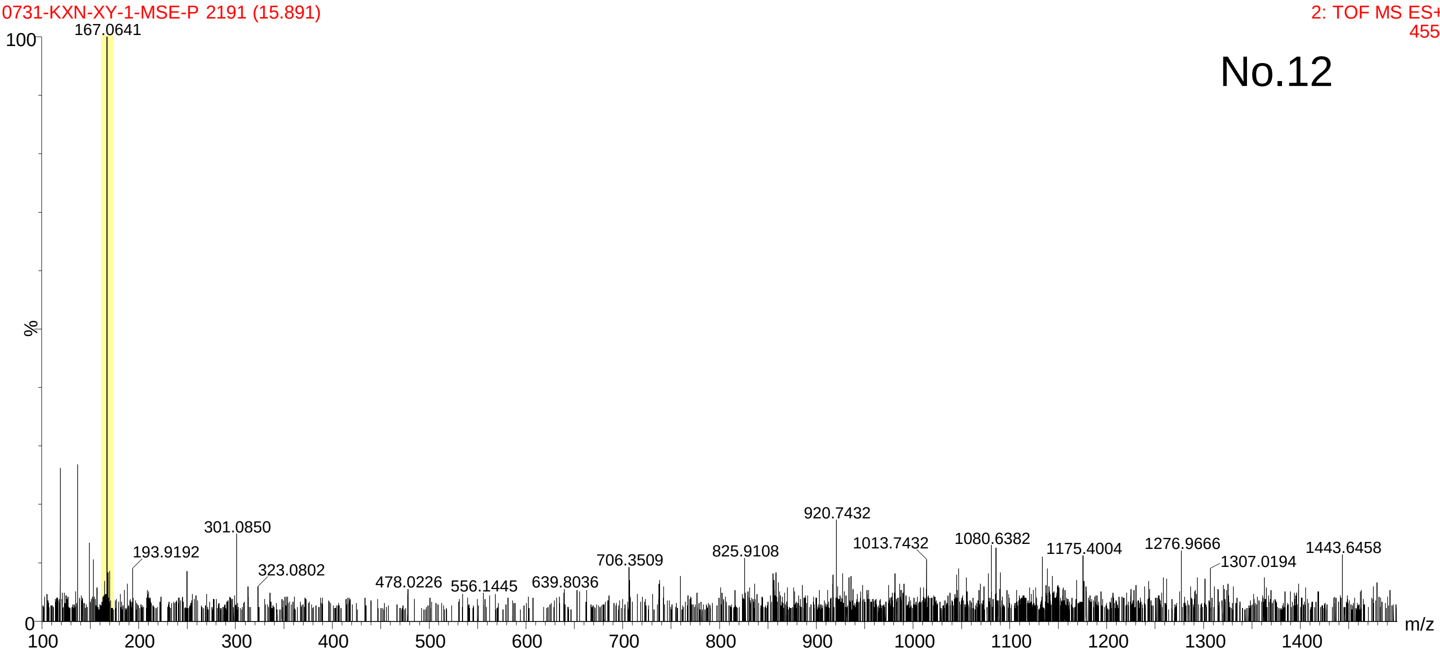
0731-KXN-XY-1-MSE-P 2191 (15.891)
2: TOF MS ES+
455
No.12
167.0641
301.0850
193.9192
323.0802
478.0226
556.1445
639.8036
706.3509
825.9108
920.7432
1013.7432
1080.6382
1175.4004
1276.9666
1307.0194
1443.6458
100
%
0
m/z

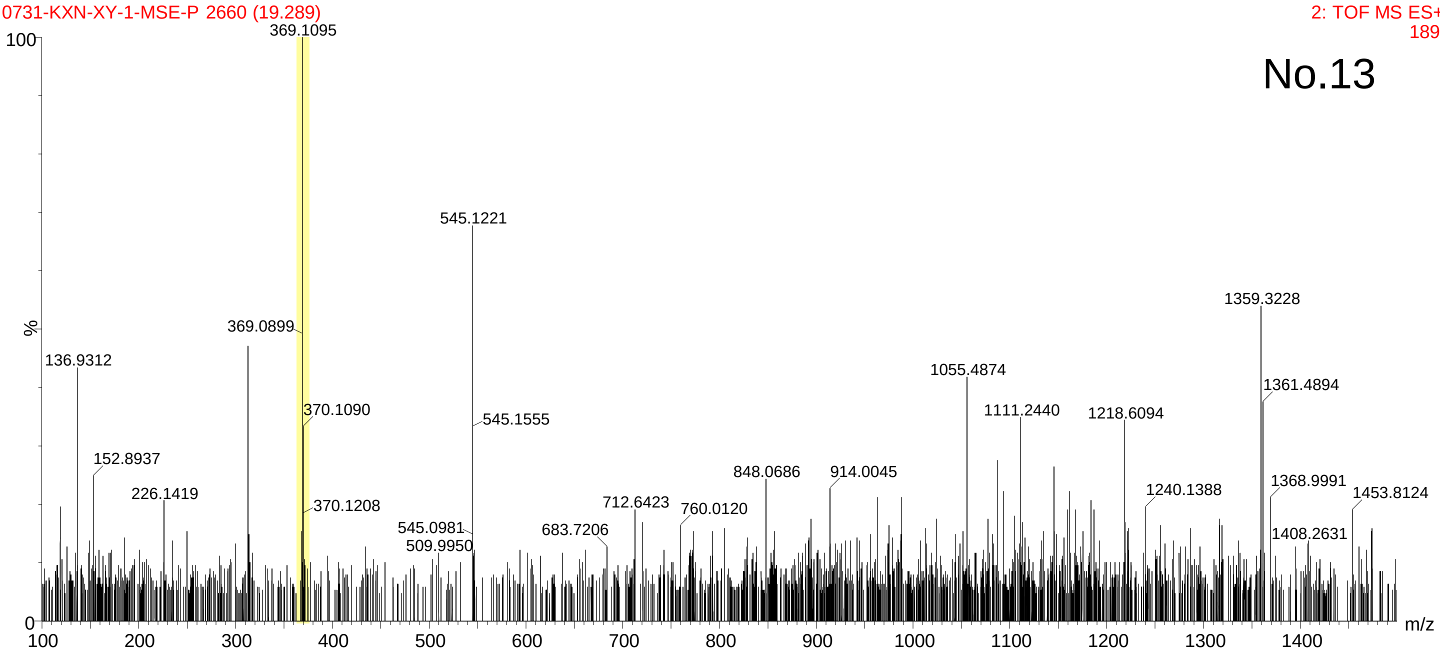
0731-KXN-XY-1-MSE-P 2660 (19.289)
2: TOF MS ES+
189
No.13
369.1095
545.1221
1359.3228
369.0899
136.9312
1055.4874
1361.4894
370.1090
545.1555
1111.2440
1218.6094
152.8937
848.0686
914.0045
226.1419
1240.1388
1368.9991
1453.8124
712.6423
760.0120
370.1208
683.7206
545.0981
509.9950
1408.2631
%
m/z

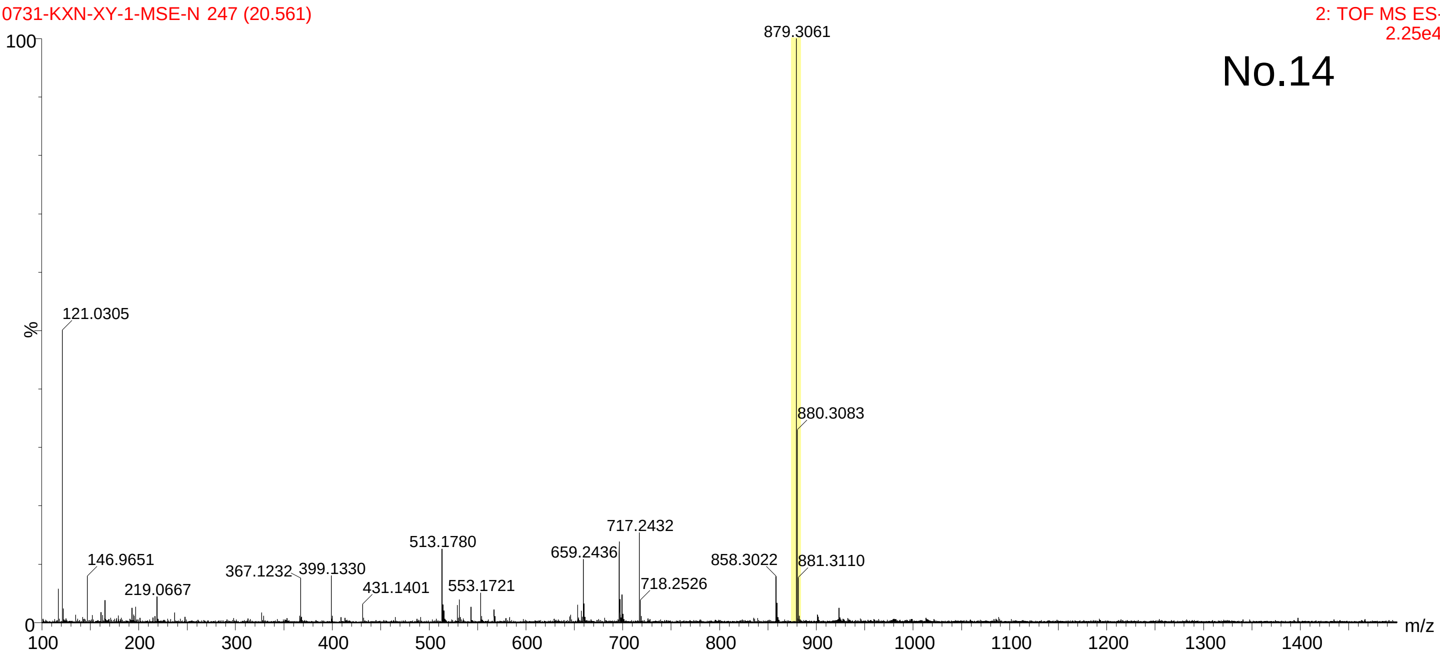
0731-KXN-XY-1-MSE-N 247 (20.561)
2: TOF MS ES-
2.25e4
No.14
879.3061
121.0305
880.3083
717.2432
513.1780
659.2436
146.9651
858.3022
881.3110
367.1232
399.1330
219.0667
431.1401
553.1721
718.2526
%
m/z

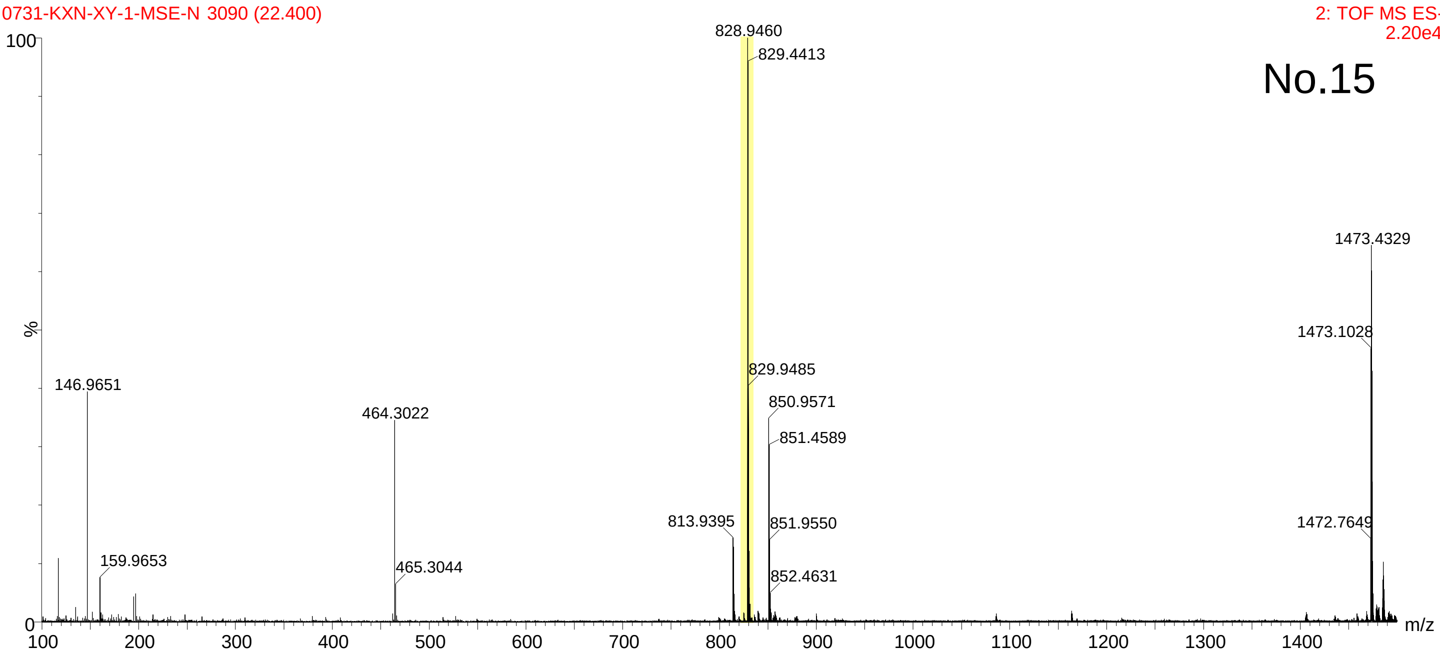
0731-KXN-XY-1-MSE-N 3090 (22.400)
2: TOF MS ES-
2.20e4
No.15
828.9460
829.4413
1473.4329
1473.1028
146.9651
829.9485
464.3022
850.9571
851.4589
813.9395
851.9550
1472.7649
159.9653
465.3044
852.4631
%
m/z

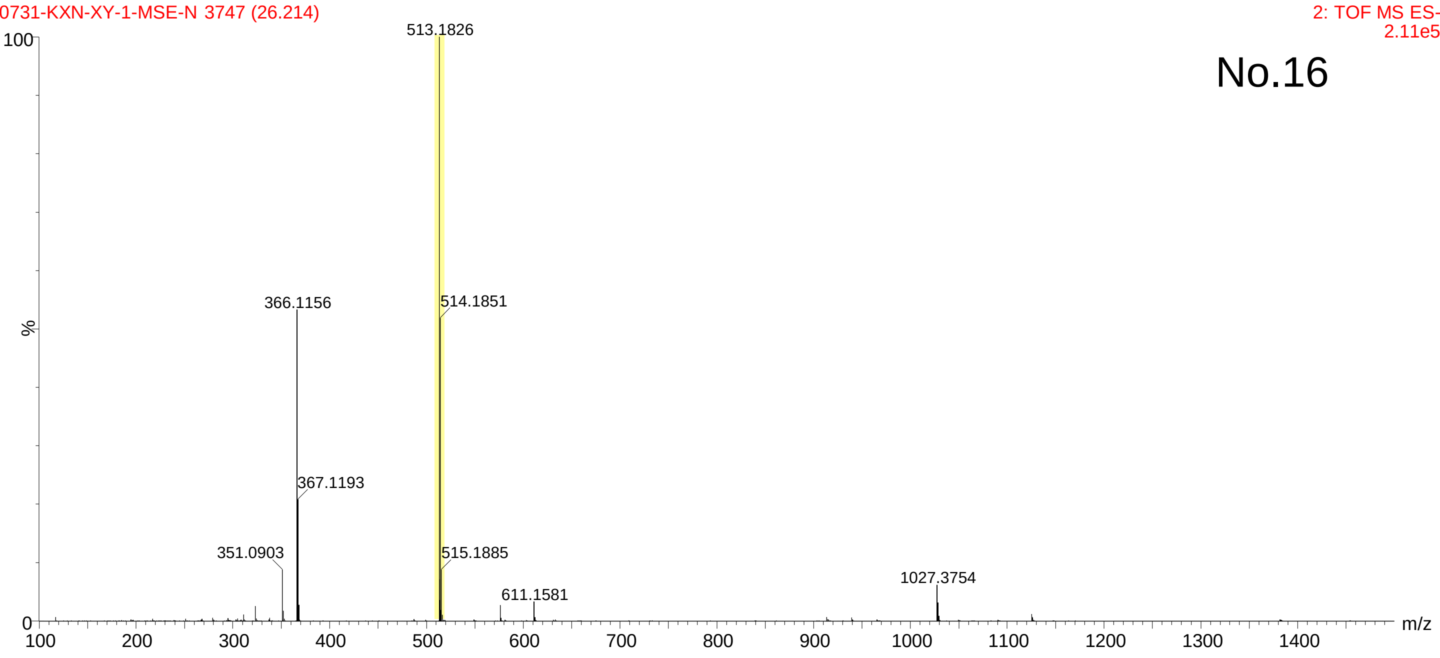
0731-KXN-XY-1-MSE-N 3747 (26.214)
2: TOF MS ES-
2.11e5
No.16
513.1826
366.1156
514.1851
367.1193
351.0903
515.1885
611.1581
1027.3754
100
%
0
m/z
100
200
300
400
500
600
700
800
900
1000
1100
1200
1300
1400
