# Supplementary material for: Kunxinning granules alleviate perimenopausal syndrome by supplementing estrogen deficiency
Source: Front Pharmacol. 2025 Mar 26;16:1554479. doi: 10.3389/fphar.2025.1554479 (PMC11979375; doi:10.3389/fphar.2025.1554479)
Supplement: Supplementary file 4 [file DataSheet1.pdf]

Supplementary Material

1. Chemical constituents of Kunxinning Granules identified by UPLC/Q-TOF/MS

1.1 Preparation of Kunxinning Granule sample

Take 2 bags of Kunxinning Granules (KXN) and grind them into a uniform powder. Take an appropriate amount of powder and dissolve it with 50% methanol to make 20 mg/mL KXN sample solution.

1.2 Chromatographic condition

The chromatography was performed on ACQUITY UPLC BEHC18 (2.1 mm ×100 mm, 1.7 μm) column at 30℃ with mobile phase A consisting of 0.1% formic acid water and mobile phase B consisting of acetonitrile by gradient elution (0-3 min, 5%-10% B; 3~4 min, 10%~18%B; 4~10 min, 18~22%B; 10~13 min, 22%~24%B; 13~18 min, 24%-26% B; 18-20 min, 26%-28% B; 20~23 min, 28~45%B; 23~26 min, 45%~65%B; 26~29 min, 65%~95%B; 29~31 min, 95%~5%B), the flow rate was 0.3 mL • min^{-1}, and the sample size was 2 μL.

1.3 Mass spectrum condition

The ion source was electrospray ion source (ESI), the ion source temperature was 100℃. The desolvent temperature was 250℃, the desolvent gas flow rate was 600 L • h^{-1}. The atomizer pressure was set to 45 psig. The source offset voltage was 80 V, the cone hole voltage was 30 V, and the capillary voltage was 2.5 kV. The m/z scan range is set from 100 to 1500, using the positive and negative ion scan mode.

The chemical composition of KXN was analyzed by UPLC/Q-TOF-MS. The Base Peak Ion (BPI) of KXN in positive and negative ion mode is shown in Supplementary Figure S1.

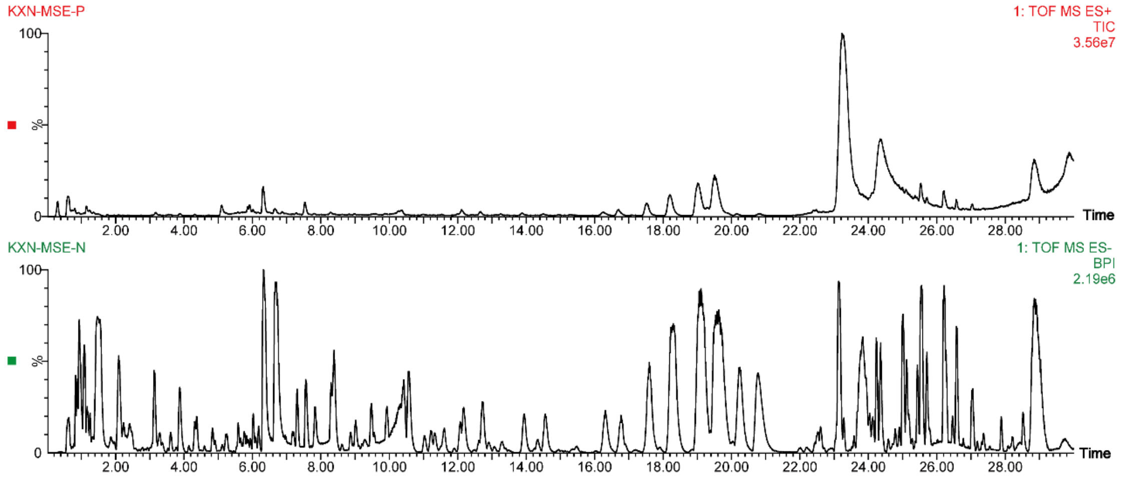

Supplementary Figure S1. BPI diagram of KXN UPLC/Q-TOF-MS in positive and negative ion mode.

According to database matching analysis, precise molecular weight, fragment ion peak and chromatographic retention time comparison, a total of 115 chemical components of KXN were identified, the specific information is shown in Supplementary Table S1. The mass spectras of each component based on UPLC/Q-TOF-MS are shown in supplementary data sheet S3.

Supplementary Table S1. Chemical components of KXN

No.	t_R/min	Measured value	Precursor ions	Formula	Theoretical value	Compound	Fragment ion	CAS No.
1	0.88	179.0573	$[M-H]^-$	$C_6H_{12}O_6$	179.0556	Inositol	160.9172	551-72-4
2	0.90	387.1156	$[M-H]^-$	$C_{17}H_{24}O_{10}$	387.1291	Geniposide	225.0657	24512-63-8
3	0.92	397.0942	$[M-H]^-$	$C_{22}H_{22}O_7$	397.1287	Baohuosu	397.0804, 259.0176, 191.0193	119730-90-4
4	1.42	191.0221	$[M-H]^-$	$C_6H_8O_7$	191.0192	Citric acid	173.0098, 129.0203	77-92-9
5	1.54	191.0193	$[M-H]^-$	$C_6H_8O_7$	191.0192	Citric acid isomer	173.0063, 129.0203, 111.0083	-
6	1.96	685.2255	$[M-H]^-$	$C_{27}H_{42}O_{20}$	685.2191	Rehmannioside D	517.1172, 365.1054	81720-08-3
7	2.10	169.0146	$[M-H]^-$	$C_7H_6O_5$	169.0137	Gallic acid	125.0256	149-91-7
8	2.92	509.1918	$[M-H]^-$	$C_{21}H_{34}O_{14}$	509.1870	Rehmannioside C	449.1263, 179.0573	81720-07-2
9	3.12	371.1020	$[M+Na]^+$	$C_{15}H_{24}O_9$	371.1318	Ajugol	191.0596	52949-83-4
10	3.69	527.1464	$[M-H]^-$	$C_{23}H_{28}O_{14}$	527.1401	6'-O-galloyldesbenzoyl paeoniflorin	403.0494, 169.0146, 125.0256	262350-51-6
11	3.85	471.1115	$[M+Na]^+$	$C_{19}H_{28}O_{12}$	471.1479	Anacardoside isomer	309.0966, 125.0504	164991-86-0
12	4.21	309.0714	$[M+Na]^+$	$C_{13}H_{18}O_7$	309.0950	Sakakin	125.0573	21082-33-7
13	4.31	125.0504	$[M+H]^+$	$C_7H_8O_2$	125.0603	Guaiacol	110.0286	90-05-1
14	4.84	417.1438	$[M-H]^-$	$C_{22}H_{26}O_8$	417.1549	(-)-Syringaresinol	181.0548	6216-81-5
15	5.10	342.1419	$[M+H]^+$	$C_{20}H_{23}O_4N$	342.1705	Magnoflorine	297.0087, 282.0677, 265.0652	2141-09-5
16	5.59	495.1552	$[M-H]^-$	$C_{23}H_{28}O_{12}$	495.1508	Oxypaeoniflorin	465.1453, 333.1017, 281.0671, 177.0571, 165.0555, 137.0257	39011-91-1
17	5.69	317.0785	$[M+H]^+$	$C_{16}H_{12}O_7$	317.0661	Isorhamnetin	302.0945	480-19-3
18	5.83	525.1642	$[M+HCOO]^-$	$C_{23}H_{28}O_{11}$	525.1608	Paeoniflorine isomer	479.1608, 449.1176	23180-57-6
19	5.94	337.0939	$[M-H]^-$	$C_{16}H_{18}O_8$	337.0923	5-p-coumaroylquinic-acid	191.0561, 173.0475, 163.0412, 137.0257	1899-30-5
20	6.03	353.0884	$[M-H]^-$	$C_{16}H_{18}O_9$	353.0873	Neochlorogenic-acid	191.0589, 179.0353, 135.0458	906-33-2
21	6.33	525.1688	$[M+HCOO]^-$	$C_{23}H_{28}O_{11}$	525.1608	Albiflorin	479.1608, 357.1246, 283.0859, 121,0305	39011-90-0
22	6.56	711.2562	$[M-H]^-$	$C_{33}H_{44}O_{17}$	711.2500	(-) - Syringol-4-o-β- D-carvacosyl - (1 → 2)- β- D-glucopyranoside	417.1605, 181.0521	136997-64-3
23	6.63	579.2137	$[M-H]^-$	$C_{28}H_{36}O_{13}$	579.2078	(-)-Syringaresinol4-O-β-D-	449.1481, 417.1563, 181.0493	137038-13-2

						glucopyranoside		
24	6.68	525.1688	[M+HCOO] $^-$	$C_{23}H_{28}O_{11}$	525.1608	Paeoniflorine	449.1524, 327.1119, 165.0581, 121.0305	23180-57-6
25	6.79	475.1307	[M+HCOO]$^-$	$C_{22}H_{22}O_9$	475.1231	Ononin	267.0677	486-62-4
26	7.28	593.1547	[M-H]$^-$	$C_{27}H_{30}O_{15}$	593.1512	Quercetin3,7-O-rhamnopyranoside	447.0977, 301.0397	28638-13-3
27	7.29	181.0352	[M+H]$^+$	$C_9H_8O_4$	181.0501	Theobromine		83-67-0
28	7.53	447.0938	[M+H]$^+$	$C_{22}H_{22}O_{10}$	447.1291	Calycosin-7-O-β-D-glucoside	285.0525, 270.0334, 213.0376	20633-67-4
29	7.53	285.0525	[M+H]$^+$	$C_{16}H_{13}O_5$	285.0763	Wogonin	270.0334, 183.0345	632-85-9
30	7.56	283.0618	[M-H]$^-$	$C_{16}H_{12}O_5$	283.0607	Calycosin	268.0391, 239.0355, 211.0403, 195.0477	20575-57-9
31	7.81	503.1127	[M+Na]$^+$	$C_{23}H_{28}O_{11}$	503.1529	Albiflorin isomer	341.0789	-
32	8.30	465.1453	[M-H]$^-$	$C_{22}H_{26}O_{11}$	465.1397	Curculigoside	327.1082, 204.9773, 123.0461	85643-19-2
33	8.38	631.1736	[M-H]$^-$	$C_{30}H_{32}O_{15}$	631.1663	Galloyl-paeoniflorin	613.1644, 509.1364, 491.1263, 463.1293	122965-41-7
34	8.87	711.2562	[M-H]$^-$	$C_{33}H_{44}O_{17}$	711.2500	(-)-Syringaresinol4-O-β-D-glucopyranoside isomer	417.1605, 181.0548	-
35	8.88	547.1693	[M+Na]$^+$	$C_{21}H_{32}O_{15}$	547.1639	Rehmannioside A isomer	347.1309	-
36	8.89	547.1693	[M+Na]$^+$	$C_{26}H_{36}O_{11}$	547.2155	Icariside E3	489.1534, 205.0713	137822-23-2
37	9.02	463.0896	[M-H]$^-$	$C_{21}H_{20}O_{12}$	463.0877	Hyperoside	300.0280, 271.0277, 255.0323, 151.0040	482-36-0
38	9.20	417.1564	[M-H]$^-$	$C_{18}H_{26}O_{11}$	417.1397	Orcinol-1-O-β-D-apiofuranosyl-(1→6)-β-D-glucopyranoside	109.0280	868557-54-4
39	9.22	547.1693	[M+Na]$^+$	$C_{21}H_{32}O_{15}$	547.1639	Rehmannioside A isomer	347.1042	-
40	9.24	547.1693	[M+Na]$^+$	$C_{26}H_{36}O_{11}$	547.2155	Icariside E3 isomer	489.1670, 205.0831	-
41	9.28	301.0006	[M-H]$^-$	$C_{14}H_6O_8$	300.9984	Ellagic acide	283.9997, 229.0150	476-66-4
42	9.48	623.2043	[M-H]$^-$	$C_{29}H_{36}O_{15}$	623.1976	Acteoside	461.1704, 161.0264	61276-17-3
43	9.48	623.2043	[M-H]$^-$	$C_{29}H_{36}O_{15}$	623.1976	Isoacteoside	461.1703, 161.0264	61303-13-7
44	9.48	623.2043	[M-H]$^-$	$C_{29}H_{36}O_{15}$	623.1976	Isoverproside	461.1703, 161.0264	61303-13-7
45	9.67	939.1226	[M-H]$^-$	$C_{41}H_{32}O_{26}$	939.1104	Pentagalloyl-glucose	617.0902, 465.0791, 295.0512, 169.0146	14937-32-7
46	9.73	503.1127	[M+Na]$^+$	$C_{23}H_{28}O_{11}$	503.1529	Albiflorin isomer	381.0847, 341.0751, 219.0484	-
47	9.93	631.1736	[M-H]$^-$	$C_{30}H_{32}O_{15}$	631.1663	Galloylpaeoni-florin isomer	509.1502, 463.1248, 271.0547	-
48	10.18	503.1127	[M+Na]$^+$	$C_{23}H_{28}O_{11}$	503.1529	Albiflorin isomer	381.0887,	-

							341.0826, 219.0484	
49	10.48	503.1127	$[M+Na]^+$	$C_{23}H_{28}O_{11}$	503.1529	Albiflorin isomer	487.1952, 341.1091	-
50	10.86	367.0872	$[M+Na]^+$	$C_{16}H_{24}O_8$	367.1369	Mudanpioside F	205.1124	172670-08-5
51	10.88	163.0412	$[M-H]^-$	$C_9H_8O_3$	163.0395	p-Hydroxy-cinnamic acid	119.0495	7400-08-0
52	11.03	677.2142	$[M-H]^-$	$C_{32}H_{38}O_{16}$	677.2082	Demethylicaritin-7-O-sophoroside	530.1925, 370.1111	101072-83-7
53	11.03	677.2142	$[M-H]^-$	$C_{32}H_{38}O_{16}$	677.2082	Hexandraside E	515.1606, 353.1076	139955-75-2
54	11.25	447.0977	$[M-H]^-$	$C_{21}H_{20}O_{11}$	447.0933	Quercetin 3-rhamnoside	301.0361, 284.0445, 255.0323	522-12-3
55	11.60	823.2723	$[M-H]^-$	$C_{38}H_{48}O_{20}$	823.2661	Rouhuoside	661.2167, 515.1606, 353.1076	131862-37-8
56	11.90	523.1858	$[M-H]^-$	$C_{21}H_{32}O_{15}$	523.2663	Rehmannioside A	323.1022, 199.1009	81720-05-0
57	12.07	793.2632	$[M-H]^-$	$C_{37}H_{46}O_{19}$	793.2555	Epimedoside E	631.2095, 352.0925	39049-19-9
58	12.10	431.1008	$[M+H]^+$	$C_{22}H_{22}O_9$	431.1342	Ononin	269.0623	486-62-4
59	12.11	269.0623	$[M+H]^+$	$C_{16}H_{13}O_4$	269.0814	Formononetin	254.0390, 237.0399	485-72-3
60	12.17	267.0677	$[M-H]^-$	$C_{16}H_{12}O_4$	267.0658	Formononetin isomer	252.0456, 223.0427, 195.0477	-
61	12.41	385.0988	$[M+Na]^+$	$C_{15}H_{22}O_{10}$	385.1111	Catalpol	355.1005, 223.0653, 203.0597	2415-24-9
62	12.61	807.2815	$[M-H]^-$	$C_{38}H_{48}O_{19}$	807.2712	Diphylloside B	661.2220, 645.2285, 499.1685, 514.1526, 353.1076	-
63	12.72	661.2167	$[M-H]^-$	$C_{32}H_{38}O_{15}$	661.2138	Epimedoside A	514.1526, 499.1685, 395.1136, 353.1037	39012-04-9
64	13.00	517.1289	$[M+Na]^+$	$C_{19}H_{26}O_{15}$	517.1169	Galloylsucrose	355.0927	-
65	13.07	807.2815	$[M-H]^-$	$C_{38}H_{48}O_{19}$	807.2712	Epimedin B	645.2285, 367.1232, 351.0903, 323.1022	110623-73-9
66	13.26	385.0988	$[M+Na]^+$	$C_{15}H_{22}O_{10}$	385.1111	Catalpol isomer	355.0889, 223.0683, 203.0656	-
67	13.68	629.1945	$[M-H]^-$	$C_{31}H_{34}O_{14}$	629.1876	Mudanpioside J	599.1794, 507.1469, 477.1548, 461.2407	262350-52-7
68	13.81	485.1012	$[M+Na]^+$	$C_{23}H_{26}O_{10}$	485.1424	Lactiflorin isomer	105.0306	-
69	13.88	167.0588	$[M+H]^+$	$C_9H_{10}O_3$	167.0708	Paeonol	149.0061, 124.8925, 121.0297	552-41-0
70	13.90	485.1012	$[M+Na]^+$	$C_{23}H_{26}O_{10}$	485.1424	Lactiflorin	105.0285	1361049-59-3
71	13.95	983.3507	$[M-H]^-$	$C_{45}H_{60}O_{24}$	983.3402	Acuminatoside	675.2385, 367.1350, 211.0670	142735-71-5
72	13.98	485.1103	$[M+Na]^+$	$C_{23}H_{26}O_{10}$	485.1424	Lactiflorin isomer	105.0285	-
73	14.56	465.2160	$[M-H]^-$	$C_{21}H_{22}O_{12}$	465.2130	Taxifolin-7-O-glucoside	285.1534, 259.1725, 241.1591	14292-40-1
74	14.76	477.1047	$[M+HCOO]^-$	$C_{21}H_{20}O_{10}$	477.1028	Genistin	477.1422, 431.0998,	529-59-9

							301.0376, 269.0456, 167.7634	
75	14.85	599.1844	$[M-H]^-$	$C_{30}H_{32}O_{13}$	599.1770	Mudanpioside C	569.1843, 477.1458, 281.0706, 165.0581, 137.0257	172760-03-1
76	15.34	529.1746	$[M-H]^-$	$C_{27}H_{30}O_{11}$	529.1710	Icariside I	367.1232, 309.0795, 297.0454	56725-99-6
77	16.24	285.0525	$[M+H]^+$	$C_{16}H_{13}O_5$	285.0763	Calycosin	270.0334, 253.0319	20575-57-9
78	16.72	839.2284	$[M+H]^+$	$C_{39}H_{50}O_{20}$	839.2974	Epimedin A	677.1909, 531.1440, 369.1056, 313.0495	110623-72-8
79	16.76	675.2385	$[M-H]^-$	$C_{33}H_{40}O_{15}$	675.2289	Sagittatoside A	367.1193, 351.0492, 323,0949	118525-35-2
80	17.54	839.2284	$[M+H]^+$	$C_{39}H_{50}O_{20}$	839.2974	Epimedin A isomer	677.1855, 531.1440, 369.1056, 313.0458	-
81	17.61	675.2385	[M-H]-	$C_{33}H_{40}O_{15}$	675.2289	Icariin	529.1887, 513.1673, 367.1232	489-32-7
82	18.27	645.2285	$[M-H]^-$	$C_{32}H_{38}O_{14}$	645.2183	Sagittatoside B	366.1156, 351.0903, 323.0986	118525-36-3
83	19.00	823.2366	$[M+Na]^+$	$C_{36}H_{48}O_{20}$	823.2637	Jionoside A1	677.1909	120444-60-2
84	19.05	823.2366	$[M+Na]^+$	$C_{36}H_{48}O_{20}$	823.2637	Jionoside A1 isomer	677.1909	-
85	19.12	867.3004	$[M+HCOO]^-$	$C_{39}H_{50}O_{19}$	867.2928	Baohuoside VI	659.2437, 366.1156, 351.0903, 323.0949	119760-73-5
86	19.14	659.2437	$[M-H]^-$	$C_{33}H_{40}O_{14}$	659.2345	2″-O-rhamnosylicariside II	366.1156, 351.0903, 323.0949	135293-13-9
87	19.48	369.1135	$[M+H]^+$	$C_{21}H_{20}O_6$	369.1338	Icaritin	369.1056, 313.0495, 243.0459, 135.0337	118525-40-9
88	19.58	513.1826	$[M-H]^-$	$C_{27}H_{30}O_{10}$	513.1761	Baohuoside I isomer	366.1156, 351.0903, 323.0949, 217.0528	-
89	5.99	269.0824	$[M-H]^-$	$C_{16}H_{14}O_4$	269.0808	Echinatin	269.1403, 225.1494, 181.1594, 125.0961	34221-41-5
90	20.56	879.3061	$[M-H]^-$	$C_{41}H_{52}O_{21}$	879.2923	Epimedin I	717.2432, 367.1232	205445-00-7
91	22.01	531.1926	$[M-H]^-$	$C_{27}H_{32}O_{11}$	531.1872	Icaritin-3-O-rhamnopyranoside isomer	384.1239, 367.1232, 341.1021, 311.0591	-
92	22.43	717.2488	$[M-H]^-$	$C_{35}H_{42}O_{16}$	717.2395	Sagittatoside C isomer	513.1873, 367.1232	-
93	22.90	687.2371	$[M+HCOO]^-$	$C_{29}H_{38}O_{16}$	687.2142	Isomalto-paeoniflorin	611.2189, 593.1946, 283.0583, 121.0305	262350-54-9
94	23.14	819.2820	$[M-H]^-$	$C_{39}H_{48}O_{19}$	819.2712	Anhydroicaritin-3-O-rhamnoside	529.1793, 367.1232,	-

						(1-2)-furanacid-7-O-glucoside	289.0951	
95	23.95	385.0988	$[M+Na]^+$	$C_{15}H_{22}O_{10}$	385.1111	Catalpol	355.0851, 223.0879, 203.0539	2415-24-9
96	24.09	269.0623	$[M+H]^+$	$C_{16}H_{13}O_4$	269.0814	Formononetin isomer	254.0390, 237.0399	-
97	24.24	829.4650	$[M+HCOO]^-$	$C_{45}H_{56}O_{23}$	829.4586	Astragaloside IV	783.3661	84687-43-4
98	24.369	821.2583	$[M-H]^-$	$C_{39}H_{50}O_{19}$	821.2868	Epimedin C	659.2068, 366.1078, 351.0942, 323.0654, 311.0627	110642-44-9
99	24.43	631.2095	$[M-H]^-$	$C_{31}H_{36}O_{14}$	631.2027	Demethylanhydroicaritin-3-O-rhamnopyranosyl-xylopyranoside	352.1002	-
100	24.90	499.1639	$[M-H]^-$	$C_{26}H_{28}O_{10}$	499.1604	Baohuoside II	353.1037, 352.0963, 295.1040	55395-07-8
101	25.12	675.2385	[M-H]-	$C_{33}H_{40}O_{15}$	675.2289	Icariin isomer	529.1887, 513.1673, 367.1232	-
102	25.12	675.2385	$[M-H]^-$	$C_{33}H_{40}O_{15}$	675.2289	Baohuoside VII	367.1232, 352.1002	119730-89-1
103	25.14	659.2340	$[M-H]^-$	$C_{33}H_{40}O_{14}$	659.2340	2″-O-rhamnosyl-icariside II	366.1156, 351.0942, 323.0912	135293-13-9
104	25.54	659.2345	$[M-H]^-$	$C_{33}H_{40}O_{14}$	659.2437	2″-O-rhamnosyl-icariside II isomer	366.1156, 351.0903, 323.0949	-
105	25.54	479.1330	$[M+H]^+$	$C_{23}H_{26}O_{11}$	479.1553	Curculigoside D	179.0633, 161.0503	-
106	25.71	871.4845	$[M+HCOO]^-$	$C_{43}H_{70}O_{15}$	871.4692	Astragaloside II	825.4771	84676-89-1
107	25.71	871.4845	$[M+HCOO]^-$	$C_{43}H_{70}O_{15}$	871.4692	AstragalosideII isomer	825.4713	-
108	25.74	659.2437	$[M-H]^-$	$C_{33}H_{40}O_{14}$	659.2340	2″-O-rhamnosyl-Icariside II isomer	366.1156, 351.0903, 323.0949	-
109	25.99	717.2488	$[M-H]^-$	$C_{35}H_{42}O_{16}$	717.2395	Sagittatoside C isomer	513.1826, 367.1232	-
110	26.20	313.0495	$[M+H]^+$	$C_{17}H_{12}O_6$	313.0712	Curculigoside A	151.0317	85643-19-2
111	26.21	513.1826	$[M-H]^-$	$C_{27}H_{30}O_{10}$	513.1761	Baohuoside I	366.1156, 351.0903, 323.0949, 217.0528	113558-15-9
112	26.28	913.4961	$[M+HCOO]^{-}$	$C_{45}H_{72}O_{16}$	913.4797	Astragaloside I	867.4873	84680-75-1
113	26.58	913.4900	$[M+HCOO]^{-}$	$C_{45}H_{72}O_{16}$	913.4797	Astragaloside I isomer	867.4813	-
114	27.03	657.2263	$[M-H]^-$	$C_{33}H_{38}O_{14}$	657.2183	Anhydroicaritin-3-O-rhamnopyranosyl-furanacid isomer	513.1826, 367.1193, 352.0963, 289.1229	-
115	27.05	913.4961	$[M+HCOO]^-$	$C_{45}H_{72}O_{16}$	913.4797	Astragaloside I isomer	867.4873	-

2. Components in plasma after KXN administration identified by UPLC/Q-TOF-MS

The preparation of KXN plasma sample is described in the text. Chromatographic condition and Mass spectrum condition UPLC/Q-TOF-MS analysis were consistent with above. The Base Peak Ion (BPI) of KXN plasma in positive and negative ion mode is shown in Supplementary Figure S2.

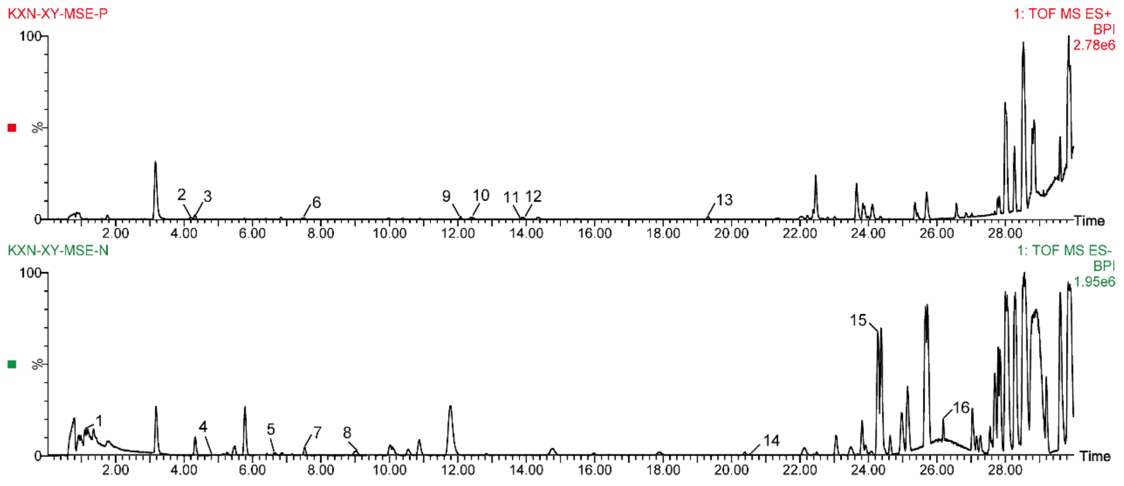

Supplementary Figure S2. BPI diagram of KXN plasma UPLC/Q-TOF-MS in positive and negative ion mode.

Combined with molecular network results and Mass spectrometry information comparison, a total of 16 components in plasma of KXN were determined, the specific information is shown in Supplementary Table S2. The mass spectras of each component based on UPLC/Q-TOF-MS are shown in supplementary data sheet S3.

Supplementary Table S2 16 absorbable components *in vivo* of KXN

No.	t_R/min	Measured value	Precursor ions	Formula	Theoretical value	Compound	Fragment ion	CAS No.
1	0.92	397.0942	$[M-H]^-$	$C_{22}H_{22}O_7$	397.1287	Baohuosu	268.8040, 259.0176, 191.0193	119730-90-4
2	4.30	309.0714	$[M+Na]^+$	$C_{13}H_{18}O_7$	309.0950	Sakakin	125.0573	21082-33-7
3	4.31	125.0504	$[M+H]^+$	$C_7H_8O_2$	125.0603	Guaiacol	110.0286	90-05-1
4	4.84	417.1438	$[M-H]^-$	$C_{22}H_{26}O_8$	417.1549	(-)-Syringaresinol	181.0548	6216-81-5
5	6.68	525.1688	$[M+HCOO]^-$	$C_{23}H_{28}O_{11}$	525.1608	Paeoniflorine	449.1524, 327.1119, 165.0581, 121.0305	23180-57-6
6	7.53	285.0525	$[M+H]^+$	$C_{16}H_{13}O_5$	285.0763	Wogonin	270.0334, 183.0345	632-85-9
7	7.56	283.0618	$[M-H]^-$	$C_{16}H_{12}O_5$	283.0607	Calycosin	268.0391, 239.0355, 211.0403, 195.0477	20575-57-9
8	9.02	463.0896	$[M-H]^-$	$C_{21}H_{20}O_{12}$	463.0877	Hyperoside	300.0280, 271.0277, 255.0323, 151.0040	482-36-0
9	12.11	269.0623	$[M+H]^+$	$C_{16}H_{13}O_4$	269.0814	Formononetin	254.0390, 237.0399	485-72-3
10	12.41	385.0988	$[M+Na]^+$	$C_{15}H_{22}O_{10}$	385.1111	Catalpol	355.0851, 223.0879, 203.0539	2415-24-9
11	15.94	167.0588	$[M+H]^+$	$C_9H_{10}O_3$	167.0708	Paeonol	149.0061, 124.8925, 121.0297	552-41-0
12	15.89	485.1012	$[M+Na]^+$	$C_{23}H_{26}O_{10}$	485.1424	Lactiflorin	105.0285	1361049-59-3
13	19.29	369.1135	$[M+H]^+$	$C_{21}H_{20}O_6$	369.1338	Icaritin	313.2378, 243.1672, 135.1075	118525-40-9
14	20.56	879.3061	$[M-H]^-$	$C_{41}H_{52}O_{21}$	879.2923	Epimedin I	717.2432, 367.1232	205445-00-7
15	24.24	829.4650	$[M+HCOO]^-$	$C_{45}H_{56}O_{23}$	829.4586	Astragaloside IV	783.3661	84687-43-4
16	26.21	513.1826	$[M-H]^-$	$C_{27}H_{30}O_{10}$	513.1761	Baohuoside I	366.1156, 351.0903,	113558-15-9

	323.0949, 217.0528

3. OVX model establishment

3.1 Surgical Method

The OVX rat model was established using 12-week-old female Sprague-Dawley (SD) rats, weighing 200 – 220 g. The rats were anesthetized using isoflurane via an inhalation system. The surgical area was shaved, sterilized, and disinfected. Two dorsolateral incisions were made to expose the ovaries. After the ovaries were ligated with a surgical line, the ovaries were removed with surgical scissors. The muscle and skin layers were sutured and disinfected. During anesthesia, Duratears Ophtalmic Ointment was applied to prevent corneal drying.

3.2 Postoperative Care

Postoperatively, the rats were administered sodium penicillin (5 mg/kg intramuscularly) and ketoprofen (5 mg/kg intramuscularly) for 3 days to prevent infection and manage pain. The rats were closely monitored until they regained consciousness. They were housed at a temperature of 20 – 26°C, with a humidity of 50 ± 10%, and a 12-hour light–dark cycle. Standard rodent diet and water were provided ad libitum.

3.3 Assessment Criteria

The success of the OVX model was assessed through several criteria. Specifically, the estrus cycle of ovariectomized rats was observed by vaginal smear on the 4th day after operation, and the ovariectomized model was initially successful with the disturbance of estrus cycle for 5 consecutive days. The blood of castrated rats was collected from posterior orbital venous plexus, and the levels of E2 and FSH in plasma samples were detected by enzyme-linked immunosorbent assay (ELISA). E2 levels were significantly decreased and FSH levels significantly increased as the criteria for successful modeling.

Vaginal lavage cytology of rats after ovariectomy revealed that the blank control group had a higher number of keratinized cells and a lower number of inflammatory cells. In contrast, the ovariectomized group exhibited a marked reduction in keratinized cells and a significant increase in neutrophils. The rats in the model group remained in a persistent diestrus phase, indicating successful initial model establishment (Supplementary Figure S3). Further detection of plasma E2 and FSH levels in the rats showed that the ovariectomized group had significantly decreased plasma E2 levels and significantly increased FSH levels.

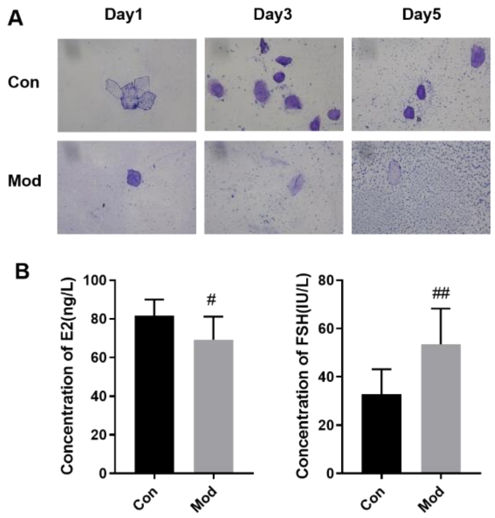

Supplementary Figure S3. Modeling evaluation of PMS rats. **(A)** Estrous cycle observation of vaginal douching smear with Wright-Giemsa; **(B)** Plasma E2 and FSH levels were detected by ELISA. Bars represent the Mean ± SD (n = 10). $^{\#}p < 0.05$, $^{\#\#}p < 0.01$ compared with the Con group.

4. Proteomic detection procedure in adrenal

4.1 Protein Extraction

Samples were first grinded by liquid nitrogen and then the powder was transferred to a 1.5 mL centrifuge tube and sonicated three times on ice, using a high intensity ultrasonic processor in a lysis buffer (8M urea including 1mM PMSF、2mM EDTA). After, the remaining debris was removed by centrifugation at 15000g at 4°C for 10 min. Finally, the protein concentration was determined with a BCA kit according to the instructions of the manufacturer.

4.2 Digestion and Cleanup

Equal amount of proteins from each sample were used for tryptic digestion. Add 8M urea to 200ul to the supernatants, then reduced with 10 mM DTT for 45 minutes at 37°C and alkylated with 50 mM iodoacetamide (IAM) for 15 minutes in a dark room at room temperature. 4 × volume of chilled acetone was added and precipitated at -20°C for 2 hours. After centrifugation, the protein precipitate was air-dried and resuspended in 200 μL of 25 mM ammonium bicarbonate solution and 3ul of trypsin (Promega) and digested overnight at 37°C. After digestion, peptides were desalted using C18 Cartridge followed by drying with Vacuum concentration meter, concentrated by vacuum centrifugation and redissolved in 0.1% (v/V) formic acid.

4.3 LC-MS/MS Analysis

Liquid chromatography (LC) was performed on a nanoElute UHPLC (Bruker Daltonics, Germany). About 200 ng peptides were separated within 40 min at a flow rate of 0.3 μL/min on a commercially available reverse-phase C18 column with an integrated CaptiveSpray Emitter (25 cm x 75 μm ID, 1.6 μm, Aurora Series with CSI, IonOpticks, Australia).The separation temperature was

kept by an integrated Toaster column oven at 50°C. Mobile phases A and B were produced with 0.1 vol.-% formic acid in water and 0.1% formic acid in ACN. Mobile phase B was increased from 2 to 22% over the first 25 min, increased to 35% over the next 5 min, further increased to 80% over the next 5 min, and then held at 80% for 5 min. The LC was coupled online to a hybrid timsTOF Pro2 (Bruker Daltonics, Germany) via a CaptiveSpray nano-electrospray ion source (CSI).To establish the applicable acquisition windws for diaPASEF mode, the timsTOF Pro2 was operated in Data-Dependent Parallel Accumulation-Serial Fragmentation (PASEF) mode with 4 PASEF MS/MS frames in 1 complete frame. The capillary voltage was set to 1500 V, and the MS and MS/MS spectra were acquired from 100 to 1700 m/z. As for ion mobility range ($1/K_0$), 0.85 to 1.3 Vs/cm^2 was used. The "target value" of 10,000 was applied to a repeated schedule, and the intensity threshold was set at 1500. The collision energy was ramped linearly as a function of mobility from 45eV at $1/K_0$ = 1.3 Vs/cm^2 to 27 eV at $1/K_0$ = 0.85 Vs/cm^2. The quadrupole isolation width was set to 2Th for m/z < 700 and 3Th for m/z > 800.

In diaPASEF mode, the instrument control software was extended to define quadrupole isolation windows as a function of the TIMS scan time. Seamless and synchronous ramping of all applied voltage is achieved by modifying the instrument control electronics. We defined 25 Th isolation windows from m/z about 400 to 1200 and totally 48windows were defined. Other parameters were the same as DDA-PASEF mode.

4.4 Database search and quantification

MS raw data were analyzed using DIA-NN (v1.8.1) with library-free method. the uniprot-proteome_UP000002494_Rat_20220719.fasta database (A total of 46069 sequences) was used to create a spectra library with deep learning algrithms of neural networks. the option of MBR was employed to create a spectral library from DIA data and then reanlyse using this library. The false discovery rate (FDR) of search results was adjusted to < 1% at both protein and precursor ion levels, the remaining identifications were used for further quantification analysis.

5. Proteomic analysis of the effects of KXN on proteins in OVX rats

Differences and similarities between the control group (Con), OVX model group (Mod) and KXN administration group (KXN) were evaluated by using OPLS-DA (Supplementary Figure S4). It was found that Mod was significantly separated from the Con group, while KXN group converged toward CON group and separated from Mod group.

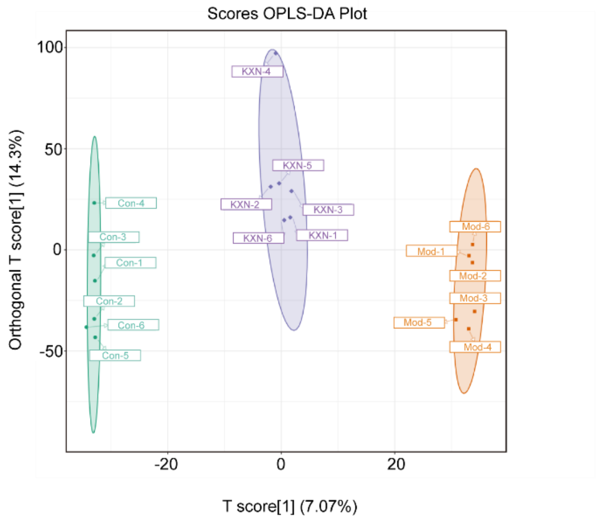

Supplementary Figure S4. OPLS-DA plot of the control group (Con), OVX model group (Mod) and KXN administration group (KXN).

Based on OPLS-DA model, peak features that met the screening criteria for both multivariate statistical analysis (VIP > 1) and univariate statistical analysis ($p < 0.05$ and fold change ≥ 1.5 or ≤ 0.6667) were selected as the significant differential proteins. The differences in the expression levels of the different proteins in the two groups of samples and the statistical significance of the differences were presented in the volcano maps (Supplementary Figure S5).

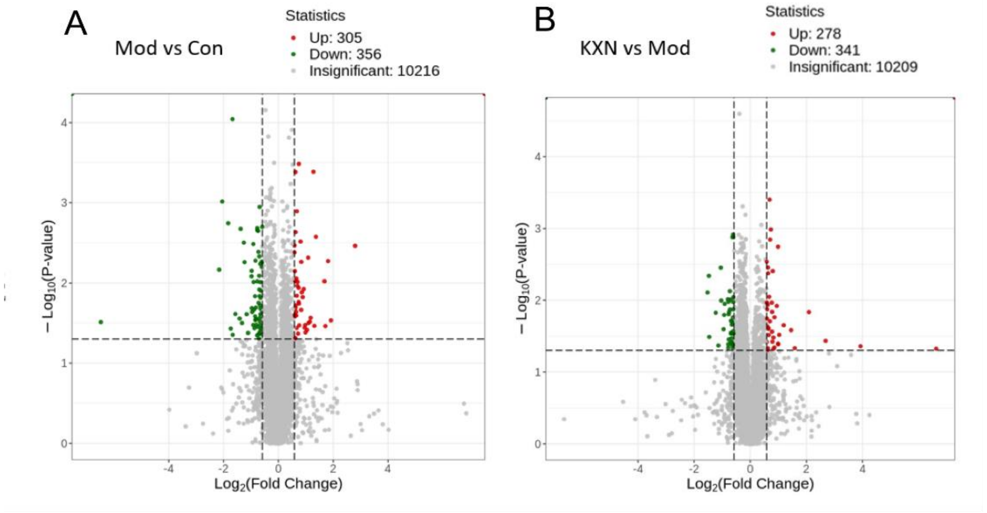

Supplementary Figure S5. (A) Volcano diagram depicted the differential expression proteins in OVX model rats versus control rats. **(B)** Volcano diagram depicted the differential expression proteins in KXN administration rats versus OVX model rats. Green, down-regulation; red, up-regulation.

Utilizing z-score normalization of a range of proteins, we generated a clustering heat map to elucidate the expression profiles of distinct proteins across various samples (Supplementary Figure S6).

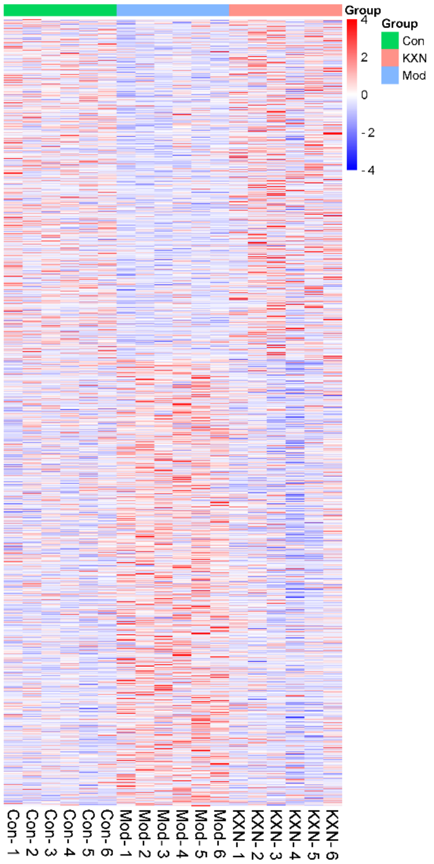

Supplementary Figure S6. Heatmap visualized the differentially expressed proteins with $p < 0.05$ and fold change ≥ 1.5 or ≤ 0.6667.

6. Quantitative analysis of representative associated proteins in steroid hormone biosynthesis pathway

Compared with OVX model group, the expression of HSD3B, CYP21A2, StAR, HSD11B2, which are representative of steroid hormone biosynthesis, was reversed after KXN treatment (Supplementary Figure S7).

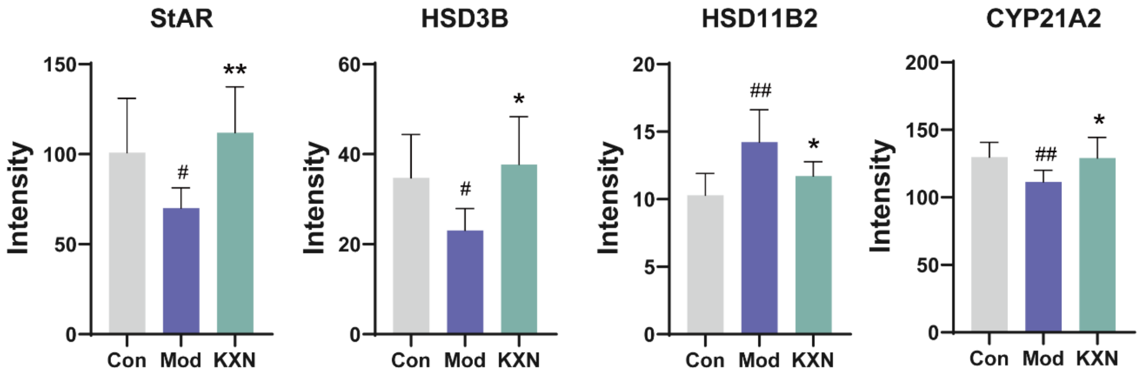

Supplementary Figure S7. Quantification of steroid hormone biosynthesis associated proteins detected by adrenal proteomics. Bars represent the Mean ± SD (n = 6). $^{\#}p < 0.05$, $^{\#\#}p < 0.01$ compared with the Con group; $^{*}p < 0.05$, $^{**}p < 0.01$ compared with the Mod group.

7. Metabolomics analysis of the effects of KXN on metabolites in OVX rats

The OPLS-DA plot demonstrated a clear separation between the Mod group and the Con group, indicating a significant alteration in either the type or level of metabolites. Following drug intervention, the KXN group exhibited distinct separation from the Mod group, suggesting that KXN effectively regulates metabolic levels in OVX rats (Supplementary Figure S8).

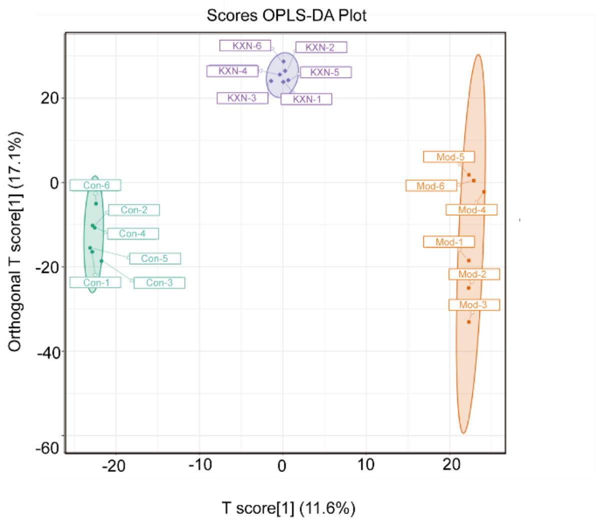

Supplementary Figure S8. OPLS-DA plot of the Con, Mod and KXN in metabolomics analysis.

Based on OPLS-DA model, peak features that met the screening criteria for both multivariate statistical analysis (VIP > 1) and univariate statistical analysis (p < 0.05) were selected as the significant differential metabolites. The relative content difference of metabolites in the two groups of samples and the statistical significance of the difference are shown in the Volcano Plot (Supplementary Figure S9).

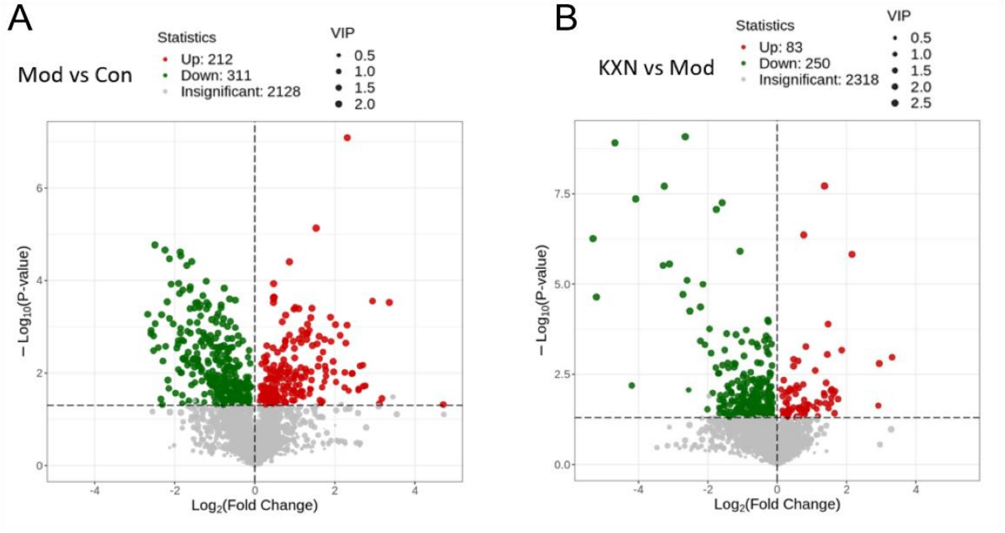

Supplementary Figure S9. (A) Volcano diagram depicted the differential metabolites in OVX model rats versus control rats. **(B)** Volcano diagram depicted the differential metabolites in KXN administration rats versus OVX model rats. Green, down-regulation; red, up-regulation.

8. The expression of CYP19A1 rat adrenal, uterus, hypothalamus tissues

The expression levels of CYP19A1 in rat adrenal, uterus, hypothalamus tissues were detected by ELISA. The result showed that KXN improve the expression of CYP19A1 in rat adrenal, uterus, hypothalamus tissues (Supplementary Figure S10).

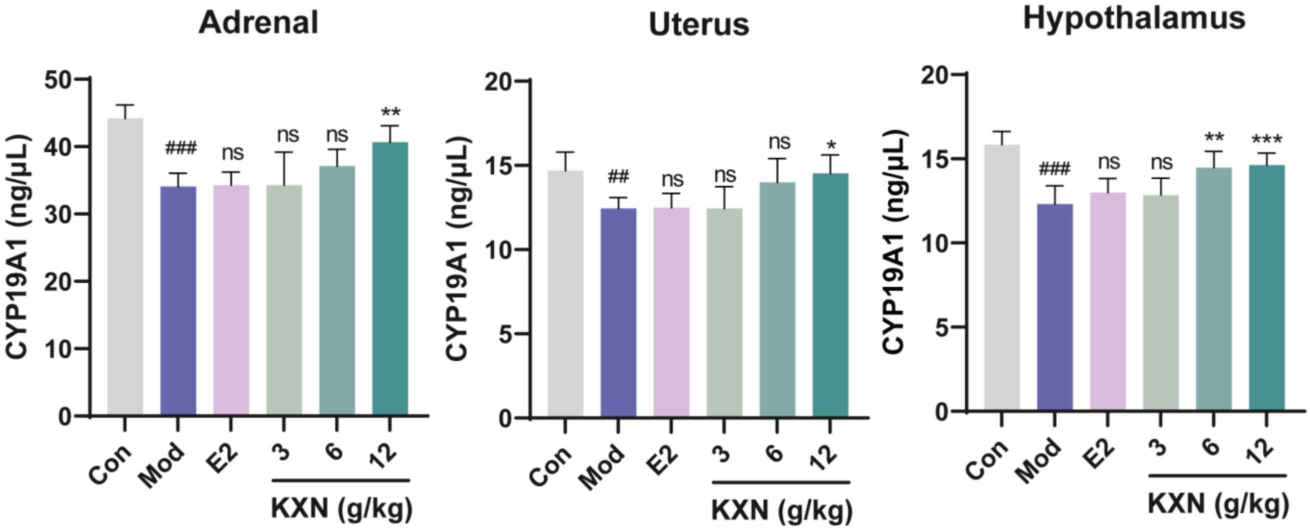

Supplementary Figure S10. KXN increase the expression of CYP19A1 in rat adrenal, uterus, hypothalamus tissues. Bars represent the Mean ± SD (n = 6). $^{##}p< 0.01$, $^{###}p < 0.001$ compared with the Con group. $^{*}p < 0.05$, $^{**}p < 0.01$, $^{***}p < 0.001$ compared with the Mod group; ns indicates no significant difference compared with the Mod group.

9. siRNA-mediated knockdown of CYP19A1 in H295R cells.

A knockdown of CYP19A1 in H295R cells to verify the necessity of CYP19A1 in the mechanism of KXN action. Four siCYP19A1 targeting different gene regions were designed, and WB verification found that SICYP19A1-4 has the best knockdown effect (Supplementary Figure S11A). Treatment with siCYP19A1-4 and other drugs had no effect on cell viability (Supplementary Figure S11B). Notably, after CYP19A1 knockout, the promoting effects of KXN and its three active components (astragaloside IV, icaritin, and baohuoside I) on estradiol secretion were significantly inhibited (Supplementary Material Figure S11C).

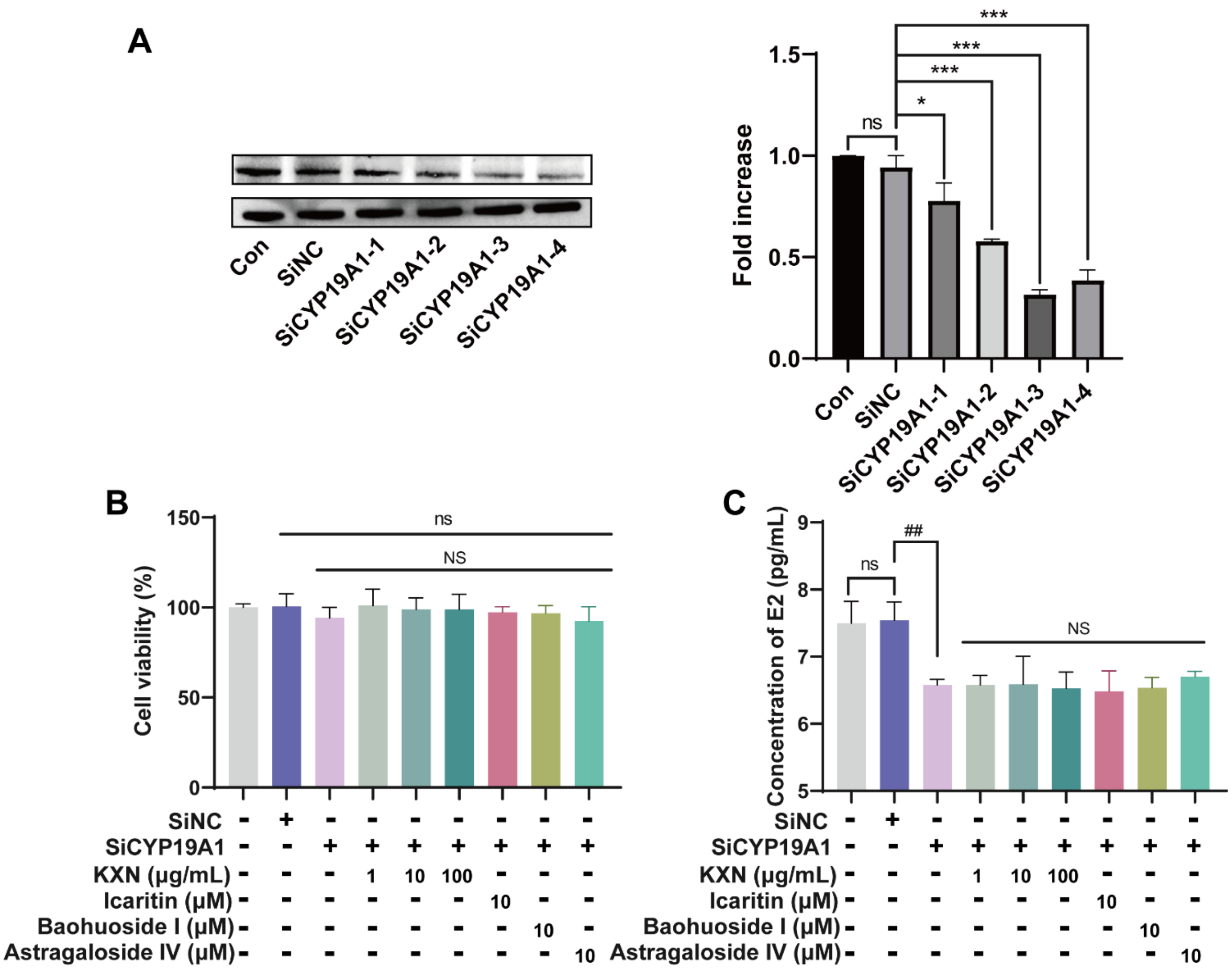

Supplementary Figure S11. KXN and its active components were unable to promote estradiol secretion in the absence of CYP19A1. **(A)** The knockout effect of four kinds of SiCYP19A1 in H295R cells by western blot. Bars represent the Mean ± SD (n = 3). ns indicates no significant difference compared with the Con group; $^{*}p < 0.05$, $^{***}p < 0.001$ compared with the SiNC group. **(B)** The cell viability of each group was insignificant after SiCYP19A1 treatmen in H295R cells. Bars represent the Mean ± SD (n = 3). ns indicates no significant difference compared with the Con group; NS indicates no significant difference compared with the SiNC group. **(C)** The promotion effect of KXN and its active components on estradiol secretion was abolished after CYP19A1 knockdown. Bars represent the Mean ± SD (n = 3). ns indicates no significant difference compared with the Con group; $^{##}p < 0.01$ compared with the SiNC group; NS indicates no significant difference compared with the CYP19A1 knockdown group.
