# Supplementary material for: Kunxinning granules alleviate perimenopausal syndrome by supplementing estrogen deficiency
Source: Front Pharmacol. 2025 Mar 26;16:1554479. doi: 10.3389/fphar.2025.1554479 (PMC11979375; doi:10.3389/fphar.2025.1554479)
Supplement: Supplementary file 5 [file DataSheet5.pdf]

Original data and significance analysis of all graphs in the manuscript:

Figure 1A

Con	Mod	E2	3 g/kg	6 g/kg	12 g/kg
0.169591	0.033183	0.064205	0.0452	0.046571	0.055904
0.209524	0.038167	0.0422	0.018992	0.044226	0.056122
0.244275	0.035486	0.065603	0.047101	0.040179	0.064615
0.145973	0.031082	0.071944	0.039121	0.034023	0.043722
0.257031	0.037219	0.089557	0.046853	0.035135	0.048462
0.160256	0.031889	0.052985	0.037647	0.039205	0.049725

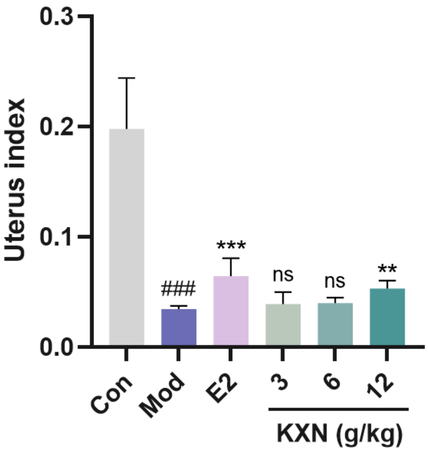

Unpaired t test
Tabular results

1	Table Analyzed	Uterus index
2		
3	Column B	Mod
4	vs.	vs.
5	Column A	Con
6		
7	**Unpaired t test**	
8	P value	<0.0001
9	P value summary	****
10	Significantly different (P < 0.05)?	Yes
11	One- or two-tailed P value?	Two-tailed
12	t, df	t=8.630, df=10
13		
14	**How big is the difference?**	
15	Mean of column A	0.1978
16	Mean of column B	0.03450
17	Difference between means (B - A) ± SEM	-0.1633 ± 0.01892
18	95% confidence interval	-0.2054 to -0.1211
19	R squared (eta squared)	0.8816

Ordinary one-way ANOVA
Multiple comparisons

1	Number of families	1				
2	Number of comparisons per family	4				
3	Alpha	0.05				
4						
5	**Dunnett's multiple comparisons test**	**Mean Diff.**	**95.00% CI of diff.**	**Below threshold?**	**Summary**	**Adjusted P Value**
6	Mod vs. E2	-0.02991	-0.04440 to -0.01542	Yes	****	<0.0001
7	Mod vs. 3	-0.004648	-0.01913 to 0.009838	No	ns	0.8181
8	Mod vs. 6	-0.005385	-0.01987 to 0.009101	No	ns	0.7346
9	Mod vs. 12	-0.01859	-0.03307 to -0.004101	Yes	**	0.0092
10						
11	**Test details**	**Mean 1**	**Mean 2**	**Mean Diff.**	**SE of diff.**	**n1**
12	Mod vs. E2	0.03450	0.06442	-0.02991	0.005557	6
13	Mod vs. 3	0.03450	0.03915	-0.004648	0.005557	6
14	Mod vs. 6	0.03450	0.03989	-0.005385	0.005557	6
15	Mod vs. 12	0.03450	0.05309	-0.01859	0.005557	6

Figure 1B Endometrial thickness

Con	Mod	E2	3 g/kg	6 g/kg	12 g/kg
1695.0760	470.1085	1068.7900	563.5905	567.9988	666.9434
1541.2600	592.9658	740.8469	602.2489	601.4534	692.7456
1453.1120	349.1581	836.1333	538.7459	633.9563	719.3886
1484.8060	464.5740	890.4351	582.9291	743.1830	685.7125
1946.4540	511.6885	811.5062	607.7424	749.3059	787.8463
1683.6660	596.2105	804.7989	676.5074	678.4441	889.3329

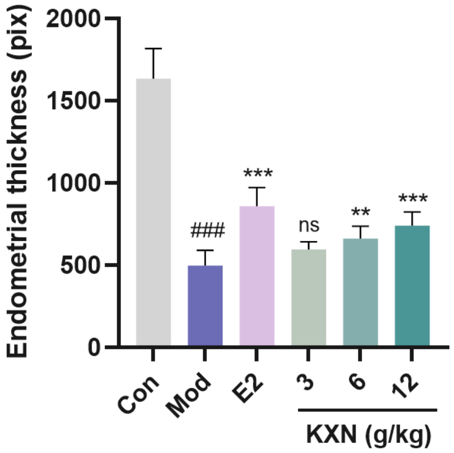

	Unpaired t test<br>Tabular results	
1	Table Analyzed	Endometrial thickness
2		
3	Column B	Mod
4	vs.	vs.
5	Column A	Con
6		
7	**Unpaired t test**	
8	P value	<0.0001
9	P value summary	****
10	Significantly different (P < 0.05)?	Yes
11	One- or two-tailed P value?	Two-tailed
12	t, df	t=13.58, df=10
13		
14	**How big is the difference?**	
15	Mean of column A	1634
16	Mean of column B	497.5
17	Difference between means (B - A) ± SE	-1137 ± 83.73
18	95% confidence interval	-1323 to -950.1
19	R squared (eta squared)	0.9485

	Ordinary one-way ANOVA<br>Multiple comparisons					
1	Number of families	1				
2	Number of comparisons per family	4				
3	Alpha	0.05				
4						
5	**Dunnett's multiple comparisons test**	**Mean Diff.**	**95.00% CI of diff.**	**Below threshold?**	**Summary**	**Adjusted P Value**
6	Mod vs. E2	-361.3	-489.7 to -232.9	Yes	****	<0.0001
7	Mod vs. 3	-97.84	-226.3 to 30.60	No	ns	0.1735
8	Mod vs. 6	-164.9	-293.4 to -36.50	Yes	**	0.0091
9	Mod vs. 12	-242.9	-371.3 to -114.4	Yes	***	0.0002
10						
11	**Test details**	**Mean 1**	**Mean 2**	**Mean Diff.**	**SE of diff.**	**n1**
12	Mod vs. E2	497.5	858.8	-361.3	49.27	6
13	Mod vs. 3	497.5	595.3	-97.84	49.27	6
14	Mod vs. 6	497.5	662.4	-164.9	49.27	6
15	Mod vs. 12	497.5	740.3	-242.9	49.27	6

Figure 1B Endometrial area

Con	Mod	E2	3 g/kg	6 g/kg	12 g/kg
13208496	884388	3885753	1799226	2083669	2045068
13792544	1520806	3310324	2608218	2431842	2489060
9982116	1528406	3138320	2070116	2165182	3337612
10666374	2063212	3292659	3001396	2200405	2183844
8006412	1806180	4730306	2563931	2843852	3643548
12791835	1562303	3166602	2238277	2433885	3232387

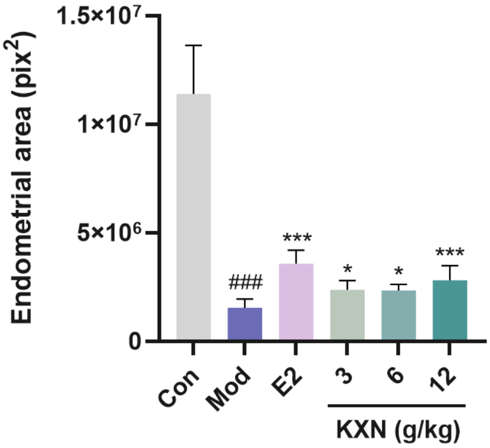

Unpaired t test
Tabular results

1	Table Analyzed	Endometrial area
2		
3	Column B	Mod
4	vs.	vs.
5	Column A	Con
6		
7	**Unpaired t test**	
8	P value	<0.0001
9	P value summary	****
10	Significantly different (P < 0.05)?	Yes
11	One- or two-tailed P value?	Two-tailed
12	t, df	t=10.62, df=10
13		
14	**How big is the difference?**	
15	Mean of column A	11407963
16	Mean of column B	1560883
17	Difference between means (B - A) ± SEN	-9847080 ± 926815
18	95% confidence interval	-11912154 to -7782007
19	R squared (eta squared)	0.9186

Ordinary one-way ANOVA
Multiple comparisons

1	Number of families	1				
2	Number of comparisons per family	4				
3	Alpha	0.05				
4						
5	**Dunnett's multiple comparisons test**	**Mean Diff.**	**95.00% CI of diff.**	**Below threshold?**	**Summary**	**Adjusted P Value**
6	Mod vs. E2	-2026445	-2779129 to -1273761	Yes	****	<0.0001
7	Mod vs. 3	-819312	-1571996 to -66627	Yes	*	0.0300
8	Mod vs. 6	-798923	-1551607 to -46239	Yes	*	0.0352
9	Mod vs. 12	-1261037	-2013721 to -508353	Yes	***	0.0007
10						
11	**Test details**	**Mean 1**	**Mean 2**	**Mean Diff.**	**SE of diff.**	**n1**
12	Mod vs. E2	1560883	3587327	-2026445	288706	6
13	Mod vs. 3	1560883	2380194	-819312	288706	6
14	Mod vs. 6	1560883	2359806	-798923	288706	6
15	Mod vs. 12	1560883	2821920	-1261037	288706	6

Figure 1C

Con	Mod	E2	3 g/kg	6 g/kg	12 g/kg
102.51	23.43	92.23	24.42	36.03	55.13
83.26	29.77	72.96	29.06	39.64	51.04
104.53	26.74	90.57	29.75	40.06	53.13
93.68	27.04	83.74	29.01	42.72	46.55
88.87	24.13	83.77	31.47	30.43	49.02
104.85	23.40	88.60	31.30	36.92	49.62

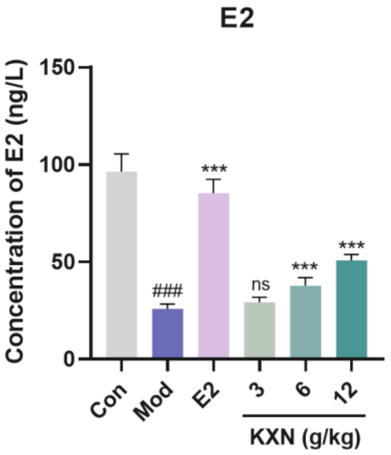

Unpaired t test
Tabular results

1	Table Analyzed	E2
2		
3	Column B	Mod
4	vs.	vs.
5	Column A	Con
6		
7	**Unpaired t test**	
8	P value	<0.0001
9	P value summary	****
10	Significantly different (P < 0.05)?	Yes
11	One- or two-tailed P value?	Two-tailed
12	t, df	t=18.34, df=10
13		
14	**How big is the difference?**	
15	Mean of column A	96.28
16	Mean of column B	25.75
17	Difference between means (B - A) ± SEM	-70.53 ± 3.846
18	95% confidence interval	-79.10 to -61.96
19	R squared (eta squared)	0.9711

Ordinary one-way ANOVA
Multiple comparisons

1	Number of families	1				
2	Number of comparisons per family	4				
3	Alpha	0.05				
4						
5	**Dunnett's multiple comparisons test**	**Mean Diff.**	**95.00% CI of diff.**	**Below threshold?**	**Summary**	**Adjusted P Value**
6	Mod vs. E2	-59.56	-65.92 to -53.20	Yes	****	<0.0001
7	Mod vs. 3	-3.415	-9.776 to 2.946	No	ns	0.4475
8	Mod vs. 6	-11.88	-18.24 to -5.516	Yes	***	0.0002
9	Mod vs. 12	-24.99	-31.35 to -18.63	Yes	****	<0.0001
10						
11	**Test details**	**Mean 1**	**Mean 2**	**Mean Diff.**	**SE of diff.**	**n1**
12	Mod vs. E2	25.75	85.31	-59.56	2.440	6
13	Mod vs. 3	25.75	29.17	-3.415	2.440	6
14	Mod vs. 6	25.75	37.63	-11.88	2.440	6
15	Mod vs. 12	25.75	50.75	-24.99	2.440	6

Figure 1D

Con	Mod	E2	3 g/kg	6 g/kg	12 g/kg
19.97	42.57	22.91	29.79	28.69	27.32
20.43	38.60	19.03	30.31	29.26	22.34
21.14	35.47	20.14	27.81	29.06	23.51
21.68	41.01	20.11	27.91	27.31	25.27
20.97	41.26	20.33	31.57	25.76	24.08
17.79	39.63	23.45	30.06	29.04	27.47

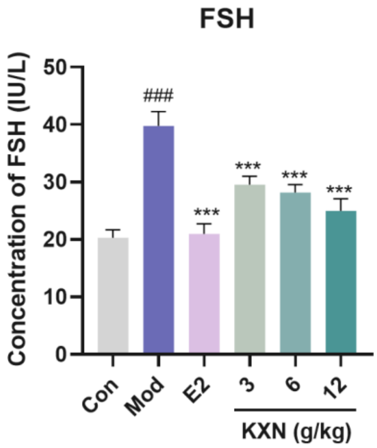

Unpaired t test
Tabular results

1	Table Analyzed	FSH
2		
3	Column B	Mod
4	vs.	vs.
5	Column A	Con
6		
7	**Unpaired t test**	
8	P value	<0.0001
9	P value summary	****
10	Significantly different (P < 0.05)?	Yes
11	One- or two-tailed P value?	Two-tailed
12	t, df	t=16.63, df=10
13		
14	**How big is the difference?**	
15	Mean of column A	20.33
16	Mean of column B	39.76
17	Difference between means (B - A) ± SEM	19.43 ± 1.168
18	95% confidence interval	16.82 to 22.03
19	R squared (eta squared)	0.9651

Ordinary one-way ANOVA
Multiple comparisons

1	Number of families	1				
2	Number of comparisons per family	4				
3	Alpha	0.05				
4						
5	**Dunnett's multiple comparisons test**	**Mean Diff.**	**95.00% CI of diff.**	**Below threshold?**	**Summary**	**Adjusted P Value**
6	Mod vs. E2	18.76	15.92 to 21.60	Yes	****	<0.0001
7	Mod vs. 3	10.18	7.341 to 13.02	Yes	****	<0.0001
8	Mod vs. 6	11.57	8.730 to 14.41	Yes	****	<0.0001
9	Mod vs. 12	14.76	11.92 to 17.59	Yes	****	<0.0001
10						
11	**Test details**	**Mean 1**	**Mean 2**	**Mean Diff.**	**SE of diff.**	**n1**
12	Mod vs. E2	39.76	21.00	18.76	1.089	6
13	Mod vs. 3	39.76	29.58	10.18	1.089	6
14	Mod vs. 6	39.76	28.19	11.57	1.089	6
15	Mod vs. 12	39.76	25.00	14.76	1.089	6

Figure 1E

Con	Mod	E2	3 g/kg	6 g/kg	12 g/kg
23.40	20.93	20.88	23.24	21.80	20.70
23.84	18.63	24.21	20.54	22.07	22.68
28.17	22.69	22.93	15.24	21.93	23.44
26.84	17.88	21.51	21.01	22.15	24.23
29.07	21.40	21.95	26.66	23.00	24.60
23.15	17.87	22.05	21.66	21.96	23.43

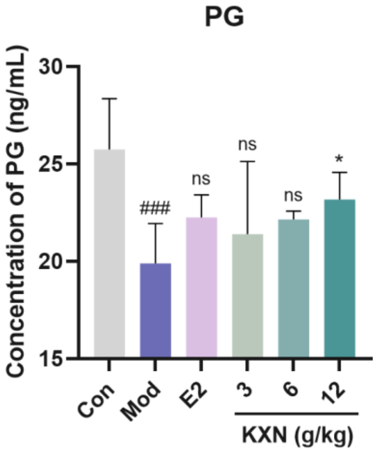

Unpaired t test
Tabular results

1	Table Analyzed	PG
2		
3	Column B	Mod
4	vs.	vs.
5	Column A	Con
6		
7	**Unpaired t test**	
8	P value	0.0015
9	P value summary	**
10	Significantly different (P < 0.05)?	Yes
11	One- or two-tailed P value?	Two-tailed
12	t, df	t=4.321, df=10
13		
14	**How big is the difference?**	
15	Mean of column A	25.75
16	Mean of column B	19.90
17	Difference between means (B - A) ± SE[illegible]	-5.846 ± 1.353
18	95% confidence interval	-8.860 to -2.831
19	R squared (eta squared)	0.6512

Ordinary one-way ANOVA
Multiple comparisons

1	Number of families	1				
2	Number of comparisons per family	4				
3	Alpha	0.05				
4						
5	**Dunnett's multiple comparisons test**	**Mean Diff.**	**95.00% CI of diff.**	**Below threshold?**	**Summary**	**Adjusted P Value**
6	Mod vs. E2	-2.353	-5.485 to 0.7797	No	ns	0.1826
7	Mod vs. 3	-1.492	-4.625 to 1.640	No	ns	0.5493
8	Mod vs. 6	-2.251	-5.384 to 0.8814	No	ns	0.2122
9	Mod vs. 12	-3.280	-6.412 to -0.1473	Yes	*	0.0382
10						
11	**Test details**	**Mean 1**	**Mean 2**	**Mean Diff.**	**SE of diff.**	**n1**
12	Mod vs. E2	19.90	22.25	-2.353	1.202	6
13	Mod vs. 3	19.90	21.39	-1.492	1.202	6
14	Mod vs. 6	19.90	22.15	-2.251	1.202	6
15	Mod vs. 12	19.90	23.18	-3.280	1.202	6

Figure 1F

Con	Mod	E2	3 g/kg	6 g/kg	12 g/kg
2732.60	7154.12	4468.65	2678.36	3398.90	5028.05
900.67	7651.62	4807.67	5677.52	3739.64	4237.66
3370.96	5081.70	3700.15	4646.01	4379.94	2358.02
2974.79	6479.31	5295.87	4197.86	5022.12	5063.75
2176.29	5765.25	4695.59	6166.16	6166.16	4779.40
2456.05	6602.56	3645.94	6343.68	5451.75	3722.04

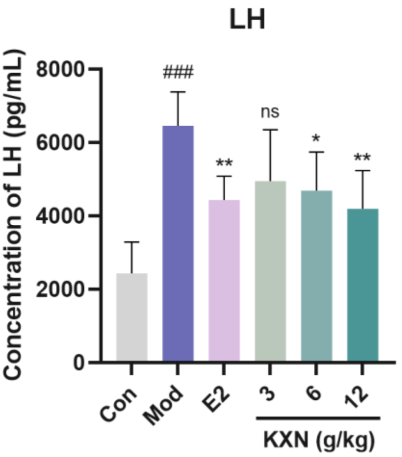

Unpaired t test
Tabular results

1	Table Analyzed	LH
2		
3	Column B	Mod
4	vs.	vs.
5	Column A	Con
6		
7	**Unpaired t test**	
8	P value	<0.0001
9	P value summary	****
10	Significantly different (P < 0.05)?	Yes
11	One- or two-tailed P value?	Two-tailed
12	t, df	t=7.794, df=10
13		
14	**How big is the difference?**	
15	Mean of column A	2435
16	Mean of column B	6456
17	Difference between means (B - A) ± SEM	4021 ± 515.8
18	95% confidence interval	2871 to 5170
19	R squared (eta squared)	0.8587

Ordinary one-way ANOVA
Multiple comparisons

1	Number of families	1				
2	Number of comparisons per family	4				
3	Alpha	0.05				
4						
5	**Dunnett's multiple comparisons test**	**Mean Diff.**	**95.00% CI of diff.**	**Below threshold?**	**Summary**	**Adjusted P Value**
6	Mod vs. E2	2020	452.6 to 3588	Yes	**	0.0088
7	Mod vs. 3	1504	-63.32 to 3072	No	ns	0.0626
8	Mod vs. 6	1763	195.2 to 3330	Yes	*	0.0243
9	Mod vs. 12	2258	690.1 to 3825	Yes	**	0.0034
10						
11	**Test details**	**Mean 1**	**Mean 2**	**Mean Diff.**	**SE of diff.**	**n1**
12	Mod vs. E2	6456	4436	2020	601.2	6
13	Mod vs. 3	6456	4952	1504	601.2	6
14	Mod vs. 6	6456	4693	1763	601.2	6
15	Mod vs. 12	6456	4198	2258	601.2	6

Figure 5B Progesterone

Con	Mod	KXN
34181.61	1225.05	5228.58
14802.97	2675.42	7595.14
20568.33	2203.92	4300.81
27765.94	4500.81	4422.2
12444.33	2095.96	5921.9
20635.85	1684.34	5543.85

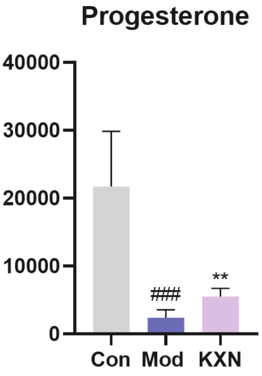

	Unpaired t test<br>Tabular results	
1	Table Analyzed	Progesterone
2		
3	Column B	Mod
4	vs.	vs.
5	Column A	Con
6		
7	**Unpaired t test**	
8	P value	0.0002
9	P value summary	***
10	Significantly different (P < 0.05)?	Yes
11	One- or two-tailed P value?	Two-tailed
12	t, df	t=5.788, df=10
13		
14	**How big is the difference?**	
15	Mean of column A	21733
16	Mean of column B	2398
17	Difference between means (B - A) ± SEM	-19336 ± 3340
18	95% confidence interval	-26778 to -11893
19	R squared (eta squared)	0.7701

	Unpaired t test<br>Tabular results	
1	Table Analyzed	Progesterone
2		
3	Column C	KXN
4	vs.	vs.
5	Column B	Mod
6		
7	**Unpaired t test**	
8	P value	0.0010
9	P value summary	**
10	Significantly different (P < 0.05)?	Yes
11	One- or two-tailed P value?	Two-tailed
12	t, df	t=4.585, df=10
13		
14	**How big is the difference?**	
15	Mean of column B	2398
16	Mean of column C	5502
17	Difference between means (C - B) ± SEM	3104 ± 677.1
18	95% confidence interval	1596 to 4613
19	R squared (eta squared)	0.6776

Figure 5B 11-Deoxycortisol

Con	Mod	KXN
9850.86	2034.89	8578.57
16315.81	4350.35	9931.54
23705.12	7298.88	23388.41
11438.87	4299.27	24812.8
17529.63	5896.12	11861.92
15175.32	12067.81	10846.63

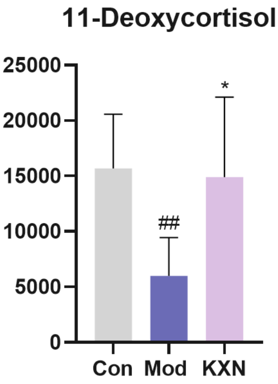

	Unpaired t test<br>Tabular results	
1	Table Analyzed	11-Deoxycortisol
2		
3	Column B	Mod
4	vs.	vs.
5	Column A	Con
6		
7	**Unpaired t test**	
8	P value	0.0027
9	P value summary	**
10	Significantly different (P < 0.05)?	Yes
11	One- or two-tailed P value?	Two-tailed
12	t, df	t=3.948, df=10
13		
14	**How big is the difference?**	
15	Mean of column A	15669
16	Mean of column B	5991
17	Difference between means (B - A) ± SEM	-9678 ± 2452
18	95% confidence interval	-15140 to -4216
19	R squared (eta squared)	0.6091

	Unpaired t test<br>Tabular results	
1	Table Analyzed	11-Deoxycortisol
2		
3	Column C	KXN
4	vs.	vs.
5	Column B	Mod
6		
7	**Unpaired t test**	
8	P value	0.0213
9	P value summary	*
10	Significantly different (P < 0.05)?	Yes
11	One- or two-tailed P value?	Two-tailed
12	t, df	t=2.727, df=10
13		
14	**How big is the difference?**	
15	Mean of column B	5991
16	Mean of column C	14903
17	Difference between means (C - B) ± SEM	8912 ± 3268
18	95% confidence interval	1630 to 16195
19	R squared (eta squared)	0.4264

Figure 5B Cortisol

Con	Mod	KXN
13082.39	3505.46	5287.94
24939.23	6839.8	5860.89
18324.32	2817.36	6299.01
16041.72	5047.56	6560.6
11559.87	3780.52	5790.1
14389.46	4246.78	7309.08

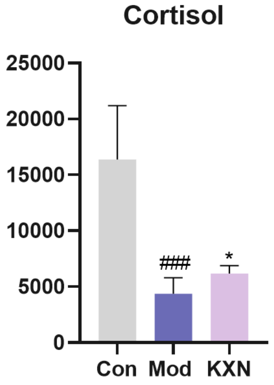

	Unpaired t test Tabular results	
1	Table Analyzed	Cortisol
2		
3	Column B	Mod
4	vs.	vs.
5	Column A	Con
6		
7	**Unpaired t test**	
8	P value	0.0002
9	P value summary	***
10	Significantly different (P < 0.05)?	Yes
11	One- or two-tailed P value?	Two-tailed
12	t, df	t=5.880, df=10
13		
14	**How big is the difference?**	
15	Mean of column A	16389
16	Mean of column B	4373
17	Difference between means (B - A) ± SEM	-12017 ± 2044
18	95% confidence interval	-16570 to -7463
19	R squared (eta squared)	0.7756

	Unpaired t test Tabular results	
1	Table Analyzed	Cortisol
2		
3	Column C	KXN
4	vs.	vs.
5	Column B	Mod
6		
7	**Unpaired t test**	
8	P value	0.0188
9	P value summary	*
10	Significantly different (P < 0.05)?	Yes
11	One- or two-tailed P value?	Two-tailed
12	t, df	t=2.800, df=10
13		
14	**How big is the difference?**	
15	Mean of column B	4373
16	Mean of column C	6185
17	Difference between means (C - B) ± SEM	1812 ± 647.1
18	95% confidence interval	369.8 to 3254
19	R squared (eta squared)	0.4394

Figure 5B 11-Dehydrocorticosterone

Con	Mod	KXN
9219.86	21596.71	4098.57
12790.94	19177.46	5718.1
4321.22	27802.13	9510.49
12943.37	25228.62	4601.09
25615.84	17154.79	14964.71
13969.5	19488	6145.38

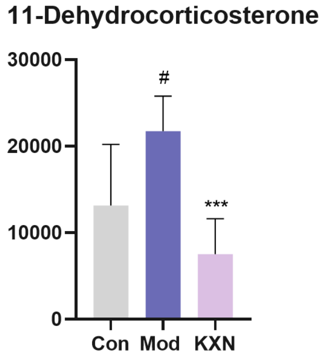

	Unpaired t test Tabular results	
1	Table Analyzed	11-Dehydrocorticosterone
2		
3	Column B	Mod
4	vs.	vs.
5	Column A	Con
6		
7	**Unpaired t test**	
8	P value	0.0271
9	P value summary	*
10	Significantly different (P < 0.05)?	Yes
11	One- or two-tailed P value?	Two-tailed
12	t, df	t=2.588, df=10
13		
14	**How big is the difference?**	
15	Mean of column A	13143
16	Mean of column B	21741
17	Difference between means (B - A) ± SEM	8598 ± 3323
18	95% confidence interval	1194 to 16002
19	R squared (eta squared)	0.4010

	Unpaired t test Tabular results	
1	Table Analyzed	11-Dehydrocorticosterone
2		
3	Column C	KXN
4	vs.	vs.
5	Column B	Mod
6		
7	**Unpaired t test**	
8	P value	0.0001
9	P value summary	***
10	Significantly different (P < 0.05)?	Yes
11	One- or two-tailed P value?	Two-tailed
12	t, df	t=6.045, df=10
13		
14	**How big is the difference?**	
15	Mean of column B	21741
16	Mean of column C	7506
17	Difference between means (C - B) ± SEM	-14235 ± 2355
18	95% confidence interval	-19482 to -8988
19	R squared (eta squared)	0.7851

Figure 5B Cortisone

Con	Mod	KXN
561.84	12573.34	872.16
1254.64	14499.26	1671.5
1464.98	11121.16	5286.26
3614.3	12333.85	701.53
3824.45	10818.41	5514.75
9208.38	13778.96	3963.77

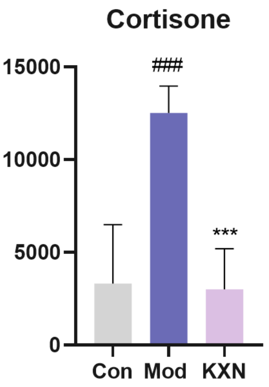

Unpaired t test
Tabular results

1	Table Analyzed	Cortisone
2		
3	Column B	Mod
4	vs.	vs.
5	Column A	Con
6		
7	**Unpaired t test**	
8	P value	<0.0001
9	P value summary	****
10	Significantly different (P < 0.05)?	Yes
11	One- or two-tailed P value?	Two-tailed
12	t, df	t=6.466, df=10
13		
14	**How big is the difference?**	
15	Mean of column A	3321
16	Mean of column B	12521
17	Difference between means (B - A) ± SEM	9199 ± 1423
18	95% confidence interval	6029 to 12369
19	R squared (eta squared)	0.8070

Unpaired t test
Tabular results

1	Table Analyzed	Cortisone
2		
3	Column C	KXN
4	vs.	vs.
5	Column B	Mod
6		
7	**Unpaired t test**	
8	P value	<0.0001
9	P value summary	****
10	Significantly different (P < 0.05)?	Yes
11	One- or two-tailed P value?	Two-tailed
12	t, df	t=8.883, df=10
13		
14	**How big is the difference?**	
15	Mean of column B	12521
16	Mean of column C	3002
17	Difference between means (C - B) ± SEM	-9519 ± 1072
18	95% confidence interval	-11907 to -7132
19	R squared (eta squared)	0.8875

Figure 5B Corticosterone

Con	Mod	KXN
9361.52	137895.8	2458.09
21928.3	142749.7	7298.18
33030.99	105549.5	43891.09
27302.71	151161	3761.31
99352.45	113262.1	74039.3
57784.72	62673.67	36790.27

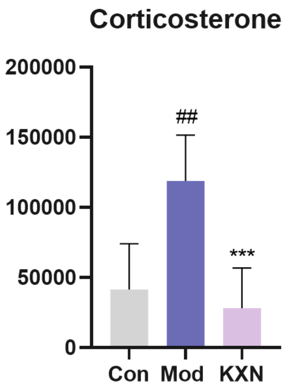

Unpaired t test
Tabular results

1	Table Analyzed	Corticosterone
2		
3	Column B	Mod
4	vs.	vs.
5	Column A	Con
6		
7	**Unpaired t test**	
8	P value	0.0021
9	P value summary	**
10	Significantly different (P < 0.05)?	Yes
11	One- or two-tailed P value?	Two-tailed
12	t, df	t=4.110, df=10
13		
14	**How big is the difference?**	
15	Mean of column A	41460
16	Mean of column B	118882
17	Difference between means (B - A) ± SEM	77422 ± 18835
18	95% confidence interval	35454 to 119390
19	R squared (eta squared)	0.6282

Unpaired t test
Tabular results

1	Table Analyzed	Corticosterone
2		
3	Column C	KXN
4	vs.	vs.
5	Column B	Mod
6		
7	**Unpaired t test**	
8	P value	0.0005
9	P value summary	***
10	Significantly different (P < 0.05)?	Yes
11	One- or two-tailed P value?	Two-tailed
12	t, df	t=5.116, df=10
13		
14	**How big is the difference?**	
15	Mean of column B	118882
16	Mean of column C	28040
17	Difference between means (C - B) ± SEM	-90842 ± 17757
18	95% confidence interval	-130408 to -51276
19	R squared (eta squared)	0.7235

Figure 5B 21-Deoxycortisol

Con	Mod	KXN
61332.33	646204.2	33155.32
79829.44	637818.2	74449.76
46800.47	376964.5	253447.6
120376.1	613875.3	61768.58
196874.2	491285.7	356402.3
360749.6	705692.5	183095.1

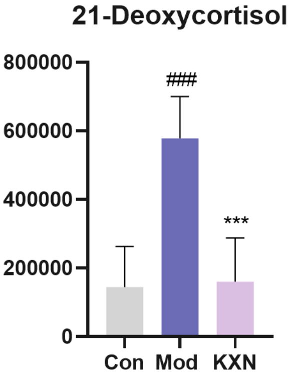

	Unpaired t test<br>Tabular results	
1	Table Analyzed	21-Deoxycortisol
2		
3	Column B	Mod
4	vs.	vs.
5	Column A	Con
6		
7	**Unpaired t test**	
8	P value	<0.0001
9	P value summary	****
10	Significantly different (P < 0.05)?	Yes
11	One- or two-tailed P value?	Two-tailed
12	t, df	t=6.258, df=10
13		
14	**How big is the difference?**	
15	Mean of column A	144327
16	Mean of column B	578640
17	Difference between means (B - A) ± SEM	434313 ± 69399
18	95% confidence interval	279682 to 588944
19	R squared (eta squared)	0.7966

	Unpaired t test<br>Tabular results	
1	Table Analyzed	21-Deoxycortisol
2		
3	Column C	KXN
4	vs.	vs.
5	Column B	Mod
6		
7	**Unpaired t test**	
8	P value	0.0002
9	P value summary	***
10	Significantly different (P < 0.05)?	Yes
11	One- or two-tailed P value?	Two-tailed
12	t, df	t=5.825, df=10
13		
14	**How big is the difference?**	
15	Mean of column B	578640
16	Mean of column C	160386
17	Difference between means (C - B) ± SEM	-418254 ± 71799
18	95% confidence interval	-578231 to -258276
19	R squared (eta squared)	0.7724

Figure 5B Estrone

Con	Mod	KXN
14377.7	13257.49	15002.06
14329.49	11655	13289.86
13940.07	11577.82	12954.93
13497.89	13358.03	13466.19
13047.44	12429.64	15018.22
13366.65	13401.99	14953.72

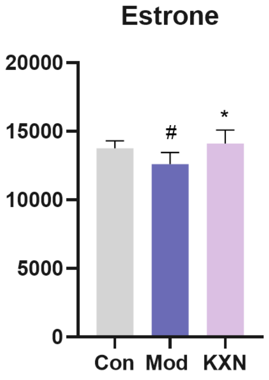

	Unpaired t test<br>Tabular results	
1	Table Analyzed	Estrone
2		
3	Column B	Mod
4	vs.	vs.
5	Column A	Con
6		
7	**Unpaired t test**	
8	P value	0.0193
9	P value summary	*
10	Significantly different (P < 0.05)?	Yes
11	One- or two-tailed P value?	Two-tailed
12	t, df	t=2.785, df=10
13		
14	**How big is the difference?**	
15	Mean of column A	13760
16	Mean of column B	12613
17	Difference between means (B - A) ± SEM	-1147 ± 411.7
18	95% confidence interval	-2064 to -229.2
19	R squared (eta squared)	0.4368

	Unpaired t test<br>Tabular results	
1	Table Analyzed	Estrone
2		
3	Column C	KXN
4	vs.	vs.
5	Column B	Mod
6		
7	**Unpaired t test**	
8	P value	0.0175
9	P value summary	*
10	Significantly different (P < 0.05)?	Yes
11	One- or two-tailed P value?	Two-tailed
12	t, df	t=2.841, df=10
13		
14	**How big is the difference?**	
15	Mean of column B	12613
16	Mean of column C	14114
17	Difference between means (C - B) ± SEM	1501 ± 528.2
18	95% confidence interval	323.9 to 2678
19	R squared (eta squared)	0.4467

Figure 5D

NC	Con	1 µg/mL	10 µg/mL	100 µg/mL	stragaloside	Hyperoside	Icaritin	-Syringares	Baohuoside
38612	40739	42307	44547	45405	43413	41288	44246	41492	41789
38289	39773	41518	42475	44842	42257	41599	43943	41656	41814
37961	40449	42319	42244	46291	41663	42333	45304	42125	42692

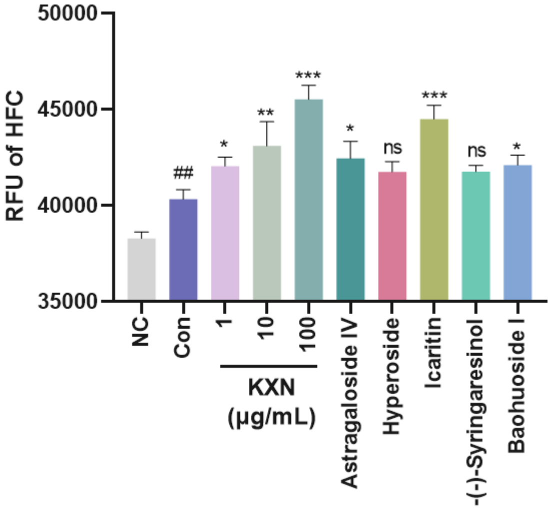

Unpaired t test
Tabular results

1	Table Analyzed	Data 1
2		
3	Column B	Con
4	vs.	vs.
5	Column A	NC
6		
7	**Unpaired t test**	
8	P value	0.0040
9	P value summary	**
10	Significantly different (P < 0.05)?	Yes
11	One- or two-tailed P value?	Two-tailed
12	t, df	t=5.938, df=4
13		
14	**How big is the difference?**	
15	Mean of column A	38287
16	Mean of column B	40320
17	Difference between means (B - A) ± SEM	2033 ± 342.4
18	95% confidence interval	1082 to 2984
19	R squared (eta squared)	0.8981

Ordinary one-way ANOVA
Multiple comparisons

1	Number of families	1				
2	Number of comparisons per family	8				
3	Alpha	0.05				
4						
5	**Dunnett's multiple comparisons test**	**Mean Diff.**	**95.00% CI of diff.**	**Below threshold?**	**Summary**	**Adjusted P Value**
6	Con vs. 1	-1728	-3434 to -21.46	Yes	*	0.0464
7	Con vs. 10	-2768	-4475 to -1062	Yes	**	0.0010
8	Con vs. 100	-5192	-6899 to -3486	Yes	****	<0.0001
9	Con vs. Astragaloside IV	-2124	-3830 to -417.8	Yes	*	0.0113
10	Con vs. Hyperoside	-1420	-3126 to 286.5	No	ns	0.1290
11	Con vs. Icaritin	-4177	-5884 to -2471	Yes	****	<0.0001
12	Con vs. -(-)-Syringaresinol	-1437	-3144 to 268.9	No	ns	0.1220
13	Con vs. Baohuoside I	-1778	-3484 to -71.79	Yes	*	0.0390
14						
15	**Test details**	**Mean 1**	**Mean 2**	**Mean Diff.**	**SE of diff.**	**n1**
16	Con vs. 1	40320	42048	-1728	581.3	3
17	Con vs. 10	40320	43089	-2768	581.3	3
18	Con vs. 100	40320	45513	-5192	581.3	3
19	Con vs. Astragaloside IV	40320	42444	-2124	581.3	3
20	Con vs. Hyperoside	40320	41740	-1420	581.3	3
21	Con vs. Icaritin	40320	44498	-4177	581.3	3
22	Con vs. -(-)-Syringaresinol	40320	41758	-1437	581.3	3

Figure 5E

Con	1 μg/mL	10 μg/mL	100 μg/mL	stragaloside	Hyperoside	Icaritin	-Syringares	Baohuoside
120.7411	190.5665	215.7365	192.5777	199.3168	141.5095	217.803	179.4436	231.3848
103.8052	188.2526	259.7931	261.5829	125.5551	155.9027	198.3885	177.7761	189.9018
112.8387	172.4952	233.9354	246.3006	178.8411	124.3157	188.3484	154.0938	203.3936

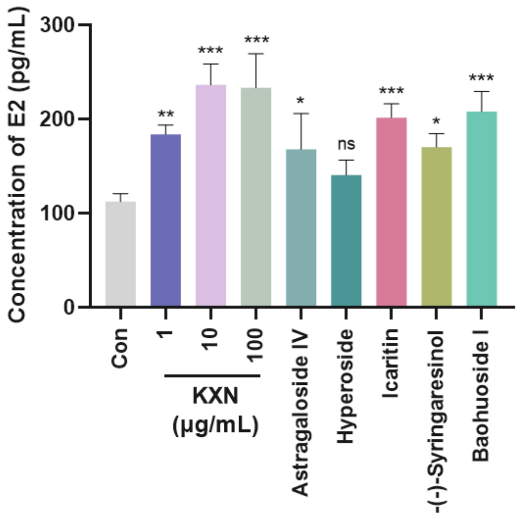

	Ordinary one-way ANOVA<br>Multiple comparisons					
1	Number of families	1				
2	Number of comparisons per family	8				
3	Alpha	0.05				
4						
5	**Dunnett's multiple comparisons test**	**Mean Diff.**	**95.00% CI of diff.**	**Below threshold?**	**Summary**	**Adjusted P Value**
6	Con vs. 1	-71.31	-125.2 to -17.45	Yes	**	0.0069
7	Con vs. 10	-124.0	-177.9 to -70.17	Yes	****	<0.0001
8	Con vs. 100	-121.0	-174.9 to -67.17	Yes	****	<0.0001
9	Con vs. Astragaloside IV	-55.44	-109.3 to -1.583	Yes	*	0.0420
10	Con vs. Hyperoside	-28.11	-81.97 to 25.75	No	ns	0.5386
11	Con vs. Icaritin	-89.05	-142.9 to -35.19	Yes	***	0.0009
12	Con vs. -(-)-Syringaresinol	-57.98	-111.8 to -4.116	Yes	*	0.0317
13	Con vs. Baohuoside I	-95.77	-149.6 to -41.90	Yes	***	0.0004
14						
15	**Test details**	**Mean 1**	**Mean 2**	**Mean Diff.**	**SE of diff.**	**n1**
16	Con vs. 1	112.5	183.8	-71.31	18.35	3
17	Con vs. 10	112.5	236.5	-124.0	18.35	3
18	Con vs. 100	112.5	233.5	-121.0	18.35	3
19	Con vs. Astragaloside IV	112.5	167.9	-55.44	18.35	3
20	Con vs. Hyperoside	112.5	140.6	-28.11	18.35	3
21	Con vs. Icaritin	112.5	201.5	-89.05	18.35	3
22	Con vs. -(-)-Syringaresinol	112.5	170.4	-57.98	18.35	3

Figure 6B

Con	Mod	E2	3 g/kg	6 g/kg	12 g/kg
7.92	4.47	5.95	4.53	4.61	6.59
6.96	3.23	4.21	5.84	5.01	4.29
6.14	3	3.6	3.96	4.57	4.59
7.05	2.95	5.28	6.17	5.03	5.47
6.38	3	4.19	6.03	4.97	4.26
5.96	2.7	4.16	4.98	4.39	4.07

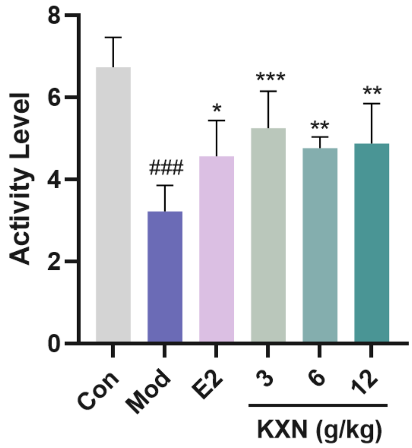

Unpaired t test
Tabular results

1	Table Analyzed	Activity Level
2		
3	Column B	Mod
4	vs.	vs.
5	Column A	Con
6		
7	**Unpaired t test**	
8	P value	<0.0001
9	P value summary	****
10	Significantly different (P < 0.05)?	Yes
11	One- or two-tailed P value?	Two-tailed
12	t, df	t=8.928, df=10
13		
14	**How big is the difference?**	
15	Mean of column A	6.735
16	Mean of column B	3.225
17	Difference between means (B - A) ± SEM	-3.510 ± 0.3932
18	95% confidence interval	-4.386 to -2.634
19	R squared (eta squared)	0.8885

Ordinary one-way ANOVA
Multiple comparisons

1	Number of families	1				
2	Number of comparisons per family	4				
3	Alpha	0.05				
4						
5	**Dunnett's multiple comparisons test**	**Mean Diff.**	**95.00% CI of diff.**	**Below threshold?**	**Summary**	**Adjusted P Value**
6	Mod vs. E2	-1.340	-2.505 to -0.1752	Yes	*	0.0208
7	Mod vs. 3	-2.027	-3.192 to -0.8618	Yes	***	0.0005
8	Mod vs. 6	-1.538	-2.703 to -0.3735	Yes	**	0.0072
9	Mod vs. 12	-1.653	-2.818 to -0.4885	Yes	**	0.0039
10						
11	**Test details**	**Mean 1**	**Mean 2**	**Mean Diff.**	**SE of diff.**	**n1**
12	Mod vs. E2	3.225	4.565	-1.340	0.4468	6
13	Mod vs. 3	3.225	5.252	-2.027	0.4468	6
14	Mod vs. 6	3.225	4.763	-1.538	0.4468	6
15	Mod vs. 12	3.225	4.878	-1.653	0.4468	6

Figure 6C

Con	Mod	E2	3 g/kg	6 g/kg	12 g/kg
0.0516	0.0000	0.0287	0.0611	0.0264	0.0656
0.0740	0.0000	0.0102	0.0436	0.0393	0.0251
0.0931	0.0000	0.0049	0.0860	0.0144	0.0293
0.1569	0.0000	0.0838	0.0289	0.0276	0.0133
0.0287	0.0167	0.0731	0.0500	0.0333	0.0218
0.0409	0.0000	0.0491	0.0520	0.0369	0.0213

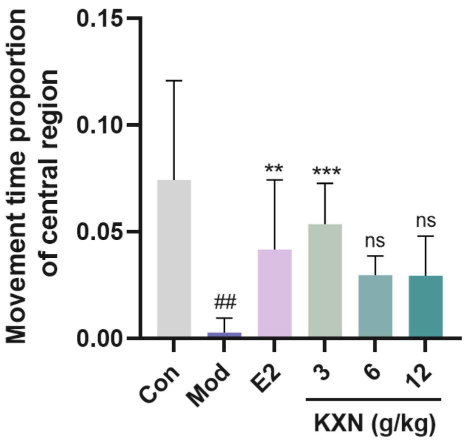

	Unpaired t test<br>Tabular results	
1	Table Analyzed	Movement time proportion of central region
2		
3	Column B	Mod
4	vs.	vs.
5	Column A	Con
6		
7	**Unpaired t test**	
8	P value	0.0040
9	P value summary	**
10	Significantly different (P < 0.05)?	Yes
11	One- or two-tailed P value?	Two-tailed
12	t, df	t=3.709, df=10
13		
14	**How big is the difference?**	
15	Mean of column A	0.07419
16	Mean of column B	0.002778
17	Difference between means (B - A) ± SEM	-0.07141 ± 0.01925
18	95% confidence interval	-0.1143 to -0.02851
19	R squared (eta squared)	0.5790

	Ordinary one-way ANOVA<br>Multiple comparisons					
1	Number of families	1				
2	Number of comparisons per family	4				
3	Alpha	0.05				
4						
5	**Dunnett's multiple comparisons test**	**Mean Diff.**	**95.00% CI of diff.**	**Below threshold?**	**Summary**	**Adjusted P Value**
6	Mod vs. E2	-0.03885	-0.06820 to -0.009504	Yes	**	0.0071
7	Mod vs. 3	-0.05082	-0.08016 to -0.02147	Yes	***	0.0005
8	Mod vs. 6	-0.02689	-0.05623 to 0.002457	No	ns	0.0792
9	Mod vs. 12	-0.02663	-0.05597 to 0.002718	No	ns	0.0831
10						
11	**Test details**	**Mean 1**	**Mean 2**	**Mean Diff.**	**SE of diff.**	**n1**
12	Mod vs. E2	0.002778	0.04163	-0.03885	0.01126	6
13	Mod vs. 3	0.002778	0.05359	-0.05082	0.01126	6
14	Mod vs. 6	0.002778	0.02967	-0.02689	0.01126	6
15	Mod vs. 12	0.002778	0.02941	-0.02663	0.01126	6

Figure 6D

Con	Mod	E2	3 g/kg	6 g/kg	12 g/kg
816.67	758.43	776.97	935.50	705.77	963.57
960.90	640.40	850.50	726.57	796.00	1111.10
1053.33	645.80	775.60	655.50	722.57	984.60
931.57	716.63	1036.53	593.70	782.23	979.23
805.27	566.50	877.23	727.93	792.90	972.20
751.13	458.07	831.20	711.53	702.53	1024.63

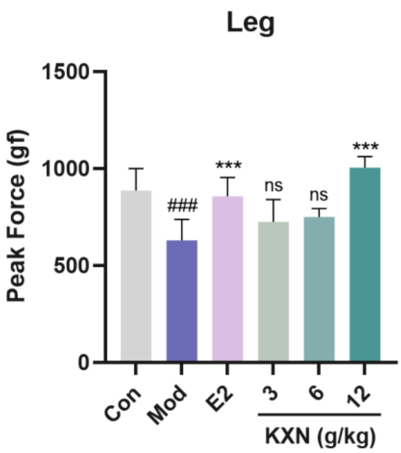

Unpaired t test
Tabular results

1	Table Analyzed	Leg
2		
3	Column B	Mod
4	vs.	vs.
5	Column A	Con
6		
7	**Unpaired t test**	
8	P value	0.0026
9	P value summary	**
10	Significantly different (P < 0.05)?	Yes
11	One- or two-tailed P value?	Two-tailed
12	t, df	t=3.988, df=10
13		
14	**How big is the difference?**	
15	Mean of column A	886.5
16	Mean of column B	631.0
17	Difference between means (B - A) ± SEM	-255.5 ± 64.07
18	95% confidence interval	-398.3 to -112.8
19	R squared (eta squared)	0.6140

Ordinary one-way ANOVA
Multiple comparisons

1	Number of families	1				
2	Number of comparisons per family	4				
3	Alpha	0.05				
4						
5	**Dunnett's multiple comparisons test**	**Mean Diff.**	**95.00% CI of diff.**	**Below threshold?**	**Summary**	**Adjusted P Value**
6	Mod vs. E2	-227.0	-360.4 to -93.63	Yes	***	0.0006
7	Mod vs. 3	-94.15	-227.5 to 39.25	No	ns	0.2250
8	Mod vs. 6	-119.4	-252.8 to 14.04	No	ns	0.0888
9	Mod vs. 12	-374.9	-508.3 to -241.5	Yes	****	<0.0001
10						
11	**Test details**	**Mean 1**	**Mean 2**	**Mean Diff.**	**SE of diff.**	**n1**
12	Mod vs. E2	631.0	858.0	-227.0	51.17	6
13	Mod vs. 3	631.0	725.1	-94.15	51.17	6
14	Mod vs. 6	631.0	750.3	-119.4	51.17	6
15	Mod vs. 12	631.0	1006	-374.9	51.17	6

Figure 6E

Con	Mod	E2	3 g/kg	6 g/kg	12 g/kg
1680.40	1215.67	1500.97	1450.77	1509.03	1672.70
1317.00	1007.17	1357.50	1503.67	1514.70	1633.60
1680.83	1007.33	1598.77	1256.03	1548.37	1640.93
1635.77	1149.40	1375.10	1291.50	1531.27	1665.27
1618.03	1165.10	1386.83	1593.33	1526.27	1702.17
1620.47	1335.40	1536.87	1329.73	1454.47	1682.60

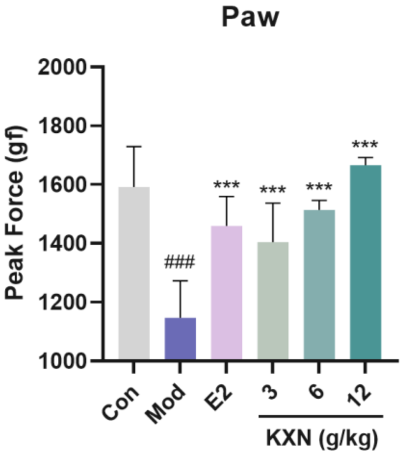

	Unpaired t test<br>Tabular results	
1	Table Analyzed	Paw
2		
3	Column B	Mod
4	vs.	vs.
5	Column A	Con
6		
7	**Unpaired t test**	
8	P value	0.0002
9	P value summary	***
10	Significantly different (P < 0.05)?	Yes
11	One- or two-tailed P value?	Two-tailed
12	t, df	t=5.843, df=10
13		
14	**How big is the difference?**	
15	Mean of column A	1592
16	Mean of column B	1147
17	Difference between means (B - A) ± SEM	-445.4 ± 76.23
18	95% confidence interval	-615.3 to -275.6
19	R squared (eta squared)	0.7734

	Ordinary one-way ANOVA<br>Multiple comparisons					
1	Number of families	1				
2	Number of comparisons per family	4				
3	Alpha	0.05				
4						
5	**Dunnett's multiple comparisons test**	**Mean Diff.**	**95.00% CI of diff.**	**Below threshold?**	**Summary**	**Adjusted P Value**
6	Mod vs. E2	-312.7	-455.8 to -169.5	Yes	****	<0.0001
7	Mod vs. 3	-257.5	-400.6 to -114.4	Yes	***	0.0003
8	Mod vs. 6	-367.3	-510.5 to -224.2	Yes	****	<0.0001
9	Mod vs. 12	-519.5	-662.7 to -376.4	Yes	****	<0.0001
10						
11	**Test details**	**Mean 1**	**Mean 2**	**Mean Diff.**	**SE of diff.**	**n1**
12	Mod vs. E2	1147	1459	-312.7	54.90	6
13	Mod vs. 3	1147	1404	-257.5	54.90	6
14	Mod vs. 6	1147	1514	-367.3	54.90	6
15	Mod vs. 12	1147	1666	-519.5	54.90	6

Figure 6G

Con	Mod	E2	3 g/kg	6 g/kg	12 g/kg
1.5306	1.4727	1.5549	1.4453	1.5294	1.5164
1.5543	1.4492	1.4795	1.5210	1.5355	1.5261
1.5102	1.4546	1.5045	1.5187	1.4916	1.5441
1.5336	1.4483	1.5333	1.5141	1.5216	1.5017
1.5703	1.4766	1.5071	1.5115	1.4891	1.5293
1.5254	1.4581	1.5674	1.4738	1.5289	1.5199

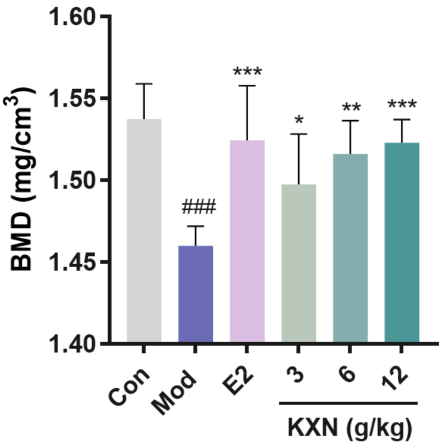

	Unpaired t test	
1	Table Analyzed	BMD
2		
3	Column B	Mod
4	vs.	vs.
5	Column A	Con
6		
7	**Unpaired t test**	
8	P value	<0.0001
9	P value summary	****
10	Significantly different (P < 0.05)?	Yes
11	One- or two-tailed P value?	Two-tailed
12	t, df	t=7.706, df=10
13		
14	**How big is the difference?**	
15	Mean of column A	1.537
16	Mean of column B	1.460
17	Difference between means (B - A) ± SEM	-0.07751 ± 0.01006
18	95% confidence interval	-0.09992 to -0.05510
19	R squared (eta squared)	0.8559

	Ordinary one-way ANOVA<br>Multiple comparisons					
1	Number of families	1				
2	Number of comparisons per family	4				
3	Alpha	0.05				
4						
5	**Dunnett's multiple comparisons test**	**Mean Diff.**	**95.00% CI of diff.**	**Below threshold?**	**Summary**	**Adjusted P Value**
6	Mod vs. E2	-0.06455	-0.1003 to -0.02878	Yes	***	0.0003
7	Mod vs. 3	-0.03748	-0.07325 to -0.001709	Yes	*	0.0381
8	Mod vs. 6	-0.05609	-0.09186 to -0.02032	Yes	**	0.0015
9	Mod vs. 12	-0.06300	-0.09877 to -0.02723	Yes	***	0.0004
10						
11	**Test details**	**Mean 1**	**Mean 2**	**Mean Diff.**	**SE of diff.**	**n1**
12	Mod vs. E2	1.460	1.524	-0.06455	0.01372	6
13	Mod vs. 3	1.460	1.497	-0.03748	0.01372	6
14	Mod vs. 6	1.460	1.516	-0.05609	0.01372	6
15	Mod vs. 12	1.460	1.523	-0.06300	0.01372	6

Figure 6H

Con	Mod	E2	3 g/kg	6 g/kg	12 g/kg
84.16	73.27	83.26	73.65	82.56	81.52
92.97	77.04	81.42	89.03	79.99	78.93
90.31	74.14	79.79	78.41	77.88	78.11
84.24	77.54	81.74	77.31	78.46	78.64
91.32	77.27	82.27	73.39	76.52	79.02
93.49	74.58	81.32	90.27	81.23	83.79

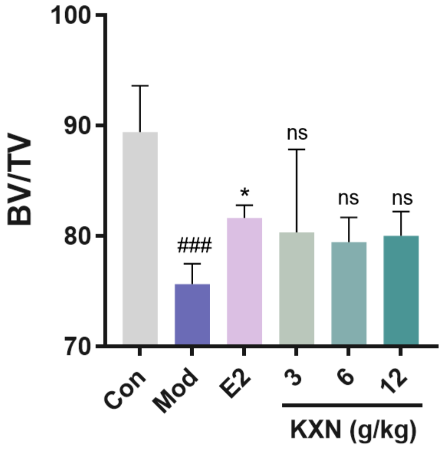

	Unpaired t test<br>Tabular results	
1	Table Analyzed	BV/TV
2		
3	Column B	Mod
4	vs.	vs.
5	Column A	Con
6		
7	**Unpaired t test**	
8	P value	<0.0001
9	P value summary	****
10	Significantly different (P < 0.05)?	Yes
11	One- or two-tailed P value?	Two-tailed
12	t, df	t=7.353, df=10
13		
14	**How big is the difference?**	
15	Mean of column A	89.42
16	Mean of column B	75.64
17	Difference between means (B - A) ± SEM	-13.78 ± 1.873
18	95% confidence interval	-17.95 to -9.601
19	R squared (eta squared)	0.8439

	Ordinary one-way ANOVA<br>Multiple comparisons					
1	Number of families	1				
2	Number of comparisons per family	4				
3	Alpha	0.05				
4						
5	**Dunnett's multiple comparisons test**	**Mean Diff.**	**95.00% CI of diff.**	**Below threshold?**	**Summary**	**Adjusted P Value**
6	Mod vs. E2	-5.993	-11.65 to -0.3351	Yes	*	0.0356
7	Mod vs. 3	-4.703	-10.36 to 0.9549	No	ns	0.1234
8	Mod vs. 6	-3.800	-9.458 to 1.858	No	ns	0.2618
9	Mod vs. 12	-4.362	-10.02 to 1.297	No	ns	0.1662
10						
11	**Test details**	**Mean 1**	**Mean 2**	**Mean Diff.**	**SE of diff.**	**n1**
12	Mod vs. E2	75.64	81.63	-5.993	2.170	6
13	Mod vs. 3	75.64	80.34	-4.703	2.170	6
14	Mod vs. 6	75.64	79.44	-3.800	2.170	6
15	Mod vs. 12	75.64	80.00	-4.362	2.170	6

Figure 6I

Con	Mod	E2	3 g/kg	6 g/kg	12 g/kg
0.5429	1.1300	0.6747	1.0106	0.8253	0.8447
0.5267	0.9484	0.7419	0.5777	0.8263	0.7639
0.4270	0.9220	0.7065	1.1210	0.9494	0.8721
0.4985	0.9976	0.9986	1.0180	0.7231	0.7198
0.5705	1.0339	0.8101	1.0352	0.8540	0.9296
0.4065	0.9640	0.8750	0.5793	0.9127	0.9387

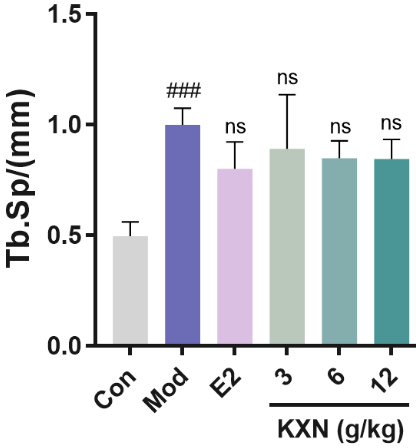

	Unpaired t test<br>Tabular results	
1	Table Analyzed	BV/TV
2		
3	Column B	Mod
4	vs.	vs.
5	Column A	Con
6		
7	**Unpaired t test**	
8	P value	<0.0001
9	P value summary	****
10	Significantly different (P < 0.05)?	Yes
11	One- or two-tailed P value?	Two-tailed
12	t, df	t=7.353, df=10
13		
14	**How big is the difference?**	
15	Mean of column A	89.42
16	Mean of column B	75.64
17	Difference between means (B - A) ± SEM	-13.78 ± 1.873
18	95% confidence interval	-17.95 to -9.601
19	R squared (eta squared)	0.8439

	Ordinary one-way ANOVA<br>Multiple comparisons					
1	Number of families	1				
2	Number of comparisons per family	4				
3	Alpha	0.05				
4						
5	**Dunnett's multiple comparisons test**	**Mean Diff.**	**95.00% CI of diff.**	**Below threshold?**	**Summary**	**Adjusted P Value**
6	Mod vs. E2	0.1982	-0.008315 to 0.4047	No	ns	0.0625
7	Mod vs. 3	0.1090	-0.09748 to 0.3155	No	ns	0.4620
8	Mod vs. 6	0.1509	-0.05565 to 0.3573	No	ns	0.2010
9	Mod vs. 12	0.1545	-0.05198 to 0.3610	No	ns	0.1850
10						
11	**Test details**	**Mean 1**	**Mean 2**	**Mean Diff.**	**SE of diff.**	**n1**
12	Mod vs. E2	0.9993	0.8011	0.1982	0.07921	6
13	Mod vs. 3	0.9993	0.8903	0.1090	0.07921	6
14	Mod vs. 6	0.9993	0.8485	0.1509	0.07921	6
15	Mod vs. 12	0.9993	0.8448	0.1545	0.07921	6

Figure 6J

Con	Mod	E2	3 g/kg	6 g/kg	12 g/kg
0.4339	0.3023	0.4768	0.3438	0.3947	0.3546
0.4034	0.3464	0.4004	0.2718	0.4754	0.3733
0.4578	0.2793	0.3615	0.4282	0.2900	0.3981
0.3532	0.3243	0.3784	0.4442	0.4520	0.4670
0.3388	0.2668	0.4565	0.2934	0.4387	0.4366
0.3277	0.2798	0.4377	0.2556	0.3030	0.4305

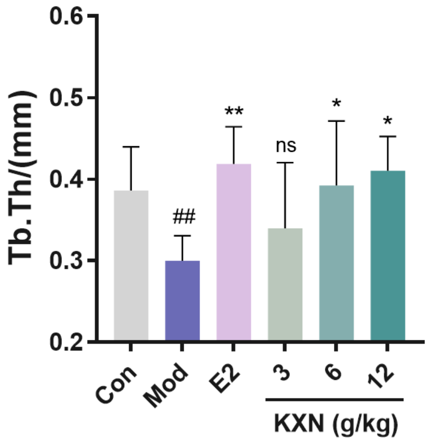

Unpaired t test
Tabular results

1	Table Analyzed	Tb.Th
2		
3	Column B	Mod
4	vs.	vs.
5	Column A	Con
6		
7	**Unpaired t test**	
8	P value	0.0067
9	P value summary	**
10	Significantly different (P < 0.05)?	Yes
11	One- or two-tailed P value?	Two-tailed
12	t, df	t=3.404, df=10
13		
14	**How big is the difference?**	
15	Mean of column A	0.3858
16	Mean of column B	0.2998
17	Difference between means (B - A) ± SEN	-0.08598 ± 0.02526
18	95% confidence interval	-0.1423 to -0.02970
19	R squared (eta squared)	0.5368

Ordinary one-way ANOVA
Multiple comparisons

1	Number of families	1				
2	Number of comparisons per family	4				
3	Alpha	0.05				
4						
5	**Dunnett's multiple comparisons test**	**Mean Diff.**	**95.00% CI of diff.**	**Below threshold?**	**Summary**	**Adjusted P Value**
6	Mod vs. E2	-0.1187	-0.2079 to -0.02959	Yes	**	0.0067
7	Mod vs. 3	-0.03968	-0.1288 to 0.04946	No	ns	0.6046
8	Mod vs. 6	-0.09248	-0.1816 to -0.003342	Yes	*	0.0404
9	Mod vs. 12	-0.1102	-0.1993 to -0.02106	Yes	*	0.0123
10						
11	**Test details**	**Mean 1**	**Mean 2**	**Mean Diff.**	**SE of diff.**	**n1**
12	Mod vs. E2	0.2998	0.4186	-0.1187	0.03419	6
13	Mod vs. 3	0.2998	0.3395	-0.03968	0.03419	6
14	Mod vs. 6	0.2998	0.3923	-0.09248	0.03419	6
15	Mod vs. 12	0.2998	0.4100	-0.1102	0.03419	6

Supplementary Figure S10-Adrenal

Con	Mod	E2	3 g/kg	6 g/kg	12 g/kg
45.48611	30.95679	35.47068	38.22917	38.3912	39.1821
43.37963	33.54552	32.72377	37.28781	37.53086	41.99846
41.18056	34.48302	31.67438	36.43904	41.14198	42.12191
43.08256	33.1713	34.43673	33.14429	35.58256	43.3642
45.5054	35.80247	33.97377	35.6713	36.08796	36.59722
46.51235	36.46991	37.27238	24.84954	34.00463	40.57099

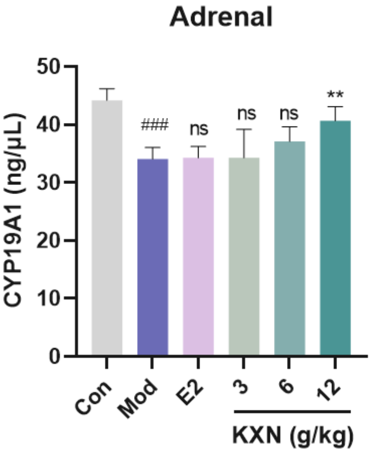

	Unpaired t test<br>Tabular results	
1	Table Analyzed	Adrenal
2		
3	Column B	Mod
4	vs.	vs.
5	Column A	Con
6		
7	**Unpaired t test**	
8	P value	<0.0001
9	P value summary	****
10	Significantly different (P < 0.05)?	Yes
11	One- or two-tailed P value?	Two-tailed
12	t, df	t=8.825, df=10
13		
14	**How big is the difference?**	
15	Mean of column A	44.19
16	Mean of column B	34.07
17	Difference between means (B - A) ± SEM	-10.12 ± 1.147
18	95% confidence interval	-12.67 to -7.565
19	R squared (eta squared)	0.8862

	Ordinary one-way ANOVA<br>Multiple comparisons					
1	Number of families	1				
2	Number of comparisons per family	4				
3	Alpha	0.05				
4						
5	**Dunnett's multiple comparisons test**	**Mean Diff.**	**95.00% CI of diff.**	**Below threshold?**	**Summary**	**Adjusted P Value**
6	Mod vs. E2	-0.1871	-4.672 to 4.297	No	ns	0.9999
7	Mod vs. 3	-0.1987	-4.683 to 4.286	No	ns	0.9998
8	Mod vs. 6	-3.052	-7.536 to 1.433	No	ns	0.2518
9	Mod vs. 12	-6.568	-11.05 to -2.083	Yes	**	0.0029
10						
11	**Test details**	**Mean 1**	**Mean 2**	**Mean Diff.**	**SE of diff.**	**n1**
12	Mod vs. E2	34.07	34.26	-0.1871	1.720	6
13	Mod vs. 3	34.07	34.27	-0.1987	1.720	6
14	Mod vs. 6	34.07	37.12	-3.052	1.720	6
15	Mod vs. 12	34.07	40.64	-6.568	1.720	6

Supplementary Figure S10- Uterus

Con	Mod	E2	3 g/kg	6 g/kg	12 g/kg
13.94676	13.07099	12.74306	12.0409	16.15934	12.71605
14.97878	12.59066	13.04977	14.20525	15.02122	14.49846
13.11343	12.65432	12.3939	11.39082	13.18094	15.38387
14.48881	12.19522	11.92708	13.95448	12.94946	14.96721
15.50154	12.88387	13.61304	11.63387	14.22261	13.89468
16.16898	11.2635	11.21914	11.45255	12.37269	15.72145

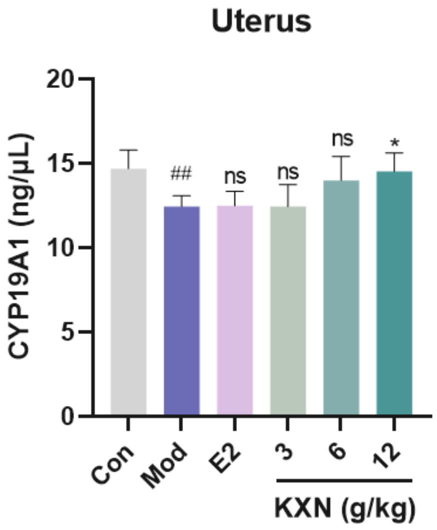

Unpaired t test
Tabular results

1	Table Analyzed	Uterus
2		
3	Column B	Mod
4	vs.	vs.
5	Column A	Con
6		
7	**Unpaired t test**	
8	P value	0.0015
9	P value summary	**
10	Significantly different (P < 0.05)?	Yes
11	One- or two-tailed P value?	Two-tailed
12	t, df	t=4.339, df=10
13		
14	**How big is the difference?**	
15	Mean of column A	14.70
16	Mean of column B	12.44
17	Difference between means (B - A) ± SEN	-2.257 ± 0.5201
18	95% confidence interval	-3.415 to -1.098
19	R squared (eta squared)	0.6531

Ordinary one-way ANOVA
Multiple comparisons

1	Number of families	1				
2	Number of comparisons per family	4				
3	Alpha	0.05				
4						
5	**Dunnett's multiple comparisons test**	**Mean Diff.**	**95.00% CI of diff.**	**Below threshold?**	**Summary**	**Adjusted P Value**
6	Mod vs. E2	-0.04791	-1.702 to 1.606	No	ns	0.9999
7	Mod vs. 3	-0.003218	-1.657 to 1.651	No	ns	>0.9999
8	Mod vs. 6	-1.541	-3.195 to 0.1129	No	ns	0.0729
9	Mod vs. 12	-2.087	-3.741 to -0.4330	Yes	*	0.0105
10						
11	**Test details**	**Mean 1**	**Mean 2**	**Mean Diff.**	**SE of diff.**	**n1**
12	Mod vs. E2	12.44	12.49	-0.04791	0.6345	6
13	Mod vs. 3	12.44	12.45	-0.003218	0.6345	6
14	Mod vs. 6	12.44	13.98	-1.541	0.6345	6
15	Mod vs. 12	12.44	14.53	-2.087	0.6345	6

Supplementary Figure S10- Hypothalamus

Con	Mod	E2	3 g/kg	6 g/kg	12 g/kg
15.625	12.90123	12.77585	13.19637	14.85147	15.63079
16.98495	11.36188	14.33449	13.98341	15.41088	14.35571
16.40239	11.7419	13.41049	11.30594	14.96335	13.59568
15.1061	13.58796	11.9946	13.53974	14.3885	14.87076
15.99344	10.9973	13.09028	11.98495	12.64853	15.05594
14.9267	13.2909	12.30903	13.03241	14.57948	14.23804

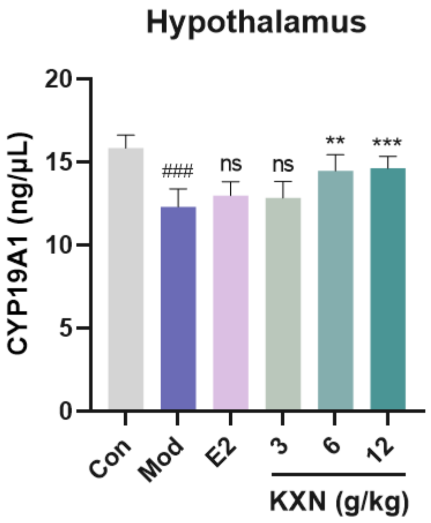

Unpaired t test
Tabular results

1	Table Analyzed	Hypothalamus
2		
3	Column B	Mod
4	vs.	vs.
5	Column A	Con
6		
7	**Unpaired t test**	
8	P value	<0.0001
9	P value summary	****
10	Significantly different (P < 0.05)?	Yes
11	One- or two-tailed P value?	Two-tailed
12	t, df	t=6.453, df=10
13		
14	**How big is the difference?**	
15	Mean of column A	15.84
16	Mean of column B	12.31
17	Difference between means (B - A) ± SEN	-3.526 ± 0.5465
18	95% confidence interval	-4.744 to -2.309
19	R squared (eta squared)	0.8063

Ordinary one-way ANOVA
Multiple comparisons

1	Number of families	1				
2	Number of comparisons per family	4				
3	Alpha	0.05				
4						
5	**Dunnett's multiple comparisons test**	**Mean Diff.**	**95.00% CI of diff.**	**Below threshold?**	**Summary**	**Adjusted P Value**
6	Mod vs. E2	-0.6723	-2.071 to 0.7262	No	ns	0.5419
7	Mod vs. 3	-0.5269	-1.925 to 0.8715	No	ns	0.7259
8	Mod vs. 6	-2.160	-3.559 to -0.7617	Yes	**	0.0017
9	Mod vs. 12	-2.311	-3.709 to -0.9125	Yes	***	0.0008
10						
11	**Test details**	**Mean 1**	**Mean 2**	**Mean Diff.**	**SE of diff.**	**n1**
12	Mod vs. E2	12.31	12.99	-0.6723	0.5364	6
13	Mod vs. 3	12.31	12.84	-0.5269	0.5364	6
14	Mod vs. 6	12.31	14.47	-2.160	0.5364	6
15	Mod vs. 12	12.31	14.62	-2.311	0.5364	6

Supplementary Figure S11A

Con	SiNC	SiCYP19A1-1	SiCYP19A1-2	SiCYP19A1-3	SiCYP19A1-4
1.000064	0.913955	0.813672	0.583704	0.321948	0.346941
1.000021	0.906161	0.678354	0.568762	0.289797	0.444273
0.999958	1.009737	0.84217	0.5854	0.336434	0.366518

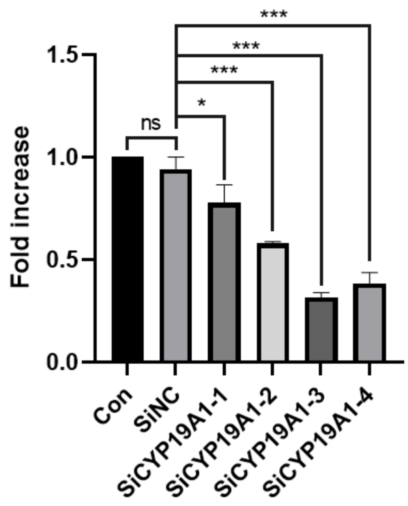

	Unpaired t test<br>Tabular results	
1	Table Analyzed	Data 1
2		
3	Column B	SiNC
4	vs.	vs.
5	Column A	Con
6		
7	**Unpaired t test**	
8	P value	0.1637
9	P value summary	ns
10	Significantly different (P < 0.05)?	No
11	One- or two-tailed P value?	Two-tailed
12	t, df	t=1.703, df=4
13		
14	**How big is the difference?**	
15	Mean of column A	1.000
16	Mean of column B	0.9433
17	Difference between means (B - A) ± SEM	-0.05673 ± 0.03330
18	95% confidence interval	-0.1492 to 0.03573
19	R squared (eta squared)	0.4204

	Ordinary one-way ANOVA<br>Multiple comparisons					
1	Number of families	1				
2	Number of comparisons per family	4				
3	Alpha	0.05				
4						
5	**Dunnett's multiple comparisons test**	**Mean Diff.**	**95.00% CI of diff.**	**Below threshold?**	**Summary**	**Adjusted P Value**
6	SiNC vs. SiCYP19A1-1	0.1652	0.03904 to 0.2914	Yes	*	0.0117
7	SiNC vs. SiCYP19A1-2	0.3640	0.2378 to 0.4902	Yes	****	<0.0001
8	SiNC vs. SiCYP19A1-3	0.6272	0.5010 to 0.7534	Yes	****	<0.0001
9	SiNC vs. SiCYP19A1-4	0.5574	0.4312 to 0.6836	Yes	****	<0.0001
10						
11	**Test details**	**Mean 1**	**Mean 2**	**Mean Diff.**	**SE of diff.**	**n1**
12	SiNC vs. SiCYP19A1-1	0.9433	0.7781	0.1652	0.04365	3
13	SiNC vs. SiCYP19A1-2	0.9433	0.5793	0.3640	0.04365	3
14	SiNC vs. SiCYP19A1-3	0.9433	0.3161	0.6272	0.04365	3
15	SiNC vs. SiCYP19A1-4	0.9433	0.3859	0.5574	0.04365	3

Supplementary Figure S11B

Con	SiNC	P19A1 knockdc	1 μg/mL	10 μg/mL	100 μg/mL	Icaritin	Baohuoside I	Astragaloside IV
100.0189	97.35849	99.09434	94.18868	103.2075	89.32075	94.77358	100.4528	91.88679
101.9434	95.88679	95.79245	97.66038	102.0377	103.8302	100.7736	97.83019	84.64151
98.0566	108.5849	87.83019	111.4717	91.62264	103.6604	96.56604	92.03774	100.6604

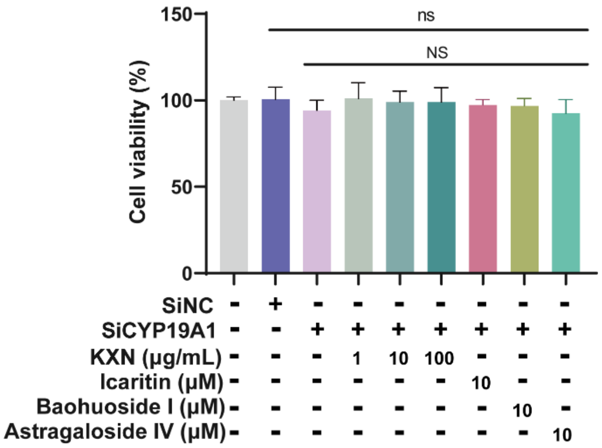

Ordinary one-way ANOVA
Multiple comparisons

1	Number of families	1				
2	Number of comparisons per family	8				
3	Alpha	0.05				
4						
5	**Dunnett's multiple comparisons test**	**Mean Diff.**	**95.00% CI of diff.**	**Below threshold?**	**Summary**	**Adjusted P Value**
6	Con vs. SiNC	-0.6038	-16.00 to 14.80	No	ns	0.9999
7	Con vs. CYP19A1 knockdown	5.767	-9.632 to 21.17	No	ns	0.8243
8	Con vs. 1	-1.101	-16.50 to 14.30	No	ns	0.9997
9	Con vs. 10	1.050	-14.35 to 16.45	No	ns	0.9997
10	Con vs. 100	1.069	-14.33 to 16.47	No	ns	0.9997
11	Con vs. Icaritin	2.635	-12.76 to 18.03	No	ns	0.9969
12	Con vs. Baohuoside I	3.233	-12.17 to 18.63	No	ns	0.9900
13	Con vs. Astragaloside IV	7.610	-7.789 to 23.01	No	ns	0.5932
14						
15	**Test details**	**Mean 1**	**Mean 2**	**Mean Diff.**	**SE of diff.**	**n1**
16	Con vs. SiNC	100.0	100.6	-0.6038	5.246	3
17	Con vs. CYP19A1 knockdown	100.0	94.24	5.767	5.246	3
18	Con vs. 1	100.0	101.1	-1.101	5.246	3
19	Con vs. 10	100.0	98.96	1.050	5.246	3
20	Con vs. 100	100.0	98.94	1.069	5.246	3
21	Con vs. Icaritin	100.0	97.37	2.635	5.246	3
22	Con vs. Baohuoside I	100.0	96.77	3.233	5.246	3

Ordinary one-way ANOVA
Multiple comparisons

1	Number of families	1				
2	Number of comparisons per family	7				
3	Alpha	0.05				
4						
5	**Dunnett's multiple comparisons test**	**Mean Diff.**	**95.00% CI of diff.**	**Below threshold?**	**Summary**	**Adjusted P Value**
6	SiNC vs. CYP19A1 knockdown	6.371	-9.816 to 22.56	No	ns	0.7618
7	SiNC vs. 1	-0.4969	-16.68 to 15.69	No	ns	0.9999
8	SiNC vs. 10	1.654	-14.53 to 17.84	No	ns	0.9996
9	SiNC vs. 100	1.673	-14.51 to 17.86	No	ns	0.9996
10	SiNC vs. Icaritin	3.239	-12.95 to 19.43	No	ns	0.9875
11	SiNC vs. Baohuoside I	3.836	-12.35 to 20.02	No	ns	0.9692
12	SiNC vs. Astragaloside IV	8.214	-7.973 to 24.40	No	ns	0.5408
13						
14	**Test details**	**Mean 1**	**Mean 2**	**Mean Diff.**	**SE of diff.**	**n1**
15	SiNC vs. CYP19A1 knockdown	100.6	94.24	6.371	5.536	3
16	SiNC vs. 1	100.6	101.1	-0.4969	5.536	3
17	SiNC vs. 10	100.6	98.96	1.654	5.536	3
18	SiNC vs. 100	100.6	98.94	1.673	5.536	3
19	SiNC vs. Icaritin	100.6	97.37	3.239	5.536	3
20	SiNC vs. Baohuoside I	100.6	96.77	3.836	5.536	3
21	SiNC vs. Astragaloside IV	100.6	92.40	8.214	5.536	3

Supplementary Figure S11C

Con	SiNC	P19A1 knockdc	1 µg/mL	10 µg/mL	100 µg/mL	Icaritin	Baohuoside I	Astragaloside IV
7.61029	7.6825	6.656888	6.723141	6.84644	6.293016	6.68848	6.500056	6.773666
7.754711	7.227801	6.584678	6.439129	6.810899	6.522081	6.136748	6.397947	6.61627
7.125127	7.718605	6.491594	6.574523	6.110234	6.774128	6.630125	6.706826	6.713302

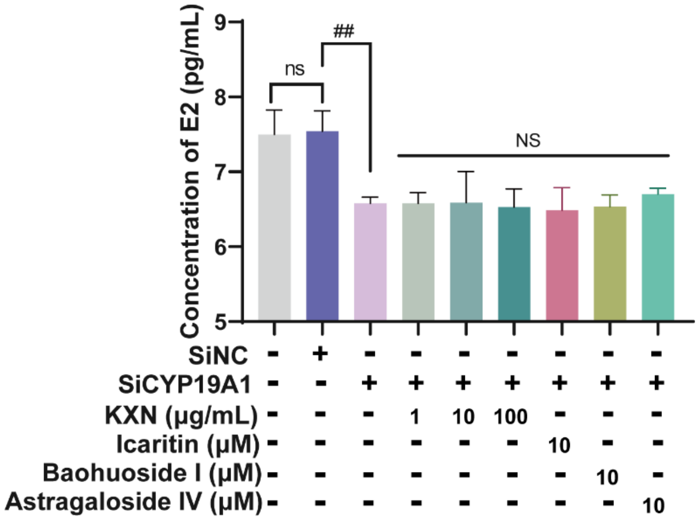

Unpaired t test
Tabular results

		Data 1
1	Table Analyzed	Data 1
2		
3	Column B	SiNC
4	vs.	vs.
5	Column A	Con
6		
7	**Unpaired t test**	
8	P value	0.8608
9	P value summary	ns
10	Significantly different (P < 0.05)?	No
11	One- or two-tailed P value?	Two-tailed
12	t, df	t=0.1870, df=4
13		
14	**How big is the difference?**	
15	Mean of column A	7.497
16	Mean of column B	7.543
17	Difference between means (B - A) ± SEN	0.04626 ± 0.2474
18	95% confidence interval	-0.6406 to 0.7331
19	R squared (eta squared)	0.008666

Unpaired t test
Tabular results

		Data 1
1	Table Analyzed	Data 1
2		
3	Column C	CYP19A1 knockdow
4	vs.	vs.
5	Column B	SiNC
6		
7	**Unpaired t test**	
8	P value	0.0043
9	P value summary	**
10	Significantly different (P < 0.05)?	Yes
11	One- or two-tailed P value?	Two-tailed
12	t, df	t=5.849, df=4
13		
14	**How big is the difference?**	
15	Mean of column B	7.543
16	Mean of column C	6.578
17	Difference between means (C - B) ± SEN	-0.9652 ± 0.1650
18	95% confidence interval	-1.423 to -0.5071
19	R squared (eta squared)	0.8953

Ordinary one-way ANOVA
Multiple comparisons

1	Number of families	1				
2	Number of comparisons per family	6				
3	Alpha	0.05				
4						
5	**Dunnett's multiple comparisons test**	**Mean Diff.**	**95.00% CI of diff.**	**Below threshold?**	**Summary**	**Adjusted P Value**
6	CYP19A1 knockdown vs. 1	-0.001211	-0.5555 to 0.5531	No	ns	>0.9999
7	CYP19A1 knockdown vs. 10	-0.01147	-0.5657 to 0.5428	No	ns	>0.9999
8	CYP19A1 knockdown vs. 100	0.04798	-0.5063 to 0.6022	No	ns	0.9996
9	CYP19A1 knockdown vs. Icaritin	0.09260	-0.4617 to 0.6469	No	ns	0.9914
10	CYP19A1 knockdown vs. Baohuoside I	0.04278	-0.5115 to 0.5970	No	ns	0.9997
11	CYP19A1 knockdown vs. Astragaloside I\	-0.1234	-0.6776 to 0.4309	No	ns	0.9653
12						
13	**Test details**	**Mean 1**	**Mean 2**	**Mean Diff.**	**SE of diff.**	**n1**
14	CYP19A1 knockdown vs. 1	6.578	6.579	-0.001211	0.1903	3
15	CYP19A1 knockdown vs. 10	6.578	6.589	-0.01147	0.1903	3
16	CYP19A1 knockdown vs. 100	6.578	6.530	0.04798	0.1903	3
17	CYP19A1 knockdown vs. Icaritin	6.578	6.485	0.09260	0.1903	3
18	CYP19A1 knockdown vs. Baohuoside I	6.578	6.535	0.04278	0.1903	3
19	CYP19A1 knockdown vs. Astragaloside I\	6.578	6.701	-0.1234	0.1903	3
